# Youth Risk Behavior Surveillance — United States, 2017

**DOI:** 10.15585/mmwr.ss6708a1

**Published:** 2018-06-15

**Authors:** Laura Kann, Tim McManus, William A. Harris, Shari L. Shanklin, Katherine H. Flint, Barbara Queen, Richard Lowry, David Chyen, Lisa Whittle, Jemekia Thornton, Connie Lim, Denise Bradford, Yoshimi Yamakawa, Michelle Leon, Nancy Brener, Kathleen A. Ethier

**Affiliations:** ^1^*Division of Adolescent and School Health, National Center for HIV/AIDS, Viral Hepatitis, STD, and TB Prevention, CDC, Atlanta, GA;* ^2^*ICF International, Rockville, Maryland;* ^3^*Westat, Rockville, Maryland*

## Abstract

**Problem:**

Health-risk behaviors contribute to the leading causes of morbidity and mortality among youth and adults in the United States. In addition, significant health disparities exist among demographic subgroups of youth defined by sex, race/ethnicity, and grade in school and between sexual minority and nonsexual minority youth. Population-based data on the most important health-related behaviors at the national, state, and local levels can be used to help monitor the effectiveness of public health interventions designed to protect and promote the health of youth at the national, state, and local levels.

**Reporting Period Covered:**

September 2016–December 2017.

**Description of the System:**

The Youth Risk Behavior Surveillance System (YRBSS) monitors six categories of priority health-related behaviors among youth and young adults: 1) behaviors that contribute to unintentional injuries and violence; 2) tobacco use; 3) alcohol and other drug use; 4) sexual behaviors related to unintended pregnancy and sexually transmitted infections (STIs), including human immunodeficiency virus (HIV) infection; 5) unhealthy dietary behaviors; and 6) physical inactivity. In addition, YRBSS monitors the prevalence of other health-related behaviors, obesity, and asthma. YRBSS includes a national school-based Youth Risk Behavior Survey (YRBS) conducted by CDC and state and large urban school district school-based YRBSs conducted by state and local education and health agencies. Starting with the 2015 YRBSS cycle, a question to ascertain sexual identity and a question to ascertain sex of sexual contacts were added to the national YRBS questionnaire and to the standard YRBS questionnaire used by the states and large urban school districts as a starting point for their questionnaires. This report summarizes results from the 2017 national YRBS for 121 health-related behaviors and for obesity, overweight, and asthma by demographic subgroups defined by sex, race/ethnicity, and grade in school and by sexual minority status; updates the numbers of sexual minority students nationwide; and describes overall trends in health-related behaviors during 1991–2017. This reports also summarizes results from 39 state and 21 large urban school district surveys with weighted data for the 2017 YRBSS cycle by sex and sexual minority status (where available).

**Results:**

Results from the 2017 national YRBS indicated that many high school students are engaged in health-risk behaviors associated with the leading causes of death among persons aged 10–24 years in the United States. During the 30 days before the survey, 39.2% of high school students nationwide (among the 62.8% who drove a car or other vehicle during the 30 days before the survey) had texted or e-mailed while driving, 29.8% reported current alcohol use, and 19.8% reported current marijuana use. In addition, 14.0% of students had taken prescription pain medicine without a doctor’s prescription or differently than how a doctor told them to use it one or more times during their life. During the 12 months before the survey, 19.0% had been bullied on school property and 7.4% had attempted suicide. Many high school students are engaged in sexual risk behaviors that relate to unintended pregnancies and STIs, including HIV infection. Nationwide, 39.5% of students had ever had sexual intercourse and 9.7% had had sexual intercourse with four or more persons during their life. Among currently sexually active students, 53.8% reported that either they or their partner had used a condom during their last sexual intercourse. Results from the 2017 national YRBS also indicated many high school students are engaged in behaviors associated with chronic diseases, such as cardiovascular disease, cancer, and diabetes. Nationwide, 8.8% of high school students had smoked cigarettes and 13.2% had used an electronic vapor product on at least 1 day during the 30 days before the survey. Forty-three percent played video or computer games or used a computer for 3 or more hours per day on an average school day for something that was not school work and 15.4% had not been physically active for a total of at least 60 minutes on at least 1 day during the 7 days before the survey. Further, 14.8% had obesity and 15.6% were overweight. The prevalence of most health-related behaviors varies by sex, race/ethnicity, and, particularly, sexual identity and sex of sexual contacts. Specifically, the prevalence of many health-risk behaviors is significantly higher among sexual minority students compared with nonsexual minority students. Nonetheless, analysis of long-term temporal trends indicates that the overall prevalence of most health-risk behaviors has moved in the desired direction.

**Interpretation:**

Most high school students cope with the transition from childhood through adolescence to adulthood successfully and become healthy and productive adults. However, this report documents that some subgroups of students defined by sex, race/ethnicity, grade in school, and especially sexual minority status have a higher prevalence of many health-risk behaviors that might place them at risk for unnecessary or premature mortality, morbidity, and social problems (e.g., academic failure, poverty, and crime).

**Public Health Action:**

YRBSS data are used widely to compare the prevalence of health-related behaviors among subpopulations of students; assess trends in health-related behaviors over time; monitor progress toward achieving 21 national health objectives; provide comparable state and large urban school district data; and take public health actions to decrease health-risk behaviors and improve health outcomes among youth. Using this and other reports based on scientifically sound data is important for raising awareness about the prevalence of health-related behaviors among students in grades 9–12, especially sexual minority students, among decision makers, the public, and a wide variety of agencies and organizations that work with youth. These agencies and organizations, including schools and youth-friendly health care providers, can help facilitate access to critically important education, health care, and high-impact, evidence-based interventions.

## Introduction

In 2016 in the United States, 74% of all deaths among persons aged 10–24 years resulted from four causes: motor vehicle crashes (22%), other unintentional injuries (20%), suicide (17%), and homicide (15%) (*1*). Among persons aged 15–19 years, 209,809 births (*2*); 488,700 cases of chlamydia, gonorrhea, and syphilis (*3*); and 1,652 diagnoses of human immunodeficiency virus (HIV) (*4*) were reported. Among persons aged ≥25 years, 54% of all deaths in the United States resulted from cardiovascular disease (31%) and cancer (23%) (*1*). These leading causes of mortality, morbidity, and social problems (e.g., academic failure, poverty, and crime) among youth and adults in the United States are associated with six categories of priority health-related behaviors: 1) behaviors that contribute to unintentional injuries and violence; 2) tobacco use; 3) alcohol and other drug use; 4) sexual behaviors that related to unintended pregnancy and sexually transmitted infections (STIs), including HIV infection; 5) unhealthy dietary behaviors; and 6) physical inactivity. These behaviors, as well as obesity, overweight, and asthma, frequently are related, are established during childhood and adolescence, and extend into adulthood.

Significant health disparities exist among demographic subgroups of youth defined by sex, race/ethnicity, and grade in school, and especially between sexual minority and nonsexual minority youth (*5*–*7*). More specifically, violence, human immunodeficiency virus (HIV) infection, STIs, and pregnancy occur more frequently among sexual minority youth than nonsexual minority youth. In addition, some sexual minority youth struggle with stigma, discrimination, family disapproval, and social rejection. However, although differences based on sex, race/ethnicity, and grade in school have been well documented, not enough is known about health-related behaviors that contribute to negative health outcomes among sexual minority youth (*5*,*7*).

Sexual identity and sex of sexual contacts can both be used to identify sexual minority youth. Sexual minority youth include those who identify as gay, lesbian, and bisexual and those who are not sure about their sexual identity as well as those who have sexual contact with only the same sex or with both sexes. Dissonance between sexual identity and sex of sexual contact occurs, particularly among youth (*6*–*12*). Some youth who identify as heterosexual, gay, lesbian, or bisexual and some youth who are not sure of their sexual identity might not have had any sexual contact. Some youth who have had sexual contact with only the same sex or with both sexes might identify as heterosexual and some youth who have had sexual contact with only the opposite sex might identify as gay, lesbian, or bisexual or might not be sure of their sexual identity. Sexual identity and sex of sexual contacts can change throughout the life span.

To monitor health-related behaviors and the prevalence of obesity, overweight, and asthma among youth, CDC developed the Youth Risk Behavior Surveillance System (YRBSS) (*13*). The YRBSS includes a school-based national Youth Risk Behavior Survey (YRBS) and state and large urban school district YRBSs conducted among representative samples of students in grades 9–12. National, state, and large urban school district surveys have been conducted biennially since 1991 (Supplementary Table 1). Since 1995, the need for more and higher quality data on the health-related behaviors of sexual minority high school students has been recognized by an increasing number of states and large urban school districts (Supplementary Table 2). With CDC support, these states and large urban school districts began adding at least one of two questions to their YRBS questionnaire to ascertain sexual identity, sex of sexual contacts, or both and to generate estimates of health-related behaviors by sexual identity and sex of sexual contacts. For the 1997 YRBSS cycle, a question on sexual identity and a question on sex of sexual contacts were placed on the YRBS Optional Question List indicating CDC’s support for the use of these questions. Results from seven states and six large urban school districts that used these questions during 2001–2009 were then summarized in a previous report in June 2011 (*14*). For the 2015 YRBSS cycle, on the basis of substantial support from the state and large urban school district YRBS coordinators, the two questions ascertaining sexual minority status were added to the standard YRBS questionnaire used by the states and large urban school districts as a starting point for their YRBS questionnaires. The two questions also were added to the national YRBS questionnaire. A report summarizing these national, state, and large urban school district results and providing the first national estimates of the numbers of sexual minority high school students was published in August 2016 (*15*).

This report summarizes results from the 2017 national YRBS, including 121 health-related behaviors and obesity, overweight, and asthma. Specifically, this report provides the latest update on the prevalence of health-related behaviors among United States high school students by demographic subgroups (i.e., sex, race/ethnicity, and grade) and by sexual minority status, updates the numbers of sexual minority students nationwide, and describes overall trends in health-related behaviors during 1991–2017. Results by sex and sexual minority status (where available) from the 39 state and 21 large urban school district surveys with weighted data for the 2017 YRBSS cycle (Figure) also are included in this report. Data from seven state surveys with unweighted data are not included. Among those sites with weighted data for 2017, three state and two large urban school district surveys were conducted during fall 2016; the national survey, 33 state, and 18 large urban school district surveys were conducted during spring 2017; and three state and one large urban school district surveys were conducted during fall 2017. Results from 30 state and all 21 large urban school district surveys that asked at least one of the questions to ascertain sexual minority status and had weighted data for the 2017 YRBSS cycle also are included in this report. Additional information about YRBSS is available at https://www.cdc.gov/yrbs.

**FIGURE:**
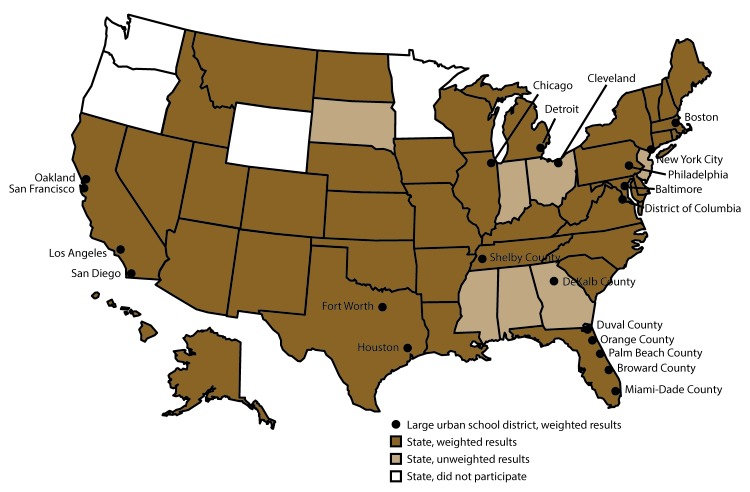
State and large urban school district Youth Risk Behavior Surveys — United States, 2017

## Methods

Detailed information about the methodology of the national, state, and large urban school district YRBSs has been described elsewhere (*13*). Information also is available at https://www.cdc.gov/yrbs.

## Sampling

### National Youth Risk Behavior Survey

The sampling frame for the 2017 national YRBS consisted of all regular public (including charter schools), Catholic, and other non-public schools with students in at least one of grades 9–12 in the 50 states and the District of Columbia. Alternative schools, special education schools, schools operated by the Department of Defense, Bureau of Indian Education schools, and vocational schools serving only pull-out populations were excluded. The sampling frame combined data sets obtained from Market Data Retrieval, Inc. (MDR) (*16*) and the National Center for Education Statistics (NCES) (*17*). The NCES data sets were based on the Common Core of Data for public schools and the Private School Survey for nonpublic schools. Very small schools with an enrollment of ≤40 across grades 9–12 were excluded.

A three-stage cluster sample design was used to produce a nationally representative sample of students in grades 9–12 who attend public and private schools. The first-stage sampling frame consisted of 1,257 primary sampling units (PSUs), consisting of counties; groups of smaller, adjacent counties; or parts of larger counties. The 1,257 PSUs were categorized into 16 strata according to their metropolitan statistical area (MSA) status (e.g., urban city) and the percentages of black and Hispanic students in the PSUs. From the 1,257 PSUs, 54 were sampled with probability proportional to overall school enrollment size for the PSU.

For the second stage of sampling, secondary sampling units (SSUs) were defined as a physical school with grades 9–12 or a school created by combining nearby schools to provide all four grades. From the 54 PSUs, 162 SSUs were sampled with probability proportional to school enrollment size. These 162 SSUs corresponded to 192 physical schools.

The third stage of sampling consisted of random sampling in each of grades 9–12, one or two classrooms from either a required subject (e.g., English or social studies) or a required period (e.g., homeroom or second period). All students in sampled classes were eligible to participate. Schools, classes, and students that refused to participate were not replaced.

In order to enable a separate analysis of data for black and Hispanic students, two classes per grade, rather than one, were sampled in schools with a high minority enrollment. Before the 2013 national YRBS, three strategies were used to oversample black and Hispanic students: 1) larger sampling rates were used to select PSUs that were in high-black and high-Hispanic strata; 2) a modified measure of size was used to increase the probability of sampling schools with a disproportionately high minority enrollment; and 3) two classes per grade, rather than one, were sampled in schools with a high minority enrollment. Because of increases in the proportions of black and Hispanic students in the population, only selection of two classes per grade was needed in the 2013, 2015, and 2017 national YRBS to balance the precision needed for subgroup estimates with minimum variance for overall estimates.

### State and Large Urban School District Youth Risk Behavior Surveys

States and large urban school districts used a two-stage cluster sample design to produce representative samples of students in grades 9–12 in their jurisdiction. In 2017, the samples were representative of regular public school and in some jurisdictions, charter school students, in grades 9–12 in 26 states and 13 large urban school districts and regular public school students plus students in grades 9–12 in other types of public schools (e.g., public alternative, special education, or vocational schools or schools overseen by the Bureau of Indian Education) in 13 states and eight large urban school districts.

In the first sampling stage, schools with any of grades 9–12 were sampled with probability proportional to school enrollment size in 36 states and four large urban school districts; all schools with any of grades 9–12 were invited to participate in three states and 17 large urban school districts. In the second sampling stage, intact classes from either a required subject (e.g., English or social studies) or a required period (e.g., homeroom or second period) were sampled randomly in 38 states and 20 large urban school districts, and all students in the sampled classes were eligible to participate. In one state and one large urban school district, all students in sampled schools were eligible to participate.

### Data Collection Procedures and Questionnaires

Survey procedures for the national, state, and large urban school district surveys were designed to protect students’ privacy by allowing for anonymous and voluntary participation. Before survey administration, local parental permission procedures were followed. Students completed the self-administered questionnaire during one class period and recorded their responses directly on a computer-scannable booklet or answer sheet. CDC’s Institutional Review Board approved the protocol for the national YRBS.

The 2017 YRBS standard questionnaire contained 89 questions. This questionnaire was used as the starting point for the state and large urban school district questionnaires. States and large urban school districts could add and delete questions from the standard questionnaire. Only two states and two large urban school districts included in this report used the 2017 YRBS standard questionnaire without modifications.

The 2017 national YRBS questionnaire contained 99 questions, including all 89 questions on the standard questionnaire. This report presents national results and state and large urban school district results for questions on the 2017 standard questionnaire and national (only) results from eight additional questions measuring having driven when they had been using marijuana, having ever used hallucinogenic drugs, sports drink consumption, plain water consumption, having done muscle-strengthening exercises on 3 or more days during the 7 days before the survey, indoor tanning device use, having had a sunburn, and having to avoid some foods because eating the food could cause an allergic reaction.

Two questions on the standard questionnaire and national questionnaire measured sexual minority status. Sexual identity was ascertained with the following question: “Which of the following best describes you?” Response options were “heterosexual (straight),” “gay or lesbian,” “bisexual,” and “not sure.” Sex of sexual contacts was ascertained with, “During your life, with whom have you had sexual contact?” Response options were “I have never had sexual contact,” “females,” “males,” and “females and males.” No definition was provided for sexual contact. Across all the states and large urban school districts included in this report, 30 states and 21 large urban school districts included the question on sexual identity and 26 states and 21 large urban school districts included the question on sex of sexual contacts.

Introductions on the standard questionnaire and national questionnaire before some questions provided additional information about the behaviors being measured. For example, bullying was defined as “when 1 or more students tease, threaten, spread rumors about, hit, shove, or hurt another student over and over again. It is not bullying when two students of about the same strength or power argue or fight or tease each other in a friendly way.” The questions on attempted suicide were preceded by, “Sometimes people feel so depressed about the future that they may consider attempting suicide, that is, taking some action to end their own life.” The introduction to the questions on electronic vapor products included brand names (blu, NJOY, Vuse, MarkTen, Logic, Vapin Plus, eGo, and Halo) and examples of types of electronic vapor products (e-cigarettes, e-cigars, e-pipes, vape pipes, vaping pens, e-hookahs, and hookah pens). The introduction to the questions on alcohol use clarified that drinking alcohol “includes drinking beer, wine, wine coolers, and liquor such as rum, gin, vodka, or whiskey. For these questions, drinking alcohol does not include drinking a few sips of wine for religious purposes.” The questions on dietary behaviors were preceded by, “Think about all the meals and snacks you had from the time you got up until you went to bed. Be sure to include food you ate at home, at school, at restaurants, or anywhere else.” Concussions were defined as, “when a blow or jolt to the head causes problems such as headaches, dizziness, being dazed or confused, difficulty remembering or concentrating, vomiting, blurred vision, or being knocked out.”

Except for six demographic questions (sex, grade in school, age, Hispanic ethnicity, race, and sexual identity) and three questions assessing height, weight, and asthma, all the remaining questions on the standard questionnaire and the national questionnaire in this report measured behaviors practiced or experienced by the student (referred to as “behaviors”). Skip patterns, which occur when a particular response to one question indicates to the respondents that they should not answer one or more subsequent questions, were not included in any YRBS questionnaire to protect students’ privacy by ensuring all students took about the same amount of time to complete the questionnaire. All questions (except for two questions assessing height and weight and the race question) were multiple choice with a maximum of eight mutually exclusive response options and only one possible answer per respondent. Information about the reliability of the standard questionnaire has been published elsewhere (*18*,*19*). The wording of each question, including recall periods and response options, and operational definitions for each variable can be found by reviewing the 2017 standard and national YRBS questionnaires and Data User’s Guide at https://www.cdc.gov/yrbs.

### Data Processing Procedures and Response Rates

For the 2017 national YRBS, 14,956 questionnaires were completed in 144 schools. The national data set was cleaned and edited for inconsistencies. Missing data were not statistically imputed. Among the 14,956 completed questionnaires, 191 failed quality control* and were excluded from analysis, resulting in 14,765 usable questionnaires (Supplementary Table 3). The school response rate was 75%, the student response rate was 81%, and the overall response rate was 60%^†^ (Supplementary Table 3).

Data from each state and large urban school district survey were cleaned and edited for inconsistencies with the same procedures used for the national data set. The percentage of completed questionnaires that failed quality control checks and were excluded from analysis ranged from 0.1% to 8.8% (median: 0.9%) across the states and from 0.3% to 10.7% (median: 1.7%) across the large urban school districts. The student sample sizes ranged from 1,273 to 51,087 (median: 2,139) across the states and from 805 to 10,191 (median: 1,971) across the large urban school districts (Supplementary Table 3). Among the states, the school response rates ranged from 68% to 100%, student response rates ranged from 66% to 90%, and overall response rates ranged from 60% to 82%. Among the large urban school districts, the school response rates ranged from 84% to 100%, student response rates ranged from 63% to 89%, and overall response rates ranged from 61% to 89% (Supplementary Table 3).

To obtain a sufficient sample size for analyses of health-related behaviors by sexual identity subgroups, students who selected “gay or lesbian” or “bisexual” were combined into a single subgroup and are referred to as “gay, lesbian, and bisexual students.” Students who selected “heterosexual (straight)” are referred to as “heterosexual students,” and students who selected “not sure” are referred to as “not sure students.” Sex of sexual contacts was ascertained from the questions, “During your life, with whom have you had sexual contact?” and “What is your sex?” Response options were “female” and “male.” To obtain a sufficient sample size for analyses of health-related behaviors by sex of sexual contact subgroups, students who had sexual contact with only the same sex or with both sexes were combined into a single subgroup and are referred to as “students who had sexual contact with only the same sex or with both sexes.” Students who had sexual contact with only the opposite sex are referred to as “students who had sexual contact with only the opposite sex.” Students who selected “I have never had sexual contact” are referred to as “students who had no sexual contact.” Students who had no sexual contact were excluded from analyses on sexual behaviors, female students who had sexual contact with only females were excluded from analyses on condom use and birth control use, and male students who had sexual contact with only males were excluded from analyses on birth control use.

Race/ethnicity was ascertained from two questions: 1) “Are you Hispanic or Latino?” (response options were “yes” or “no”), and 2) “What is your race?” Response options were “American Indian or Alaska Native,” “Asian,” “black or African American,” “Native Hawaiian or other Pacific Islander,” or “white.” For the second question, students could select more than one response option. For this report, students were classified as “Hispanic/Latino” and are referred to as “Hispanic” if they answered “yes” to the first question, regardless of how they answered the second question. Students who answered “no” to the first question and selected only “black or African American” to the second question were classified as “black or African American” and are referred to as “black.” Students who answered “no” to the first question and selected only “white” to the second question were classified and are referred to as “white.” Race/ethnicity was classified as missing for students who did not answer the first question and for students who answered “no” to the first question but did not answer the second question.

Students were classified as having obesity or being overweight based on their body mass index (kg/m^2^) (BMI), which was calculated from self-reported height and weight. BMI values were compared with sex- and age-specific reference data from the 2000 CDC growth charts (*20*). Obesity was defined as a BMI of ≥95th percentile for age and sex. Overweight was defined as a BMI of ≥85th percentile and <95th percentile for age and sex. These classifications are not intended to diagnose obesity or overweight in individual students but to provide population-level estimates of obesity and overweight.

### Weighting

For the national YRBS, a weight based on student sex, race/ethnicity, and grade was applied to each record to adjust for school and student nonresponse and oversampling of black and Hispanic students. The overall weights were scaled so that the weighted count of students equals the total sample size, and the weighted proportions of students in each grade match the national population proportions. Therefore, weighted estimates are representative of all students in grades 9–12 attending public and private schools in the United States.

Data from states and large urban school districts that had a representative sample of students, appropriate documentation, and an overall response rate of ≥60% were weighted. A weight based on student sex, race/ethnicity, and grade was applied to each record to adjust for school and student nonresponse in each jurisdiction. The weighted count of students equals the student population in each jurisdiction. Data from 39 states and 21 large urban school districts were weighted. In 26 states and 13 large urban school districts weighted estimates are representative of all students in grades 9–12 attending regular public schools and in 13 states and eight large urban school districts weighted estimates are representative of regular public school students plus students in grades 9–12 in other types of public schools (e.g., public alternative, special education, or vocational schools or schools overseen by the Bureau of Indian Education).

### Analytic Methods

Statistical analyses were conducted on weighted data using SAS (*21*) and SUDAAN (*22*) software to account for the complex sampling designs. Prevalence estimates and confidence intervals were computed for all variables and all data sets. In the supplementary tables, prevalence estimates, confidence intervals, or both are not provided in the following instances: 1) the question was not asked; 2) the number of students in the relevant subgroup is <100 for sites with an overall sample size ≥1,000 students, <50 for sites with an overall sample size of 200–999 students, and <30 for any analyses including either of the variables ascertaining sexual minority status; or 3) the prevalence estimate was 0%. In addition, ranges and medians for the overall prevalence estimates were computed across states and across large urban school districts for all variables unless fewer than five sites had data available. 

In addition, for the national YRBS data, t tests were used to determine pairwise differences between subpopulations (*23*). Differences between prevalence estimates were considered statistically significant if the t test p value was <0.05 for main effects (sex, race/ethnicity, grade, sexual identity, and sex of sexual contacts) and for interactions (sex by race/ethnicity, sex by grade, race/ethnicity by sex, grade by sex, sex by sexual identity, sexual identity by sex, sex by sex of sexual contacts, and sex of sexual contacts by sex). In the results section, only statistically significant differences in national YRBS prevalence estimates are reported in the following order: sex, sex by race/ethnicity, sex by grade, race/ethnicity, race/ethnicity by sex, grade, grade by sex, sexual identity, sex by sexual identity, sexual identity by sex, sex of sexual contacts, sex by sex of sexual contacts, and sex of sexual contacts by sex.

To identify overall long-term temporal trends in health-related behaviors nationwide, prevalence estimates from the earliest year of data collection to 2017 for each variable assessed with identically worded questions were examined. Logistic regression analyses were used to account for all available estimates; control for sex, grade, and racial/ethnic changes over time; and assess long-term linear and quadratic trends (*23*). A p value associated with the regression coefficient that was <0.05 was considered statistically significant. Linear and quadratic time variables were treated as continuous and were coded using orthogonal coefficients calculated with PROC IML in SAS. A minimum of 3 survey years was required to calculate linear trends, and a minimum of 6 survey years was required to calculate quadratic trends. Separate regression models were used to assess linear and quadratic trends for every variable. When a significant quadratic trend was identified, Joinpoint software (*24*) was used to automate identification of the year, or joinpoint, where the nonlinear (i.e., quadratic) trend changed, then regression models were used to identify linear trends occurring in each segment. Cubic and higher order trends were not assessed. A quadratic trend indicates a significant but nonlinear trend in prevalence over time. A long-term temporal change that includes a significant linear and quadratic trend demonstrates nonlinear variation (e.g., leveling off or change in direction) in addition to an overall increase or decrease over time.

To identify 2-year changes in health-related behaviors nationwide, prevalence estimates from 2015 and 2017 were compared using t tests for each variable assessed with identically worded questions in both survey years. Prevalence estimates were considered statistically different if the t test p value was <0.05.

In the results section, long-term linear and quadratic trends are described first, followed by results from the t tests used to assess 2-year changes. Prevalence estimates not provided in the results section can be found at Youth Online (https://nccd.cdc.gov/youthonline/App/Default.aspx). Information about long-term temporal trends and 2-year changes are not available because of changes in question or response option wording or because the question was asked for the first time during 2017 for the following variables: having driven when they had been using marijuana; having carried a gun; having experienced sexual violence by anyone; having first tried cigarette smoking before age 13 years; having usually gotten their own electronic vapor products by buying them in a store; current, current frequent, and current daily smokeless tobacco use; current cigarette, cigar, or smokeless tobacco use; current cigarette, cigar, smokeless tobacco, or electronic vapor product use; having tried to quit using all tobacco products; current binge drinking; having ever taken prescription pain medicine without a doctor’s prescription or differently than how a doctor told them to use it; and having had a concussion one or more times from playing a sport or being physically active.

## Results

### Demographic Characteristics

#### Sex, Grade, and Race/Ethnicity

Data from the national YRBS were weighted to match national population proportions. Thus, 50.7% of the students were female, 27.3% were in 9th grade, 25.6% were in 10th grade, 23.9% were in 11th grade, and 23.0% were in 12th grade (Supplementary Table 3). A total of 53.5% were white, 13.4% were black, 22.8% were Hispanic, and 10.3% were American Indian or Alaska Native, Asian, Native Hawaiian or other Pacific Islander, or multiple race (non-Hispanic). The demographic characteristics of the state and local samples varied by jurisdiction but were weighted to match the demographic characteristics of each student population.

#### Sexual Identity

Nationwide, 85.4% of students identified as heterosexual, 2.4% identified as gay or lesbian, 8.0% identified as bisexual, and 4.2% were not sure of their sexual identity (Supplementary Table 4). Across 30 states, 79.9%–88.0% (median: 85.1%) of students identified as heterosexual, 1.7%–6.4% (median: 2.9%) identified as gay or lesbian, 6.4%–10.3% (median 7.8%) identified as bisexual, and 2.6%–8.4% (median: 4.2%) were not sure of their sexual identity. Across 21 large urban school districts, 74.7%–88.4% (median: 82.8%) of students identified as heterosexual, 1.7%–5.5% (median: 3.5%) identified as gay or lesbian, 5.5%–11.9% (median: 7.9%) identified as bisexual, and 3.3%–14.9% (median: 4.7%) were not sure of their sexual identity.

#### Sex of Sexual Contacts

Nationwide, 45.3% of students had had sexual contact with only the opposite sex, 1.6% had had sexual contact with only the same sex, 5.3% had had sexual contact with both sexes, and 47.8% had had no sexual contact (Supplementary Table 5). Across 26 states, 33.6%–51.4% (median: 45.3%) of students had had sexual contact with only the opposite sex, from 1.5% to 6.9% (median: 2.8%) had had sexual contact with only the same sex, 3.1%–6.2% (median: 5.0%) had had sexual contact with both sexes, and 40.0%–58.9% (median: 47.0%) had had no sexual contact. Across 21 large urban school districts, 28.6%–50.5% (median: 44.6%) of students had had sexual contact with only the opposite sex, 2.7%–6.6% (median: 4.0%) had had sexual contact with only the same sex, 3.3%–9.8% (median: 5.5%) had had sexual contact with both sexes, and 36.3%–64.3% (median: 45.8%) had had no sexual contact.

#### Dissonance Between Sexual Identity and Sex of Sexual Contact

Nationwide, among students who had sexual contact with only the opposite sex, 94.1% identified as heterosexual; 4.0% identified as gay, lesbian, or bisexual; and 1.9% were not sure of their sexual identity (Supplementary Table 6). Across 26 states, among students who had sexual contact with only the opposite sex, 88.1%–96.2% (median: 93.3%) identified as heterosexual; 2.9%–7.9% (median: 4.6%) identified as gay, lesbian, or bisexual; and 0.9%–5.2% (median: 2.2%) were not sure of their sexual identity. Across 21 large urban school districts, among students who had sexual contact with only the opposite sex, 83.5%–94.3%% (median: 92.4%) identified as heterosexual; 3.1%–6.7% (median: 5.2%) identified as gay, lesbian, or bisexual; and 1.3%–10.0% (median: 2.5%) were not sure of their sexual identity.

Nationwide, among students who had sexual contact with only the same sex or with both sexes, 20.1% identified as heterosexual; 68.4% identified as gay, lesbian, or bisexual; and 11.4% were not sure of their sexual identity (Supplementary Table 6). Across 26 states, among students who had sexual contact with only the same sex or with both sexes, 18.7%–43.0% (median: 29.4%) identified as heterosexual; 48.8%–71.1% (median: 60.2%) identified as gay, lesbian, or bisexual; and 4.1%–18.7% (median: 9.0%) were not sure of their sexual identity. Across 21 large urban school districts, among students who had sexual contact with only the same sex or with both sexes, 20.8%–47.4% (median: 30.6%) identified as heterosexual; 43.2%–66.9% (median: 59.1%) identified as gay, lesbian, or bisexual; and 1.3%–20.2% (median: 10.3%) were not sure of their sexual identity.

Nationwide, among students who had no sexual contact, 87.6% identified as heterosexual; 7.7% identified as gay, lesbian, or bisexual; and 4.7% were not sure of their sexual identity (Supplementary Table 6). Across 26 states, among students who had no sexual contact, 84.2%–91.7% (median: 88.0%) identified as heterosexual; 5.0%–10.1% (median: 7.3%) identified as gay, lesbian, or bisexual; and 3.0%–8.0% (median: 4.6%) were not sure of their sexual identity. Across 21 large urban school districts, among students who had no sexual contact, 78.3%–90.2% (median: 87.8%) identified as heterosexual; 5.2%–13.6% (median: 7.4%) identified as gay, lesbian, or bisexual; and 3.0%–14.6% (median: 5.0%) were not sure of their sexual identity.

### Behaviors that Contribute to Unintentional Injuries

#### Rarely or Never Wear a Seat Belt

Nationwide, 5.9% of students rarely or never wore a seat belt when riding in a car driven by someone else (Supplementary Table 7). The prevalence of rarely or never wearing a seat belt was higher among male (6.6%) than female (5.1%) students, higher among white male (5.3%) than white female (3.4%) students, and higher among 11th-grade male (6.9%) and 12th-grade male (7.9%) than 11th-grade female (4.6%) and 12th-grade female (4.0%) students, respectively. The prevalence of rarely or never wearing a seat belt was higher among black (9.8%) and Hispanic (7.3%) than white (4.3%) students, higher among black (9.8%) than Hispanic (7.3%) students, higher among black female (8.1%) and Hispanic female (7.6%) than white female (3.4%) students, and higher among black male (11.3%) than white male (5.3%) and Hispanic male (7.0%) students. The prevalence of rarely or never wearing a seat belt was higher among 9th-grade female (6.5%) than 12th-grade female (4.0%) students.

Analyses based on the question ascertaining sexual identity indicated that nationwide, 5.8% of heterosexual students; 6.1% of gay, lesbian, and bisexual students; and 7.9% of not sure students rarely or never wore a seat belt when riding in a car driven by someone else (Supplementary Table 7). Among male students, the prevalence of rarely or never wearing a seat belt was higher among not sure (11.6%) than heterosexual (6.4%) students. The prevalence also was higher among not sure male (11.6%) than not sure female (4.1%) students.

Analyses based on the question ascertaining the sex of sexual contacts indicated that nationwide, 8.3% of students who had sexual contact with only the opposite sex, 8.1% of students who had sexual contact with only the same sex or with both sexes, and 2.9% of students who had no sexual contact rarely or never wore a seat belt (Supplementary Table 7). The prevalence of rarely or never wearing a seat belt was higher among students who had sexual contact with only the opposite sex (8.3%) and students who had sexual contact with only the same sex or with both sexes (8.1%) than students who had no sexual contact (2.9%). Among female students, the prevalence was higher among those who had sexual contact with only males (6.5%) and those who had sexual contact with only females or with both sexes (7.8%) than those who had no sexual contact (3.3%). Among male students, the prevalence was higher among those who had sexual contact with only females (9.7%) and those who had sexual contact with only males or with both sexes (8.7%) than those who had no sexual contact (2.5%). The prevalence also was higher among male students who had sexual contact with only females (9.7%) than female students who had sexual contact with only males (6.5%).

Trend analyses indicated that during 1991–2017, a significant linear decrease (25.9%–5.9%) occurred in the overall prevalence of rarely or never wearing a seat belt. A significant quadratic trend was not identified. The prevalence of rarely or never wearing a seat belt did not change significantly from 2015 (6.1%) to 2017 (5.9%).

Analyses of state and large urban school district data indicated that across 33 states, the overall prevalence of rarely or never wearing a seat belt ranged from 5.0% to 17.5% across state surveys (median: 7.3%) (Supplementary Table 8). Across 16 large urban school districts, the prevalence ranged from 5.3% to 22.1% (median: 9.6%).

#### Rode with a Driver Who Had Been Drinking Alcohol

Nationwide, 16.5% of students had ridden one or more times during the 30 days before the survey in a car or other vehicle driven by someone who had been drinking alcohol (Supplementary Table 9). The prevalence of having ridden with a driver who had been drinking alcohol was higher among black female (19.1%) than black male (14.8%) students. The prevalence of having ridden with a driver who had been drinking alcohol was higher among Hispanic (20.7%) than white (15.0%) and black (17.0%) students, higher among Hispanic female (21.9%) than white female (15.7%) students, and higher among Hispanic male (19.5%) than white male (14.2%) and black male (14.8%) students.

Analyses based on the question ascertaining sexual identity indicated that nationwide, 16.1% of heterosexual students; 20.1% of gay, lesbian, and bisexual students; and 20.9% of not sure students had ridden with a driver who had been drinking alcohol (Supplementary Table 9). The prevalence of having ridden with a driver who had been drinking alcohol was higher among gay, lesbian, and bisexual (20.1%) than heterosexual (16.1%) students. The prevalence also was higher among heterosexual female (17.1%) than heterosexual male (15.3%) students.

Analyses based on the question ascertaining the sex of sexual contacts indicated that nationwide, 21.1% of students who had sexual contact with only the opposite sex, 26.7% of students who had sexual contact with only the same sex or with both sexes, and 10.6% of students who had no sexual contact (Supplementary Table 9) had ridden with a driver who had been drinking alcohol. The prevalence of having ridden with a driver who had been drinking alcohol was higher among students who had sexual contact with only the opposite sex (21.1%) and students who had sexual contact with only the same sex or with both sexes (26.7%) than students who had no sexual contact (10.6%) and higher among students who had sexual contacts with only the same sex or with both sexes (26.7%) than students who had sexual contact with only the opposite sex (21.1%). Among female students, the prevalence was higher among those who had sexual contact with only males (21.1%) and those who had sexual contact with only females or with both sexes (28.0%) than those who had no sexual contact (12.0%) and higher among those who had sexual contact with only females or with both sexes (28.0%) than those who had sexual contact with only males (21.1%). Among male students, the prevalence was higher among those who had sexual contact with only females (21.2%) and those who had sexual contact with only males or with both sexes (23.1%) than those who had no sexual contact (9.2%). The prevalence also was higher among female students who had no sexual contact (12.0%) than male students who had no sexual contact (9.2%).

Trend analyses indicated that during 1991–2017, a significant linear decrease (39.9%–16.5%) occurred in the overall prevalence of having ridden with a driver who had been drinking alcohol. A significant quadratic trend also was identified. The prevalence of having ridden with a driver who had been drinking alcohol decreased during 1991–2009 (39.9%–28.3%) and then decreased more rapidly during 2009–2017 (28.3%–16.5%). The prevalence of having ridden with a driver who had been drinking alcohol decreased significantly from 2015 (20.0%) to 2017 (16.5%).

Analyses of state and large urban school district data indicated that across 34 states, the overall prevalence of having ridden with a driver who had been drinking alcohol ranged from 12.8% to 28.2% across state surveys (median: 16.5%) (Supplementary Table 10). Across 19 large urban school districts, the prevalence ranged from 14.0% to 27.0% (median: 19.5%).

#### Drove When They Had Been Drinking Alcohol

Among the 62.6% of students nationwide who drove a car or other vehicle during the 30 days before the survey,^§^ 5.5% had driven a car or other vehicle one or more times when they had been drinking alcohol during the 30 days before the survey (Supplementary Table 11). The prevalence of having driven a car or other vehicle when they had been drinking alcohol was higher among male (6.8%) than female (4.1%) students; higher among white male (6.3%) and Hispanic male (8.5%) than white female (3.8%) and Hispanic female (5.4%) students, respectively; and higher among 11th-grade male (6.9%) and 12th-grade male (10.4%) than 11th-grade female (4.1%) and 12th-grade female (5.9%) students, respectively. The prevalence of having driven a car or other vehicle when they had been drinking alcohol was higher among Hispanic (7.0%) than white (5.0%) and black (4.1%) students, higher among Hispanic female (5.4%) than white female (3.8%) students, and higher among white male (6.3%) and Hispanic male (8.5%) than black male (4.1%) students. The prevalence of having driven a car or other vehicle when they had been drinking alcohol was higher among 11th-grade (5.5%) and 12th-grade (8.1%) than 9th-grade (3.2%) and 10th-grade (3.2%) students, respectively; higher among 12th-grade (8.1%) than 11th-grade (5.5%) students; higher among 12th-grade female (5.9%) than 9th-grade female (2.4%), 10th-grade female (2.4%), and 11th-grade female (4.1%) students; higher among 11th-grade male (6.9%) than 10th grade male (4.0%) students; and higher among 12th-grade male (10.4%) than 9th-grade male (4.0%), 10th-grade male (4.0%), and 11th-grade male (6.9%) students.

Analyses based on the question ascertaining sexual identity indicated that nationwide, among the students who drove a car or other vehicle during the 30 days before the survey, 5.2% of heterosexual students; 6.9% of gay, lesbian, and bisexual students; and 9.5% of not sure students had driven a car or other vehicle when they had been drinking alcohol (Supplementary Table 11). Among female students, the prevalence of having driven a car or other vehicle when they had been drinking alcohol was higher among gay and bisexual (7.1%) than heterosexual (3.5%) students. The prevalence also was higher among heterosexual male (6.8%) than heterosexual female (3.5%) students.

Analyses based on the question ascertaining the sex of sexual contacts indicated that nationwide, among the students who drove a car or other vehicle during the 30 days before the survey, 8.4% of students who had sexual contact with only the opposite sex, 10.3% of students who had sexual contact with only the same sex or with both sexes, and 1.0% of students who had no sexual contact had driven a car or other vehicle when they had been drinking alcohol (Supplementary Table 11). The prevalence of having driven a car or other vehicle when they had been drinking alcohol was higher among students who had sexual contact with only the opposite sex (8.4%) and students who had sexual contact with only the same sex or with both sexes (10.3%) than students who had no sexual contact. Among female students, the prevalence was higher among those who had sexual contact with only males (5.4%) and those who had sexual contact with only females or with both sexes (10.3%) than those who had no sexual contact (1.2%) and among those who had sexual contact with only females or with both sexes (10.3%) than those who had sexual contact only with males (5.4%). Among male students, the prevalence was higher among those who had sexual contact with only females (10.8%) and those who had sexual contact with only males or with both sexes (10.5%) than those who had no sexual contact (0.9%). The prevalence also was higher among male students who had sexual contact with only females (10.8%) than female students who had sexual contact with only males (5.4%).

Trend analyses indicated that during 2013–2017, a significant linear decrease (10.0%–5.5%) occurred in the overall prevalence of having driven a car or other vehicle when they had been drinking alcohol, among the students who drove a car or other vehicle during the 30 days before the survey. Not enough data points were available to identify a quadratic trend. The prevalence of having driven a car or other vehicle when they had been drinking alcohol decreased significantly from 2015 (7.8%) to 2017 (5.5%).

Analyses of state and large urban school district data indicated that across 34 states, the overall prevalence of having driven a car or other vehicle when they had been drinking alcohol, among the students who drove a car or other vehicle during the 30 days before the survey, ranged from 2.8% to 10.7% across state surveys (median: 5.7%) (Supplementary Table 12). Across 18 large urban school districts, the prevalence ranged from 2.2% to 8.0% (median: 5.5%).

#### Drove When They Had Been Using Marijuana

Among the 64.5% of students nationwide who drove a car or other vehicle during the 30 days before the survey,^§^ 13.0% had driven a car or other vehicle one or more times when they had been using marijuana (also called grass, pot, or weed) during the 30 days before the survey (Supplementary Table 13). The prevalence of having driven a car or other vehicle when they had been using marijuana was higher among male (14.6%) than female (11.3%) students; higher among white male (13.7%) than white female (10.2%) students; and higher among 9th-grade male (10.2%) and 10th-grade male (13.5%) than 9th-grade female (4.5%) and 10th-grade female (8.9%) students, respectively. The prevalence of having driven a car or other vehicle when they had been using marijuana was higher among 10th-grade (11.3%), 11th-grade (12.3%), and 12th-grade (18.3%) than 9th-grade (7.3%) students; higher among 12th-grade (18.3%) than 10th-grade (11.3%) and 11th-grade (12.3%) students, higher among 10th-grade female (8.9%), 11th-grade female (11.7%), and 12th-grade female (16.5%) than 9th-grade female (4.5%) students; higher among 12th-grade female (16.5%) than 10th-grade female (8.9%) and 11th-grade female (11.7%) students; and higher among 12th-grade male (20.1%) than 9th-grade male (10.2%), 10th-grade male (13.5%), and 11th-grade male (12.8%) students.

Analyses based on the question ascertaining sexual identity indicated that nationwide, among the students who drove a car or other vehicle during the 30 days before the survey, 12.2% of heterosexual students; 20.5% of gay, lesbian, and bisexual students; and 21.7% of not sure students had driven a car or other vehicle one or more times when they had been using marijuana (Supplementary Table 13). The prevalence of having driven a car or other vehicle when they had been using marijuana was higher among gay, lesbian, and bisexual (20.5%) than heterosexual (12.2%) students. Among female students, the prevalence was higher among lesbian and bisexual (20.2%) than heterosexual (10.0%) students. The prevalence also was higher among heterosexual male (14.1%) than heterosexual female (10.0%) students.

Analyses based on the question ascertaining the sex of sexual contacts indicated that nationwide, among the students who drove a car or other vehicle during the 30 days before the survey, 19.1% of students who had sexual contact with only the opposite sex, 30.0% of students who had sexual contact with only the same sex or with both sexes, and 2.6% of students who had no sexual contact had driven a car or other vehicle one or more times when they had been using marijuana (Supplementary Table 13). The prevalence of having driven a car or other vehicle when they had been using marijuana was higher among students who had sexual contact with only the opposite sex (19.1%) and students who had sexual contact with only the same sex or with both sexes (30.0%) than students who had no sexual contact (2.6%) and higher among students who had sexual contact with only the same sex or with both sexes (30.0%) than students who had sexual contact with only the opposite sex (19.1%). Among female students, the prevalence was higher among those who had sexual contact with only males (16.0%) and those who had sexual contact with only females or with both sexes (30.5%) than those who had no sexual contact (2.0%) and higher among those who had sexual contact with only females or with both sexes (30.5%) than those who had sexual contact with only males (16.0%). Among male students, the prevalence was higher among those who had sexual contact with only females (21.4%) and those who had sexual contact with only males or with both sexes (28.7%) than those who had no sexual contact (3.2%). The prevalence also was higher among male students who had sexual contact with only females (21.4%) than female students who had sexual contact with only males (16.0%).

The question measuring the prevalence of having driven a car or other vehicle when using marijuana was used for the first time in the 2017 national YRBS. As a result, long-term temporal trends and 2-year temporal changes are not available for this variable.

The question also was not included in the standard questionnaire used in the state and large urban school district surveys in 2017. As a result, the range and median prevalence estimates across states and large urban school districts for the prevalence of having driven a car or other vehicle when using marijuana are not available.

#### Texted or E-Mailed While Driving

Among the 62.8% of students nationwide who drove a car or other vehicle during the 30 days before the survey,^§^ 39.2% had texted or e-mailed while driving a car or other vehicle on at least 1 day during the 30 days before the survey (Supplementary Table 14). The prevalence of having texted or e-mailed while driving was higher among white (43.9%) and Hispanic (36.6%) than black (26.9%) students, higher among white (43.9%) than Hispanic (36.6%) students, higher among white female (46.0%) and Hispanic female (36.8%) than black female (27.4%) students, higher among white female (46.0%) than Hispanic female (36.8%) students, higher among white male (41.7%) and Hispanic male (36.5%) than black male (26.3%) students, and higher among white male (41.7%) than Hispanic male (36.5%) students. The prevalence of having texted or e-mailed while driving was higher among 10th-grade (24.5%), 11th-grade (45.5%), and 12th-grade (59.3%) than 9th-grade (12.9%) students; higher among 11th-grade (45.5%) and 12th-grade (59.3%) than 10th-grade (24.5%) students; higher among 12th-grade (59.3%) than 11th-grade (45.5%) students; higher among 10th-grade female (25.1%), 11th-grade female (47.9%), and 12th-grade female (60.3%) than 9th-grade female (11.3%) students; higher among 11th-grade female (47.9%) and 12th-grade female (60.3%) than 10th-grade female (25.1%) students; higher among 12th-grade female (60.3%) than 11th-grade female (47.9%) students; higher among 10th-grade male (24.0%), 11th-grade male (43.2%), and 12th-grade male (58.5%) than 9th-grade male (14.4%) students; higher among 11th-grade male (43.2%) and 12th-grade male (58.5%) than 10th-grade male (24.0%) students; and higher among 12th-grade male (58.5%) than 11th-grade male (43.2%) students.

Analyses based on the question ascertaining sexual identity indicated that nationwide, among the students who drove a car or other vehicle during the 30 days before the survey, 39.5% of heterosexual students; 38.1% of gay, lesbian, and bisexual students; and 35.9% of not sure students texted or e-mailed while driving a car or other vehicle (Supplementary Table 14). The prevalence of having texted or e-mailed while driving was higher among heterosexual female (41.5%) than heterosexual male (38.0%) students.

Analyses based on the question ascertaining the sex of sexual contacts indicated that nationwide, among the students who drove a car or other vehicle during the 30 days before the survey, 52.9% of students who had sexual contact with only the opposite sex, 44.0% of students who had sexual contact with only the same sex or with both sexes, and 23.0% of students who had no sexual contact had texted or e-mailed while driving (Supplementary Table 14). The prevalence of having texted or e-mailed while driving was higher among students who had sexual contact with only the opposite sex (52.9%) and students who had sexual contact with only the same sex or with both sexes (44.0%) than students who had no sexual contact (23.0%) and higher among students who had sexual contact with only the opposite sex (52.9%) than students who had sexual contact with only the same sex or with both sexes (44.0%). Among female students, the prevalence was higher among those who had sexual contact with only males (55.6%) and those who had sexual contact with only females or with both sexes (45.3%) than those who had no sexual contact (25.1%) and higher among those who had sexual contact with only males (55.6%) than those who had sexual contact with only females or with both sexes (45.3%). Among male students, the prevalence was higher among those who had sexual contact with only females (50.7%) and those who had sexual contact with only males or with both sexes (40.2%) than those who had no sexual contact (20.9%). The prevalence also was higher among female students who had sexual contact with only males (55.6%) than male students who had sexual contact with only females (50.7%) and higher among female students who had no sexual contact (25.1%) than male students who had no sexual contact (20.9%).

Trend analyses did not identify a significant linear trend in the overall prevalence of having texted or e-mailed while driving among the students who drove a car or other vehicle during the 30 days before the survey during 2013–2017 (41.4%–39.2%). Not enough data points were available to identify a quadratic trend. The prevalence of texting or e-mailing while driving did not change significantly from 2015 (41.5%) to 2017 (39.2%).

Analyses of state and large urban school district data indicated that across 36 states, the overall prevalence of having texted or e-mailed while driving, among the students who drove a car or other vehicle during the 30 days before the survey, ranged from 27.4% to 55.2% across state surveys (median: 39.3%) (Supplementary Table 15). Across 19 large urban school districts, the prevalence ranged from 18.0% to 36.6% (median: 31.4%).

### Behaviors That Contribute to Violence

#### Carried a Weapon

Nationwide, 15.7% of students had carried a weapon (e.g., gun, knife, or club) on at least 1 day during the 30 days before the survey (Supplementary Table 16). The prevalence of having carried a weapon was higher among male (24.2%) than female (7.4%) students; higher among white male (29.0%), black male (15.3%), and Hispanic male (18.4%) than white female (8.0%), black female (6.1%), and Hispanic female (6.9%) students, respectively; and higher among 9th-grade male (23.2%), 10th-grade male (24.5%), 11th-grade male (25.3%), and 12th-grade male (23.2%) than 9th-grade female (7.6%), 10th-grade female (6.3%), 11th-grade female (8.6%), and 12th-grade female (6.6%) students, respectively. The prevalence of having carried a weapon was higher among white (18.1%) than black (10.8%) and Hispanic (12.7%) students and higher among white male (29.0%) than black male (15.3%) and Hispanic male (18.4%) students. The prevalence of having carried a weapon was higher among 11th-grade (16.8%) than 12th-grade (14.6%) students and higher among 11th-grade female (8.6%) than 10th-grade female (6.3%) students.

Analyses based on the question ascertaining sexual identity indicated that nationwide, 15.6% of heterosexual students; 16.2% of gay, lesbian, and bisexual students; and 17.4% of not sure students had carried a weapon (Supplementary Table 16). Among female students, the prevalence of having carried a weapon was higher among lesbian and bisexual (14.1%) than heterosexual (6.1%) students. The prevalence also was higher among heterosexual male (23.7%) than heterosexual female (6.1%) students, higher among gay and bisexual male (22.9%) than lesbian and bisexual female (14.1%) students, and higher among not sure male (27.6%) than not sure female (9.3%) students.

Analyses based on the question ascertaining the sex of sexual contacts indicated that nationwide, 20.1% of students who had sexual contact with only the opposite sex, 21.4% of students who had sexual contact with only the same sex or with both sexes, and 10.5% of students who had no sexual contact had carried a weapon (Supplementary Table 16). The prevalence of having carried a weapon was higher among students who had sexual contact with only the opposite sex (20.1%) and students who had sexual contact with only the same sex or with both sexes (21.4%) than students who had no sexual contact (10.5%). Among female students, the prevalence was higher among those who had sexual contact with only males (7.9%) and those who had sexual contact with only females or with both sexes (17.2%) than those who had no sexual contact (5.1%) and higher among those who had sexual contact with only females or with both sexes (17.2%) than those who had sexual contact with only males (7.9%). Among male students, the prevalence was higher among those who had sexual contact with only females (30.2%) and those who had sexual contact with only males or with both sexes (33.4%) than those who had no sexual contact (16.2%). The prevalence also was higher among male students who had sexual contact with only females (30.2%) than female students who had sexual contact with only males (7.9%), higher among male students who had sexual contact with only males or with both sexes (33.4%) than female students who had sexual contact with only females or with both sexes (17.2%), and higher among male students who had no sexual contact (16.2%) than female students who had no sexual contact (5.1%).

Trend analyses indicated that during 1991–2017, a significant linear decrease (26.1%–15.7%) occurred in the overall prevalence of having carried a weapon. A significant quadratic trend also was identified. The prevalence of having carried a weapon decreased during 1991–1997 (26.1%–18.3%) and then did not change significantly during 1997–2017 (18.3%–15.7%). The prevalence of having carried a weapon did not change significantly from 2015 (16.2%) to 2017 (15.7%).

Analyses of state and large urban school district data indicated that across 26 states, the overall prevalence of having carried a weapon ranged from 11.1% to 29.6% across state surveys (median: 18.2%) (Supplementary Table 17). Across 20 large urban school districts, the prevalence ranged from 7.8% to 19.0% (median: 11.7%).

#### Carried a Weapon on School Property

Nationwide, 3.8% of students had carried a weapon (e.g., a gun, knife, or club) on school property on at least 1 day during the 30 days before the survey (Supplementary Table 18). The prevalence of having carried a weapon on school property was higher among male (5.6%) than female (1.9%) students; higher among white male (5.9%), black male (5.4%), and Hispanic male (4.5%) than white female (1.7%), black female (1.7%), and Hispanic female (2.5%) students, respectively; and higher among 9th-grade male (3.6%), 10th-grade male (4.8%), 11th-grade male (7.1%), and 12th-grade male (7.0%) than 9th-grade female (1.3%), 10th-grade female (1.4%), 11th-grade female (3.0%), and 12th-grade female (1.5%) students, respectively. The prevalence of having carried a weapon on school property was higher among 11th-grade (5.0%) and 12th-grade (4.2%) than 9th-grade (2.5%) students; higher among 11th-grade (5.0%) than 10th-grade (3.2%) students; higher among 11th-grade female (3.0%) than 9th-grade female (1.3%), 10th-grade female (1.4%), and 12th-grade female (1.5%) students; and higher among 11th-grade male (7.1%) and 12th-grade male (7.0%) than 9th-grade male (3.6%) students.

Analyses based on the question ascertaining sexual identity indicated that nationwide, 3.4% of heterosexual students; 5.9% of gay, lesbian, and bisexual students; and 4.9% of not sure students had carried a weapon on school property (Supplementary Table 18). The prevalence of having carried a weapon on school property was higher among gay, lesbian, and bisexual (5.9%) than heterosexual (3.4%) students. Among female students, the prevalence was higher among lesbian and bisexual (4.9%) than heterosexual (1.4%) students. The prevalence also was higher among heterosexual male (5.0%) than heterosexual female (1.4%) students and higher among not sure male (6.8%) than not sure female (2.2%) students.

Analyses based on the question ascertaining the sex of sexual contacts indicated that nationwide, 4.5% of students who had sexual contact with only the opposite sex, 6.8% of students who had sexual contact with only the same sex or with both sexes, and 1.6% of students who had no sexual contact had carried a weapon on school property (Supplementary Table 18). The prevalence of having carried a weapon on school property was higher among students who had sexual contact with only the opposite sex (4.5%) and students who had sexual contact with only the same sex or with both sexes (6.8%) than students who had no sexual contact (1.6%). Among female students, the prevalence was higher among those who had sexual contact with only females or with both sexes (6.0%) than those who had sexual contact with only males (1.5%) and those who had no sexual contact (1.1%). Among male students, the prevalence was higher among those who had sexual contact with only females (7.0%) and those who had sexual contact with only males or with both sexes (9.1%) than those who had no sexual contact (2.2%). The prevalence also was higher among male students who had sexual contact with only females (7.0%) than female students who had sexual contact with only males (1.5%) and higher among male students who had no sexual contact (2.2%) than female students who had no sexual contact (1.1%).

Trend analyses indicated that during 1993–2017, a significant linear decrease (11.8%–3.8%) occurred in the overall prevalence of having carried a weapon on school property. A significant quadratic trend also was identified. The prevalence of having carried a weapon on school property decreased during 1993–1997 (11.8%–8.5%) and then decreased more slowly during 1997–2017 (8.5%–3.8%). The prevalence of having carried a weapon on school property did not change significantly from 2015 (4.1%) to 2017 (3.8%).

Analyses of state and large urban school district data indicated that across 35 states, the overall prevalence of having carried a weapon on school property ranged from 2.2% to 10.2% across state surveys (median: 4.9%) (Supplementary Table 19). Across 18 large urban school districts, the prevalence ranged from 1.6% to 7.8% (median: 3.3%).

#### Carried a Gun

Nationwide, 4.8% of students had carried a gun on at least 1 day (not counting the days when they carried a gun only for hunting or for a sport, such as target shooting) during the 12 months before the survey (Supplementary Table 20). The prevalence of having carried a gun was higher among male (7.7%) than female (1.9%) students; higher among white male (7.0%), black male (9.8%), and Hispanic male (9.0%) than white female (1.3%), black female (3.0%), and Hispanic female (2.5%) students, respectively; and higher among 9th-grade male (6.4%), 10th-grade male (6.9%), 11th-grade male (8.2%), and 12th-grade male (9.4%) than 9th-grade female (2.4%), 10th-grade female (1.4%), 11th-grade female (1.7%), and 12th-grade female (1.8%) students, respectively. The prevalence of having carried a gun was higher among 12th-grade (5.5%) than 10th-grade (4.1%) students and higher among 12th-grade male (9.4%) than 9th-grade male (6.4%) students.

Analyses based on the question ascertaining sexual identity indicated that nationwide, 4.8% of heterosexual students; 3.7% of gay, lesbian, and bisexual students; and 7.9% of not sure students had carried a gun (Supplementary Table 20). The prevalence of having carried a gun was higher among not sure (7.9%) than gay, lesbian, and bisexual (3.7%) students. Among male students, the prevalence was higher among not sure (12.0%) than gay and bisexual (4.7%) students. The prevalence also was higher among heterosexual male (7.6%) than heterosexual female (1.6%) students and higher among not sure male (12.0%) than not sure female (3.3%) students.

Analyses based on the question ascertaining the sex of sexual contacts indicated that nationwide, 7.2% of students who had sexual contact with only the opposite sex, 6.6% of students who had sexual contact with only the same sex or with both sexes, and 2.0% of students who had no sexual contact had carried a gun (Supplementary Table 20). The prevalence of having carried a gun was higher among students who had sexual contact with only the opposite sex (7.2%) and students who had sexual contact with only the same sex or with both sexes (6.6%) than students who had no sexual contact (2.0%). Among female students, the prevalence was higher among those who had sexual contact with only females or with both sexes (4.9%) than those who had sexual contact with only males (2.1%) or those who had no sexual contact (1.1%). Among male students, the prevalence was higher among those who had sexual contact with only females (11.4%) and those who had sexual contact with only males or with both sexes (11.7%) than those who had no sexual contact (2.9%). The prevalence also was higher among male students who had sexual contact with only females (11.4%) than female students who had sexual contact with only males (2.1%), higher among male students who had sexual contact with only males or with both sexes (11.7%) than female students who had sexual contact with only females or with both sexes (4.9%), and higher among male students who had no sexual contact (2.9%) than female students who had no sexual contact (1.1%).

The question measuring the prevalence of having carried a gun (not counting the days when they carried a gun only for hunting or for a sport, such as target shooting) during the 12 months before the survey was used for the first time in the 2017 national YRBS. As a result, long-term temporal trends and 2-year temporal changes are not available for this variable.

Analyses of state and large urban school district data indicated that across 22 states, the overall prevalence of having carried a gun ranged from 2.7% to 12.2% across state surveys (median: 6.0%) (Supplementary Table 21). Across 15 large urban school districts, the prevalence ranged from 3.4% to 10.8% (median: 5.9%).

#### Were Threatened or Injured with a Weapon on School Property

Nationwide, 6.0% of students had been threatened or injured with a weapon (e.g., a gun, knife, or club) on school property one or more times during the 12 months before the survey (Supplementary Table 22). The prevalence of having been threatened or injured with a weapon on school property was higher among male (7.8%) than female (4.1%) students; higher among white male (6.5%), black male (10.0%), and Hispanic male (8.3%) than white female (3.6%), black female (5.5%), and Hispanic female (3.8%) students, respectively; and higher among 9th-grade male (8.8%), 10th-grade male (8.5%), 11th-grade male (6.7%), and 12th-grade male (6.6%) than 9th-grade female (4.9%), 10th-grade female (5.0%), 11th-grade female (3.2%), and 12th-grade female (2.7%) students, respectively. The prevalence of having been threatened or injured with a weapon on school property was higher among black (7.8%) than white (5.0%) and Hispanic (6.1%) students and higher among black male (10.0%) and Hispanic male (8.3%) than white male (6.5%) students. The prevalence of having been threatened or injured with a weapon on school property was higher among 9th-grade (6.8%) and 10th-grade (6.8%) than 12th-grade (4.6%) students, higher among 9th-grade (6.8%) than 11th-grade (5.1%) students, higher among 9th-grade female (4.9%) and 10th-grade female (5.0%) than 12th-grade female (2.7%) students, and higher among 9th-grade female (4.9%) than 11th-grade female (3.2%) students.

Analyses based on the question ascertaining sexual identity indicated that nationwide, 5.4% of heterosexual students; 9.4% of gay, lesbian, and bisexual students; and 11.1% of not sure students had been threatened or injured with a weapon on school property (Supplementary Table 22). The prevalence of having been threatened or injured with a weapon on school property was higher among gay, lesbian, and bisexual (9.4%) and not sure (11.1%) than heterosexual (5.4%) students. Among female students, the prevalence was higher among lesbian and bisexual (7.4%) than heterosexual (3.6%) students. Among male students, the prevalence was higher among gay and bisexual (14.6%) and not sure (17.2%) than heterosexual (6.9%) students. The prevalence also was higher among heterosexual male (6.9%) than heterosexual female (3.6%) students, higher among gay and bisexual male (14.6%) than lesbian and bisexual female (7.4%) students, and higher among not sure male (17.2%) than not sure female (5.3%) students.

Analyses based on the question ascertaining the sex of sexual contacts indicated that nationwide, 7.6% of students who had sexual contact with only the opposite sex, 12.1% of students who had sexual contact with only the same sex or with both sexes, and 3.1% of students who had no sexual contact had been threatened or injured with a weapon on school property (Supplementary Table 22). The prevalence of having been threatened or injured with a weapon on school property was higher among students who had sexual contact with only the same sex or with both sexes (12.1%) and students who had sexual contact with only the opposite sex (7.6%) than students who had no sexual contact (3.1%) and higher among students who had sexual contact with only the same sex or with both sexes (12.1%) than students who had sexual contact with only the opposite sex (7.6%). Among female students, the prevalence was higher among those who had sexual contact with only males (4.9%) and those who had sexual contact with only females or with both sexes (8.8%) than those who had no sexual contact (2.7%) and higher among those who had sexual contact with only females or with both sexes (8.8%) than those who had sexual contact with only males (4.9%). Among male students, the prevalence was higher among those who had sexual contact with only females (9.9%) and those who had sexual contact with only males or with both sexes (21.5%) than those who had no sexual contact (3.6%) and higher among those who had sexual contact with only males or with both sexes (21.5%) than those who had sexual contact with only females (9.9%). The prevalence also was higher among male students who had sexual contact with only females (9.9%) than female students who had sexual contact with only males (4.9%) and higher among male students who had sexual contact with only males or with both sexes (21.5%) than female students who had sexual contact with only females or with both sexes (8.8%).

Trend analyses indicated that during 1993–2017, a significant linear decrease (7.3%–6.0%) occurred in the overall prevalence of having been threatened or injured with a weapon on school property. A significant quadratic trend was identified. The prevalence of having been threatened or injured with a weapon on school property did not change significantly during 1993–2003 (7.3%–9.2%) and then decreased during 2003–2017 (9.2%–6.0%). The prevalence of having been threatened or injured with a weapon on school property did not change significantly from 2015 (6.0%) to 2017 (6.0%).

Analyses of state and large urban school district data indicated that across 33 states, the overall prevalence of having been threatened or injured with a weapon on school property ranged from 4.8% to 12.8% across state surveys (median: 6.9%) (Supplementary Table 23). Across 21 large urban school districts, the prevalence ranged from 4.9% to 12.3% (median: 7.1%).

#### Were in a Physical Fight

Nationwide, 23.6% of students had been in a physical fight one or more times during the 12 months before the survey (Supplementary Table 24). The prevalence of having been in a physical fight was higher among male (30.0%) than female (17.2%) students; higher among white male (28.7%), black male (37.2%), and Hispanic male (29.9%) than white female (13.5%), black female (29.1%), and Hispanic female (21.1%) students, respectively; and higher among 9th-grade male (33.9%), 10th-grade male (34.7%), 11th-grade male (25.8%), and 12th-grade male (24.1%) than 9th-grade female (22.7%), 10th-grade female (18.0%), 11th-grade female (15.2%), and 12th-grade female (11.8%) students, respectively. The prevalence of having been in a physical fight was higher among black (33.2%) and Hispanic (25.7%) than white (20.8%) students, higher among black (33.2%) than Hispanic (25.7%) students, higher among black female (29.1%) and Hispanic female (21.1%) than white female (13.5%) students, higher among black female (29.1%) than Hispanic female (21.1%) students, and higher among black male (37.2%) than white male (28.7%) students. The prevalence of having been in a physical fight was higher among 9th-grade (28.3%) and 10th-grade (26.2%) than 11th-grade (20.4%) and 12th-grade (17.8%) students; higher among 9th-grade female (22.7%) than 10th-grade female (18.0%), 11th-grade female (15.2%), and 12th-grade female (11.8%) students; higher among 10th-grade female (18.0%) than 12th-grade female (11.8%) students; and higher among 9th-grade male (33.9%) and 10th-grade male (34.7%) than 11th-grade male (25.8%) and 12th-grade male (24.1%) students.

Analyses based on the question ascertaining sexual identity indicated that nationwide, 23.2% of heterosexual students; 27.9% of gay, lesbian, and bisexual students; and 19.8% of not sure students had been in a physical fight (Supplementary Table 24). The prevalence of having been in a physical fight was higher among gay, lesbian, and bisexual (27.9%) than heterosexual (23.2%) and not sure (19.8%) students. Among female students, the prevalence was higher among lesbian and bisexual (27.6%) than heterosexual (15.5%) and not sure (14.8%) students. The prevalence also was higher among heterosexual male (29.9%) than heterosexual female (15.5%) students and higher among not sure male (24.5%) than not sure female (14.8%) students.

Analyses based on the question ascertaining the sex of sexual contacts indicated that nationwide, 32.2% of students who had sexual contact with only the opposite sex, 36.6% of students who had sexual contact with only the same sex or with both sexes, and 13.4% of students who had no sexual contact had been in a physical fight (Supplementary Table 24). The prevalence of having been in a physical fight was higher among students who had sexual contact with only the same sex or with both sexes (36.6%) than students who had sexual contact with only the opposite sex (32.2%) and students who had no sexual contact (13.4%) and higher among students who had sexual contact with only the opposite sex (32.2%) than students who had no sexual contact (13.4%). Among female students, the prevalence was higher among those who had sexual contact with only females or with both sexes (35.9%) than students who had sexual contact with only males (20.9%) or students who had no sexual contact (10.2%) and higher among students who had sexual contact with only males (20.9%) than students who had no sexual contact (10.2%). Among male students, the prevalence was higher among those who had sexual contact with only females (41.6%) and those who had sexual contact with only males or with both sexes (38.5%) than those who had no sexual contact (16.8%). The prevalence also was higher among male students who had sexual contact with only females (41.6%) than female students who had sexual contact with only males (20.9%) and higher among male students who had no sexual contact (16.8%) than female students who had no sexual contact (10.2%).

Trend analyses indicated that during 1991–2017, a significant linear decrease (42.5%–23.6%) occurred in the overall prevalence of having been in a physical fight. A significant quadratic trend was identified. The prevalence of having been in a physical fight decreased during 1991–2011 (42.5%–32.8%) and then decreased more rapidly during 2011–2017 (32.8%–23.6%). The prevalence of having been in a physical fight did not change significantly from 2015 (22.6%) to 2017 (23.6%).

Analyses of state and large urban school district data indicated that across 36 states, the overall prevalence of having been in a physical fight ranged from 15.3% to 30.6% across state surveys (median: 20.1%) (Supplementary Table 25). Across 20 large urban school districts, the prevalence ranged from 15.4% to 39.1% (median: 24.5%).

#### Were in a Physical Fight on School Property

Nationwide, 8.5% of students had been in a physical fight on school property one or more times during the 12 months before the survey (Supplementary Table 26). The prevalence of having been in a physical fight on school property was higher among male (11.6%) than female (5.6%) students; higher among white male (10.1%) and Hispanic male (11.6%) than white female (3.1%) and Hispanic female (7.0%) students, respectively; and higher among 9th-grade male (16.9%), 10th-grade male (13.5%), 11th-grade male (7.5%), and 12th-grade male (6.5%) than 9th-grade female (7.7%), 10th-grade female (5.8%), 11th-grade female (4.5%), and 12th-grade female (3.6%) students, respectively. The prevalence of having been in a physical fight on school property was higher among black (15.3%) and Hispanic (9.4%) than white (6.5%) students, higher among black (15.3%) than Hispanic (9.4%) students, higher among black female (13.7%) and Hispanic female (7.0%) than white female (3.1%) students, higher among black female (13.7%) than Hispanic female (7.0%) students, and higher among black male (16.9%) than white male (10.1%) and Hispanic male (11.6%) students. The prevalence of having been in a physical fight on school property was higher among 9th-grade (12.3%) than 10th-grade (9.6%), 11th-grade (6.0%), and 12th-grade (5.0%) students; higher among 10th-grade (9.6%) than 11th-grade (6.0%) and 12th-grade (5.0%) students; higher among 9th-grade female (7.7%) and 10th-grade female (5.8%) than 12th-grade female (3.6%) students; and higher among 9th-grade male (16.9%) and 10th-grade male (13.5%) than 11th-grade male (7.5%) and 12th-grade male (6.5%) students.

Analyses based on the question ascertaining sexual identity indicated that nationwide, 8.3% of heterosexual students; 9.6% of gay, lesbian, and bisexual students; and 11.8% of not sure students had been in a physical fight on school property (Supplementary Table 26). Among female students, the prevalence of having been in a physical fight on school property was higher among lesbian and bisexual (8.9%) than heterosexual (4.9%) students. The prevalence also was higher among heterosexual male (11.3%) than heterosexual female (4.9%) students and higher among not sure male (16.4%) than not sure female (7.3%) students.

Analyses based on the question ascertaining the sex of sexual contacts indicated that nationwide, 12.2% of students who had sexual contact with only the opposite sex, 12.7% of students who had sexual contact with only the same sex or with both sexes, and 4.0% of students who had no sexual contact had been in a physical fight on school property (Supplementary Table 26). The prevalence of having been in a physical fight on school property was higher among students who had sexual contact with only the opposite sex (12.2%) and students who had sexual contact with only the same sex or with both sexes (12.7%) than students who had no sexual contact (4.0%). Among female students, the prevalence was higher among those who had sexual contact with only males (7.3%) and those who had sexual contact with only females or with both sexes (10.3%) than students who had no sexual contact (2.7%). Among male students, the prevalence was higher among those who had sexual contact with only females (16.2%) and those who had sexual contact with only males or with both sexes (19.6%) than those who had no sexual contact (5.5%). The prevalence also was higher among male students who had sexual contact with only females (16.2%) than female students who had sexual contact with only males (7.3%) and higher among male students who had no sexual contact (5.5%) than female students who had no sexual contact (2.7%).

Trend analyses indicated that during 1993–2017, a significant linear decrease (16.2%–8.5%) occurred in the overall prevalence of having been in a physical fight on school property. A significant quadratic trend was not identified. The prevalence of having been in a physical fight on school property did not change significantly from 2015 (7.8%) to 2017 (8.5%).

Analyses of state and large urban school district data indicated that across 32 states, the overall prevalence of having been in a physical fight on school property ranged from 4.6% to 12.3% across state surveys (median: 7.3%) (Supplementary Table 27). Across 17 large urban school districts, the prevalence ranged from 6.2% to 17.9% (median: 9.5%).

#### Were Electronically Bullied

Nationwide, 14.9% of students had been electronically bullied (counting being bullied through texting, Instagram, Facebook, or other social media) during the 12 months before the survey (Supplementary Table 28). The prevalence of having been electronically bullied was higher among female (19.7%) than male (9.9%) students; higher among white female (23.0%), black female (13.3%), and Hispanic female (17.2%) than white male (11.2%), black male (8.4%), and Hispanic male (7.6%) students, respectively; and higher among 9th-grade female (22.3%), 10th-grade female (19.7%), 11th-grade female (19.9%), and 12th-grade female (16.4%) than 9th-grade male (10.9%), 10th-grade male (9.7%), 11th-grade male (8.2%), and 12th-grade male (10.4%) students, respectively. The prevalence of having been electronically bullied was higher among white (17.3%) than black (10.9%) and Hispanic (12.3%) students, higher among white female (23.0%) and Hispanic female (17.2%) than black female (13.3%) students, higher among white female (23.0%) than Hispanic female (17.2%) students, and higher among white male (11.2%) than black male (8.4%) and Hispanic male (7.6%) students. The prevalence of having been electronically bullied was higher among 9th-grade (16.7%) than 10th-grade (14.8%) and 12th-grade (13.5%) students, higher among 9th-grade female (22.3%) and 10th-grade female (19.7%) than 12th-grade female (16.4%) students, and higher among 9th-grade male (10.9%) than 11th-grade male (8.2%) students.

Analyses based on the question ascertaining sexual identity indicated that nationwide, 13.3% of heterosexual students; 27.1% of gay, lesbian, and bisexual students; and 22.0% of not sure students had been electronically bullied (Supplementary Table 28). The prevalence of having been electronically bullied was higher among gay, lesbian, and bisexual (27.1%) and not sure (22.0%) than heterosexual (13.3%) students. Among female students, the prevalence was higher among lesbian and bisexual (28.5%) than heterosexual (18.6%) students. Among male students, the prevalence was higher among gay and bisexual (22.3%) and not sure (18.2%) than heterosexual (8.8%) students. The prevalence also was higher among heterosexual female (18.6%) than heterosexual male (8.8%) students and higher among lesbian and bisexual female (28.5%) than gay and bisexual male (22.3%) students.

Analyses based on the question ascertaining the sex of sexual contacts indicated that nationwide, 17.7% of students who had sexual contact with only the opposite sex, 31.4% of students who had sexual contact with only the same sex or with both sexes, and 10.5% of students who had no sexual contact had been electronically bullied (Supplementary Table 28). The prevalence of having been electronically bullied was higher among students who had sexual contact with only the opposite sex (17.7%) and students who had sexual contact with only the same sex or with both sexes (31.4%) than students who had no sexual contact (10.5%) and higher among students who had sexual contact with only the same sex or with both sexes (31.4%) than students who had sexual contact with only the opposite sex (17.7%). Among female students, the prevalence was higher among those who had sexual contact with only males (26.6%) and those who had sexual contact with only females or with both sexes (32.0%) than those who had no sexual contact (13.3%). Among male students, the prevalence was higher among those who had sexual contact with only females (10.5%) and those who had sexual contact with only males or with both sexes (29.7%) than those who had no sexual contact (7.4%) and higher among those who had sexual contact with only males or with both sexes (29.7%) than those who had sexual contact with only females (10.5%). The prevalence also was higher among female students who had sexual contact with only males (26.6%) than male students who had sexual contact with only females (10.5%) and higher among female students who had no sexual contact (13.3%) than male students who had no sexual contact (7.4%).

Trend analyses did not identify a significant linear trend in the overall prevalence of having been electronically bullied during 2011–2017 (16.2%–14.9%). Not enough data points were available to identify a quadratic trend. The prevalence of having been electronically bullied did not change significantly from 2015 (15.5%) to 2017 (14.9%).

Analyses of state and large urban school district data indicated that across 39 states, the overall prevalence of having been electronically bullied ranged from 10.1% to 21.2% across state surveys (median: 16.1%) (Supplementary Table 29). Across 21 large urban school districts, the prevalence ranged from 8.8% to 16.0% (median: 11.7%).

#### Were Bullied on School Property

Nationwide, 19.0% of students had been bullied on school property during the 12 months before the survey (Supplementary Table 30). The prevalence of having been bullied on school property was higher among female (22.3%) than male (15.6%) students; higher among white female (24.6%) and Hispanic female (21.0%) than white male (18.1%) and Hispanic male (11.8%) students, respectively; and higher among 9th-grade female (25.2%), 10th-grade female (23.6%), 11th-grade female (23.5%), and 12th-grade female (16.3%) than 9th-grade male (20.0%), 10th-grade male (16.8%), 11th-grade male (12.8%), and 12th-grade male (11.6%) students, respectively. The prevalence of having been bullied on school property was higher among white (21.5%) and Hispanic (16.3%) than black (13.2%) students, higher among white (21.5%) than Hispanic (16.3%) students, higher among white female (24.6%) and Hispanic female (21.0%) than black female (14.5%) students and higher among white male (18.1%) than black male (11.8%) and Hispanic male (11.8%) students. The prevalence of having been bullied on school property was higher among 9th-grade (22.7%), 10th-grade (20.3%), and 11th-grade (18.3%) than 12th-grade (14.0%) students; higher among 9th-grade (22.7%) than 11th-grade (18.3%) students; higher among 9th-grade female (25.2%), 10th-grade female (23.6%), and 11th-grade female (23.5%) than 12th-grade female (16.3%) students; and higher among 9th-grade male (20.0%) and 10th-grade male (16.8%) than 11th-grade male (12.8%) and 12th-grade male (11.6%) students.

Analyses based on the question ascertaining sexual identity indicated that nationwide, 17.1% of heterosexual students; 33.0% of gay, lesbian, and bisexual students; and 24.3% of not sure students had been bullied on school property (Supplementary Table 30). The prevalence of having been bullied on school property was higher among gay, lesbian, and bisexual (33.0%) than heterosexual (17.1%) and not sure (24.3%) students and higher among not sure (24.3%) than heterosexual (17.1%) students. Among female students, the prevalence was higher among lesbian and bisexual (32.2%) than heterosexual (20.5%) and not sure (25.2%) students. Among male students, the prevalence was higher among gay and bisexual (35.0%) and not sure (21.5%) than heterosexual (14.2%) students. The prevalence also was higher among heterosexual female (20.5%) than heterosexual male (14.2%) students.

Analyses based on the question ascertaining the sex of sexual contacts indicated that nationwide, 19.3% of students who had sexual contact with only the opposite sex, 35.8% of students who had sexual contact with only the same sex or with both sexes, and 16.8% of students who had no sexual contact had been bullied on school property (Supplementary Table 30). The prevalence of having been bullied on school property was higher among students who had sexual contact with only the opposite sex (19.3%) and students who had sexual contact with only the same sex or with both sexes (35.8%) than students who had no sexual contact (16.8%) and higher among students who had sexual contact with only the same sex or with both sexes (35.8%) than students who had sexual contact with only the opposite sex (19.3%). Among female students, the prevalence was higher among those who had sexual contact with only males (25.4%) and those who had sexual contact with only females or with both sexes (35.9%) than those who had no sexual contact (18.1%) and higher among those who had sexual contact with only females or with both sexes (35.9%) than those who had sexual contact with only males (25.4%). Among male students, the prevalence was higher among those who had sexual contact with only males or with both sexes (35.5%) than those who had sexual contact with only females (14.4%) and those who had no sexual contact (15.4%). The prevalence also was higher among female students who had sexual contact with only males (25.4%) than male students who had sexual contact with only females (14.4%) and higher among female students who had no sexual contact (18.1%) than male students who had no sexual contact (15.4%).

Trend analyses did not identify a significant linear trend in the overall prevalence of having been bullied on school property during 2009–2017 (19.9%–19.0%). Not enough data points were available to identify a quadratic trend. The prevalence of having been bullied on school property did not change significantly from 2015 (20.2%) to 2017 (19.0%).

Analyses of state and large urban school district data indicated that across 38 states, the overall prevalence of having been bullied on school property ranged from 14.1% to 26.7% across state surveys (median: 21.2%) (Supplementary Table 31). Across 21 large urban school districts, the prevalence ranged from 10.6% to 19.7% (median: 13.9%).

#### Did Not Go to School Because of Safety Concerns

Nationwide, 6.7% of students had not gone to school on at least 1 day during the 30 days before the survey because they felt they would be unsafe at school or on their way to or from school (i.e., did not go to school because of safety concerns) (Supplementary Table 32). The prevalence of having not gone to school because of safety concerns was higher among white female (5.7%) than white male (3.9%) students. The prevalence of having not gone to school because of safety concerns was higher among black (9.0%) and Hispanic (9.4%) than white (4.9%) students, higher among black female (9.5%) and Hispanic female (9.3%) than white female (5.7%) students, and higher among black male (8.2%) and Hispanic male (9.4%) than white male (3.9%) students. The prevalence of having not gone to school because of safety concerns was higher among 9th-grade (7.6%) and 10th-grade (7.9%) than 11th-grade (5.4%) and 12th-grade (5.2%) students, higher among 9th-grade female (8.7%) and 10th-grade female (8.6%) than 11th-grade female (5.7%) and 12th-grade female (4.7%) students, and higher among 10th-grade male (7.2%) than 11th-grade male (4.8%) students.

Analyses based on the question ascertaining sexual identity indicated that nationwide, 6.1% of heterosexual students; 10.0% of gay, lesbian, and bisexual students; and 10.7% of not sure students did not go to school because of safety concerns (Supplementary Table 32). The prevalence of having not gone to school because of safety concerns was higher among gay, lesbian, and bisexual (10.0%) and not sure (10.7%) than heterosexual (6.1%) students. Among male students, the prevalence was higher among gay and bisexual (12.3%) and not sure (12.6%) than heterosexual (5.5%) students. The prevalence also was higher among heterosexual female (6.7%) than heterosexual male (5.5%) students.

Analyses based on the question ascertaining the sex of sexual contacts indicated that nationwide, 7.9% of students who had sexual contact with only the opposite sex, 11.5% of students who had sexual contact with only the same sex or with both sexes, and 4.5% of students who had no sexual contact did not go to school because of safety concerns (Supplementary Table 32). The prevalence of having not gone to school because of safety concerns was higher among students who had sexual contact with only the opposite sex (7.9%) and students who had sexual contact with only the same sex or with both sexes (11.5%) than students who had no sexual contact (4.5%) and higher among students who had sexual contact with only the same sex or with both sexes (11.5%) than students who had sexual contact with only the opposite sex (7.9%). Among female students, the prevalence was higher among those who had sexual contact with only males (8.0%) and those who had sexual contact with only females or with both sexes (11.4%) than those who had no sexual contact (5.5). Among male students, the prevalence was higher among those who had sexual contact with only females (7.8%) and those who had sexual contact with only males or with both sexes (11.8%) than those who had no sexual contact (3.5%). The prevalence also was higher among female students who had no sexual contact (5.5%) than male students who had no sexual contact (3.5%).

Trend analyses indicated that during 1993–2017, a significant linear increase (4.4%–6.7%) occurred in the overall prevalence of having not gone to school because of safety concerns. A significant quadratic trend was not identified. The prevalence of having not gone to school because of safety concerns did not change significantly from 2015 (5.6%) to 2017 (6.7%).

Analyses of state and large urban school district data indicated that across 36 states, the overall prevalence of having not gone to school because of safety concerns ranged from 4.5% to 11.8% across state surveys (median: 7.3%) (Supplementary Table 33). Across 20 large urban school districts, the prevalence ranged from 5.8% to 13.3% (median: 9.6%).

#### Were Physically Forced to Have Sexual Intercourse

Nationwide, 7.4% of students had ever been physically forced to have sexual intercourse when they did not want to (Supplementary Table 34). The prevalence of having been forced to have sexual intercourse was higher among female (11.3%) than male (3.5%) students; higher among white female (11.2%), black female (11.7%), and Hispanic female (11.2%) than white male (3.3%), black male (3.4%), and Hispanic male (3.6%) students, respectively; and higher among 9th-grade female (8.1%), 10th-grade female (11.2%), 11th-grade female (12.1%), and 12th-grade female (13.9%) than 9th-grade male (2.7%), 10th-grade male (3.5%), 11th-grade male (2.8%), and 12th-grade male (4.8%) students, respectively. The prevalence of having been forced to have sexual intercourse was higher among 10th-grade (7.4%), 11th-grade (7.5%), and 12th-grade (9.4%) than 9th-grade (5.4%) students; higher among 12th-grade (9.4%) than 10th-grade (7.4%) students; higher among 10th-grade female (11.2%), 11th-grade female (12.1%), and 12th-grade female (13.9%) than 9th-grade female (8.1%) students; and higher among 12th-grade male (4.8%) than 9th-grade male (2.7%) students.

Analyses based on the question ascertaining sexual identity indicated that nationwide, 5.4% of heterosexual students; 21.9% of gay, lesbian, and bisexual students; and 13.1% of not sure students had ever been physically forced to have sexual intercourse when they did not want to (Supplementary Table 34). The prevalence of having been forced to have sexual intercourse was higher among gay, lesbian, and bisexual (21.9%) than heterosexual (5.4%) and not sure (13.1%) students and higher among not sure (13.1%) than heterosexual (5.4%) students. Among female students, the prevalence was higher among lesbian and bisexual (23.7%) than heterosexual (8.8%) and not sure (12.7%) students. Among male students, the prevalence was higher among gay and bisexual (15.6%) and not sure (11.8%) than heterosexual (2.5%) students. The prevalence also was higher among heterosexual female (8.8%) than heterosexual male (2.5%) students and higher among lesbian and bisexual female (23.7%) than gay and bisexual male (15.6%) students.

Analyses based on the question ascertaining the sex of sexual contacts indicated that nationwide, 9.9% of students who had sexual contact with only the opposite sex, 30.3% of students who had sexual contact with only the same sex or with both sexes, and 1.5% of students who had no sexual contact had ever been physically forced to have sexual intercourse when they did not want to (Supplementary Table 34). The prevalence of having been forced to have sexual intercourse was higher among students who had sexual contact with only the opposite sex (9.9%) and students who had sexual contact with only the same sex or with both sexes (30.3%) than students who had no sexual contact (1.5%) and higher among students who had sexual contact with only the same sex or with both sexes (30.3%) than students who had sexual contact with only the opposite sex (9.9%). Among female students, the prevalence was higher among those who had sexual contact with only males (17.5%) and those who had sexual contact with only females or with both sexes (31.7%) than those who had no sexual contact (2.1%) and higher among those who had sexual contact with only females or with both sexes (31.7%) than those who had sexual contact with only males (17.5%). Among male students, the prevalence was higher among those who had sexual contact with only females (3.6%) and those who had sexual contact with only males or with both sexes (26.4%) than those who had no sexual contact (0.8%) and higher among those who had sexual contact with only males or with both sexes (26.4%) than those who had sexual contact with only females (3.6%). The prevalence also was higher among female students who had sexual contact with only males (17.5%) than male students who had sexual contact with only females (3.6%) and higher among female students who had no sexual contact (2.1%) than male students who had no sexual contact (0.8%).

Trend analyses indicated that during 2001–2017, a significant linear decrease (7.7%–7.4%) occurred in the overall prevalence of having been forced to have sexual intercourse. A significant quadratic trend was not identified. The prevalence of having been forced to have sexual intercourse did not change significantly from 2015 (6.7%) to 2017 (7.4%).

Analyses of state and large urban school district data indicated that across 34 states, the overall prevalence of having been forced to have sexual intercourse ranged from 5.7% to 19.2% across state surveys (median: 8.3%) (Supplementary Table 35). Across 20 large urban school districts, the prevalence ranged from 6.8% to 11.9% (median: 9.2%).

#### Experienced Sexual Violence by Anyone

Nationwide, 9.7% of students had been forced to do “sexual things” (e.g., kissing, touching, or being physically forced to have sexual intercourse) they did not want to do one or more times during the 12 months before the survey by anyone (i.e., sexual violence) (Supplementary Table 36). The prevalence of having experienced sexual violence by anyone was higher among female (15.2%) than male (4.3%) students; higher among white female (16.6%), black female (11.0%), and Hispanic female (15.1%) than white male (3.5%), black male (5.8%), and Hispanic male (4.2%) students, respectively; and higher among 9th-grade female (14.7%), 10th-grade female (15.3%), 11th-grade female (16.1%), and 12th-grade female (14.4%) than 9th-grade male (3.8%), 10th-grade male (4.4%), 11th-grade male (4.1%), and 12th-grade male (4.7%) students, respectively. The prevalence of having experienced sexual violence was higher among white female (16.6%) and Hispanic female (15.1%) than black female (11.0%) students.

Analyses based on the question ascertaining sexual identity indicated that nationwide, 7.9% of heterosexual students; 22.2% of gay, lesbian, and bisexual students; and 16.7% of not sure students had experienced sexual violence by anyone (Supplementary Table 36). The prevalence of having experienced sexual violence by anyone was higher among gay, lesbian, and bisexual (22.2%) and not sure (16.7%) than heterosexual (7.9%) students and higher among gay, lesbian, and bisexual (22.2%) than not sure (16.7%) students. Among female students, the prevalence was higher among lesbian and bisexual (22.8%) and not sure (18.9%) than heterosexual (13.4%) students. Among male students, the prevalence was higher among gay and bisexual (19.6%) and not sure (11.3%) than heterosexual (3.1%) students. The prevalence also was higher among heterosexual female (13.4%) than heterosexual male (3.1%) students and higher among not sure female (18.9%) than not sure male (11.3%) students.

Analyses based on the question ascertaining the sex of sexual contacts indicated that nationwide, 12.0% of students who had sexual contact with only the opposite sex, 31.4% of students who had sexual contact with only the same sex or with both sexes, and 4.3% of students who had no sexual contact had experienced sexual violence by anyone (Supplementary Table 36). The prevalence of having experienced sexual violence by anyone was higher among students who had sexual contact with only the opposite sex (12.0%) and students who had sexual contact with only the same sex or with both sexes (31.4%) than students who had no sexual contact (4.3%) and higher among students who had sexual contact with only the same sex or with both sexes (31.4%) than students who had sexual contact with only the opposite sex (12.0%). Among female students, the prevalence was higher among those who had sexual contact with only males (21.2%) and those who had sexual contact with only females or with both sexes (33.1%) than those who had no sexual contact (7.0%) and higher among those who had sexual contact with only females or with both sexes (33.1%) than those who had sexual contact with only males (21.2%). Among male students, the prevalence was higher among those who had sexual contact with only females (4.7%) and those who had sexual contact with only males or with both sexes (26.4%) than those who had no sexual contact (1.4%) and higher among those who had sexual contact with only males or with both sexes (26.4%) than those who had sexual contact with only females (4.7%). The prevalence also was higher among female students who had sexual contact with only males (21.2%) than male students who had sexual contact with only females (4.7%) and higher among female students who had no sexual contact (7.0%) than male students who had no sexual contact (1.4%).

The question measuring the prevalence of having experienced sexual violence by anyone was used for the first time in the 2017 national YRBS. As a result, long-term temporal trends and 2-year temporal changes are not available for this variable.

Analyses of state and large urban school district data indicated that across 26 states, the overall prevalence of having experienced sexual violence by anyone ranged from 7.7% to 18.5% across state surveys (median: 10.5%) (Supplementary Table 37). Across 15 large urban school districts, the prevalence ranged from 8.4% to 14.1% (median: 11.0%).

#### Experienced Sexual Dating Violence

Among the 68.3% of students nationwide who dated or went out with someone during the 12 months before the survey,^¶^ 6.9% had been forced to do “sexual things” (e.g., kissing, touching, or being physically forced to have sexual intercourse) they did not want to do one or more times during the 12 months before the survey by someone they were dating or going out with (i.e., sexual dating violence) (Supplementary Table 38). The prevalence of having experienced sexual dating violence was higher among female (10.7%) than male (2.8%) students; higher among white female (11.1%), black female (6.8%), and Hispanic female (11.4%) than white male (2.6%), black male (2.7%), and Hispanic male (2.5%) students, respectively; and higher among 9th-grade female (11.0%), 10th-grade female (10.6%), 11th-grade female (11.5%), and 12th-grade female (9.4%) than 9th-grade male (2.2%), 10th-grade male (2.9%), 11th-grade male (1.8%), and 12th-grade male (4.0%) students, respectively. The prevalence of having experienced sexual dating violence was higher among white (6.9%) and Hispanic (6.9%) than black (4.8%) students and higher among white female (11.1%) and Hispanic female (11.4%) than black female (6.8%) students. The prevalence of having experienced sexual dating violence was higher among 12th-grade male (4.0%) than 11th-grade male (1.8%) students.

Analyses based on the question ascertaining sexual identity indicated that nationwide, among the students who dated or went out with someone during the 12 months before the survey, 5.5% of heterosexual students; 15.8% of gay, lesbian, and bisexual students; and 14.1% of not sure students had experienced sexual dating violence (Supplementary Table 38). The prevalence of having experienced sexual dating violence was higher among gay, lesbian, and bisexual (15.8%) and not sure (14.1%) than heterosexual (5.5%) students. Among female students, the prevalence was higher among lesbian and bisexual (16.3%) than heterosexual (9.3%) students. Among male students, the prevalence was higher among gay and bisexual (13.5%) than heterosexual (2.1%) students. The prevalence also was higher among heterosexual female (9.3%) than heterosexual male (2.1%) students.

Analyses based on the question ascertaining the sex of sexual contacts indicated that nationwide, among the students who dated or went out with someone during the 12 months before the survey, 7.2% of students who had sexual contact with only the opposite sex, 19.5% of students who had sexual contact with only the same sex or with both sexes, and 3.5% of students who had no sexual contact had experienced sexual dating violence (Supplementary Table 38). The prevalence of having experienced sexual dating violence was higher among students who had sexual contact with only the opposite sex (7.2%) and students who had sexual contact with only the same sex or with both sexes (19.5%) than students who had no sexual contact (3.5%) and higher among students who had sexual contact with only the same sex or with both sexes (19.5%) than students who had sexual contact with only the opposite sex (7.2%). Among female students, the prevalence was higher among those who had sexual contact with only males (12.4%) and those who had sexual contact with only females or with both sexes (19.2%) than those who had no sexual contact (6.0%) and higher among those who had sexual contact with only females or with both sexes (19.2%) than those who had sexual contact with only males (12.4%). Among male students, the prevalence was higher among those who had sexual contact with only females (2.8%) and those who had sexual contact with only males or with both sexes (20.2%) than those who had no sexual contact (0.6%) and higher among those who had sexual contact with only males or with both sexes (20.2%) than those who had sexual contact with only females (2.8%). The prevalence also was higher among female students who had sexual contact with only males (12.4%) than male students who had sexual contact with only females (2.8%) and higher among female students who had no sexual contact (6.0%) than male students who had no sexual contact (0.6%).

Trend analyses indicated that during 2013–2017, a significant linear decrease (10.4%–6.9%) occurred in the overall prevalence of having experienced sexual dating violence, among the students who dated or went out with someone during the 12 months before the survey. Not enough data points were available to identify a quadratic trend. The prevalence of having experienced sexual dating violence decreased significantly from 2015 (10.6%) to 2017 (6.9%).

Analyses of state and large urban school district data indicated that across 27 states, the overall prevalence of having experienced sexual dating violence, among the students who dated or went out with someone during the 12 months before the survey, ranged from 5.2% to 12.0% across state surveys (median: 7.3%) (Supplementary Table 39). Across 19 large urban school districts, the prevalence ranged from 3.5% to 15.4% (median: 5.8%).

#### Experienced Physical Dating Violence

Among the 69.0% of students nationwide who dated or went out with someone during the 12 months before the survey,^¶^ 8.0% had been physically hurt on purpose (e.g., being hit, slammed into something, or injured with an object or weapon) one or more times during the 12 months before the survey by someone they were dating or going out with (i.e., physical dating violence) (Supplementary Table 40). The prevalence of having experienced physical dating violence was higher among female (9.1%) than male (6.5%) students; higher among black female (13.1%) and Hispanic female (9.2%) than black male (7.1%) and Hispanic male (5.9%) students, respectively; and higher among 9th-grade female (8.1%), 10th-grade female (10.1%), and 11th-grade female (8.4%) than 9th-grade male (5.6%), 10th-grade male (6.5%), and 11th-grade male (4.8%) students, respectively. The prevalence of having experienced physical dating violence was higher among black (10.2%) than white (7.0%) and Hispanic (7.6%) students and higher among black female (13.1%) than white female (8.0%) students. The prevalence of having experienced physical dating violence was higher among 12th-grade (9.2%) than 9th-grade (7.0%) and 11th-grade (6.8%) students and higher among 12th-grade male (8.9%) than 9th-grade male (5.6%) and 11th-grade male (4.8%) students.

Analyses based on the question ascertaining sexual identity indicated that nationwide, among the students who dated or went out with someone during the 12 months before the survey, 6.4% of heterosexual students; 17.2% of gay, lesbian, and bisexual students; and 14.1% of not sure students had experienced physical dating violence (Supplementary Table 40). The prevalence of having experienced physical dating violence was higher among gay, lesbian, and bisexual (17.2%) and not sure (14.1%) than heterosexual (6.4%) students. Among female students, the prevalence was higher among lesbian and bisexual (16.9%) than heterosexual (7.1%) students. Among male students, the prevalence was higher among gay and bisexual (16.8%) and not sure (14.1%) than heterosexual (5.8%) students.

Analyses based on the question ascertaining the sex of sexual contacts indicated that nationwide, among the students who dated or went out with someone during the 12 months before the survey, 9.1% of students who had sexual contact with only the opposite sex, 20.2% of students who had sexual contact with only the same sex or with both sexes, and 2.4% of students who had no sexual contact had experienced physical dating violence (Supplementary Table 40). The prevalence of having experienced physical dating violence was higher among students who had sexual contact with only the opposite sex (9.1%) and students who had sexual contact with only the same sex or with both sexes (20.2%) than students who had no sexual contact (2.4%) and higher among students who had sexual contact with only the same sex or with both sexes (20.2%) than students who had sexual contact with only the opposite sex (9.1%). Among female students, the prevalence was higher among those who had sexual contact with only males (10.5%) and those who had sexual contact with only females or with both sexes (19.8%) than those who had no sexual contact (2.9%) and higher among those who had sexual contact with only females or with both sexes (19.8%) than those who had sexual contact with only males (10.5%). Among male students, the prevalence was higher among those who had sexual contact with only females (7.9%) and those who had sexual contact with only males or with both sexes (21.4%) than those who had no sexual contact (1.8%) and higher among those who had sexual contact with only males or with both sexes (21.4%) than those who had sexual contact with only females (7.9%). The prevalence also was higher among female students who had sexual contact with only males (10.5%) than male students who had sexual contact with only females (7.9%).

Trend analyses indicated that during 2013–2017, a significant linear decrease (10.3%–8.0%) occurred in the overall prevalence of having experienced physical dating violence, among the students who dated or went out with someone during the 12 months before the survey. Not enough data points were available to identify a quadratic trend. The prevalence of having experienced physical dating violence decreased significantly from 2015 (9.6%) to 2017 (8.0%).

Analyses of state and large urban school district data indicated that across 36 states, the overall prevalence of having experienced physical dating violence, among the students who dated or went out with someone during the 12 months before the survey, ranged from 5.5% to 12.1% across state surveys (median: 8.4%) (Supplementary Table 41). Across 21 large urban school districts, the prevalence ranged from 5.2% to 14.1% (median: 8.7%).

#### Felt Sad or Hopeless

During the 12 months before the survey, 31.5% of students nationwide had felt so sad or hopeless almost every day for 2 or more weeks in a row that they stopped doing some usual activities (Supplementary Table 42). The prevalence of having felt sad or hopeless was higher among female (41.1%) than male (21.4%) students; higher among white female (38.2%), black female (40.7%), and Hispanic female (46.8%) than white male (21.4%), black male (17.3%), and Hispanic male (21.2%) students, respectively; and higher among 9th-grade female (40.0%), 10th-grade female (43.1%), 11th-grade female (43.6%), and 12th-grade female (37.5%) than 9th-grade male (19.5%), 10th-grade male (21.5%), 11th-grade male (20.9%), and 12th-grade male (24.1%) students, respectively. The prevalence of having felt sad or hopeless was higher among Hispanic (33.7%) than white (30.2%) and black (29.2%) students and higher among Hispanic female (46.8%) than white female (38.2%) students. The prevalence of having felt sad or hopeless was higher among 10th-grade female (43.1%) and 11th-grade female (43.6%) than 12th-grade female (37.5%) students and higher among 12th-grade male (24.1%) than 9th-grade male (19.5%) students.

Analyses based on the question ascertaining sexual identity indicated that nationwide, 27.5% of heterosexual students; 63.0% of gay, lesbian, and bisexual students; and 46.4% of not sure students had felt sad or hopeless (Supplementary Table 42). The prevalence of having felt sad or hopeless was higher among gay, lesbian, and bisexual (63.0%) and not sure (46.4%) than heterosexual (27.5%) students and higher among gay, lesbian, and bisexual (63.0%) than not sure (46.4%) students. Among female students, the prevalence was higher among lesbian and bisexual (68.8%) and not sure (51.9%) than heterosexual (36.8%) students and higher among lesbian and bisexual (68.8%) than not sure (51.9%) students. Among male students, the prevalence was higher among gay and bisexual (45.5%) and not sure (36.4%) than heterosexual (19.5%) students. The prevalence also was higher among heterosexual female (36.8%) than heterosexual male (19.5%) students, higher among lesbian and bisexual female (68.8%) than gay and bisexual male (45.5%) students, and higher among not sure female (51.9%) than not sure male (36.4%) students.

Analyses based on the question ascertaining the sex of sexual contacts indicated that nationwide, 34.8% of students who had sexual contact with only the opposite sex, 63.9% of students who had sexual contact with only the same sex or with both sexes, and 25.4% of students who had no sexual contact had felt sad or hopeless (Supplementary Table 42). The prevalence of having felt sad or hopeless was higher among students who had sexual contact with only the opposite sex (34.8%) and students who had sexual contact with only the same sex or with both sexes (63.9%) than students who had no sexual contact (25.4%) and higher among students who had sexual contact with only the same sex or with both sexes (63.9%) than students who had sexual contact with only the opposite sex (34.8%). Among female students, the prevalence was higher among those who had sexual contact with only males (48.4%) and those who had sexual contact with only females or with both sexes (68.9%) than those who had no sexual contact (33.2%) and higher among those who had sexual contact with only females or with both sexes (68.9%) than those who had sexual contact with only males (48.4%). Among male students, the prevalence was higher among those who had sexual contact with only females (23.6%) and those who had sexual contact with only males or with both sexes (49.8%) than those who had no sexual contact (17.0%) and higher among those who had sexual contact with only males or with both sexes (49.8%) than those who had sexual contact with only females (23.6%). The prevalence also was higher among female students who had sexual contact with only males (48.4%) than male students who had sexual contact with only females (23.6%), higher among female students who had sexual contact with only females or with both sexes (68.9%) than male students who had sexual contact with only males or with both sexes (49.8%), and higher among female students who had no sexual contact (33.2%) than male students who had no sexual contact (17.0%).

Trend analyses indicated that during 1999–2017, a significant linear increase (28.3%–31.5%) occurred in the overall prevalence of having felt sad or hopeless. A significant quadratic trend also was identified. The prevalence of having felt sad or hopeless decreased during 1999–2009 (28.3%–26.1%) and then increased during 2009–2017 (26.1%–31.5%). The prevalence of having felt sad or hopeless did not change significantly from 2015 (29.9%) to 2017 (31.5%).

Analyses of state and large urban school district data indicated that across 39 states, the overall prevalence of having felt sad or hopeless ranged from 24.8% to 40.2% across state surveys (median: 30.4%) (Supplementary Table 43). Across 21 large urban school districts, the prevalence ranged from 26.1% to 35.5% (median: 31.4%).

#### Seriously Considered Attempting Suicide

Nationwide, 17.2% of students had seriously considered attempting suicide during the 12 months before the survey (Supplementary Table 44). The prevalence of having seriously considered attempting suicide was higher among female (22.1%) than male (11.9%) students; higher among white female (21.2%), black female (22.4%), and Hispanic female (22.2%) than white male (13.0%), black male (6.6%), and Hispanic male (10.8%) students, respectively; and higher among 9th-grade female (22.1%), 10th-grade female (23.4%), 11th-grade female (23.1%), and 12th-grade female (19.5%) than 9th-grade male (10.3%), 10th-grade male (10.9%), 11th-grade male (11.7%), and 12th-grade male (15.1%) students, respectively. The prevalence of having seriously considered attempting suicide was higher among white (17.3%) than black (14.7%) students and higher among white male (13.0%) and Hispanic male (10.8%) than black male (6.6%) students. The prevalence of having seriously considered attempting suicide was higher among 10th-grade female (23.4%) than 12th-grade female (19.5%) students and higher among 12th-grade male (15.1%) than 9th-grade male (10.3%), 10th-grade male (10.9%), and 11th-grade male (11.7%) students.

Analyses based on the question ascertaining sexual identity indicated that nationwide, 13.3% of heterosexual students; 47.7% of gay, lesbian, and bisexual students; and 31.8% of not sure students had seriously considered attempting suicide (Supplementary Table 44). The prevalence of having seriously considered attempting suicide was higher among gay, lesbian, and bisexual (47.7%) and not sure (31.8%) than heterosexual (13.3%) students and higher among gay, lesbian, and bisexual (47.7%) than not sure (31.8%) students. Among female students, the prevalence was higher among lesbian and bisexual (51.0%) and not sure (35.9%) than heterosexual (16.9%) students and higher among lesbian and bisexual (51.0%) than not sure (35.9%) students. Among male students, the prevalence was higher among gay and bisexual (37.0%) and not sure (23.9%) than heterosexual (10.2%) students and higher among gay and bisexual (37.0%) than not sure (23.9%) students. The prevalence also was higher among heterosexual female (16.9%) than heterosexual male (10.2%) students, higher among lesbian and bisexual female (51.0%) than gay and bisexual male (37.0%) students, and higher among not sure female (35.9%) than not sure male (23.9%) students.

Analyses based on the question ascertaining the sex of sexual contacts indicated that nationwide, 19.0% of students who had sexual contact with only the opposite sex, 45.1% of students who had sexual contact with only the same sex or with both sexes, and 12.3% of students who had no sexual contact had seriously considered attempting suicide (Supplementary Table 44). The prevalence of having seriously considered attempting suicide was higher among students who had sexual contact with only the opposite sex (19.0%) and students who had sexual contact with only the same sex or with both sexes (45.1%) than students who had no sexual contact (12.3%) and higher among students who had sexual contact with only the same sex or with both sexes (45.1%) than students who had sexual contact with only the opposite sex (19.0%). Among female students, the prevalence was higher among those who had sexual contact with only males (25.8%) and those who had sexual contact with only females or with both sexes (48.7%) than those who had no sexual contact (15.9%) and higher among those who had sexual contact with only females or with both sexes (48.7%) than those who had sexual contact with only males (25.8%). Among male students, the prevalence was higher among those who had sexual contact with only females (13.5%) and those who had sexual contact with only males or with both sexes (34.6%) than those who had no sexual contact (8.5%) and higher among those who had sexual contact with only males or with both sexes (34.6%) than those who had sexual contact with only females (13.5%). The prevalence also was higher among female students who had sexual contact with only males (25.8%) than male students who had sexual contact with only females (13.5%), higher among female students who had sexual contact with only females or with both sexes (48.7%) than male students who had sexual contact with only males or with both sexes (34.6%), and higher among female students who had no sexual contact (15.9%) than male students who had no sexual contact (8.5%).

Trend analyses indicated that during 1991–2017, a significant linear decrease (29.0%–17.2%) occurred in the overall prevalence of having seriously considered attempting suicide. A significant quadratic trend also was identified. The prevalence of having seriously considered attempting suicide decreased during 1991–2007 (29.0%–14.5%) and then increased during 2007–2017 (14.5%–17.2%). The prevalence of having seriously considered attempting suicide did not change significantly from 2015 (17.7%) to 2017 (17.2%).

Analyses of state and large urban school district data indicated that across 38 states, the overall prevalence of having seriously considered attempting suicide ranged from 12.4% to 23.2% across state surveys (median: 17.0%) (Supplementary Table 45). Across 21 large urban school districts, the prevalence ranged from 11.9% to 20.5% (median: 15.7%).

#### Made a Suicide Plan

During the 12 months before the survey, 13.6% of students nationwide had made a plan about how they would attempt suicide (Supplementary Table 46). The prevalence of having made a suicide plan was higher among female (17.1%) than male (9.7%) students; higher among white female (15.3%), black female (18.9%), and Hispanic female (17.2%) than white male (9.6%), black male (6.5%), and Hispanic male (9.9%) students, respectively; and higher among 9th-grade female (16.3%), 10th-grade female (19.0%), and 11th-grade female (18.5%) than 9th-grade male (8.8%), 10th-grade male (9.0%), and 11th-grade male (9.7%) students, respectively. The prevalence of having made a suicide plan was higher among white male (9.6%) and Hispanic male (9.9%) than black male (6.5%) students. The prevalence of having made a suicide plan was higher among 10th-grade female (19.0%) and 11th-grade female (18.5%) than 12th-grade female (14.2%) students.

Analyses based on the question ascertaining sexual identity indicated that nationwide, 10.4% of heterosexual students; 38.0% of gay, lesbian, and bisexual students; and 25.6% of not sure students had made a plan about how they would attempt suicide (Supplementary Table 46). The prevalence of having made a suicide plan was higher among gay, lesbian, and bisexual (38.0%) and not sure (25.6%) than heterosexual (10.4%) students and higher among gay, lesbian, and bisexual (38.0%) than not sure (25.6%) students. Among female students, the prevalence was higher among lesbian and bisexual (40.8%) and not sure (26.8%) than heterosexual (12.8%) students and higher among lesbian and bisexual (40.8%) than not sure (26.8%) students. Among male students, the prevalence was higher among gay and bisexual (28.7%) and not sure (21.9%) than heterosexual (8.2%) students. The prevalence also was higher among heterosexual female (12.8%) than heterosexual male (8.2%) students and higher among lesbian and bisexual female (40.8%) than gay and bisexual male (28.7%) students.

Analyses based on the question ascertaining the sex of sexual contacts indicated that nationwide, 14.4% of students who had sexual contact with only the opposite sex, 41.2% of students who had sexual contact with only the same sex or with both sexes, and 9.1% of students who had no sexual contact had made a plan about how they would attempt suicide (Supplementary Table 46). The prevalence of having made a suicide plan was higher among students who had sexual contact with only the opposite sex (14.4%) and students who had sexual contact with only the same sex or with both sexes (41.2%) than students who had no sexual contact (9.1%) and higher among students who had sexual contact with only the same sex or with both sexes (41.2%) than students who had sexual contact with only the opposite sex (14.4%). Among female students, the prevalence was higher among those who had sexual contact with only males (19.4%) and those who had sexual contact with only females or with both sexes (42.3%) than those who had no sexual contact (11.3%) and higher among those who had sexual contact with only females or with both sexes (42.3%) than those who had sexual contact with only males (19.4%). Among male students, the prevalence was higher among those who had sexual contact with only females (10.2%) and those who had sexual contact with only males or with both sexes (38.0%) than those who had no sexual contact (6.7%) and higher among those who had sexual contact with only males or with both sexes (38.0%) than those who had sexual contact with only females (10.2%). The prevalence also was higher among female students who had sexual contact with only males (19.4%) than male students who had sexual contact with only females (10.2%) and higher among female students who had no sexual contact (11.3%) than male students who had no sexual contact (6.7%).

Trend analyses indicated that during 1991–2017, a significant linear decrease (18.6%–13.6%) occurred in the overall prevalence of having made a suicide plan. A significant quadratic trend also was identified. The prevalence of having made a suicide plan decreased during 1991–2009 (18.6%–10.9%) and then increased during 2009–2017 (10.9%–13.6%). The prevalence of having made a suicide plan did not change significantly from 2015 (14.6%) to 2017 (13.6%).

Analyses of state and large urban school district data indicated that across 36 states, the overall prevalence of having made a suicide plan ranged from 10.7% to 26.1% across state surveys (median: 14.2%) (Supplementary Table 47). Across 18 large urban school districts, the prevalence ranged from 10.1% to 18.4% (median: 13.2%).

#### Attempted Suicide

Nationwide, 7.4% of students had actually attempted suicide one or more times during the 12 months before the survey (Supplementary Table 48). The prevalence of having attempted suicide was higher among female (9.3%) than male (5.1%) students; higher among white female (7.3%), black female (12.5%), and Hispanic female (10.5%) than white male (4.6%), black male (6.7%), and Hispanic male (5.8%) students, respectively; and higher among 9th-grade female (11.3%) and 10th-grade female (11.7%) than 9th-grade male (5.0%) and 10th-grade male (5.2%) students, respectively. The prevalence of having attempted suicide was higher among black (9.8%) than white (6.1%) students and higher among black female (12.5%) than white female (7.3%) students. The prevalence of having attempted suicide was higher among 9th-grade (8.3%) and 10th-grade (8.6%) than 11th-grade (6.1%) and 12th-grade (5.8%) students and higher among 9th-grade female (11.3%) and 10th-grade female (11.7%) than 11th-grade female (7.3%) and 12th-grade female (6.2%) students.

Analyses based on the question ascertaining sexual identity indicated that nationwide, 5.4% of heterosexual students; 23.0% of gay, lesbian, and bisexual students; and 14.3% of not sure students had attempted suicide (Supplementary Table 48). The prevalence of having attempted suicide was higher among gay, lesbian, and bisexual (23.0%) and not sure (14.3%) than heterosexual (5.4%) students and higher among gay, lesbian, and bisexual (23.0%) than not sure (14.3%) students. Among female students, the prevalence was higher among lesbian and bisexual (23.7%) and not sure (12.9%) than heterosexual (7.0%) students and higher among lesbian and bisexual (23.7%) than not sure (12.9%) students. Among male students, the prevalence was higher among gay and bisexual (18.3%) and not sure (13.8%) than heterosexual (4.1%) students. The prevalence also was higher among heterosexual female (7.0%) than heterosexual male (4.1%) students.

Analyses based on the question ascertaining the sex of sexual contacts indicated that nationwide, 8.1% of students who had sexual contact with only the opposite sex, 23.8% of students who had sexual contact with only the same sex or with both sexes, and 4.2% of students who had no sexual contact had attempted suicide (Supplementary Table 48). The prevalence of having attempted suicide was higher among students who had sexual contact with only the opposite sex (8.1%) and students who had sexual contact with only the same sex or with both sexes (23.8%) than students who had no sexual contact (4.2%) and higher among students who had sexual contact with only the same sex or with both sexes (23.8%) than students who had sexual contact with only the opposite sex (8.1%). Among female students, the prevalence was higher among those who had sexual contact with only males (10.9%) and those who had sexual contact with only females or with both sexes (24.1%) than those who had no sexual contact (5.8%) and higher among those who had sexual contact with only females or with both sexes (24.1%) than those who had sexual contact with only males (10.9%). Among male students, the prevalence was higher among those who had sexual contact with only females (5.8%) and those who had sexual contact with only males or with both sexes (22.6%) than those who had no sexual contact (2.5%) and higher among those who had sexual contact with only males or with both sexes (22.6%) than those who had sexual contact with only females (5.8%). The prevalence also was higher among female students who had sexual contact with only males (10.9%) than male students who had sexual contact with only females (5.8%) and higher among female students who had no sexual contact (5.8%) than male students who had no sexual contact (2.5%).

Trend analyses indicated that during 1991–2017, a significant linear decrease (7.3%–7.4%) occurred in the overall prevalence of having attempted suicide.** A significant quadratic trend was not identified. The prevalence of having attempted suicide did not change significantly from 2015 (8.6%) to 2017 (7.4%).

Analyses of state and large urban school district data indicated that across 38 states, the overall prevalence of having attempted suicide ranged from 5.4% to 16.8% across state surveys (median: 9.3%) (Supplementary Table 49). Across 21 large urban school districts, the prevalence ranged from 5.6% to 19.5% (median: 11.0%).

#### Made a Suicide Attempt Resulting in an Injury, Poisoning, or Overdose that Had to be Treated by a Doctor or Nurse

During the 12 months before the survey, 2.4% of students nationwide had made a suicide attempt resulting in an injury, poisoning, or overdose that had to be treated by a doctor or nurse (Supplementary Table 50). The prevalence of having made a suicide attempt resulting in an injury, poisoning, or overdose that had to be treated by a doctor or nurse was higher among female (3.1%) than male (1.5%) students; higher among white female (2.3%) and Hispanic female (3.8%) than white male (1.3%) and Hispanic male (1.7%) students, respectively; and higher among 9th-grade female (3.8%) and 12th-grade female (2.7%) than 9th-grade male (1.2%) and 12th-grade male (1.1%) students, respectively. The prevalence of having made a suicide attempt resulting in an injury, poisoning, or overdose that had to be treated by a doctor or nurse was higher among black (3.4%) than white (1.9%) students.

Analyses based on the question ascertaining sexual identity indicated that nationwide, 1.7% of heterosexual students; 7.5% of gay, lesbian, and bisexual students; and 5.6% of not sure students had made a suicide attempt resulting in an injury, poisoning, or overdose that had to be treated by a doctor or nurse (Supplementary Table 50). The prevalence of having made a suicide attempt resulting in an injury, poisoning, or overdose that had to be treated by a doctor or nurse was higher among gay, lesbian, and bisexual (7.5%) and not sure (5.6%) than heterosexual (1.7%) students. Among female students, the prevalence was higher among lesbian and bisexual (8.2%) than heterosexual (2.2%) and not sure (4.4%) students. The prevalence also was higher among heterosexual female (2.2%) than heterosexual male (1.3%) students and higher among lesbian and bisexual female (8.2%) than gay and bisexual male (3.8%) students.

Analyses based on the question ascertaining the sex of sexual contacts indicated that nationwide, 2.7% of students who had sexual contact with only the opposite sex, 7.8% of students who had sexual contact with only the same sex or with both sexes, and 1.2% of students who had no sexual contact had made a suicide attempt resulting in an injury, poisoning, or overdose that had to be treated by a doctor or nurse (Supplementary Table 50). The prevalence of having made a suicide attempt resulting in an injury, poisoning, or overdose that had to be treated by a doctor or nurse was higher among students who had sexual contact with only the opposite sex (2.7%) and students who had sexual contact with only the same sex or with both sexes (7.8%) than students who had no sexual contact (1.2%) and higher among students who had sexual contact with only the same sex or with both sexes (7.8%) than students who had sexual contact with only the opposite sex (2.7%). Among female students, the prevalence was higher among those who had sexual contact with only males (3.9%) and those who had sexual contact with only females or with both sexes (8.2%) than those who had no sexual contact (1.7%) and higher among those who had sexual contact with only females or with both sexes (8.2%) than those who had sexual contact with only males (3.9%). Among male students, the prevalence was higher among those who had sexual contact with only females (1.6%) and those who had sexual contact with only males or with both sexes (6.5%) than those who had no sexual contact (0.6%). The prevalence also was higher among female students who had sexual contact with only males (3.9%) than male students who had sexual contact with only females (1.6%) and higher among female students who had no sexual contact (1.7%) than male students who had no sexual contact (0.6%).

Trend analyses did not identify a significant linear trend in the overall prevalence of having made a suicide attempt resulting in an injury, poisoning, or overdose that had to be treated by a doctor or nurse during 1991–2017 (1.7%–2.4%). A significant quadratic trend also was not identified. The prevalence of having made a suicide attempt resulting in an injury, poisoning, or overdose that had to be treated by a doctor or nurse did not change significantly from 2015 (2.8%) to 2017 (2.4%).

Analyses of state and large urban school district data indicated that across 33 states, the overall prevalence of having made a suicide attempt resulting in an injury, poisoning, or overdose that had to be treated by a doctor or nurse ranged from 1.9% to 7.6% across state surveys (median: 3.1%) (Supplementary Table 51). Across 19 large urban school districts, the prevalence ranged from 1.5% to 7.5% (median: 3.6%).

### Tobacco Use

#### Ever Tried Cigarette Smoking

Nationwide, 28.9% of students had ever tried cigarette smoking (even one or two puffs) (Supplementary Table 52). The prevalence of having ever tried cigarette smoking was higher among male (30.7%) than female (27.3%) students. The prevalence of having ever tried cigarette smoking was higher among white (31.0%) and Hispanic (29.7%) than black (21.1%) students, higher among white female (29.1%) and Hispanic female (27.5%) than black female (21.2%) students, and higher among white male (33.0%) and Hispanic male (31.8%) than black male (20.8%) students. The prevalence of having ever tried cigarette smoking was higher among 10th-grade (26.1%), 11th-grade (33.1%), and 12th-grade (37.1%) than 9th-grade (20.9%) students; higher among 11th-grade (33.1%) and 12th-grade (37.1%) than 10th-grade (26.1%) students; higher among 12th-grade (37.1%) than 11th-grade (33.1%) students, higher among 10th-grade female (24.6%), 11th-grade female (30.5%), and 12th-grade female (34.8%) than 9th-grade female (20.3%) students; higher among 11th-grade female (30.5%) and 12th-grade female (34.8%) than 10th-grade female (24.6%) students; higher among 10th-grade male (27.8%), 11th-grade male (35.8%), and 12th-grade male (39.5%) than 9th-grade male (21.4%) students; and higher among 11th-grade male (35.8%) and 12th-grade male (39.5%) than 10th-grade male (27.8%) students.

Analyses based on the question ascertaining sexual identity indicated that nationwide, 28.2% of heterosexual students; 41.8% of gay, lesbian, and bisexual students; and 27.5% of not sure students had ever tried cigarette smoking (Supplementary Table 52). The prevalence of having ever tried cigarette smoking was higher among gay, lesbian, and bisexual (41.8%) than heterosexual (28.2%) and not sure (27.5%) students. Among female students, the prevalence was higher among lesbian and bisexual (42.1%) than heterosexual (25.7%) and not sure (25.4%) students. Among male students, the prevalence was higher among gay and bisexual (40.2%) than heterosexual (30.5%) and not sure (28.6%) students. The prevalence also was higher among heterosexual male (30.5%) than heterosexual female (25.7%) students.

Analyses based on the question ascertaining the sex of sexual contacts indicated that nationwide, 43.3% of students who had sexual contact with only the opposite sex, 57.2% of students who had sexual contact with only the same sex or with both sexes, and 13.0% of students who had no sexual contact had ever tried cigarette smoking (Supplementary Table 52). The prevalence of having ever tried cigarette smoking was higher among students who had sexual contact with only the opposite sex (43.3%) and students who had sexual contact with only the same sex or with both sexes (57.2%) than students who had no sexual contact (13.0%) and higher among students who had sexual contact with only the same sex or with both sexes (57.2%) than students who had sexual contact with only the opposite sex (43.3%). Among female students, the prevalence was higher among those who had sexual contact with only males (40.0%) and those who had sexual contact with only females or with both sexes (57.4%) than those who had no sexual contact (12.9%) and higher among those who had sexual contact with only females or with both sexes (57.4%) than those who had sexual contact with only males (40.0%). Among male students, the prevalence was higher among those who had sexual contact with only females (46.1%) and those who had sexual contact with only males or with both sexes (56.8%) than those who had no sexual contact (13.2%) and higher among those who had sexual contact with only males or with both sexes (56.8%) than those who had sexual contact with only females (46.1%). The prevalence also was higher among male students who had sexual contact with only females (46.1%) than female students who had sexual contact with only males (40.0%).

Trend analyses indicated that during 1991–2017, a significant linear decrease (70.1%–28.9%) occurred in the overall prevalence of having ever tried cigarette smoking. A significant quadratic trend also was identified. The prevalence of having ever tried cigarette smoking did not change significantly during 1991–1999 (70.1%–70.4%) and then decreased during 1999–2017 (70.4%–28.9%). The prevalence of having ever tried cigarette smoking did not change significantly from 2015 (32.3%) to 2017 (28.9%).

Analyses of state and large urban school district data indicated that across 30 states, the overall prevalence of having ever tried cigarette smoking ranged from 16.4% to 40.5% across state surveys (median: 28.3%) (Supplementary Table 53). Across 16 large urban school districts, the prevalence ranged from 15.0% to 27.3% (median: 18.6%).

#### Tried Cigarette Smoking Before Age 13 Years

Nationwide, 9.5% of students had first tried cigarette smoking (even one or two puffs) before age 13 years (Supplementary Table 54). The prevalence of having first tried cigarette smoking before age 13 years was higher among male (10.9%) than female (8.0%) students; higher among white male (10.0%) and Hispanic male (13.0%) than white female (7.7%) and Hispanic female (7.1%) students, respectively; and higher among 11th-grade male (10.7%) and 12th-grade male (11.6%) than 11th-grade female (8.3%) and 12th-grade female (7.5%) students, respectively. The prevalence of having smoked a whole cigarette before age 13 years was higher among black female (10.9%) than Hispanic female (7.1%) students.

Analyses based on the question ascertaining sexual identity indicated that nationwide, 8.8% of heterosexual students; 14.2% of gay, lesbian, and bisexual students; and 14.8% of not sure students had first tried cigarette smoking before age 13 years (Supplementary Table 54). The prevalence of having first tried cigarette smoking before age 13 years was higher among gay, lesbian, and bisexual (14.2%) and not sure (14.8%) than heterosexual (8.8%) students. Among female students, the prevalence was higher among lesbian and bisexual (13.2%) than heterosexual (7.0%) students. Among male students, the prevalence was higher among gay and bisexual (15.9%) and not sure (16.7%) than heterosexual (10.4%) students. The prevalence also was higher among heterosexual male (10.4%) than heterosexual female (7.0%) students.

Analyses based on the question ascertaining the sex of sexual contacts indicated that nationwide, 13.5% of students who had sexual contact with only the opposite sex, 23.9% of students who had sexual contact with only the same sex or with both sexes, and 3.9% of students who had no sexual contact had first tried cigarette smoking (even one or two puffs) before age 13 years (Supplementary Table 54). The prevalence of was higher among students who had sexual contact with only the opposite sex (13.5%) and students who had sexual contact with only the same sex or with both sexes (23.9%) than students who had no sexual contact (3.9%) and higher among students who had sexual contact with only the same sex or with both sexes (23.9%) than students who had sexual contact with only the opposite sex (13.5%). Among female students, the prevalence was higher among those who had sexual contact with only males (10.3%) and those who had sexual contact with only females or with both sexes (23.1%) than those who had no sexual contact (3.5%) and higher among those who had sexual contact with only females or with both sexes (23.1%) than those who had sexual contact with only males (10.3%). Among male students, the prevalence was higher among those who had sexual contact with only females (16.1%) and those who had sexual contact with only males or with both sexes (26.2%) than those who had no sexual contact (4.3%) and higher among those who had sexual contact with only males or with both sexes (26.2%) than those who had sexual contact with only females (16.1%). The prevalence also was higher among male students who had sexual contact with only females (16.1%) than female students who had sexual contact with only males (10.3%).

The question measuring the prevalence of having first tried cigarette smoking (even one or two puffs) before age 13 years was used for the first time in the 2017 national YRBS. As a result, long-term temporal trends and 2-year temporal changes are not available for this variable.

Analyses of state and large urban school district data indicated that across 32 states, the overall prevalence of having first tried cigarette smoking before age 13 years ranged from 5.7% to 16.7% across state surveys (median: 9.9%) (Supplementary Table 55). Across 18 large urban school districts, the prevalence ranged from 5.9% to 12.6% (median: 9.1%).

#### Current Cigarette Use

Nationwide, 8.8% of students had smoked cigarettes on at least 1 day during the 30 days before the survey (i.e., current cigarette use) (Supplementary Table 56). The prevalence of current cigarette use was higher among male (9.8%) than female (7.8%) students; higher among white male (12.3%) and black male (5.7%) than white female (9.9%) and black female (2.8%) students, respectively; and higher among 12th-grade male (15.7%) than 12th-grade female (11.1%) students. The prevalence of current cigarette use was higher among white (11.1%) and Hispanic (7.0%) than black (4.4%) students, higher among white (11.1%) than Hispanic (7.0%) students, higher among white female (9.9%) and Hispanic female (6.6%) than black female (2.8%) students, higher among white female (9.9%) than Hispanic female (6.6%) students, higher among white male (12.3%) than black male (5.7%) and Hispanic male (7.4%) students. The prevalence of current cigarette use was higher among 10th-grade (7.6%), 11th-grade (9.5%), and 12th-grade (13.4%) than 9th-grade (5.2%) students; higher among 11th-grade (9.5%) and 12th-grade (13.4%) than 10th-grade (7.6%) students; higher among 12th-grade (13.4%) than 11th-grade (9.5%) students; higher among 11th-grade female (8.6%) and 12th-grade female (11.1%) than 9th-grade female (4.9%) students; higher among 12th-grade female (11.1%) than 10th-grade female (6.8%) students; higher among 10th-grade male (8.4%), 11th-grade male (10.2%), and 12th-grade male (15.7%) than 9th-grade male (5.6%) students; and higher among 12th-grade male (15.7%) than 10th-grade male (8.4%) and 11th-grade male (10.2%) students.

Analyses based on the question ascertaining sexual identity indicated that nationwide, the prevalence of current cigarette use was 8.1% among heterosexual students; 16.2% among gay, lesbian, and bisexual students; and 10.1% among not sure students (Supplementary Table 56). The prevalence of current cigarette use was higher among gay, lesbian, and bisexual (16.2%) than heterosexual (8.1%) and not sure (10.1%) students. Among female students, the prevalence was higher among lesbian and bisexual (15.4%) than heterosexual (6.6%) and not sure (8.6%) students. Among male students, the prevalence was higher among gay and bisexual (17.1%) than heterosexual (9.4%) and not sure (9.7%) students. The prevalence also was higher among heterosexual male (9.4%) than heterosexual female (6.6%) students.

Analyses based on the question ascertaining the sex of sexual contacts indicated that nationwide, the prevalence of current cigarette use was 14.2% among students who had sexual contact with only the opposite sex, 24.5% among students who had sexual contact with only the same sex or with both sexes, and 1.9% among students who had no sexual contact (Supplementary Table 56). The prevalence of current cigarette use was higher among students who had sexual contact with only the opposite sex (14.2%) and students who had sexual contact with only the same sex or with both sexes (24.5%) than students who had no sexual contact (1.9%) and higher among students who had sexual contact with only the same sex or with both sexes (24.5%) than students who had sexual contact with only the opposite sex (14.2%). Among female students, the prevalence was higher among those who had sexual contact with only males (12.1%) and those who had sexual contact with only females or with both sexes (24.9%) than those who had no sexual contact (1.5%) and higher among those who had sexual contact with only females or with both sexes (24.9%) than those who had sexual contact with only males (12.1%). Among male students, the prevalence was higher among those who had sexual contact with only females (15.9%) and those who had sexual contact with only males or with both sexes (23.4%) than those who had no sexual contact (2.3%). The prevalence also was higher among male students who had sexual contact with only females (15.9%) than female students who had sexual contact with only males (12.1%).

Trend analyses indicated that during 1991–2017, a significant linear decrease (27.5%–8.8%) occurred in the overall prevalence of current cigarette use. A significant quadratic trend also was identified. The prevalence of current cigarette use increased during 1991–1997 (27.5%–36.4%) and then decreased during 1997–2017 (36.4%–8.8%). The prevalence of current cigarette use did not change significantly from 2015 (10.8%) to 2017 (8.8%).

Analyses of state and large urban school district data indicated that across 39 states, the overall prevalence of current cigarette use ranged from 3.8% to 14.4% across state surveys (median: 8.2%) (Supplementary Table 57). Across 19 large urban school districts, the prevalence ranged from 2.7% to 6.7% (median: 4.2%).

#### Current Frequent Cigarette Use

Nationwide, 2.6% of students had smoked cigarettes on 20 or more days during the 30 days before the survey (i.e., current frequent cigarette use) (Supplementary Table 58). The prevalence of current frequent cigarette use was higher among Hispanic male (2.2%) than Hispanic female (1.1%) students. The prevalence of current frequent cigarette use was higher among white (3.6%) than black (1.1%) and Hispanic (1.7%) students, higher among white female (3.7%) than black female (0.9%) and Hispanic female (1.1%) students, and higher among white male (3.4%) than black male (1.2%) and Hispanic male (2.2%) students. The prevalence of current frequent cigarette use was higher among 12th-grade (4.7%) than 9th-grade (1.3%), 10th-grade (1.8%), and 11th-grade (2.8%) students; higher among 11th-grade (2.8%) than 9th-grade (1.3%) students; higher among 12th-grade female (4.8%) than 9th-grade female (1.1%), 10th-grade female (1.5%), and 11th-grade female (2.9%) students; higher among 11th-grade female (2.9%) than 9th-grade female (1.1%) students; and higher among 12th-grade male (4.5%) than 9th-grade male (1.5%), 10th-grade male (2.1%), and 11th-grade male (2.7%) students.

Analyses based on the question ascertaining sexual identity indicated that nationwide, the prevalence of current frequent cigarette use was 2.3% among heterosexual students; 5.4% among gay, lesbian, and bisexual students; and 4.0% among not sure students (Supplementary Table 58). The prevalence of current frequent cigarette use was higher among gay, lesbian, and bisexual (5.4%) than heterosexual (2.3%) students. Among female students, the prevalence was higher among lesbian and bisexual (5.3%) than heterosexual (2.1%) students. Among male students, the prevalence was higher among gay and bisexual (5.9%) than heterosexual (2.4%) students.

Analyses based on the question ascertaining the sex of sexual contacts indicated that nationwide, the prevalence of current frequent cigarette use was 4.2% among students who had sexual contact with only the opposite sex, 10.3% among students who had sexual contact with only the same sex or with both sexes, and 0.2% among students who had no sexual contact (Supplementary Table 58). The prevalence of current frequent cigarette use was higher among students who had sexual contact with only the opposite sex (4.2%) and students who had sexual contact with only the same sex or with both sexes (10.3%) than students who had no sexual contact (0.2%) and higher among students who had sexual contact with only the same sex or with both sexes (10.3%) than students who had sexual contact with only the opposite sex (4.2%). Among female students, the prevalence was higher among those who had sexual contact with only males (3.7%) and those who had sexual contact with only females or with both sexes (11.0%) than those who had no sexual contact (0.3%) and higher among those who had sexual contact with only females or with both sexes (11.0%) than those who had sexual contact with only males (3.7%). Among male students, the prevalence was higher among those who had sexual contact with only females (4.7%) and those who had sexual contact with only males or with both sexes (8.0%) than those who had no sexual contact (0.1%).

Trend analyses indicated that during 1991–2017, a significant linear decrease (12.7%–2.6%) occurred in the overall prevalence of current frequent cigarette use. A significant quadratic trend also was identified. The prevalence of current frequent cigarette use increased during 1991–1999 (12.7%–16.8%) and then decreased during 1999–2017 (16.8%–2.6%). The prevalence of current frequent cigarette use did not change significantly from 2015 (3.4%) to 2017 (2.6%).

Analyses of state and large urban school district data indicated that across 39 states, the overall prevalence of current frequent cigarette use ranged from 0.4% to 5.5% across state surveys (median: 2.2%) (Supplementary Table 59). Across 19 large urban school districts, the prevalence ranged from 0.1% to 1.4% (median: 0.8%).

#### Current Daily Cigarette Use

Nationwide, 2.0% of students had smoked cigarettes on all 30 days during the 30 days before the survey (i.e., current daily cigarette use) (Supplementary Table 60). The prevalence of current daily cigarette use was higher among white (2.6%) than black (1.1%) and Hispanic (1.3%) students and higher among white female (2.9%) than black female (0.9%) and Hispanic female (0.8%) students. The prevalence of having currently smoked cigarettes daily was higher among 11th-grade (2.2%) and 12th-grade (3.5%) than 9th-grade (0.9%) students, higher among 12th-grade (3.5%) than 10th-grade (1.4%) students, higher among 11th-grade female (2.4%) and 12th-grade female (3.7%) than 9th-grade female (0.9%) students, higher among 12th-grade female (3.7%) than 10th-grade female (1.1%) students, and higher among 11th-grade male (2.1%) and 12th-grade male (3.1%) than 9th-grade male (1.0%) students.

Analyses based on the question ascertaining sexual identity indicated that nationwide, the prevalence of current daily cigarette use was 1.7% among heterosexual students; 3.9% among gay, lesbian, and bisexual students; and 3.4% among not sure students (Supplementary Table 60). The prevalence of current daily cigarette use was higher among gay, lesbian, and bisexual (3.9%) than heterosexual (1.7%) students.

Analyses based on the question ascertaining the sex of sexual contacts indicated that nationwide, the prevalence of current daily cigarette use was 3.2% among students who had sexual contact with only the opposite sex, 8.3% among students who had sexual contact with only the same sex or with both sexes, and 0.2% among students who had no sexual contact (Supplementary Table 60). The prevalence of current daily cigarette use was higher among students who had sexual contact with only the opposite sex (3.2%) and students who had sexual contact with only the same sex or with both sexes (8.3%) than students who had no sexual contact (0.2%) and higher among students who had sexual contact with only the same sex or with both sexes (8.3%) than students who had sexual contact with only the opposite sex (3.2%). Among female students, the prevalence was higher among those who had sexual contact with only males (2.8%) and those who had sexual contact with only females or with both sexes (9.0%) than those who had no sexual contact (0.3%) and higher among those who had sexual contact with only females or with both sexes (9.0%) than those who had sexual contact with only males (2.8%). Among male students, the prevalence was higher among those who had sexual contact with only females (3.4%) and those who had sexual contact with only males or with both sexes (6.3%) than those who had no sexual contact (0.1%).

Trend analyses indicated that during 1991–2017, a significant linear decrease (9.8%–2.0%) occurred in the overall prevalence of current daily cigarette use. A significant quadratic trend also was identified. The prevalence of current daily cigarette use increased during 1991–1999 (9.8%–12.8%) and then decreased during 1999–2017 (12.8%–2.0%). The prevalence of current daily cigarette use did not change significantly from 2015 (2.3%) to 2017 (2.0%).

Analyses of state and large urban school district data indicated that across 39 states, the overall prevalence of current daily cigarette use ranged from 0.3% to 4.5% across state surveys (median: 1.6%) (Supplementary Table 61). Across 19 large urban school districts, the prevalence ranged from 0.1% to 0.8% (median: 0.6%).

#### Smoked More than 10 Cigarettes per Day

Among the 8.8% of students nationwide who currently smoked cigarettes, 9.7% of students had smoked more than 10 cigarettes per day on the days they smoked during the 30 days before the survey (Supplementary Table 62). The prevalence of having smoked more than 10 cigarettes per day was higher among male (11.7%) than female (6.5%) students and higher among white male (10.4%) than white female (5.6%) students. The prevalence of having smoked more than 10 cigarettes per day was higher among 12th-grade (11.6%) than 11th-grade (5.1%) students.

Analyses based on the question ascertaining sexual identity indicated that nationwide, among the students who currently smoked cigarettes, 8.1% of heterosexual students; 5.7% of gay, lesbian, and bisexual students; and 39.6% of not sure students had smoked more than 10 cigarettes per day (Supplementary Table 62). The prevalence of having smoked more than 10 cigarettes per day was higher among not sure (39.6%) than heterosexual (8.1%) and gay, lesbian, and bisexual (5.7%) students.

Analyses based on the question ascertaining the sex of sexual contacts indicated that nationwide, among the students who currently smoked cigarettes, 8.5% of students who had sexual contact with only the opposite sex, 14.8% of students who had sexual contact with only the same sex or with both sexes, and 1.5% of students who had no sexual contact had smoked more than 10 cigarettes per day (Supplementary Table 62). The prevalence of having smoked more than 10 cigarettes per day was higher among students who had sexual contact with only the opposite sex (8.5%) and students who had sexual contact with only the same sex or with both sexes (14.8%) than students who had no sexual contact (1.5%). Among male students, the prevalence was higher among those who had sexual contact with only females (10.5%) and those who had sexual contact with only males or with both sexes (32.3%) than those who had no sexual contact (1.2%). The prevalence also was higher among male students who had sexual contact with only females (10.5%) than female students who had sexual contact with only males (5.2%) and higher among male students who had sexual contact with only males or with both sexes (32.3%) than female students who had sexual contact with only females or with both sexes (9.4%).

Trend analyses indicated that during 1991–2017, a significant linear decrease (18.0%–9.7%) occurred in the overall prevalence of having smoked more than 10 cigarettes per day, among the students who currently smoked cigarettes. A significant quadratic trend was not identified. The prevalence of having smoked more than 10 cigarettes per day did not change significantly from 2015 (7.9%) to 2017 (9.7%).

Analyses of state and large urban school district data indicated that across 28 states, the overall prevalence of having smoked more than 10 cigarettes per day, among the students who currently smoked cigarettes, ranged from 2.3% to 18.1% across state surveys (median: 8.0%) (Supplementary Table 63). Across seven large urban school districts, the prevalence ranged from 3.6% to 12.9% (median: 7.9%).

#### Ever Used an Electronic Vapor Product

Nationwide, 42.2% of students had ever used an electronic vapor product (including e-cigarettes, e-cigars, e-pipes, vape pipes, vaping pens, e-hookahs, and hookah pens) (Supplementary Table 64). The prevalence of having ever used an electronic vapor product was higher among male (44.9%) than female (39.7%) students, higher among Hispanic male (50.5%) than Hispanic female (46.8%) students, and higher among 12th-grade male (52.4%) than 12th-grade female (45.0%) students. The prevalence of having ever used an electronic vapor product was higher among white (41.8%) and Hispanic (48.7%) than black (36.2%) students, higher among Hispanic (48.7%) than white (41.8%) students, higher among Hispanic female (46.8%) than white female (39.1%) and black female (35.5%) students, higher among white male (44.9%) and Hispanic male (50.5%) than black male (36.7%) students, and higher among Hispanic male (50.5%) than white male (44.9%) students. The prevalence of having ever used an electronic vapor product was higher among 10th-grade (41.0%), 11th-grade (48.0%), and 12th-grade (48.6%) than 9th-grade (32.7%) students; higher among 11th-grade (48.0%) and 12th-grade (48.6%) than 10th-grade (41.0%) students; higher among 10th-grade female (38.7%), 11th-grade female (45.6%), and 12th-grade female (45.0%) than 9th-grade female (30.8%) students; higher among 11th-grade female (45.6%) and 12th-grade female (45.0%) than 10th-grade female (38.7%) students; and higher among 10th-grade male (43.6%), 11th-grade male (50.5%), and 12th-grade male (52.4%) than 9th-grade male (34.6%) students, and higher among 11th-grade male (50.5%) and 12th-grade male (52.4%) than 10th-grade male (43.6%) students.

Analyses based on the question ascertaining sexual identity indicated that nationwide, 42.8% of heterosexual students; 50.5% of gay, lesbian, and bisexual students; and 37.3% of not sure students had ever used an electronic vapor product (Supplementary Table 64). The prevalence of having ever used an electronic vapor product was higher among heterosexual (42.8%) and gay, lesbian, and bisexual (50.5%) than not sure (37.3%) students and higher among gay, lesbian, and bisexual (50.5%) than heterosexual (42.8%) students. Among female students, the prevalence was higher among lesbian and bisexual (53.2%) than heterosexual (39.6%) and not sure (36.5%) students. Among male students, the prevalence was higher among heterosexual (45.6%) than not sure (36.7%) students. The prevalence also was higher among heterosexual male (45.6%) than heterosexual female (39.6%) students and higher among lesbian and bisexual female (53.2%) than gay and bisexual male (42.2%) students.

Analyses based on the question ascertaining the sex of sexual contacts indicated that nationwide, 61.5% of students who had sexual contact with only the opposite sex, 66.8% of students who had sexual contact with only the same sex or with both sexes, and 22.9% of students who had no sexual contact had ever used an electronic vapor product (Supplementary Table 64). The prevalence of having ever used an electronic vapor product was higher among students who had sexual contact with only the opposite sex (61.5%) and students who had sexual contact with only the same sex or with both sexes (66.8%) than students who had no sexual contact (22.9%) and higher among students who had sexual contact with only the same sex or with both sexes (66.8%) than students who had sexual contact with only the opposite sex (61.5%). Among female students, the prevalence was higher among those who had sexual contact with only males (57.6%) and those who had sexual contact with only females or with both sexes (69.7%) than those who had no sexual contact (22.4%) and higher among those who had sexual contact with only females or with both sexes (69.7%) than those who had sexual contact with only males (57.6%). Among male students, the prevalence was higher among those who had sexual contact with only females (64.8%) and those who had sexual contact with only males or with both sexes (57.8%) than those who had no sexual contact (23.4%). The prevalence also was higher among male students who had sexual contact with only females (64.8%) than female students who had sexual contact with only males (57.6%) and higher among female students who had sexual contact with only females or with both sexes (69.7%) than male students who had sexual contact with only males or with both sexes (57.8%).

The question measuring the prevalence of having ever used an electronic vapor product was used for the first time in the 2015 national YRBS. As a result, long-term temporal trends are not available for this variable. The prevalence of having ever used an electronic vapor product did not change significantly from 2015 (44.9%) to 2017 (42.2%).

Analyses of state and large urban school district data indicated that across 33 states, the overall prevalence of having ever used an electronic vapor product ranged from 33.2% to 51.0% across state surveys (median: 41.1%) (Supplementary Table 65). Across 19 large urban school districts, the prevalence ranged from 25.0% to 42.0% (median: 36.6%).

#### Current Electronic Vapor Product Use

Nationwide, 13.2% of students had used an electronic vapor product (including e-cigarettes, e-cigars, e-pipes, vape pipes, vaping pens, e-hookahs, and hookah pens) on at least 1 day during the 30 days before the survey (i.e., current electronic vapor product use) (Supplementary Table 66). The prevalence of current electronic vapor product use was higher among male (15.9%) than female (10.5%) students; higher among white male (19.6%) than white female (11.8%) students; and higher among 9th-grade male (11.3%), 10th-grade male (13.4%), 11th-grade male (17.0%), and 12th-grade male (22.7%) than 9th-grade female (7.8%), 10th-grade female (9.5%), 11th-grade female (11.1%), and 12th-grade female (14.1%) students, respectively. The prevalence of current electronic vapor product use was higher among white (15.6%) and Hispanic (11.4%) than black (8.5%) students, higher among white (15.6%) than Hispanic (11.4%) students, and higher among white male (19.6%) than black male (9.2%) and Hispanic male (12.3%) students. The prevalence of current electronic vapor product use was higher among 10th-grade (11.4%), 11th-grade (14.1%), and 12th-grade (18.3%) than 9th-grade (9.5%) students; higher among 12th-grade (18.3%) than 10th-grade (11.4%) and 11th-grade (14.1%) students; higher among 12th-grade female (14.1%) than 9th-grade female (7.8%) and 10th-grade female (9.5%) students; higher among 12th-grade male (22.7%) than 9th-grade male (11.3%), 10th-grade male (13.4%), and 11th-grade male (17.0%) students; and higher among 11th-grade male (17.0%) than 9th-grade male (11.3%) students.

Analyses based on the question ascertaining sexual identity indicated that nationwide, the prevalence of current electronic vapor product use was 13.2% among heterosexual students; 17.5% among gay, lesbian, and bisexual students; and 10.8% among not sure students (Supplementary Table 66). The prevalence of current electronic vapor product use was higher among gay, lesbian, and bisexual (17.5%) than heterosexual (13.2%) and not sure (10.8%) students. Among female students, the prevalence was higher among lesbian and bisexual (17.8%) than heterosexual (9.6%) and not sure (10.3%) students. Among male students, the prevalence was higher among heterosexual (16.3%) than not sure (8.5%) students. The prevalence also was higher among heterosexual male (16.3%) than heterosexual female (9.6%) students.

Analyses based on the question ascertaining the sex of sexual contacts indicated that nationwide, the prevalence of current electronic vapor product use was 22.6% among students who had sexual contact with only the opposite sex, 27.0% among students who had sexual contact with only the same sex or with both sexes, and 3.5% among students who had no sexual contact (Supplementary Table 66). The prevalence of current electronic vapor product use was higher among students who had sexual contact with only the opposite sex (22.6%) and students who had sexual contact with only the same sex or with both sexes (27.0%) than students who had no sexual contact (3.5%) and higher among students who had sexual contact with only the same sex or with both sexes (27.0%) than students who had sexual contact with only the opposite sex (22.6%). Among female students, the prevalence was higher among those who had sexual contact with only males (16.4%) and those who had sexual contact with only females or with both sexes (28.6%) than those who had no sexual contact (3.0%) and higher among those who had sexual contact with only females or with both sexes (28.6%) than those who had sexual contact with only males (16.4%). Among male students, the prevalence was higher among those who had sexual contact with only females (27.7%) and those who had sexual contact with only males or with both sexes (22.2%) than those who had no sexual contact (4.0%). The prevalence also was higher among male students who had sexual contact with only females (27.7%) than female students who had sexual contact with only males (16.4%).

The question measuring the prevalence of current electronic vapor product use was used for the first time in the 2015 national YRBS. As a result, long-term temporal trends are not available for this variable. The prevalence of current electronic vapor product use decreased significantly from 2015 (24.1%) to 2017 (13.2%).

Analyses of state and large urban school district data indicated that across 37 states, the overall prevalence of current electronic vapor product use ranged from 7.6% to 26.2% across state surveys (median: 14.3%) (Supplementary Table 67). Across 21 large urban school districts, the prevalence ranged from 4.7% to 17.3% (median: 7.4%).

#### Current Frequent Electronic Vapor Product Use

Nationwide, 3.3% of students had used an electronic vapor product (including e-cigarettes, e-cigars, e-pipes, vape pipes, vaping pens, e-hookahs, and hookah pens) on 20 or more days during the 30 days before the survey (i.e., current frequent electronic vapor product use) (Supplementary Table 68). The prevalence of current frequent electronic vapor product use was higher among male (5.0%) than female (1.6%) students; higher among white male (6.6%), black male (2.2%), and Hispanic male (3.1%) than white female (2.2%), black female (0.5%), and Hispanic female (1.1%) students, respectively; and higher among 9th-grade male (2.6%), 10th-grade male (3.8%), 11th-grade male (6.1%), and 12th-grade male (7.9%) than 9th-grade female (1.0%), 10th-grade female (1.5%), 11th-grade female (1.4%), and 12th-grade female (2.2%) students, respectively. The prevalence of current frequent electronic vapor product use was higher among white (4.3%) than black (1.4%) and Hispanic (2.1%) students, higher among white female (2.2%) than black female (0.5%) students, and higher among white male (6.6%) than black male (2.2%) and Hispanic male (3.1%) students. The prevalence of current frequent electronic vapor product use was higher among 10th-grade (2.7%), 11th-grade (3.7%), and 12th-grade (5.0%) than 9th-grade (1.8%) students; higher among 12th-grade (5.0%) than 10th-grade (2.7%) students; higher among 11th-grade male (6.1%) and 12th-grade male (7.9%) than 9th-grade male (2.6%) students; and higher among 12th-grade male (7.9%) than 10th-grade male (3.8%) students.

Analyses based on the question ascertaining sexual identity indicated that nationwide, the prevalence of current frequent electronic vapor product use was 3.3% among heterosexual students; 4.0% among gay, lesbian, and bisexual students; and 3.4% among not sure students (Supplementary Table 68). Among female students, the prevalence of current frequent electronic vapor product use was higher among lesbian and bisexual (3.5%) than heterosexual (1.1%) students. The prevalence also was higher among heterosexual male (5.2%) than heterosexual female (1.1%) students.

Analyses based on the question ascertaining the sex of sexual contacts indicated that nationwide, the prevalence of current frequent electronic vapor product use was 6.2% among students who had sexual contact with only the opposite sex, 6.4% among students who had sexual contact with only the same sex or with both sexes, and 0.6% among students who had no sexual contact (Supplementary Table 68). The prevalence of current frequent electronic vapor product use was higher among students who had sexual contact with only the opposite sex (6.2%) and students who had sexual contact with only the same sex or with both sexes (6.4%) than students who had no sexual contact (0.6%). Among female students, the prevalence was higher among those who had sexual contact with only males (2.1%) and those who had sexual contact with only females or with both sexes (6.2%) than those who had no sexual contact (0.3%). Among male students, the prevalence was higher among those who had sexual contact with only females (9.4%) and those who had sexual contact with only males or with both sexes (7.1%) than those who had no sexual contact (1.0%). The prevalence also was higher among male students who had sexual contact with only females (9.4%) than female students who had sexual contact with only males (2.1%) and higher among male students who had no sexual contact (1.0%) than female students who had no sexual contact (0.3%).

The question measuring the prevalence of current frequent electronic vapor product use was used for the first time in the 2015 national YRBS. As a result, long-term temporal trends are not available for this variable. The prevalence of current frequent electronic vapor product use did not change significantly from 2015 (3.0%) to 2017 (3.3%).

Analyses of state and large urban school district data indicated that across 37 states, the overall prevalence of current frequent electronic vapor product use ranged from 1.5% to 5.7% across state surveys (median: 2.8%) (Supplementary Table 69). Across 21 large urban school districts, the prevalence ranged from 0.4% to 2.5% (median: 0.9%).

#### Current Daily Electronic Vapor Product Use

Nationwide, 2.4% of students had used electronic vapor products (including e-cigarettes, e-cigars, e-pipes, vape pipes, vaping pens, e-hookahs, and hookah pens) on all 30 days during the 30 days before the survey (i.e., current daily electronic vapor product use) (Supplementary Table 70). The prevalence of current daily electronic vapor product use was higher among male (3.8%) than female (1.1%) students; higher among white male (4.7%), black male (1.6%), and Hispanic male (2.5%) than white female (1.5%), black female (0.2%), and Hispanic female (0.9%) students, respectively; and higher among 9th-grade male (1.9%), 10th-grade male (2.6%), 11th-grade male (4.5%), and 12th-grade male (6.1%) than 9th-grade female (0.5%), 10th-grade female (0.7%), 11th-grade female (1.0%), and 12th-grade female (2.0%) students, respectively. The prevalence of current daily electronic vapor product use was higher among white (3.1%) than black (1.0%) and Hispanic (1.7%) students, higher among white female (1.5%) and Hispanic female (0.9%) than black female (0.2%) students, and higher among white male (4.7%) than black male (1.6%) and Hispanic male (2.5%) students. The prevalence of current daily electronic vapor product use was higher among 11th-grade (2.7%) and 12th-grade (4.0%) than 9th-grade (1.2%) and 10th-grade (1.7%) students, higher among 12th-grade female (2.0%) than 9th-grade female (0.5%) students, and higher among 11th-grade male (4.5%) and 12th-grade male (6.1%) than 9th-grade male (1.9%) and 10th-grade male (2.6%) students.

Analyses based on the question ascertaining sexual identity indicated that nationwide, the prevalence of current daily electronic vapor product use was 2.4% among heterosexual students; 2.8% among gay, lesbian, and bisexual students; and 3.1% among not sure students (Supplementary Table 70). Among female students, the prevalence of current daily electronic vapor product use was higher among lesbian and bisexual (2.7%) than heterosexual (0.7%) students. The prevalence also was higher among heterosexual male (3.8%) than heterosexual female (0.7%) students.

Analyses based on the question ascertaining the sex of sexual contacts indicated that nationwide, the prevalence of current daily electronic vapor product use was 4.5% among students who had sexual contact with only the opposite sex, 4.9% among students who had sexual contact with only the same sex or with both sexes, and 0.5% among students who had no sexual contact (Supplementary Table 70). The prevalence of current daily electronic vapor product use was higher among students who had sexual contact with only the opposite sex (4.5%) and students who had sexual contact with only the same sex or with both sexes (4.9%) than students who had no sexual contact (0.5%). Among female students, the prevalence was higher among those who had sexual contact with only males (1.4%) and those who had sexual contact with only females or with both sexes (5.1%) than those who had no sexual contact (0.2%). Among male students, the prevalence was higher among those who had sexual contact with only females (7.0%) than those who had no sexual contact (0.9%). The prevalence also was higher among male students who had sexual contact with only females (7.0%) than female students who had sexual contact with only males (1.4%) and higher among male students who had no sexual contact (0.9%) than female students who had no sexual contact (0.2%).

The question measuring the prevalence of current daily electronic vapor product use was used for the first time in the 2015 national YRBS. As a result, long-term temporal trends are not available for this variable. The prevalence of current daily electronic vapor product use did not change significantly from 2015 (2.0%) to 2017 (2.4%).

Analyses of state and large urban school district data indicated that across 37 states, the overall prevalence of current daily electronic vapor product use ranged from 0.9% to 4.0% across state surveys (median: 1.9%) (Supplementary Table 71). Across 21 large urban school districts, the prevalence ranged from 0.1% to 1.9% (median: 0.7%).

#### Usually Got Electronic Vapor Products by Buying Them in a Store

Among the 8.7% of students nationwide who currently used electronic vapor products (including e-cigarettes, e-cigars, e-pipes, vape pipes, vaping pens, e-hookahs, and hookah pens) and who were aged <18 years, 13.6% had usually gotten their own electronic vapor products by buying them in a store (e.g., convenience store, supermarket, discount store, gas station, or vape store) during the 30 days before the survey (Supplementary Table 72). The prevalence of having usually gotten their own electronic vapor products by buying them in a store was higher among 12th-grade (22.9%) than 9th-grade (8.7%), 10th-grade (11.6%), and 11th-grade (14.3%) students and higher among 12th-grade male (25.3%) than 9th-grade male (10.0%) and 10th-grade male (12.3%) students.

Analyses based on the question ascertaining sexual identity indicated that nationwide, among the students who currently used electronic vapor products and who were aged <18 years, 14.1% of heterosexual students; 10.5% of gay, lesbian, and bisexual students; and 21.3% of not sure students had usually gotten their own electronic vapor products by buying them in a store (Supplementary Table 72). Among male students, the prevalence of having usually gotten their own electronic vapor products by buying them in a store was higher among heterosexual (16.5%) than gay and bisexual (5.4%) students. The prevalence also was higher among heterosexual male (16.5%) than heterosexual female (9.7%) students.

Analyses based on the question ascertaining the sex of sexual contacts indicated that nationwide, among the students who currently used electronic vapor products and who were aged <18 years, 15.5% of students who had sexual contact with only the opposite sex, 7.5% of students who had sexual contact with only the same sex or with both sexes, and 10.7% of students who had no sexual contact had usually gotten their own electronic vapor products by buying them in a store (Supplementary Table 72). The prevalence of having usually gotten their own electronic vapor products by buying them in a store was higher among students who had sexual contact with only the opposite sex (15.5%) than students who had sexual contact with only the same sex or with both sexes (7.5%).

The question measuring the prevalence of having usually gotten their own electronic vapor products by buying them in a store was used for the first time in the 2017 national YRBS. As a result, long-term temporal trends and 2-year temporal changes are not available for this variable.

Analyses of state and large urban school district data indicated that across 26 states, the overall prevalence of having usually gotten their own electronic vapor products by buying them in a store, among the students who currently used electronic vapor products and who were aged <18 years, ranged from 6.0% to 26.7% across state surveys (median: 10.6%) (Supplementary Table 73). Across seven large urban school districts, the prevalence ranged from 10.5% to 23.6% (median: 18.4%).

#### Current Smokeless Tobacco Use

Nationwide, 5.5% of students had used chewing tobacco, snuff, dip, snus, or dissolvable tobacco products (e.g., Redman, Levi Garrett, Beechnut, Skoal, Skoal Bandits, Copenhagen, Camel Snus, Marlboro Snus, General Snus, Ariva, Stonewall, or Camel Orbs) (not counting any electronic vapor products) on at least 1 day during the 30 days before the survey (i.e., current smokeless tobacco use) (Supplementary Table 74). The prevalence of current smokeless tobacco use was higher among male (8.9%) than female (1.9%) students; higher among white male (11.9%), black male (5.0%), and Hispanic male (5.6%) than white female (2.1%), black female (1.8%), and Hispanic female (1.8%) students, respectively; and higher among 9th-grade male (6.8%), 10th-grade male (7.5%), 11th-grade male (9.7%), and 12th-grade male (12.0%) than 9th-grade female (1.5%), 10th-grade female (1.8%), 11th-grade female (1.5%), and 12th-grade female (2.7%) students, respectively. The prevalence of current smokeless tobacco use was higher among white (6.8%) than black (3.5%) and Hispanic (3.7%) students and higher among white male (11.9%) than black male (5.0%) and Hispanic male (5.6%) students. The prevalence of current smokeless tobacco use was higher among 11th-grade (5.7%) and 12th-grade (7.2%) than 9th-grade (4.1%) students, higher among 12th-grade (7.2%) than 10th-grade (4.6%) students, higher among 12th-grade female (2.7%) than 9th-grade female (1.5%) students, higher among 11th-grade male (9.7%) and 12th-grade male (12.0%) than 9th-grade male (6.8%) students, and higher among 12th-grade male (12.0%) than 10th-grade male (7.5%) students.

Analyses based on the question ascertaining sexual identity indicated that nationwide, the prevalence of current smokeless tobacco use was 5.5% among heterosexual students; 5.9% among gay, lesbian, and bisexual students; and 6.3% among not sure students (Supplementary Table 74). Among female students, the prevalence of current smokeless tobacco use was higher among lesbian and bisexual (4.0%) than heterosexual (1.4%) students. The prevalence also was higher among heterosexual male (9.0%) than heterosexual female (1.4%) students and higher among gay and bisexual male (11.1%) than lesbian and bisexual female (4.0%) students.

Analyses based on the question ascertaining the sex of sexual contacts indicated that nationwide, the prevalence of current smokeless tobacco use was 9.2% among students who had sexual contact with only the opposite sex, 8.2% among students who had sexual contact with only the same sex or with both sexes, and 1.6% among students who had no sexual contact (Supplementary Table 74). The prevalence of current smokeless tobacco use was higher among students who had sexual contact with only the opposite sex (9.2%) and students who had sexual contact with only the same sex or with both sexes (8.2%) than students who had no sexual contact (1.6%). Among female students, the prevalence was higher among those who had sexual contact with only males (2.6%) and those who had sexual contact with only females or with both sexes (6.1%) than those who had no sexual contact (0.4%) and higher among those who had sexual contact with only females or with both sexes (6.1%) than those who had sexual contact with only males (2.6%). Among male students, the prevalence was higher among those who had sexual contact with only females (14.6%) and those who had sexual contact with only males or with both sexes (14.5%) than those who had no sexual contact (2.9%). The prevalence also was higher among male students who had sexual contact with only females (14.6%) than female students who had sexual contact with only males (2.6%), higher among male students who had sexual contact with only males or with both sexes (14.5%) than female students who had sexual contact with only females or with both sexes (6.1%), and higher among male students who had no sexual contact (2.9%) than female students who had no sexual contact (0.4%).

The question measuring the prevalence of current smokeless tobacco (e.g., chewing tobacco, snuff, dip, snus, or dissolvable tobacco products, not counting any electronic vapor products) use was used for the first time in the 2017 national YRBS. As a result, long-term temporal trends and 2-year temporal changes are not available for this variable.

Analyses of state and large urban school district data indicated that across 33 states, the overall prevalence of current smokeless tobacco use ranged from 2.8% to 12.7% across state surveys (median: 5.9%) (Supplementary Table 75). Across 18 large urban school districts, the prevalence ranged from 1.9% to 5.9% (median: 3.7%).

#### Current Frequent Smokeless Tobacco Use

Nationwide, 2.1% of students had used chewing tobacco, snuff, dip, snus, or dissolvable tobacco products (e.g., Redman, Levi Garrett, Beechnut, Skoal, Skoal Bandits, Copenhagen, Camel Snus, Marlboro Snus, General Snus, Ariva, Stonewall, or Camel Orbs) (not counting any electronic vapor products) on 20 or more days during the 30 days before the survey (i.e., current frequent smokeless tobacco use) (Supplementary Table 76). The prevalence of current frequent smokeless tobacco use was higher among male (3.7%) than female (0.4%) students; higher among white male (5.5%), black male (1.5%), and Hispanic male (1.6%) than white female (0.5%), black female (0.2%), and Hispanic female (0.3%) students, respectively; and higher among 9th-grade male (1.6%), 10th-grade male (3.2%), 11th-grade male (4.0%), and 12th-grade male (6.1%) than 9th-grade female (0.2%), 10th-grade female (0.3%), 11th-grade female (0.0%), and 12th-grade female (0.8%) students, respectively. The prevalence of current frequent smokeless tobacco use was higher among white (2.9%) than black (0.9%) and Hispanic (1.0%) students and higher among white male (5.5%) than black male (1.5%) and Hispanic male (1.6%) students. The prevalence of current frequent smokeless tobacco use was higher among 10th-grade (1.7%), 11th-grade (2.0%), and 12th-grade (3.4%) than 9th-grade (0.9%) students; higher among 12th-grade (3.4%) than 10th-grade (1.7%) students; higher among 12th-grade female (0.8%) than 11th-grade female (0.0%) students; higher among 10th-grade male (3.2%), 11th-grade male (4.0%), and 12th-grade male (6.1%) than 9th-grade male (1.6%) students; and higher among 12th-grade male (6.1%) than 10th-grade male (3.2%) students.

Analyses based on the question ascertaining sexual identity indicated that nationwide, the prevalence of current frequent smokeless tobacco use was 2.1% among heterosexual students; 1.0% among gay, lesbian, and bisexual students; and 4.1% among not sure students (Supplementary Table 76). The prevalence of current frequent smokeless tobacco use was higher among heterosexual (2.1%) than gay, lesbian, and bisexual (1.0%) students. The prevalence also was higher among heterosexual male (3.7%) than heterosexual female (0.3%) students and higher among gay and bisexual male (2.7%) than lesbian and bisexual female (0.4%) students.

Analyses based on the question ascertaining the sex of sexual contacts indicated that nationwide, the prevalence of current frequent smokeless tobacco use was 3.8% among students who had sexual contact with only the opposite sex, 3.6% among students who had sexual contact with only the same sex or with both sexes, and 0.3% among students who had no sexual contact (Supplementary Table 76). The prevalence of current frequent smokeless tobacco use was higher among students who had sexual contact with only the opposite sex (3.8%) and students who had sexual contact with only the same sex or with both sexes (3.6%) than students who had no sexual contact (0.3%). Among female students, the prevalence was higher among those who had sexual contact with only females or with both sexes (1.8%) than those who had no sexual contact (0.1%). Among male students, the prevalence was higher among those who had sexual contact with only females (6.5%) and those who had sexual contact with only males or with both sexes (9.0%) than those who had no sexual contact (0.6%). The prevalence also was higher among male students who had sexual contact with only females (6.5%) than female students who had sexual contact with only males (0.4%) and higher among male students who had sexual contact with only males or with both sexes (9.0%) than female students who had sexual contact with only females or with both sexes (1.8%).

The question measuring the prevalence of current frequent smokeless tobacco (e.g., chewing tobacco, snuff, dip, snus, or dissolvable tobacco products, not counting any electronic vapor products) use was used for the first time in the 2017 national YRBS. As a result, long-term temporal trends and 2-year temporal changes are not available for this variable.

Analyses of state and large urban school district data indicated that across 33 states, the overall prevalence of current frequent smokeless tobacco use ranged from 0.6% to 5.8% across state surveys (median: 1.6%) (Supplementary Table 77). Across 18 large urban school districts, the prevalence ranged from 0.3% to 1.4% (median: 0.6%).

#### Current Daily Smokeless Tobacco Use

Nationwide, 1.6% of students had used chewing tobacco, snuff, dip, snus, or dissolvable tobacco products, (e.g., Redman, Levi Garrett, Beechnut, Skoal, Skoal Bandits, Copenhagen, Camel Snus, Marlboro Snus, General Snus, Ariva, Stonewall, or Camel Orbs) (not counting any electronic vapor products) on all 30 days during the 30 days before the survey (i.e., current daily smokeless tobacco use) (Supplementary Table 78). The prevalence of current daily smokeless tobacco use was higher among male (2.8%) than female (0.3%) students; higher among white male (4.2%), black male (1.0%), and Hispanic male (1.2%) than white female (0.3%), black female (0.2%), and Hispanic female (0.3%) students, respectively; and higher among 9th-grade male (1.0%), 10th-grade male (2.8%), 11th-grade male (3.0%), and 12th-grade male (4.7%) than 9th-grade female (0.2%), 10th-grade female (0.3%), 11th-grade female (0.0%), and 12th-grade female (0.6%) students, respectively. The prevalence of current daily smokeless tobacco use was higher among white (2.2%) than black (0.6%) and Hispanic (0.8%) students and higher among white male (4.2%) than black male (1.0%) and Hispanic male (1.2%) students. The prevalence of current daily smokeless tobacco use was higher among 10th-grade (1.5%), 11th-grade (1.5%), and 12th-grade (2.6%) than 9th-grade (0.6%) students; higher among 12th-grade (2.6%) than 10th-grade (1.5%) students; and higher among 10th-grade male (2.8%), 11th-grade male (3.0%), and 12th-grade male (4.7%) than 9th-grade male (1.0%) students.

Analyses based on the question ascertaining sexual identity indicated that nationwide, the prevalence of current daily smokeless tobacco use was 1.6% among heterosexual students; 0.7% among gay, lesbian, and bisexual students; and 3.5% among not sure students (Supplementary Table 78). The prevalence of current daily smokeless tobacco use was higher among heterosexual (1.6%) than gay, lesbian, and bisexual (0.7%) students. The prevalence also was higher among heterosexual male (2.8%) than heterosexual female (0.2%) students and higher among gay and bisexual male (2.3%) than lesbian and bisexual female (0.2%) students.

Analyses based on the question ascertaining the sex of sexual contacts indicated that nationwide, the prevalence of current daily smokeless tobacco use was 2.9% among students who had sexual contact with only the opposite sex, 2.8% among students who had sexual contact with only the same sex or with both sexes, and 0.3% among students who had no sexual contact (Supplementary Table 78). The prevalence of current daily smokeless tobacco use was higher among students who had sexual contact with only the opposite sex (2.9%) and students who had sexual contact with only the same sex or with both sexes (2.8%) than students who had no sexual contact (0.3%). Among male students, the prevalence was higher among those who had sexual contact with only females (5.0%) and those who had sexual contact with only males or with both sexes (7.0%) than those who had no sexual contact (0.5%). The prevalence also was higher among male students who had sexual contact with only females (5.0%) than female students who had sexual contact with only males (0.3%).

The question measuring the prevalence of current daily smokeless tobacco (e.g., chewing tobacco, snuff, dip, snus, or dissolvable tobacco products, not counting any electronic vapor products) use was used for the first time in the 2017 national YRBS. As a result, long-term temporal trends and 2-year temporal changes are not available for this variable.

Analyses of state and large urban school district data indicated that across 33 states, the overall prevalence of current daily smokeless tobacco use ranged from 0.4% to 5.1% across state surveys (median: 1.4%) (Supplementary Table 79). Across 18 large urban school districts, the prevalence ranged from 0.1% to 1.2% (median: 0.4%).

#### Current Cigar Use

Nationwide, 8.0% of students had smoked cigars, cigarillos, or little cigars on at least 1 day during the 30 days before the survey (i.e., current cigar use) (Supplementary Table 80). The prevalence of current cigar use was higher among male (10.5%) than female (5.4%) students; higher among white male (12.7%) and Hispanic male (7.6%) than white female (5.5%) and Hispanic female (5.0%) students, respectively; and higher among 9th-grade male (6.1%), 10th-grade male (7.4%), 11th-grade male (11.3%), and 12th-grade male (18.0%) than 9th-grade female (3.9%), 10th-grade female (3.6%), 11th-grade female (7.0%), and 12th-grade female (7.4%) students, respectively. The prevalence of current cigar use was higher among white (9.0%) than Hispanic (6.3%) students and higher among white male (12.7%) than black male (8.7%) and Hispanic male (7.6%) students. The prevalence of current cigar use was higher among 11th-grade (9.2%) and 12th-grade (12.5%) than 9th-grade (5.0%) and 10th-grade (5.5%) students, higher among 12th-grade (12.5%) than 11th-grade (9.2%) students, higher among 11th-grade female (7.0%) and 12th-grade female (7.4%) than 9th-grade female (3.9%) and 10th-grade female (3.6%) students, higher among 11th-grade male (11.3%) and 12th-grade male (18.0%) than 9th-grade male (6.1%) and 10th-grade male (7.4%) students, and higher among 12th-grade male (18.0%) than 11th-grade male (11.3%) students.

Analyses based on the question ascertaining sexual identity indicated that nationwide, the prevalence of current cigar use was 7.7% among heterosexual students; 10.8% among gay, lesbian, and bisexual students; and 10.9% among not sure students (Supplementary Table 80). The prevalence of current cigar use was higher among gay, lesbian, and bisexual (10.8%) than heterosexual (7.7%) students. Among female students, the prevalence was higher among lesbian and bisexual (10.1%) than heterosexual (4.5%) students. The prevalence also was higher among heterosexual male (10.4%) than heterosexual female (4.5%) students.

Analyses based on the question ascertaining the sex of sexual contacts indicated that nationwide, the prevalence of current cigar use was 13.2% among students who had sexual contact with only the opposite sex, 18.7% among students who had sexual contact with only the same sex or with both sexes, and 1.9% among students who had no sexual contact (Supplementary Table 80). The prevalence of current cigar use was higher among students who had sexual contact with only the opposite sex (13.2%) and students who had sexual contact with only the same sex or with both sexes (18.7%) than students who had no sexual contact (1.9%) and higher among students who had sexual contact with only the same sex or with both sexes (18.7%) than students who had sexual contact with only the opposite sex (13.2%). Among female students, the prevalence was higher among those who had sexual contact with only males (7.6%) and those who had sexual contact with only females or with both sexes (18.3%) than those who had no sexual contact (1.2%) and higher among those who had sexual contact with only females or with both sexes (18.3%) than those who had sexual contact with only males (7.6%). Among male students, the prevalence was higher among those who had sexual contact with only females (17.7%) and those who had sexual contact with only males or with both sexes (20.1%) than those who had no sexual contact (2.6%). The prevalence also was higher among male students who had sexual contact with only females (17.7%) than female students who had sexual contact with only males (7.6%) and higher among male students who had no sexual contact (2.6%) than female students who had no sexual contact (1.2%).

Trend analyses indicated that during 1997–2017, a significant linear decrease (22.0%–8.0%) occurred in the overall prevalence of current cigar use. A significant quadratic trend was not identified. The prevalence of current cigar use decreased from 2015 (10.3%) to 2017 (8.0%).

Analyses of state and large urban school district data indicated that across 33 states, the overall prevalence of current cigar use ranged from 3.2% to 14.1% across state surveys (median: 7.7%) (Supplementary Table 81). Across 19 large urban school districts, the prevalence ranged from 2.7% to 10.5% (median: 6.3%).

#### Current Frequent Cigar Use

Nationwide, 1.3% of students had smoked cigars, cigarillos, or little cigars on 20 or more days during the 30 days before the survey (i.e., current frequent cigar use) (Supplementary Table 82). The prevalence of current frequent cigar use was higher among male (1.7%) than female (0.7%) students; higher among white male (1.7%) and Hispanic male (1.5%) than white female (0.7%) and Hispanic female (0.6%) students, respectively; and higher among 10th-grade male (1.2%) than 10th-grade female (0.2%) students. The prevalence of current frequent cigar use was higher among 11th-grade (1.4%) and 12th-grade (2.2%) than 9th-grade (0.6%) students, higher among 12th-grade (2.2%) than 10th-grade (0.7%) students, higher among 11th-grade female (0.8%) and 12th-grade female (1.5%) than 9th-grade female (0.3%) and 10th-grade female (0.2%) students, and higher among 12th-grade male (2.8%) than 9th-grade male (1.0%) and 10th-grade male (1.2%) students.

Analyses based on the question ascertaining sexual identity indicated that nationwide, the prevalence of current frequent cigar use was 1.1% among heterosexual students; 1.5% among gay, lesbian, and bisexual students; and 4.3% among not sure students (Supplementary Table 82). The prevalence of current frequent cigar use was higher among heterosexual male (1.4%) than heterosexual female (0.6%) students.

Analyses based on the question ascertaining the sex of sexual contacts indicated that nationwide, the prevalence of current frequent cigar use was 1.9% among students who had sexual contact with only the opposite sex, 4.1% among students who had sexual contact with only the same sex or with both sexes, and 0.2% among students who had no sexual contact (Supplementary Table 82). The prevalence of current frequent cigar use was higher among students who had sexual contact with only the opposite sex (1.9%) and students who had sexual contact with only the same sex or with both sexes (4.1%) than students who had no sexual contact (0.2%). Among female students, the prevalence was higher among those who had sexual contact with only males (0.9%) and those who had sexual contact with only females or with both sexes (2.7%) than those who had no sexual contact (0.2%). Among male students, the prevalence was higher among those who had sexual contact with only females (2.7%) and those who had sexual contact with only males or with both sexes (8.1%) than those who had no sexual contact (0.1%). The prevalence also was higher among male students who had sexual contact with only females (2.7%) than female students who had sexual contact with only males (0.9%).

Trend analyses did not identify a significant linear trend in the overall prevalence of current frequent cigar use during 1997–2017 (1.3%–1.3%). A significant quadratic trend was identified. The prevalence of current frequent cigar use increased during 1997–2013 (1.3%–1.8%) and then decreased during 2013–2017 (1.8%–1.3%). The prevalence of current frequent cigar use did not change significantly from 2015 (1.3%) to 2017 (1.3%).

Analyses of state and large urban school district data indicated that across 33 states, the overall prevalence of current frequent cigar use ranged from 0.4% to 2.9% across state surveys (median: 1.1%) (Supplementary Table 83). Across 19 large urban school districts, the prevalence ranged from 0.5% to 1.6% (median: 1.0%).

#### Current Daily Cigar Use

Nationwide, 1.0% of students had smoked cigars, cigarillos, or little cigars on all 30 days during the 30 days before the survey (i.e., current daily cigar use) (Supplementary Table 84). The prevalence of current daily cigar use was higher among male (1.2%) than female (0.6%) students and higher among 10th-grade male (1.2%) than 10th-grade female (0.2%) students. The prevalence of current daily cigar use was higher among 12th-grade (1.7%) than 9th-grade (0.4%), 10th-grade (0.7%), and 11th-grade (0.8%) students; higher among 11th-grade (0.8%) than 9th-grade (0.4%) students; higher among 12th-grade female (1.4%) than 9th-grade female (0.2%) and 10th-grade female (0.2%) students; and higher among 12th-grade male (2.0%) than 9th-grade male (0.6%) students.

Analyses based on the question ascertaining sexual identity indicated that nationwide, the prevalence of current daily cigar use was 0.8% among heterosexual students; 1.1% among gay, lesbian, and bisexual students; and 3.9% among not sure students (Supplementary Table 84). The prevalence of current daily cigar use was higher among heterosexual male (1.0%) than heterosexual female (0.5%) students.

Analyses based on the question ascertaining the sex of sexual contacts indicated that nationwide, the prevalence of current daily cigar use was 1.4% among students who had sexual contact with only the opposite sex, 3.4% among students who had sexual contact with only the same sex or with both sexes, and 0.1% among students who had no sexual contact (Supplementary Table 84). The prevalence of current daily cigar use was higher among students who had sexual contact with only the opposite sex (1.4%) and students who had sexual contact with only the same sex or with both sexes (3.4%) than students who had no sexual contact (0.1%) and higher among students who had sexual contact with only the same sex or with both sexes (3.4%) than students who had sexual contact with only the opposite sex (1.4%). Among female students, the prevalence was higher among those who had sexual contact with only males (0.8%) and those who had sexual contact with only females or with both sexes (2.0%) than those who had no sexual contact (0.1%). Among male students, the prevalence was higher among those who had sexual contact with only females (1.8%) and those who had sexual contact with only males or with both sexes (7.7%) than those who had no sexual contact (0.1%). The prevalence also was higher among male students who had sexual contact with only females (1.8%) than female students who had sexual contact with only males (0.8%).

Trend analyses did not identify a significant linear trend in the overall prevalence of current daily cigar use during 1997–2017 (0.9%–1.0%). A significant quadratic trend was identified. The prevalence of current daily cigar use increased during 1997–2011 (0.9%–1.4%) and then decreased during 2011–2017 (1.4%–1.0%). The prevalence of current daily cigar use did not change significantly from 2015 (1.0%) to 2017 (1.0%).

Analyses of state and large urban school district data indicated that across 33 states, the overall prevalence of current daily cigar use ranged from 0.3% to 2.4% across state surveys (median: 0.8%) (Supplementary Table 85). Across 19 large urban school districts, the prevalence ranged from 0.3% to 1.1% (median: 0.7%).

#### Current Cigarette or Cigar Use

Nationwide, 12.3% of students had smoked cigarettes or cigars on at least 1 day during the 30 days before the survey (i.e., current cigarette or cigar use) (Supplementary Table 86). The prevalence of current cigarette or cigar use was higher among male (14.3%) than female (10.3%) students, higher among white male (17.5%) than white female (11.8%) students, and higher among 10th-grade male (11.3%) and 12th-grade male (23.6%) than 10th-grade female (8.4%) and 12th-grade female (14.6%) students, respectively. The prevalence of current cigarette or cigar use was higher among white (14.5%) than black (9.5%) and Hispanic (9.9%) students and higher among white male (17.5%) than black male (10.7%) and Hispanic male (10.6%) students. The prevalence of current cigarette or cigar use was higher among 10th-grade (9.8%), 11th-grade (13.4%), and 12th-grade (18.9%) than 9th-grade (7.6%) students; higher among 11th-grade (13.4%) and 12th-grade (18.9%) than 10th-grade (9.8%) students; higher among 12th-grade (18.9%) than 11th-grade (13.4%) students; higher among 11th-grade female (11.9%) and 12th-grade female (14.6%) than 9th-grade female (6.6%) and 10th-grade female (8.4%) students; higher among 10th-grade male (11.3%), 11th-grade male (14.8%), and 12th-grade male (23.6%) than 9th-grade male (8.6%) students; and higher among 12th-grade male (23.6%) than 10th-grade male (11.3%) and 11th-grade male (14.8%) students.

Analyses based on the question ascertaining sexual identity indicated that nationwide, the prevalence of current cigarette or cigar use was 11.6% among heterosexual students; 19.8% among gay, lesbian, and bisexual students; and 14.7% among not sure students (Supplementary Table 86). The prevalence of current cigarette or cigar use was higher among gay, lesbian, and bisexual (19.8%) than heterosexual (11.6%) and not sure (14.7%) students. Among female students, the prevalence was higher among lesbian and bisexual (19.0%) than heterosexual (8.9%) and not sure (12.0%) students. Among male students, the prevalence was higher among gay and bisexual (21.3%) than heterosexual (14.0%) students. The prevalence also was higher among heterosexual male (14.0%) than heterosexual female (8.9%) students.

Analyses based on the question ascertaining the sex of sexual contacts indicated that nationwide, the prevalence of current cigarette or cigar use was 19.9% among students who had sexual contact with only the opposite sex, 30.9% among students who had sexual contact with only the same sex or with both sexes, and 3.0% among students who had no sexual contact (Supplementary Table 86). The prevalence of current cigarette or cigar use was higher among students who had sexual contact with only the opposite sex (19.9%) and students who had sexual contact with only the same sex or with both sexes (30.9%) than students who had no sexual contact (3.0%) and higher among students who had sexual contact with only the same sex or with both sexes (30.9%) than students who had sexual contact with only the opposite sex (19.9%). Among female students, the prevalence was higher among those who had sexual contact with only males (15.7%) and those who had sexual contact with only females or with both sexes (30.9%) than those who had no sexual contact (2.4%) and higher among those who had sexual contact with only females or with both sexes (30.9%) than those who had sexual contact with only males (15.7%). Among male students, the prevalence was higher among those who had sexual contact with only females (23.3%) and those who had sexual contact with only males or with both sexes (31.0%) than those who had no sexual contact (3.7%). The prevalence also was higher among male students who had sexual contact with only females (23.3%) than female students who had sexual contact with only males (15.7%) and higher among male students who had no sexual contact (3.7%) than female students who had no sexual contact (2.4%).

Trend analyses indicated that during 1997–2017, a significant linear decrease (42.6%–12.3%) occurred in the overall prevalence of current cigarette or cigar use. A significant quadratic trend was not identified. The prevalence of current cigarette or cigar use decreased from 2015 (16.0%) to 2017 (12.3%).

Analyses of state and large urban school district data indicated that across 33 states, the overall prevalence of current cigarette or cigar use ranged from 5.0% to 19.7% across state surveys (median: 12.7%) (Supplementary Table 87). Across 17 large urban school districts, the prevalence ranged from 3.6% to 11.2% (median: 8.3%).

#### Current Cigarette, Cigar, or Smokeless Tobacco Use

Nationwide, 14.0% of students had smoked cigarettes or cigars or used smokeless tobacco on at least 1 day during the 30 days before the survey (i.e., current cigarette, cigar, or smokeless tobacco use) (Supplementary Table 88). The prevalence of current cigarette, cigar, or smokeless tobacco use was higher among male (17.3%) than female (10.7%) students; higher among white male (21.7%) than white female (12.3%) students; and higher among 9th-grade male (11.4%), 10th-grade male (14.1%), 11th-grade male (17.6%), and 12th-grade male (26.9%) than 9th-grade female (6.9%), 10th-grade female (8.7%), 11th-grade female (12.5%), and 12th-grade female (14.9%) students, respectively. The prevalence of current cigarette, cigar, or smokeless tobacco use was higher among white (16.8%) than black (10.2%) and Hispanic (10.5%) students and higher among white male (21.7%) than black male (11.9%) and Hispanic male (11.9%) students. The prevalence of current cigarette, cigar, or smokeless tobacco use was higher among 10th-grade (11.4%), 11th-grade (15.1%), and 12th-grade (20.7%) than 9th-grade (9.1%) students; higher among 11th-grade (15.1%) and 12th-grade (20.7%) than 10th-grade (11.4%) students; higher among 12th-grade (20.7%) than 11th-grade (15.1%) students; higher among 11th-grade female (12.5%) and 12th-grade female (14.9%) than 9th-grade female (6.9%) and 10th-grade female (8.7%) students; higher among 11th-grade male (17.6%) and 12th-grade male (26.9%) than 9th-grade male (11.4%) and 10th-grade male (14.1%) students; and higher among 12th-grade male (26.9%) than 11th-grade male (17.6%) students.

Analyses based on the question ascertaining sexual identity indicated that nationwide, the prevalence of current cigarette, cigar, or smokeless tobacco use was 13.5% among heterosexual students; 20.5% among gay, lesbian, and bisexual students; and 15.6% among not sure students (Supplementary Table 88). The prevalence of current cigarette, cigar, or smokeless tobacco use was higher among gay, lesbian, and bisexual (20.5%) than heterosexual (13.5%) students. Among female students, the prevalence was higher among lesbian and bisexual (19.6%) than heterosexual (9.2%) and not sure (13.1%) students. The prevalence also was higher among heterosexual male (17.2%) than heterosexual female (9.2%) students.

Analyses based on the question ascertaining the sex of sexual contacts indicated that nationwide, the prevalence of current cigarette, cigar, or smokeless tobacco use was 22.7% among students who had sexual contact with only the opposite sex, 31.6% among students who had sexual contact with only the same sex or with both sexes, and 3.9% among students who had no sexual contact (Supplementary Table 88). The prevalence of current cigarette, cigar, or smokeless tobacco use was higher among students who had sexual contact with only the opposite sex (22.7%) and students who had sexual contact with only the same sex or with both sexes (31.6%) than students who had no sexual contact (3.9%) and higher among students who had sexual contact with only the same sex or with both sexes (31.6%) than students who had sexual contact with only the opposite sex (22.7%). Among female students, the prevalence was higher among those who had sexual contact with only males (16.3%) and those who had sexual contact with only females or with both sexes (31.5%) than those who had no sexual contact (2.6%) and higher among those who had sexual contact with only females or with both sexes (31.5%) than those who had sexual contact with only males (16.3%). Among male students, the prevalence was higher among those who had sexual contact with only females (28.0%) and those who had sexual contact with only males or with both sexes (32.2%) than those who had no sexual contact (5.2%). The prevalence also was higher among male students who had sexual contact with only females (28.0%) than female students who had sexual contact with only males (16.3%) and higher among male students who had no sexual contact (5.2%) than female students who had no sexual contact (2.6%).

The question measuring the prevalence of current smokeless tobacco use that is used to calculate current cigarette, cigar, or smokeless tobacco use was used for the first time in the 2017 national YRBS. As a result, long-term temporal trends and 2-year temporal changes are not available for this variable.

Analyses of state and large urban school district data indicated that across 32 states, the overall prevalence of current cigarette, cigar, or smokeless tobacco use ranged from 5.8% to 23.1% across state surveys (median: 14.4%) (Supplementary Table 89). Across 17 large urban school districts, the prevalence ranged from 4.3% to 13.6% (median: 9.5%).

#### Current Cigarette, Cigar, Smokeless Tobacco, or Electronic Vapor Product Use

Nationwide, 19.5% of students had smoked cigarettes or cigars or used smokeless tobacco or an electronic vapor product on at least 1 day during the 30 days before the survey (i.e., current cigarette, cigar, smokeless tobacco, or electronic vapor product use) (Supplementary Table 90). The prevalence of current cigarette, cigar, smokeless tobacco, or electronic vapor product use was higher among male (23.4%) than female (15.6%) students; higher among white male (28.1%) and Hispanic male (18.5%) than white female (17.2%) and Hispanic female (14.6%) students, respectively; and higher among 9th-grade male (16.3%), 10th-grade male (19.6%), 11th-grade male (24.3%), and 12th-grade male (34.5%) than 9th-grade female (10.9%), 10th-grade female (13.3%), 11th-grade female (17.8%), and 12th-grade female (20.8%) students, respectively. The prevalence of current cigarette, cigar, smokeless tobacco, or electronic vapor product use was higher among white (22.4%) than black (14.9%) and Hispanic (16.6%) students and higher among white male (28.1%) than black male (16.2%) and Hispanic male (18.5%) students. The prevalence of current cigarette, cigar, smokeless tobacco, or electronic vapor product use was higher among 10th-grade (16.4%), 11th-grade (21.1%), and 12th-grade (27.5%) than 9th-grade (13.6%) students; higher among 11th-grade (21.1%) and 12th-grade (27.5%) than 10th-grade (16.4%) students; higher among 12th-grade (27.5%) than 11th-grade (21.1%) students; higher among 11th-grade female (17.8%) and 12th-grade female (20.8%) than 9th-grade female (10.9%) and 10th-grade female (13.3%) students; higher among 10th-grade male (19.6%), 11th-grade male (24.3%), and 12th-grade male (34.5%) than 9th-grade male (16.3%) students; higher among 11th-grade male (24.3%) and 12th-grade male (34.5%) than 10th-grade male (19.6%) students; and higher among 12th-grade male (34.5%) than 11th-grade male (24.3%) students.

Analyses based on the question ascertaining sexual identity indicated that nationwide, the prevalence of current cigarette, cigar, smokeless tobacco, or electronic vapor product use was 19.2% among heterosexual students; 27.2% among gay, lesbian, and bisexual students; and 18.7% among not sure students (Supplementary Table 90). The prevalence of current cigarette, cigar, smokeless tobacco, or electronic vapor product use was higher among gay, lesbian, and bisexual (27.2%) than heterosexual (19.2%) and not sure (18.7%) students. Among female students, the prevalence was higher among lesbian and bisexual (27.5%) than heterosexual (14.1%) and not sure (16.5%) students. The prevalence also was higher among heterosexual male (23.6%) than heterosexual female (14.1%) students.

Analyses based on the question ascertaining the sex of sexual contacts indicated that nationwide, the prevalence of current cigarette, cigar, smokeless tobacco, or electronic vapor product use was 32.5% among students who had sexual contact with only the opposite sex, 41.5% among students who had sexual contact with only the same sex or with both sexes, and 5.7% among students who had no sexual contact (Supplementary Table 90). The prevalence of current cigarette, cigar, smokeless tobacco, or electronic vapor product use was higher among students who had sexual contact with only the opposite sex (32.5%) and students who had sexual contact with only the same sex or with both sexes (41.5%) than students who had no sexual contact (5.7%) and higher among students who had sexual contact with only the same sex or with both sexes (41.5%) than students who had sexual contact with only the opposite sex (32.5%). Among female students, the prevalence was higher among those who had sexual contact with only males (24.4%) and those who had sexual contact with only females or with both sexes (42.2%) than those who had no sexual contact (4.5%) and higher among those who had sexual contact with only females or with both sexes (42.2%) than those who had sexual contact with only males (24.4%). Among male students, the prevalence was higher among those who had sexual contact with only females (39.0%) and those who had sexual contact with only males or with both sexes (39.1%) than those who had no sexual contact (7.0%). The prevalence also was higher among male students who had sexual contact with only females (39.0%) than female students who had sexual contact with only males (24.4%) and higher among male students who had no sexual contact (7.0%) than female students who had no sexual contact (4.5%).

The question measuring the prevalence of current smokeless tobacco use that is used to calculate current cigarette, cigar, smokeless tobacco, or electronic vapor product use was used for the first time in the 2017 national YRBS. As a result, long-term temporal trends and 2-year temporal changes are not available for this variable.

Analyses of state and large urban school district data indicated that across 32 states, the overall prevalence of current cigarette, cigar, smokeless tobacco, or electronic vapor product use ranged from 9.7% to 32.7% across state surveys (median: 21.5%) (Supplementary Table 91). Across 17 large urban school districts, the prevalence ranged from 7.1% to 21.4% (median: 13.6%).

#### Tried to Quit Using All Tobacco Products

Among the 24.2% of students nationwide who used any tobacco products during the past 12 months, 41.4% had ever tried to quit using all tobacco products (including cigarettes, cigars, smokeless tobacco, shisha or hookah tobacco, and electronic vapor products) during the 12 months before the survey (Supplementary Table 92). The prevalence of having tried to quit using all tobacco products was higher among female (47.7%) than male (36.8%) students; higher among white female (51.8%) than white male (36.6%) students; and higher among 10th-grade female (49.2%), 11th-grade female (52.2%), and 12th-grade female (47.4%) than 10th-grade male (38.7%), 11th-grade male (35.3%), and 12th-grade male (32.3%) students, respectively. The prevalence of having tried to quit using all tobacco products was higher among white (42.8%) and Hispanic (42.8%) than black (32.2%) students and higher among white female (51.8%) and Hispanic female (47.9%) than black female (33.0%) students. The prevalence of having tried to quit using all tobacco products was higher among 9th-grade male (43.9%) than 11th-grade male (35.3%) and 12th-grade male (32.3%) students.

Analyses based on the question ascertaining sexual identity indicated that nationwide, among the 19.5% of students who used any tobacco products during the past 12 months, 39.4% of heterosexual students; 53.0% of gay, lesbian, and bisexual students; and 47.6% of not sure students had tried to quit using all tobacco products (Supplementary Table 92). The prevalence of having tried to quit using all tobacco products was higher among gay, lesbian, and bisexual (53.0%) than heterosexual (39.4%) students. Among female students, the prevalence was higher among not sure (69.2%) than heterosexual (45.2%) students. The prevalence also was higher among heterosexual female (45.2%) than heterosexual male (36.2%) students and higher among not sure female (69.2%) than not sure male (23.7%) students.

Analyses based on the question ascertaining the sex of sexual contacts indicated that nationwide, among the 19.5% of students who used any tobacco products during the past 12 months, 41.0% of students who had sexual contact with only the opposite sex, 49.6% of students who had sexual contact with only the same sex or with both sexes, and 41.9% of students who had no sexual contact had tried to quit using all tobacco products (Supplementary Table 92). The prevalence of having tried to quit using all tobacco products was higher among students who had sexual contact with only the same sex or with both sexes (49.6%) than students who had sexual contact with only the opposite sex (41.0%). The prevalence also was higher among female students who had sexual contact with only males (49.8%) than male students who had sexual contact with only females (36.3%).

The question measuring the prevalence of having tried to quit using all tobacco products was used for the first time in the 2017 national YRBS. As a result, long-term temporal trends and 2-year temporal changes are not available for this variable.

Analyses of state and large urban school district data indicated that across 21 states, the overall prevalence of having tried to quit using all tobacco products, among the 19.5% of students who used any tobacco products during the past 12 months, ranged from 33.0% to 50.7% across state surveys (median: 45.1%) (Supplementary Table 93). Across 17 large urban school districts, the prevalence ranged from 34.8% to 46.0% (median: 39.1%).

### Alcohol and Other Drug Use

#### Ever Drank Alcohol

Nationwide, 60.4% of students had had at least one drink of alcohol on at least 1 day during their life (i.e., ever drank alcohol) (Supplementary Table 94). The prevalence of having ever drunk alcohol was higher among female (62.6%) than male (58.1%) students and higher among black female (57.3%) than black male (44.8%) students. The prevalence of having ever drunk alcohol was higher among white (61.7%) and Hispanic (64.7%) than black (51.3%) students, higher among Hispanic female (67.1%) than black female (57.3%) students, and higher among white male (60.5%) and Hispanic male (62.3%) than black male (44.8%) students. The prevalence of having ever drunk alcohol was higher among 10th-grade (58.0%), 11th-grade (66.4%), and 12th-grade (71.7%) than 9th-grade (47.7%) students; higher among 11th-grade (66.4%) and 12th-grade (71.7%) than 10th-grade (58.0%) students; higher among 12th-grade (71.7%) than 11th-grade (66.4%) students; higher among 10th-grade female (59.9%), 11th-grade female (68.9%), and 12th-grade female (74.0%) than 9th-grade female (49.6%) students; higher among 11th-grade female (68.9%) and 12th-grade female (74.0%) than 10th-grade female (59.9%) students; higher among 12th-grade female (74.0%) than 11th-grade female (68.9%) students; higher among 10th-grade male (56.0%), 11th-grade male (63.7%), and 12th-grade male (69.4%) than 9th-grade male (45.7%) students; higher among 11th-grade male (63.7%) and 12th-grade male (69.4%) than 10th-grade male (56.0%) students; and higher among 12th-grade male (69.4%) than 11th-grade male (63.7%) students.

Analyses based on the question ascertaining sexual identity indicated that nationwide, 60.9% of heterosexual students; 72.2% of gay, lesbian, and bisexual students; and 50.0% of not sure students had ever drunk alcohol (Supplementary Table 94). The prevalence of having ever drunk alcohol was higher among heterosexual (60.9%) and gay, lesbian, and bisexual (72.2%) than not sure (50.0%) students and higher among gay, lesbian, and bisexual (72.2%) than heterosexual (60.9%) students. Among female students, the prevalence was higher among heterosexual (63.8%) and lesbian and bisexual (74.3%) than not sure (50.6%) students and higher among lesbian and bisexual (74.3%) than heterosexual (63.8%) students. Among male students, the prevalence was higher among heterosexual (58.5%) and gay and bisexual (66.3%) than not sure (47.3%) students. The prevalence also was higher among heterosexual female (63.8%) than heterosexual male (58.5%) students.

Analyses based on the question ascertaining the sex of sexual contacts indicated that nationwide, 81.5% of students who had sexual contact with only the opposite sex, 86.8% of students who had sexual contact with only the same sex or with both sexes, and 41.6% of students who had no sexual contact had ever drunk alcohol (Supplementary Table 94). The prevalence of having ever drunk alcohol was higher among students who had sexual contact with only the opposite sex (81.5%) and students who had sexual contact with only the same sex or with both sexes (86.8%) than students who had no sexual contact (41.6%) and higher among students who had sexual contact with only the same sex or with both sexes (86.8%) than students who had sexual contact with only the opposite sex (81.5%). Among female students, the prevalence was higher among those who had sexual contact with only males (85.3%) and those who had sexual contact with only females or with both sexes (89.3%) than those who had no sexual contact (44.4%) and higher among those who had sexual contact with only females or with both sexes (89.3%) than those who had sexual contact with only males (85.3%). Among male students, the prevalence was higher among those who had sexual contact with only females (78.4%) and those who had sexual contact with only males or with both sexes (79.6%) than those who had no sexual contact (38.6%). The prevalence also was higher among female students who had sexual contact with only males (85.3%) than male students who had sexual contact with only females (78.4%), higher among female students who had sexual contact with only females or with both sexes (89.3%) than male students who had sexual contact with only males or with both sexes (79.6%), and higher among female students who had no sexual contact (44.4%) than male students who had no sexual contact (38.6%).

Trend analyses indicated that during 1991–2017, a significant linear decrease (81.6%–60.4%) occurred in the overall prevalence of having ever drunk alcohol. A significant quadratic trend also was identified. The prevalence of having ever drunk alcohol decreased during 1991–2007 (81.6%–75.0%) and then decreased more rapidly during 2007–2017 (75.0%–60.4%). The prevalence of having ever drunk alcohol did not change significantly from 2015 (63.2%) to 2017 (60.4%).

Analyses of state and large urban school district data indicated that across 29 states, the overall prevalence of having ever drunk alcohol ranged from 30.4% to 68.0% across state surveys (median: 58.7%) (Supplementary Table 95). Across 19 large urban school districts, the prevalence ranged from 38.2% to 64.8% (median: 55.4%).

#### Drank Alcohol Before Age 13 Years

Nationwide, 15.5% of students had their first drink of alcohol (other than a few sips) before age 13 years (Supplementary Table 96). The prevalence of having drunk alcohol for the first time before age 13 years was higher among male (18.2%) than female (12.8%) students; higher among white male (17.1%) and Hispanic male (22.5%) than white female (10.9%) and Hispanic female (15.9%) students, respectively; and higher among 9th-grade male (20.3%), 10th-grade male (18.1%), 11th-grade male (17.4%), and 12th-grade male (16.2%) than 9th-grade female (16.0%), 10th-grade female (12.8%), 11th-grade female (12.3%), and 12th-grade female (9.3%) students, respectively. The prevalence of having drunk alcohol for the first time before age 13 years was higher among Hispanic (19.3%) than white (14.0%) and black (14.9%) students, higher among Hispanic female (15.9%) than white female (10.9%) students, and higher among Hispanic male (22.5%) than white male (17.1%) and black male (14.9%) students. The prevalence of having drunk alcohol for the first time before age 13 years was higher among 9th-grade (18.2%), 10th-grade (15.4%), and 11th-grade (14.9%) than 12th-grade (12.7%) students; higher among 9th-grade (18.2%) than 11th-grade (14.9%) students; higher among 9th-grade female (16.0%), 10th-grade female (12.8%), and 11th-grade female (12.3%) than 12th-grade female (9.3%) students; higher among 9th-grade female (16.0%) than 11th-grade female (12.3%) students; and higher among 9th-grade male (20.3%) than 12th-grade male (16.2%) students.

Analyses based on the question ascertaining sexual identity indicated that nationwide, 14.9% of heterosexual students; 21.6% of gay, lesbian, and bisexual students; and 20.0% of not sure students had drunk alcohol for the first time before age 13 years (Supplementary Table 96). The prevalence of having drunk alcohol for the first time before age 13 years was higher among gay, lesbian, and bisexual (21.6%) than heterosexual (14.9%) students. Among female students, the prevalence was higher among lesbian and bisexual (20.2%) than heterosexual (11.5%) students. The prevalence also was higher among heterosexual male (17.7%) than heterosexual female (11.5%) students.

Analyses based on the question ascertaining the sex of sexual contacts indicated that nationwide, 20.8% of students who had sexual contact with only the opposite sex, 28.2% of students who had sexual contact with only the same sex or with both sexes, and 9.1% of students who had no sexual contact had drunk alcohol for the first time before age 13 years (Supplementary Table 96). The prevalence of having drunk alcohol for the first time before age 13 years was higher among students who had sexual contact with only the opposite sex (20.8%) and students who had sexual contact with only the same sex or with both sexes (28.2%) than students who had no sexual contact (9.1%) and higher among students who had sexual contact with only the same sex or with both sexes (28.2%) than students who had sexual contact with only the opposite sex (20.8%). Among female students, the prevalence was higher among those who had sexual contact with only males (15.9%) and those who had sexual contact with only females or with both sexes (26.9%) than those who had no sexual contact (7.9%) and higher among those who had sexual contact with only females or with both sexes (26.9%) than those who had sexual contact with only males (15.9%). Among male students, the prevalence was higher among those who had sexual contact with only females (25.0%) and those who had sexual contact with only males or with both sexes (32.3%) than those who had no sexual contact (10.4%). The prevalence also was higher among male students who had sexual contact with only females (25.0%) than female students who had sexual contact with only males (15.9%), and higher among male students who had no sexual contact (10.4%) than female students who had no sexual contact (7.9%).

Trend analyses indicated that during 1991–2017, a significant linear decrease (32.7%–15.5%) occurred in the overall prevalence of having drunk alcohol for the first time before age 13 years. A significant quadratic trend also was identified. The prevalence of having drunk alcohol for the first time before age 13 years did not change significantly during 1991–1999 (32.7%–32.2%) and then decreased during 1999–2017 (32.2%–15.5%). The prevalence of having drunk alcohol for the first time before age 13 years did not change significantly from 2015 (17.2%) to 2017 (15.5%).

Analyses of state and large urban school district data indicated that across 38 states, the overall prevalence of having drunk alcohol for the first time before age 13 years ranged from 7.8% to 22.5% across state surveys (median: 15.8%) (Supplementary Table 97). Across 20 large urban school districts, the prevalence ranged from 13.8% to 21.2% (median: 16.9%).

#### Current Alcohol Use

Nationwide, 29.8% of students had had at least one drink of alcohol on at least 1 day during the 30 days before the survey (i.e., current alcohol use) (Supplementary Table 98). The prevalence of current alcohol use was higher among female (31.8%) than male (27.6%) students; higher among black female (24.3%) and Hispanic female (35.9%) than black male (16.9%) and Hispanic male (26.8%) students, respectively; and higher among 9th-grade female (22.0%) and 11th-grade female (36.8%) than 9th-grade male (15.3%) and 11th-grade male (31.6%) students, respectively. The prevalence of current alcohol use was higher among white (32.4%) and Hispanic (31.3%) than black (20.8%) students, higher among white female (33.2%) and Hispanic female (35.9%) than black female (24.3%) students, higher among white male (31.6%) and Hispanic male (26.8%) than black male (16.9%) students, and higher among white male (31.6%) than Hispanic male (26.8%) students. The prevalence of current alcohol use was higher among 10th-grade (27.0%), 11th-grade (34.4%), and 12th-grade (40.8%) than 9th-grade (18.8%) students; higher among 11th-grade (34.4%) and 12th-grade (40.8%) than 10th-grade (27.0%) students; higher among 12th-grade (40.8%) than 11th-grade (34.4%) students; higher among 10th-grade female (28.7%), 11th-grade female (36.8%), and 12th-grade female (41.2%) than 9th-grade female (22.0%) students; higher among 11th-grade female (36.8%) and 12th-grade female (41.2%) than 10th-grade female (28.7%) students; higher among 12th-grade female (41.2%) than 11th-grade female (36.8%) students; higher among 10th-grade male (25.3%), 11th-grade male (31.6%), and 12th-grade male (40.5%) than 9th-grade male (15.3%) students; higher among 11th-grade male (31.6%) and 12th-grade male (40.5%) than 10th-grade male (25.3%) students; and higher among 12th-grade male (40.5%) than 11th-grade male (31.6%) students.

Analyses based on the question ascertaining sexual identity indicated that nationwide, the prevalence of current alcohol use was 29.7% among heterosexual students; 37.4% among gay, lesbian, and bisexual students; and 21.5% among not sure students (Supplementary Table 98). The prevalence of current alcohol use was higher among heterosexual (29.7%) and gay, lesbian, and bisexual (37.4%) than not sure (21.5%) students and higher among gay, lesbian, and bisexual (37.4%) than heterosexual (29.7%) students. Among female students, the prevalence was higher among heterosexual (32.2%) and lesbian and bisexual (39.9%) than not sure (20.6%) students and higher among lesbian and bisexual (39.9%) than heterosexual (32.2%) students. The prevalence also was higher among heterosexual female (32.2%) than heterosexual male (27.7%) students and higher among lesbian and bisexual female (39.9%) than gay and bisexual male (29.5%) students.

Analyses based on the question ascertaining the sex of sexual contacts indicated that nationwide, the prevalence of current alcohol use was 47.5% among students who had sexual contact with only the opposite sex, 52.5% among students who had sexual contact with only the same sex or with both sexes, and 13.1% among students who had no sexual contact (Supplementary Table 98). The prevalence of current alcohol use was higher among students who had sexual contact with only the opposite sex (47.5%) and students who had sexual contact with only the same sex or with both sexes (52.5%) than students who had no sexual contact (13.1%). Among female students, the prevalence was higher among those who had sexual contact with only males (50.2%) and those who had sexual contact with only females or with both sexes (55.5%) than those who had no sexual contact (15.9%). Among male students, the prevalence was higher among those who had sexual contact with only females (45.2%) and those who had sexual contact with only males or with both sexes (43.9%) than those who had no sexual contact (10.2%). The prevalence also was higher among female students who had sexual contact with only males (50.2%) than male students who had sexual contact with only females (45.2%), higher among female students who had sexual contact with only females or with both sexes (55.5%) than male students who had sexual contact with only males or with both sexes (43.9%), and higher among female students who had no sexual contact (15.9%) than male students who had no sexual contact (10.2%).

Trend analyses indicated that during 1991–2017, a significant linear decrease (50.8%–29.8%) occurred in the overall prevalence of current alcohol use. A significant quadratic trend also was identified. The prevalence of current alcohol use decreased during 1991–2007 (50.8%–44.7%) and then decreased more rapidly during 2007–2017 (44.7%–29.8%). The prevalence of current alcohol use did not change significantly from 2015 (32.8%) to 2017 (29.8%).

Analyses of state and large urban school district data indicated that across 39 states, the overall prevalence of current alcohol use ranged from 10.6% to 34.0% across state surveys (median: 27.1%) (Supplementary Table 99). Across 21 large urban school districts, the prevalence ranged from 16.8% to 32.5% (median: 22.9%).

#### Usually Got Alcohol by Someone Giving It to Them

Among the 29.8% of students nationwide who currently drank alcohol, 43.5% had usually gotten the alcohol they drank by someone giving it to them during the 30 days before the survey (Supplementary Table 100). The prevalence of having usually gotten the alcohol they drank by someone giving it to them was higher among female (48.4%) than male (37.8%) students; higher among white female (49.4%) and Hispanic female (47.9%) than white male (38.6%) and Hispanic male (36.1%) students, respectively; and higher among 9th-grade female (52.4%) and 12th-grade female (48.4%) than 9th-grade male (38.0%) and 12th-grade male (35.3%) students, respectively.

Analyses based on the question ascertaining sexual identity indicated that nationwide, among the students who currently drank alcohol, 44.2% of heterosexual students; 42.6% of gay, lesbian, and bisexual students; and 29.5% of not sure students had usually gotten the alcohol they drank by someone giving it to them (Supplementary Table 100). The prevalence of having usually gotten the alcohol they drank by someone giving it to them was higher among heterosexual (44.2%) than not sure (29.5%) students. Among female students, the prevalence was higher among heterosexual (50.8%) than not sure (28.2%) students. The prevalence also was higher among heterosexual female (50.8%) than heterosexual male (37.6%) students.

Analyses based on the question ascertaining the sex of sexual contacts indicated that nationwide, among the students who currently drank alcohol, 43.0% of students who had sexual contact with only the opposite sex, 36.6% of students who had sexual contact with only the same sex or with both sexes, and 51.4% of students who had no sexual contact had usually gotten the alcohol they drank by someone giving it to them (Supplementary Table 100). The prevalence of having usually gotten the alcohol they drank by someone giving it to them was higher among students who had no sexual contact (51.4%) than students who had sexual contact with only the opposite sex (43.0%) and students who had sexual contact with only the same sex or with both sexes (36.6%). Among female students, the prevalence was higher among those who had sexual contact with only males (50.4%) and those who had no sexual contact (51.8%) than those who had sexual contact with only females or with both sexes (37.3%). Among male students, the prevalence was higher among those who had no sexual contact (50.9%) than those who had sexual contact with only females (36.1%). The prevalence also was higher among female students who had sexual contact with only males (50.4%) than male students who had sexual contact with only females (36.1%).

Trend analyses did not identify a significant linear trend in the overall prevalence of having usually gotten the alcohol they drank by someone giving it to them, among the students who currently drank alcohol, during 2007–2017 (41.7%–43.5%). A significant quadratic trend also was not identified. The prevalence of having usually gotten the alcohol they drank by someone giving it to them did not change significantly from 2015 (44.1%) to 2017 (43.5%).

Analyses of state and large urban school district data indicated that across 31 states, the overall prevalence of having usually gotten the alcohol they drank by someone giving it to them, among the students who currently drank alcohol, ranged from 31.7% to 46.6% across state surveys (median: 40.1%) (Supplementary Table 101). Across 15 large urban school districts, the prevalence ranged from 26.9% to 45.7% (median: 40.0%).

#### Current Binge Drinking

Nationwide, 13.5% of students had had four or more drinks of alcohol in a row (if they were female) or five or more drinks of alcohol in a row (if they were male) within a couple of hours on at least 1 day during the 30 days before the survey (i.e., current binge drinking) (Supplementary Table 102). The prevalence of current binge drinking was higher among black female (6.8%) and Hispanic female (16.0%) than black male (4.1%) and Hispanic male (12.0%) students, respectively and higher among 9th-grade female (9.2%) and 10th-grade female (12.6%) than 9th-grade male (5.3%) and 10th-grade male (10.1%) students, respectively. The prevalence of current binge drinking was higher among white (15.7%) and Hispanic (14.0%) than black (5.6%) students, higher among white female (15.9%) and Hispanic female (16.0%) than black female (6.8%) students, higher among white male (15.5%) and Hispanic male (12.0%) than black male (4.1%) students, and higher among white male (15.5%) than Hispanic male (12.0%) students. The prevalence of current binge drinking was higher among 10th-grade (11.4%), 11th-grade (15.4%), and 12th-grade (20.9%) than 9th-grade (7.3%) students; higher among 11th-grade (15.4%) and 12th-grade (20.9%) than 10th-grade (11.4%) students; higher among 12th-grade (20.9%) than 11th-grade (15.4%) students; higher among 10th-grade female (12.6%), 11th-grade female (15.4%), and 12th-grade female (20.1%) than 9th-grade female (9.2%) students; higher among 12th-grade female (20.1%) than 10th-grade female (12.6%) and 11th-grade female (15.4%) students; higher among 10th-grade male (10.1%), 11th-grade male (15.4%), and 12th-grade male (21.9%) than 9th-grade male (5.3%) students; higher among 11th-grade male (15.4%) and 12th-grade male (21.9%) than 10th-grade male (10.1%) students; and higher among 12th-grade male (21.9%) than 11th-grade male (15.4%) students.

Analyses based on the question ascertaining sexual identity indicated that nationwide, the prevalence of current binge drinking was 13.2% among heterosexual students; 17.2% among gay, lesbian, and bisexual students; and 10.8% among not sure students (Supplementary Table 102). The prevalence of current binge drinking was higher among gay, lesbian, and bisexual (17.2%) than heterosexual (13.2%) and not sure (10.8%) students. Among female students, the prevalence was higher among lesbian and bisexual (18.3%) than heterosexual (13.9) and not sure (10.1%) students.

Analyses based on the question ascertaining the sex of sexual contacts indicated that nationwide, the prevalence of current binge drinking was 23.1% among students who had sexual contact with only the opposite sex, 25.2% among students who had sexual contact with only the same sex or with both sexes, and 4.0% among students who had no sexual contact (Supplementary Table 102). The prevalence of current binge drinking was higher among students who had sexual contact with only the opposite sex (23.1%) and students who had sexual contact with only the same sex or with both sexes (25.2%) than students who had no sexual contact (4.0%). Among female students, the prevalence was higher among those who had sexual contact with only males (23.8%) and those who had sexual contact with only females or with both sexes (26.7%) than those who had no sexual contact (5.1%). Among male students, the prevalence was higher among those who had sexual contact with only females (22.5%) and those who had sexual contact with only males or with both sexes (21.1%) than those who had no sexual contact (2.8%). The prevalence also was higher among female students who had no sexual contact (5.1%) than male students who had no sexual contact (2.8%).

The question measuring the prevalence of current binge drinking using different criteria for male and female students was used for the first time in the 2017 national YRBS. As a result, long-term temporal trends and 2-year temporal changes are not available for this variable.

Analyses of state and large urban school district data indicated that across 36 states, the overall prevalence of current binge drinking ranged from 4.8% to 17.9% across state surveys (median: 13.1%) (Supplementary Table 103). Across 20 large urban school districts, the prevalence ranged from 4.1% to 13.1% (median: 8.3%).

#### Largest Number of Alcoholic Drinks in a Row Was 10 or More

Nationwide, 4.4% of students had reported 10 or more as the largest number of alcoholic drinks they had had in a row, within a couple of hours, during the 30 days before the survey (Supplementary Table 104). The prevalence of having reported 10 or more as the largest number of drinks in a row was higher among male (5.8%) than female (2.9%) students; higher among white male (7.0%) and Hispanic male (5.7%) than white female (2.9%) and Hispanic female (3.7%) students, respectively; and higher among 10th-grade male (5.1%), 11th-grade male (6.6%), and 12th-grade male (10.1%) than 10th-grade female (2.1%), 11th-grade female (3.5%), and 12th-grade female (4.6%) students, respectively. The prevalence of having reported 10 or more as the largest number of drinks in a row was higher among white (4.9%) and Hispanic (4.7%) than black (1.4%) students, higher among white female (2.9%) and Hispanic female (3.7%) than black female (1.0%) students, and higher among white male (7.0%) and Hispanic male (5.7%) than black male (1.5%) students. The prevalence of having reported 10 or more as the largest number of drinks in a row was higher among 10th-grade (3.6%), 11th-grade (5.0%), and 12th-grade (7.3%) than 9th-grade (1.9%) students; higher among 12th-grade (7.3%) than 10th-grade (3.6%) students and 11th-grade (5.0%) students; higher among 11th-grade female (3.5%) and 12th-grade female (4.6%) than 9th-grade female (1.8%) students; higher among 12th-grade female (4.6%) than 10th-grade female (2.1%) students; higher among 10th-grade male (5.1%), 11th-grade male (6.6%), and 12th-grade male (10.1%) than 9th-grade male (2.1%) students; and higher among 12th-grade male (10.1%) than 10th-grade male (5.1%) and 11th-grade male (6.6%) students.

Analyses based on the question ascertaining sexual identity indicated that nationwide, 4.3% of heterosexual students; 4.8% of gay, lesbian, and bisexual students; and 6.1% of not sure students had reported 10 or more as the largest number of drinks in a row (Supplementary Table 104). The prevalence of having reported 10 or more as the largest number of drinks in a row was higher among heterosexual male (5.7%) than heterosexual female (2.7%) students.

Analyses based on the question ascertaining the sex of sexual contacts indicated that nationwide, 7.9% of students who had sexual contact with only the opposite sex, 10.1% of students who had sexual contact with only the same sex or with both sexes, and 0.8% of students who had no sexual contact had reported 10 or more as the largest number of drinks in a row (Supplementary Table 104). The prevalence of having reported 10 or more as the largest number of drinks in a row was higher among students who had sexual contact with only the opposite sex (7.9%) and students who had sexual contact with only the same sex or with both sexes (10.1%) than students who had no sexual contact (0.8%). Among female students, the prevalence was higher among those who had sexual contact with only males (4.8%) and those who had sexual contact with only females or with both sexes (9.0%) than those who had no sexual contact (0.8%) and higher among those who had sexual contact with only females or with both sexes (9.0%) than those who had sexual contact with only males (4.8%). Among male students, the prevalence was higher among those who had sexual contact with only females (10.5%) and those who had sexual contact with only males or with both sexes (13.5%) than those who had no sexual contact (0.8%). The prevalence also was higher among male students who had sexual contact with only females (10.5%) than female students who had sexual contact with only males (4.8%).

Trend analyses indicated that during 2013–2017, a significant linear decrease (6.1%–4.4%) occurred in the overall prevalence of having reported 10 or more as the largest number of drinks in a row. Not enough data points were available to identify a quadratic trend. The prevalence of having reported 10 or more as the largest number of drinks in a row did not change significantly from 2015 (4.3%) to 2017 (4.4%).

Analyses of state and large urban school district data indicated that across 21 states, the overall prevalence of having reported 10 or more as the largest number of drinks in a row ranged from 1.9% to 6.9% across state surveys (median: 4.1%) (Supplementary Table 105). Across 15 large urban school districts, the prevalence ranged from 1.1% to 3.2% (median: 2.1%).

#### Ever Used Marijuana

Nationwide, 35.6% of students had used marijuana (also called grass, pot, or weed) one or more times during their life (Supplementary Table 106). The prevalence of having ever used marijuana was higher among black (42.8%) and Hispanic (42.4%) than white (32.0%) students, higher among black female (44.9%) and Hispanic female (42.7%) than white female (32.1%) students, and higher among black male (40.5%) and Hispanic male (42.1%) than white male (31.7%) students. The prevalence of having ever used marijuana was higher among 10th-grade (33.3%), 11th-grade (41.4%), and 12th-grade (45.8%) than 9th-grade (23.8%) students; higher among 11th-grade (41.4%) and 12th-grade (45.8%) than 10th-grade (33.3%) students; higher among 12th-grade (45.8%) than 11th-grade (41.4%) students; higher among 10th-grade female (33.6%), 11th-grade female (42.3%), and 12th-grade female (45.3%) than 9th-grade female (24.1%) students; higher among 11th-grade female (42.3%) and 12th-grade female (45.3%) than 10th-grade female (33.6%) students; higher among 10th-grade male (33.1%), 11th-grade male (40.3%), and 12th-grade male (46.2%) than 9th-grade male (23.4%) students; higher among 11th-grade male (40.3%) and 12th-grade male (46.2%) than 10th-grade male (33.1%) students; and higher among 12th-grade male (46.2%) than 11th-grade male (40.3%) students.

Analyses based on the question ascertaining sexual identity indicated that nationwide, 35.2% of heterosexual students; 50.4% of gay, lesbian, and bisexual students; and 28.8% of not sure students had ever used marijuana (Supplementary Table 106). The prevalence of having ever used marijuana was higher among heterosexual (35.2%) and gay, lesbian, and bisexual (50.4%) than not sure (28.8%) students and higher among gay, lesbian, and bisexual (50.4%) than heterosexual (35.2%) students. Among female students, the prevalence was higher among lesbian and bisexual (54.3%) than heterosexual (34.7%) and not sure (29.9%) students. Among male students, the prevalence was higher among heterosexual (35.7%) and gay and bisexual (38.5%) than not sure (24.9%) students. The prevalence also was higher among lesbian and bisexual female (54.3%) than gay and bisexual male (38.5%) students.

Analyses based on the question ascertaining the sex of sexual contacts indicated that nationwide, 55.5% of students who had sexual contact with only the opposite sex, 67.5% of students who had sexual contact with only the same sex or with both sexes, and 15.0% of students who had no sexual contact had ever used marijuana (Supplementary Table 106). The prevalence of having ever used marijuana was higher among students who had sexual contact with only the opposite sex (55.5%) and students who had sexual contact with only the same sex or with both sexes (67.5%) than students who had no sexual contact (15.0%) and higher among students who had sexual contact with only the same sex or with both sexes (67.5%) than students who had sexual contact with only the opposite sex (55.5%). Among female students, the prevalence was higher among those who had sexual contact with only males (54.6%) and those who had sexual contact with only females or with both sexes (71.6%) than those who had no sexual contact (16.6%) and higher among those who had sexual contact with only females or with both sexes (71.6%) than those who had sexual contact with only males (54.6%). Among male students, the prevalence was higher among those who had sexual contact with only females (56.3%) and those who had sexual contact with only males or with both sexes (55.5%) than those who had no sexual contact (13.3%). The prevalence also was higher among female students who had sexual contact with only females or with both sexes (71.6%) than male students who had sexual contact with only males or with both sexes (55.5%).

Trend analyses did not identify a significant linear trend in the overall prevalence of having ever used marijuana during 1991–2017 (31.3%–35.6%). A significant quadratic trend was identified. The prevalence of having ever used marijuana increased during 1991–1997 (31.3%–47.1%) and then decreased during 1997–2017 (47.1%–35.6%). The prevalence of having ever used marijuana did not change significantly from 2015 (38.6%) to 2017 (35.6%).

Analyses of state and large urban school district data indicated that across 30 states, the overall prevalence of having ever used marijuana ranged from 16.6% to 44.1% across state surveys (median: 34.4%) (Supplementary Table 107). Across 16 large urban school districts, the prevalence ranged from 25.6% to 46.9% (median: 36.4%).

#### Tried Marijuana Before Age 13 Years

Nationwide, 6.8% of students had tried marijuana (also called grass, pot, or weed) for the first time before age 13 years (Supplementary Table 108). The prevalence of having tried marijuana for the first time before age 13 years was higher among male (8.3%) than female (5.3%) students; higher among black male (12.8%) and Hispanic male (12.1%) than black female (6.8%) and Hispanic female (7.5%) students, respectively; and higher among 9th-grade male (8.0%), 10th-grade male (8.6%), 11th-grade male (8.2%), and 12th-grade male (8.4%) than 9th-grade female (6.0%), 10th-grade female (5.0%), 11th-grade female (5.1%), and 12th-grade female (4.8%) students, respectively. The prevalence of having tried marijuana for the first time before age 13 years was higher among black (9.8%) and Hispanic (9.8%) than white (4.7%) students, higher among Hispanic female (7.5%) than white female (4.0%) students, and higher among black male (12.8%) and Hispanic male (12.1%) than white male (5.5%) students.

Analyses based on the question ascertaining sexual identity indicated that nationwide, 6.3% of heterosexual students; 11.1% of gay, lesbian, and bisexual students; and 8.7% of not sure students had tried marijuana for the first time before age 13 years (Supplementary Table 108). The prevalence of having tried marijuana for the first time before age 13 years was higher among gay, lesbian, and bisexual (11.1%) than heterosexual (6.3%) students. Among female students, the prevalence was higher among lesbian and bisexual (10.7%) than heterosexual (4.3%) students. The prevalence also was higher among heterosexual male (8.2%) than heterosexual female (4.3%) students.

Analyses based on the question ascertaining the sex of sexual contacts indicated that nationwide, 10.6% of students who had sexual contact with only the opposite sex, 18.0% of students who had sexual contact with only the same sex or with both sexes, and 1.7% of students who had no sexual contact had tried marijuana for the first time before age 13 years (Supplementary Table 108). The prevalence of having tried marijuana for the first time before age 13 years was higher among students who had sexual contact with only the opposite sex (10.6%) and students who had sexual contact with only the same sex or with both sexes (18.0%) than students who had no sexual contact (1.7%) and higher among students who had sexual contact with only the same sex or with both sexes (18.0%) than students who had sexual contact with only the opposite sex (10.6%). Among female students, the prevalence was higher among those who had sexual contact with only males (6.9%) and those who had sexual contact with only females or with both sexes (17.7%) than those who had no sexual contact (1.4%) and higher among those who had sexual contact with only females or with both sexes (17.7%) than those who had sexual contact with only males (6.9%). Among male students, the prevalence was higher among those who had sexual contact with only females (13.6%) and those who had sexual contact with only males or with both sexes (18.8%) than those who had no sexual contact (2.0%). The prevalence also was higher among male students who had sexual contact with only females (13.6%) than female students who had sexual contact with only males (6.9%).

Trend analyses indicated that during 1991–2017, a significant linear decrease (7.4%–6.8%) occurred in the overall prevalence of having tried marijuana for the first time before age 13 years. A significant quadratic trend was identified. The prevalence of having tried marijuana for the first time before age 13 years increased during 1991–1999 (7.4%–11.3%) and then decreased during 1999–2017 (11.3%–6.8%). The prevalence of having tried marijuana before age 13 years did not change significantly from 2015 (7.5%) to 2017 (6.8%).

Analyses of state and large urban school district data indicated that across 38 states, the overall prevalence of having tried marijuana for the first time before age 13 years ranged from 4.1% to 15.7% across state surveys (median: 6.7%) (Supplementary Table 109). Across 19 large urban school districts, the prevalence ranged from 5.7% to 15.9% (median: 8.1%).

#### Current Marijuana Use

Nationwide, 19.8% of students had used marijuana (also called grass, pot, or weed) one or more times during the 30 days before the survey (i.e., current marijuana use) (Supplementary Table 110). The prevalence of current marijuana use was higher among black (25.3%) and Hispanic (23.4%) than white (17.7%) students, higher among black female (25.0%) and Hispanic female (23.8%) than white female (17.2%) students, and higher among black male (25.4%) and Hispanic male (23.1%) than white male (18.1%) students. The prevalence of current marijuana use was higher among 10th-grade (18.7%), 11th-grade (22.6%), and 12th-grade (25.7%) than 9th-grade (13.1%) students; higher among 11th-grade (22.6%) and 12th-grade (25.7%) than 10th-grade (18.7%) students; higher among 12th-grade (25.7%) than 11th-grade (22.6%) students; higher among 10th-grade female (18.7%), 11th-grade female (23.3%), and 12th-grade female (23.8%) than 9th-grade female (13.3%) students; higher among 11th-grade female (23.3%) and 12th-grade female (23.8%) than 10th-grade female (18.7%) students; higher among 10th-grade male (18.7%), 11th-grade male (21.7%), and 12th-grade male (27.8%) than 9th-grade male (13.0%) students, and higher among 12th-grade male (27.8%) than 10th-grade male (18.7%) and 11th-grade male (21.7%) students.

Analyses based on the question ascertaining sexual identity indicated that nationwide, the prevalence of current marijuana use was 19.1% among heterosexual students; 30.6% among gay, lesbian, and bisexual students; and 18.9% among not sure students (Supplementary Table 110). The prevalence of current marijuana use was higher among gay, lesbian, and bisexual (30.6%) than heterosexual (19.1%) and not sure (18.9%) students. Among female students, the prevalence was higher among lesbian and bisexual (32.8%) than heterosexual (18.1%) and not sure (19.3%) students.

Analyses based on the question ascertaining the sex of sexual contacts indicated that nationwide, the prevalence of current marijuana use was 31.9% among students who had sexual contact with only the opposite sex, 43.3% among students who had sexual contact with only the same sex or with both sexes, and 6.4% among students who had no sexual contact (Supplementary Table 110). The prevalence of current marijuana use was higher among students who had sexual contact with only the opposite sex (31.9%) and students who had sexual contact with only the same sex or with both sexes (43.3%) than students who had no sexual contact (6.4%) and higher among students who had sexual contact with only the same sex or with both sexes (43.3%) than students who had sexual contact with only the opposite sex (31.9%). Among female students, the prevalence was higher among those who had sexual contact with only males (30.0%) and those who had sexual contact with only females or with both sexes (45.4%) than those who had no sexual contact (7.6%) and higher among those who had sexual contact with only females or with both sexes (45.4%) than those who had sexual contact with only males (30.0%). Among male students, the prevalence was higher among those who had sexual contact with only females (33.4%) and those who had sexual contact with only males or with both sexes (37.3%) than those who had no sexual contact (5.1%). The prevalence also was higher among female students who had no sexual contact (7.6%) than male students who had no sexual contact (5.1%).

Trend analyses did not identify a significant linear trend in the overall prevalence of current marijuana use during 1991–2017 (14.7%–19.8%). A significant quadratic trend was identified. The prevalence of current marijuana use increased during 1991–1995 (14.7%–25.3%) and then decreased during 1995–2017 (25.3%–19.8%). The prevalence of current marijuana use did not change significantly from 2015 (21.7%) to 2017 (19.8%).

Analyses of state and large urban school district data indicated that across 39 states, the overall prevalence of current marijuana use ranged from 8.1% to 27.3% across state surveys (median: 18.6%) (Supplementary Table 111). Across 21 large urban school districts, the prevalence ranged from 15.5% to 33.0% (median: 20.9%).

#### Ever Used Synthetic Marijuana

Nationwide, 6.9% of students had used synthetic marijuana (also called “K2,” “Spice,” “fake weed,” “King Kong,” “Yucatan Fire,” “Skunk,” or “Moon Rocks”) one or more times during their life (Supplementary Table 112). The prevalence of having ever used synthetic marijuana was higher among black male (8.4%) than black female (4.2%) students. The prevalence of having ever used synthetic marijuana was higher among Hispanic (9.1%) than white (5.9%) and black (6.3%) students, higher among Hispanic female (8.9%) than black female (4.2%) students, and higher among Hispanic male (9.3%) than white male (5.9%) students. The prevalence of having ever used synthetic marijuana was higher among 12th-grade (7.6%) than 9th-grade (5.5%) students and higher among 12th-grade male (8.6%) than 9th-grade male (5.4%) students.

Analyses based on the question ascertaining sexual identity indicated that nationwide, 6.0% of heterosexual students; 12.7% of gay, lesbian, and bisexual students; and 11.1% of not sure students had ever used synthetic marijuana (Supplementary Table 112). The prevalence of having ever used synthetic marijuana was higher among gay, lesbian, and bisexual (12.7%) and not sure (11.1%) than heterosexual (6.0%) students. Among female students, the prevalence was higher among lesbian and bisexual (11.8%) than heterosexual (5.4%) and not sure (7.2%) students. Among male students, the prevalence was higher among gay and bisexual (14.4%) and not sure (15.4%) than heterosexual (6.6%) students. The prevalence also was higher among heterosexual male (6.6%) than heterosexual female (5.4%) students and higher among not sure male (15.4%) than not sure female (7.2%) students.

Analyses based on the question ascertaining the sex of sexual contacts indicated that nationwide, 10.2% of students who had sexual contact with only the opposite sex, 19.1% of students who had sexual contact with only the same sex or with both sexes, and 1.7% of students who had no sexual contact had ever used synthetic marijuana (Supplementary Table 112). The prevalence of having ever used synthetic marijuana was higher among students who had sexual contact with only the opposite sex (10.2%) and students who had sexual contact with only the same sex or with both sexes (19.1%) than students who had no sexual contact (1.7%) and higher among students who had sexual contact with only the same sex or with both sexes (19.1%) than students who had sexual contact with only the opposite sex (10.2%). Among female students, the prevalence was higher among those who had sexual contact with only males (8.5%) and those who had sexual contact with only females or with both sexes (19.6%) than those who had no sexual contact (1.9%) and higher among those who had sexual contact with only females or with both sexes (19.6%) than those who had sexual contact with only males (8.5%). Among male students, the prevalence was higher among those who had sexual contact with only females (11.5%) and those who had sexual contact with only males or with both sexes (17.5%) than those who had no sexual contact (1.5%). The prevalence also was higher among male students who had sexual contact with only females (11.5%) than female students who had sexual contact with only males (8.5%).

The question measuring the prevalence of having ever used synthetic marijuana was used for the first time in the 2015 national YRBS. As a result, long-term temporal trends are not available for this variable. The prevalence of having ever used synthetic marijuana decreased from 2015 (9.2%) to 2017 (6.9%).

Analyses of state and large urban school district data indicated that across 28 states, the overall prevalence of having ever used synthetic marijuana ranged from 4.8% to 17.3% across state surveys (median: 6.6%) (Supplementary Table 113). Across 17 large urban school districts, the prevalence ranged from 4.9% to 10.4% (median: 6.8%).

#### Ever Used Cocaine

Nationwide, 4.8% of students had used any form of cocaine (e.g., powder, crack,^††^ or freebase^§§^) one or more times during their life (Supplementary Table 114). The prevalence of having ever used cocaine was higher among male (6.1%) than female (3.5%) students; higher among white male (5.5%), black male (4.2%), and Hispanic male (8.1%) than white female (3.4%), black female (1.2%), and Hispanic female (4.6%) students, respectively; and higher among 9th-grade male (3.6%), 10th-grade male (5.5%), 11th-grade male (6.6%), and 12th-grade male (8.7%) than 9th-grade female (2.3%), 10th-grade female (2.3%), 11th-grade female (4.1%), and 12th-grade female (5.3%) students, respectively. The prevalence of having ever used cocaine was higher among white (4.4%) and Hispanic (6.3%) than black (2.8%) students, higher among Hispanic (6.3%) than white (4.4%) students, higher among white female (3.4%) and Hispanic female (4.6%) than black female (1.2%) students, and higher among Hispanic male (8.1%) than white male (5.5%) and black male (4.2%) students. The prevalence of having ever used cocaine was higher among 12th-grade (7.0%) than 9th-grade (2.9%), 10th-grade (3.9%), and 11th-grade (5.4%) students; higher among 11th-grade (5.4%) than 9th-grade (2.9%) students; higher among 11th-grade female (4.1%) and 12th-grade female (5.3%) than 9th-grade female (2.3%) and 10th-grade female (2.3%) students; higher among 11th-grade male (6.6%) and 12th-grade male (8.7%) than 9th-grade male (3.6%) students; and higher among 12th-grade male (8.7%) than 10th-grade male (5.5%) students.

Analyses based on the question ascertaining sexual identity indicated that nationwide, 4.2% of heterosexual students; 8.0% of gay, lesbian, and bisexual students; and 10.4% of not sure students had ever used cocaine (Supplementary Table 114). The prevalence of having ever used cocaine was higher among gay, lesbian, and bisexual (8.0%) and not sure (10.4%) than heterosexual (4.2%) students. Among female students, the prevalence was higher among lesbian and bisexual (5.6%) than heterosexual (3.0%) students. Among male students, the prevalence was higher among gay and bisexual (14.6%) and not sure (15.1%) than heterosexual (5.2%) students. The prevalence also was higher among heterosexual male (5.2%) than heterosexual female (3.0%) students, higher among gay and bisexual male (14.6%) than lesbian and bisexual female (5.6%) students, and higher among not sure male (15.1%) than not sure female (6.0%) students.

Analyses based on the question ascertaining the sex of sexual contacts indicated that nationwide, 7.1% of students who had sexual contact with only the opposite sex, 14.4% of students who had sexual contact with only the same sex or with both sexes, and 0.8% of students who had no sexual contact had ever used cocaine (Supplementary Table 114). The prevalence of having ever used cocaine was higher among students who had sexual contact with only the opposite sex (7.1%) and students who had sexual contact with only the same sex or with both sexes (14.4%) than students who had no sexual contact (0.8%) and higher among students who had sexual contact with only the same sex or with both sexes (14.4%) than students who had sexual contact with only the opposite sex (7.1%).

Among female students, the prevalence was higher among those who had sexual contact with only males (4.6%) and those who had sexual contact with only females or with both sexes (11.9%) than those who had no sexual contact (0.8%) and higher among those who had sexual contact with only females or with both sexes (11.9%) than those who had sexual contact with only males (4.6%). Among male students, the prevalence was higher among those who had sexual contact with only females (9.2%) and those who had sexual contact with only males or with both sexes (21.3%) than those who had no sexual contact (0.8%) and higher among those who had sexual contact with only males or with both sexes (21.3%) than those who had sexual contact with only females (9.2%). The prevalence also was higher among male students who had sexual contact with only females (9.2%) than female students who had sexual contact with only males (4.6%) and higher among male students who had sexual contact with only males or with both sexes (21.3%) than female students who had sexual contact with only females or with both sexes (11.9%).

Trend analyses indicated that during 1991–2017, a significant linear decrease (5.9%–4.8%) occurred in the overall prevalence of having ever used cocaine. A significant quadratic trend also was identified. The prevalence of having ever used cocaine increased during 1991–2001 (5.9%–9.4%) and then decreased during 2001–2017 (9.4%–4.8%). The prevalence of having ever used cocaine did not change significantly from 2015 (5.2%) to 2017 (4.8%).

Analyses of state and large urban school district data indicated that across 32 states, the overall prevalence of having ever used cocaine ranged from 2.9% to 9.9% across state surveys (median: 4.6%) (Supplementary Table 115). Across 20 large urban school districts, the prevalence ranged from 2.3% to 7.8% (median: 5.8%).

#### Ever Used Inhalants

Nationwide, 6.2% of students had sniffed glue, breathed the contents of aerosol spray cans, or inhaled any paints or sprays to get high one or more times during their life (Supplementary Table 116). The prevalence of having ever used inhalants was higher among 9th-grade female (9.0%) than 9th-grade male (5.6%) students and higher among 12th-grade male (5.8%) than 12th-grade female (4.1%) students. The prevalence of having ever used inhalants was higher among Hispanic (7.1%) than white (5.7%) students. The prevalence of having ever used inhalants was higher among 9th-grade (7.2%) than 10th-grade (5.7%) and 12th-grade (4.9%) students and higher among 9th-grade female (9.0%) than 10th-grade female (5.6%) and 12th-grade female (4.1%) students.

Analyses based on the question ascertaining sexual identity indicated that nationwide, 5.1% of heterosexual students; 10.7% of gay, lesbian, and bisexual students; and 18.3% of not sure students had ever used inhalants (Supplementary Table 116). The prevalence of having ever used inhalants was higher among gay, lesbian, and bisexual (10.7%) and not sure (18.3%) than heterosexual (5.1%) students and higher among not sure (18.3%) than gay, lesbian, and bisexual (10.7%) students. Among female students, the prevalence was higher among lesbian and bisexual (9.9%) and not sure (15.8%) than heterosexual (5.2%) students. Among male students, the prevalence was higher among gay and bisexual (13.2%) and not sure (20.4%) than heterosexual (5.0%) students.

Analyses based on the question ascertaining the sex of sexual contacts indicated that nationwide, 7.4% of students who had sexual contact with only the opposite sex, 18.6% of students who had sexual contact with only the same sex or with both sexes, and 2.9% of students who had no sexual contact had ever used inhalants (Supplementary Table 116). The prevalence of having ever used inhalants was higher among students who had sexual contact with only the opposite sex (7.4%) and students who had sexual contact with only the same sex or with both sexes (18.6%) than students who had no sexual contact (2.9%) and higher among students who had sexual contact with only the same sex or with both sexes (18.6%) than students who had sexual contact with only the opposite sex (7.4%). Among female students, the prevalence was higher among those who had sexual contact with only males (7.6%) and those who had sexual contact with only females or with both sexes (17.1%) than those who had no sexual contact (3.4%) and higher among those who had sexual contact with only females or with both sexes (17.1%) than those who had sexual contact with only males (7.6%). Among male students, the prevalence was higher among those who had sexual contact with only females (7.3%) and those who had sexual contact with only males or with both sexes (23.0%) than those who had no sexual contact (2.4%) and higher among those who had sexual contact with only males or with both sexes (23.0%) than those who had sexual contact with only females (7.3%). The prevalence also was higher among female students who had no sexual contact (3.4%) than male students who had no sexual contact (2.4%).

Trend analyses indicated that during 1995–2017, a significant linear decrease (20.3%–6.2%) occurred in the overall prevalence of having ever used inhalants. A significant quadratic trend also was identified. The prevalence of having ever used inhalants decreased during 1995–2011 (20.3%–11.4%) and then decreased more slowly during 2011–2017 (11.4%–6.2%). The prevalence of having ever used inhalants did not change significantly from 2015 (7.0%) to 2017 (6.2%).

Analyses of state and large urban school district data indicated that across 27 states, the overall prevalence of having ever used inhalants ranged from 5.5% to 12.6% across state surveys (median: 6.7%) (Supplementary Table 117). Across 17 large urban school districts, the prevalence ranged from 4.6% to 12.4% (median: 7.4%).

#### Ever Used Heroin

Nationwide, 1.7% of students had used heroin (also called “smack,” “junk,” or “China White”) one or more times during their life (Supplementary Table 118). The prevalence of having ever used heroin was higher among male (2.4%) than female (0.9%) students; higher among white male (1.8%), black male (2.9%), and Hispanic male (2.7%) than white female (0.4%), black female (1.3%), and Hispanic female (1.0%) students, respectively; and higher among 9th-grade male (2.2%), 11th-grade male (2.1%), and 12th-grade male (3.1%) than 9th-grade female (0.5%), 11th-grade female (0.8%), and 12th-grade female (1.4%) students, respectively. The prevalence of having ever used heroin was higher among black (2.2%) than white (1.1%) students, higher among Hispanic female (1.0%) than white female (0.4%) students, and higher among black male (2.9%) than white male (1.8%) students.

Analyses based on the question ascertaining sexual identity indicated that nationwide, 1.1% of heterosexual students; 3.5% of gay, lesbian, and bisexual students; and 7.7% of not sure students had ever used heroin (Supplementary Table 118). The prevalence of having ever used heroin was higher among gay, lesbian, and bisexual (3.5%) and not sure (7.7%) than heterosexual (1.1%) students. Among female students, the prevalence was higher among lesbian and bisexual (2.2%) than heterosexual (0.6%) students. Among male students, the prevalence was higher among gay and bisexual (7.4%) and not sure (13.2%) than heterosexual (1.6%) students. The prevalence also was higher among heterosexual male (1.6%) than heterosexual female (0.6%) students, higher among gay and bisexual male (7.4%) than lesbian and bisexual female (2.2%) students, and higher among not sure male (13.2%) than not sure female (2.8%) students.

Analyses based on the question ascertaining the sex of sexual contacts indicated that nationwide, 1.7% of students who had sexual contact with only the opposite sex, 6.6% of students who had sexual contact with only the same sex or with both sexes, and 0.3% of students who had no sexual contact had ever used heroin (Supplementary Table 118). The prevalence of having ever used heroin was higher among students who had sexual contact with only the opposite sex (1.7%) and students who had sexual contact with only the same sex or with both sexes (6.6%) than students who had no sexual contact (0.3%) and higher among students who had sexual contact with only the same sex or with both sexes (6.6%) than students who had sexual contact with only the opposite sex (1.7%). Among female students, the prevalence was higher among those who had sexual contact with only females or with both sexes (3.6%) than those who had sexual contact with only males (0.4%) and those who had no sexual contact (0.4%). Among male students, the prevalence was higher among those who had sexual contact with only females (2.8%) and those who had sexual contact with only males or with both sexes (15.4%) than those who had no sexual contact (0.2%) and higher among those who had sexual contact with only males or with both sexes (15.4%) than those who had sexual contact with only females (2.8%). The prevalence also was higher among male students who had sexual contact with only females (2.8%) than female students who had sexual contact with only males (0.4%) and higher among male students who had sexual contact with only males or with both sexes (15.4%) than female students who had sexual contact with only females or with both sexes (3.6%).

Trend analyses indicated that during 1999–2017, a significant linear decrease (2.4%–1.7%) occurred in the overall prevalence of having ever used heroin. A significant quadratic trend also was identified. The prevalence of having ever used heroin did not change significantly during 1999–2011 (2.4%–2.9%) and then decreased during 2011–2017 (2.9%–1.7%). The prevalence of having ever used heroin did not change significantly from 2015 (2.1%) to 2017 (1.7%).

Analyses of state and large urban school district data indicated that across 33 states, the overall prevalence of having ever used heroin ranged from 1.2% to 9.6% across state surveys (median: 2.3%) (Supplementary Table 119). Across 20 large urban school districts, the prevalence ranged from 1.3% to 7.6% (median: 3.8%).

#### Ever Used Methamphetamines

Nationwide, 2.5% of students had used methamphetamines (also called “speed,” “crystal,” “crank,” or “ice”) one or more times during their life (Supplementary Table 120). The prevalence of having ever used methamphetamines was higher among male (3.4%) than female (1.4%) students; higher among white male (2.9%), black male (3.5%), and Hispanic male (4.0%) than white female (1.0%), black female (1.5%), and Hispanic female (1.7%) students, respectively; and higher among 9th-grade male (2.5%), 10th-grade male (3.5%), 11th-grade male (3.2%), and 12th-grade male (4.3%) than 9th-grade female (1.2%), 10th-grade female (1.0%), 11th-grade female (1.3%), and 12th-grade female (2.0%) students, respectively. The prevalence of having ever used methamphetamines was higher among 12th-grade (3.2%) than 9th-grade (1.9%) students and higher among 12th-grade male (4.3%) than 9th-grade male (2.5%) students.

Analyses based on the question ascertaining sexual identity indicated that nationwide, 1.8% of heterosexual students; 6.1% of gay, lesbian, and bisexual students; and 7.6% of not sure students had ever used methamphetamines (Supplementary Table 120). The prevalence of having ever used methamphetamines was higher among gay, lesbian, and bisexual (6.1%) and not sure (7.6%) than heterosexual (1.8%) students. Among female students, the prevalence was higher among lesbian and bisexual (3.9%) than heterosexual (0.9%) students. Among male students, the prevalence was higher among gay and bisexual (12.4%) and not sure (12.6%) than heterosexual (2.5%) students. The prevalence also was higher among heterosexual male (2.5%) than heterosexual female (0.9%) students, higher among gay and bisexual male (12.4%) than lesbian and bisexual female (3.9%) students, and higher among not sure male (12.6%) than not sure female (2.9%) students.

Analyses based on the question ascertaining the sex of sexual contacts indicated that nationwide, 2.8% of students who had sexual contact with only the opposite sex, 8.7% of students who had sexual contact with only the same sex or with both sexes, and 0.6% of students who had no sexual contact had ever used methamphetamines (Supplementary Table 120). The prevalence of having ever used methamphetamines was higher among students who had sexual contact with only the opposite sex (2.8%) and students who had sexual contact with only the same sex or with both sexes (8.7%) than students who had no sexual contact (0.6%) and higher among students who had sexual contact with only the same sex or with both sexes (8.7%) than students who had sexual contact with only the opposite sex (2.8%). Among female students, the prevalence was higher among those who had sexual contact with only males (1.1%) and those who had sexual contact with only females or with both sexes (6.8%) than those who had no sexual contact (0.5%) and higher among those who had sexual contact with only females or with both sexes (6.8%) than those who had sexual contact with only males (1.1%). Among male students, the prevalence was higher among those who had sexual contact with only females (4.2%) and those who had sexual contact with only males or with both sexes (14.3%) than those who had no sexual contact (0.8%) and higher among those who had sexual contact with only males or with both sexes (14.3%) than those who had sexual contact with only females (4.2%). The prevalence also was higher among male students who had sexual contact with only females (4.2%) than female students who had sexual contact with only males (1.1%).

Trend analyses indicated that during 1999–2017, a significant linear decrease (9.1%–2.5%) occurred in the overall prevalence of having ever used methamphetamines. A significant quadratic trend was not identified. The prevalence of having ever used methamphetamines did not change significantly from 2015 (3.0%) to 2017 (2.5%).

Analyses of state and large urban school district data indicated that across 30 states, the overall prevalence of having ever used methamphetamines ranged from 1.7% to 10.5% across state surveys (median: 2.6%) (Supplementary Table 121). Across 17 large urban school districts, the prevalence ranged from 2.0% to 7.1% (median: 4.2%).

#### Ever Used Ecstasy

Nationwide, 4.0% of students had used ecstasy (also called “MDMA” [3,4-methylenedioxymethamphetamine]) one or more times during their life (Supplementary Table 122). The prevalence of having ever used ecstasy was higher among male (5.0%) than female (2.9%) students; higher among white male (4.1%), black male (4.1%), and Hispanic male (6.6%) than white female (2.8%), black female (1.7%), and Hispanic female (3.5%) students, respectively; and higher among 9th-grade male (3.5%), 10th-grade male (4.2%), and 11th-grade male (5.2%) than 9th-grade female (1.6%), 10th-grade female (1.7%), and 11th-grade female (3.4%) students, respectively. The prevalence of having ever used ecstasy was higher among Hispanic (5.1%) than white (3.4%) and black (3.0%) students, higher among Hispanic female (3.5%) than black female (1.7%) students, and higher among Hispanic male (6.6%) than white male (4.1%) and black male (4.1%) students. The prevalence of having ever used ecstasy was higher among 11th-grade (4.4%) and 12th-grade (6.0%) than 9th-grade (2.5%) and 10th-grade (2.9%) students, higher among 11th-grade female (3.4%) and 12th-grade female (5.1%) than 9th-grade female (1.6%) and 10th-grade female (1.7%) students, and higher among 12th-grade male (6.9%) than 9th-grade male (3.5%) students.

Analyses based on the question ascertaining sexual identity indicated that nationwide, 3.3% of heterosexual students; 8.8% of gay, lesbian, and bisexual students; and 8.1% of not sure students had ever used ecstasy (Supplementary Table 122). The prevalence of having ever used ecstasy was higher among gay, lesbian, and bisexual (8.8%) and not sure (8.1%) than heterosexual (3.3%) students. Among female students, the prevalence was higher among lesbian and bisexual (6.4%) than heterosexual (2.3%) students. Among male students, the prevalence was higher among gay and bisexual (15.0%) and not sure (11.2%) than heterosexual (4.2%) students. The prevalence also was higher among heterosexual male (4.2%) than heterosexual female (2.3%) students and higher among gay and bisexual male (15.0%) than lesbian and bisexual female (6.4%) students.

Analyses based on the question ascertaining the sex of sexual contacts indicated that nationwide, 5.7% of students who had sexual contact with only the opposite sex, 14.3% of students who had sexual contact with only the same sex or with both sexes, and 0.6% of students who had no sexual contact had ever used ecstasy (Supplementary Table 122). The prevalence of having ever used ecstasy was higher among students who had sexual contact with only the opposite sex (5.7%) and students who had sexual contact with only the same sex or with both sexes (14.3%) than students who had no sexual contact (0.6%) and higher among students who had sexual contact with only the same sex or with both sexes (14.3%) than students who had sexual contact with only the opposite sex (5.7%). Among female students, the prevalence was higher among those who had sexual contact with only males (3.7%) and those who had sexual contact with only females or with both sexes (12.7%) than those who had no sexual contact (0.4%) and higher among those who had sexual contact with only females or with both sexes (12.7%) than those who had sexual contact with only males (3.7%). Among male students, the prevalence was higher among those who had sexual contact with only females (7.4%) and those who had sexual contact with only males or with both sexes (19.0%) than those who had no sexual contact (0.8%) and higher among those who had sexual contact with only males or with both sexes (19.0%) than those who had sexual contact with only females (7.4%). The prevalence also was higher among male students who had sexual contact with only females (7.4%) than female students who had sexual contact with only males (3.7%).

Trend analyses indicated that during 2001–2017, a significant linear decrease (11.1%–4.0%) occurred in the overall prevalence of having ever used ecstasy. A significant quadratic trend was not identified. The prevalence of having ever used ecstasy decreased from 2015 (5.0%) to 2017 (4.0%).

Analyses of state and large urban school district data indicated that across 28 states, the overall prevalence of having ever used ecstasy ranged from 2.8% to 13.0% across state surveys (median: 4.1%) (Supplementary Table 123). Across 17 large urban school districts, the prevalence ranged from 1.9% to 7.9% (median: 5.1%).

#### Ever Used Hallucinogenic Drugs

Nationwide, 6.6% of students had used hallucinogenic drugs (e.g., LSD, acid, PCP, angel dust, mescaline, or mushrooms) one or more times during their life (Supplementary Table 124). The prevalence of having ever used hallucinogenic drugs was higher among male (7.6%) than female (5.5%) students; higher among black male (4.8%) and Hispanic male (8.2%) than black female (1.4%) and Hispanic female (5.8%) students, respectively; and higher among 10th-grade male (7.0%) than 10th-grade female (4.0%) students. The prevalence of having ever used hallucinogenic drugs was higher among white (7.2%) and Hispanic (7.1%) than black (3.3%) students, higher among white female (6.4%) and Hispanic female (5.8%) than black female (1.4%) students, and higher among white male (7.9%) and Hispanic male (8.2%) than black male (4.8%) students. The prevalence of having ever used hallucinogenic drugs was higher among 11th-grade (8.0%) and 12th-grade (9.2%) than 9th-grade (4.0%) and 10th-grade (5.4%) students; higher among 11th-grade female (7.0%) and 12th-grade female (7.6%) than 9th-grade female (3.7%) and 10th-grade female (4.0%) students; higher among 10th-grade male (7.0%), 11th-grade male (8.8%), and 12th-grade male (10.7%) than 9th-grade male (4.4%) students; and higher among 12th-grade male (10.7%) than 10th-grade male (7.0%) students.

Analyses based on the question ascertaining sexual identity indicated that nationwide, 5.7% of heterosexual students; 11.9% of gay, lesbian, and bisexual students; and 12.0% of not sure students had ever used hallucinogenic drugs (Supplementary Table 124). The prevalence of having ever used hallucinogenic drugs was higher among gay, lesbian, and bisexual (11.9%) and not sure (12.0%) than heterosexual (5.7%) students. Among female students, the prevalence was higher among lesbian and bisexual (10.9%) than heterosexual (4.3%) students. Among male students, the prevalence was higher among gay and bisexual (15.3%) than heterosexual (7.0%) students. The prevalence also was higher among heterosexual male (7.0%) than heterosexual female (4.3%) students.

Analyses based on the question ascertaining the sex of sexual contacts indicated that nationwide, 9.9% of students who had sexual contact with only the opposite sex, 20.8% of students who had sexual contact with only the same sex or with both sexes, and 1.3% of students who had no sexual contact had ever used hallucinogenic drugs (Supplementary Table 124). The prevalence of having ever used hallucinogenic drugs was higher among students who had sexual contact with only the opposite sex (9.9%) and students who had sexual contact with only the same sex or with both sexes (20.8%) than students who had no sexual contact (1.3%) and higher among students who had sexual contact with only the same sex or with both sexes (20.8%) than students who had sexual contact with only the opposite sex (9.9%). Among female students, the prevalence was higher among those who had sexual contact with only males (7.3%) and those who had sexual contact with only females or with both sexes (20.3%) than those who had no sexual contact (1.1%) and higher among those who had sexual contact with only females or with both sexes (20.3%) than those who had sexual contact with only males (7.3%). Among male students, the prevalence was higher among those who had sexual contact with only females (22.3%) and those who had sexual contact with only males or with both sexes (12.1%) than those who had no sexual contact (1.5%) and higher among those who had sexual contact with only males or with both sexes (22.3%) than those who had sexual contact with only females (12.1%). The prevalence also was higher among male students who had sexual contact with only females (12.1%) than female students who had sexual contact with only males (7.3%).

Trend analyses indicated that during 2001–2017, a significant linear decrease (13.3%–6.6%) occurred in the overall prevalence of having ever used hallucinogenic drugs. A significant quadratic trend also was identified. The prevalence of having ever used hallucinogenic drugs decreased during 2001–2005 (13.3%–8.5%) and then decreased more slowly during 2005–2017 (8.5%–6.6%). The prevalence of having ever used hallucinogenic drugs did not change significantly from 2015 (6.4%) to 2017 (6.6%).

The question measuring the prevalence of having ever used hallucinogenic drugs was not included in the standard questionnaire used in the state and large urban school district surveys in 2017. As a result, the range and median prevalence estimates across states and large urban school districts for the prevalence of having ever used hallucinogenic drugs are not available.

#### Ever Took Steroids Without a Doctor’s Prescription

Nationwide, 2.9% of students had taken steroid pills or shots without a doctor’s prescription one or more times during their life (Supplementary Table 125). The prevalence of having ever taken steroids without a doctor’s prescription was higher among male (3.3%) than female (2.4%) students. The prevalence of having ever taken steroids without a doctor’s prescription was higher among Hispanic (3.5%) than white (2.2%) students. The prevalence of having ever taken steroids without a doctor’s prescription was higher among 12th-grade male (3.8%) than 9th-grade male (2.4%) students.

Analyses based on the question ascertaining sexual identity indicated that nationwide, 2.3% of heterosexual students; 6.1% of gay, lesbian, and bisexual students; and 6.5% of not sure students had ever taken steroids without a doctor’s prescription (Supplementary Table 125). The prevalence of having ever taken steroids without a doctor’s prescription was higher among gay, lesbian, and bisexual (6.1%) and not sure (6.5%) than heterosexual (2.3%) students. Among female students, the prevalence was higher among lesbian and bisexual (4.8%) than heterosexual (1.8%) students. Among male students, the prevalence was higher among gay and bisexual (9.8%) and not sure (7.7%) than heterosexual (2.8%) students. The prevalence also was higher among heterosexual male (2.8%) than heterosexual female (1.8%) students.

Analyses based on the question ascertaining the sex of sexual contacts indicated that nationwide, 3.9% of students who had sexual contact with only the opposite sex, 8.0% of students who had sexual contact with only the same sex or with both sexes, and 0.7% of students who had no sexual contact had ever taken steroids without a doctor’s prescription (Supplementary Table 125). The prevalence of having ever taken steroids without a doctor’s prescription was higher among students who had sexual contact with only the opposite sex (3.9%) and students who had sexual contact with only the same sex or with both sexes (8.0%) than students who had no sexual contact (0.7%) and higher among students who had sexual contact with only the same sex or with both sexes (8.0%) than students who had sexual contact with only the opposite sex (3.9%). Among female students, the prevalence was higher among those who had sexual contact with only males (2.6%) and those who had sexual contact with only females or with both sexes (7.2%) than those who had no sexual contact (1.0%) and higher among those who had sexual contact with only females or with both sexes (7.2%) than those who had sexual contact with only males (2.6%). Among male students, the prevalence was higher among those who had sexual contact with only females (4.9%) and those who had sexual contact with only males or with both sexes (10.1%) than those who had no sexual contact (0.5%). The prevalence also was higher among male students who had sexual contact with only females (4.9%) than female students who had sexual contact with only males (2.6%).

Trend analyses did not identify a significant linear trend in the overall prevalence of having ever taken steroids without a doctor’s prescription during 1991–2017 (2.7%–2.9%). A significant quadratic trend was identified. The prevalence of having ever taken steroids without a doctor’s prescription increased during 1991–2001 (2.7%–5.0%) and then decreased during 2001–2017 (5.0%–2.9%). The prevalence of having ever taken steroids without a doctor’s prescription did not change significantly from 2015 (3.5%) to 2017 (2.9%).

Analyses of state and large urban school district data indicated that across 22 states, the overall prevalence of having ever taken steroids without a doctor’s prescription ranged from 2.1% to 9.2% across state surveys (median: 3.5%) (Supplementary Table 126). Across 14 large urban school districts, the prevalence ranged from 2.6% to 7.5% (median: 4.5%).

#### Ever Took Prescription Pain Medicine Without a Doctor’s Prescription or Differently than How a Doctor Told Them to Use It

Nationwide, 14.0% of students had taken prescription pain medicine (counting drugs such as codeine, Vicodin, OxyContin, Hydrocodone, and Percocet) without a do­­ctor’s prescription or differently than how a doctor told them to use it one or more times during their life (Supplementary Table 127)). The prevalence of having ever taken prescription pain medicine without a doctor’s prescription or differently than how a doctor told them to use it was higher among Hispanic (15.1%) than black (12.3%) students and higher among Hispanic female (16.1%) than black female (12.5%) students. The prevalence of having ever taken prescription pain medicine without a doctor’s prescription or differently than how a doctor told them to use it was higher among 11th-grade (15.4%) and 12th-grade (17.0%) than 9th-grade (10.9%) students, higher among 12th-grade (17.0%) than 10th-grade (12.8%) students, higher among 11th-grade female (16.4%) and 12th-grade female (16.2%) than 9th-grade female (12.1%) students, higher among 11th-grade male (14.3%) and 12th-grade male (17.7%) than 9th-grade male (9.7%) students, and higher among 12th-grade male (17.7%) than 10th-grade male (12.2%) students.

Analyses based on the question ascertaining sexual identity indicated that nationwide, 12.9% of heterosexual students; 24.3% of gay, lesbian, and bisexual students; and 17.7% of not sure students had ever taken prescription pain medicine without a doctor’s prescription or differently than how a doctor told them to use it (Supplementary Table 127). The prevalence of having ever taken prescription pain medicine without a doctor’s prescription or differently than how a doctor told them to use it was higher among gay, lesbian, and bisexual (24.3%) and not sure (17.7%) than heterosexual (12.9%) students and higher among gay, lesbian, and bisexual (24.3%) than not sure (17.7%) students. Among female students, the prevalence was higher among lesbian and bisexual (23.8%) than heterosexual (12.9%) students. Among male students, the prevalence was higher among gay and bisexual (25.4%) than heterosexual (12.8%) and not sure (13.7%) students.

Analyses based on the question ascertaining the sex of sexual contacts indicated that nationwide, 19.9% of students who had sexual contact with only the opposite sex, 35.3% of students who had sexual contact with only the same sex or with both sexes, and 5.7% of students who had no sexual contact had ever taken prescription pain medicine without a doctor’s prescription or differently than how a doctor told them to use it (Supplementary Table 127). The prevalence of having ever taken prescription pain medicine without a doctor’s prescription or differently than how a doctor told them to use it was higher among students who had sexual contact with only the opposite sex (19.9%) and students who had sexual contact with only the same sex or with both sexes (35.3%) than students who had no sexual contact (5.7%) and higher among students who had sexual contact with only the same sex or with both sexes (35.3%) than students who had sexual contact with only the opposite sex (19.9%). Among female students, the prevalence was higher among those who had sexual contact with only males (18.9%) and those who had sexual contact with only females or with both sexes (37.2%) than those who had no sexual contact (6.9%) and higher among those who had sexual contact with only females or with both sexes (37.2%) than those who had sexual contact with only males (18.9%). Among male students, the prevalence was higher among those who had sexual contact with only females (20.8%) and those who had sexual contact with only males or with both sexes (29.8%) than those who had no sexual contact (4.4%) and higher among those who had sexual contact with only males or with both sexes (29.8%) than those who had sexual contact with only females (20.8%). The prevalence also was higher among female students who had no sexual contact (6.9%) than male students who had no sexual contact (4.4%).

The question measuring the prevalence of having ever taken prescription pain medicine without a doctor’s prescription or differently than how a doctor told them to use it was used for the first time in the 2017 national YRBS. As a result, long-term temporal trends and 2-year temporal changes are not available for this variable.

Analyses of state and large urban school district data indicated that across 36 states, the overall prevalence of having ever taken prescription pain medicine without a doctor’s prescription or differently than how a doctor told them to use it ranged from 7.8% to 19.3% across state surveys (median: 13.7%) (Supplementary Table 128). Across 20 large urban school districts, the prevalence ranged from 8.9% to 18.1% (median: 12.8%).

#### Ever Injected Any Illegal Drug

Nationwide, 1.5% of students had used a needle to inject any illegal drug into their body one or more times during their life (Supplementary Table 129). The prevalence of having ever injected any illegal drug was higher among male (2.0%) than female (0.8%) students; higher among white male (1.4%), black male (2.6%), and Hispanic male (2.1%) than white female (0.5%), black female (1.1%), and Hispanic female (0.9%) students, respectively; and higher among 9th-grade male (2.1%) and 10th-grade male (1.9%) than 9th-grade female (0.6%) and 10th-grade female (0.6%) students, respectively. The prevalence of having ever injected any illegal drug was higher among 12th-grade (1.9%) than 11th-grade (1.1%) students and higher among 12th-grade female (1.3%) than 11th-grade female (0.7%) students.

Analyses based on the question ascertaining sexual identity indicated that nationwide, 1.0% of heterosexual students; 3.4% of gay, lesbian, and bisexual students; and 6.1% of not sure students had ever injected any illegal drug (Supplementary Table 129). The prevalence of having ever injected any illegal drug was higher among gay, lesbian, and bisexual (3.4%) and not sure (6.1%) than heterosexual (1.0%) students. Among female students, the prevalence was higher among lesbian and bisexual (2.3%) than heterosexual (0.4%) students. Among male students, the prevalence was higher among gay and bisexual (5.7%) and not sure (8.0%) than heterosexual (1.5%) students. The prevalence also was higher among heterosexual male (1.5%) than heterosexual female (0.4%) students and higher among gay and bisexual male (5.7%) than lesbian and bisexual female (2.3%) students.

Analyses based on the question ascertaining the sex of sexual contacts indicated that nationwide, 1.4% of students who had sexual contact with only the opposite sex, 6.0% of students who had sexual contact with only the same sex or with both sexes, and 0.2% of students who had no sexual contact had ever injected any illegal drug (Supplementary Table 129). The prevalence of having ever injected any illegal drug was higher among students who had sexual contact with only the opposite sex (1.4%) and students who had sexual contact with only the same sex or with both sexes (6.0%) than students who had no sexual contact (0.2%) and higher among students who had sexual contact with only the same sex or with both sexes (6.0%) than students who had sexual contact with only the opposite sex (1.4%). Among female students, the prevalence was higher among those who had sexual contact with only females or with both sexes (4.3%) than those who had sexual contact with only males (0.3%) and those who had no sexual contact (0.3%). Among male students, the prevalence was higher among those who had sexual contact with only females (2.4%) and those who had sexual contact with only males or with both sexes (11.2%) than those who had no sexual contact (0.1%) and higher among those who had sexual contact with only males or with both sexes (11.2%) than those who had sexual contact with only females (2.4%). The prevalence also was higher among male students who had sexual contact with only females (2.4%) than female students who had sexual contact with only males (0.3%).

Trend analyses indicated that during 1995–2017, a significant linear decrease (2.1%–1.5%) occurred in the overall prevalence of having ever injected any illegal drug. A significant quadratic trend also was identified. The prevalence of having ever injected any illegal drug did not change significantly during 1995–2011 (2.1%–2.3%) and then decreased during 2011–2017 (2.3%–1.5%). The prevalence of having ever injected any illegal drug did not change significantly from 2015 (1.8%) to 2017 (1.5%).

Analyses of state and large urban school district data indicated that across 24 states, the overall prevalence of having ever injected any illegal drug ranged from 1.4% to 8.0% across state surveys (median: 2.4%) (Supplementary Table 130). Across 16 large urban school districts, the prevalence ranged from 1.4% to 6.1% (median: 3.3%).

#### Were Offered, Sold, or Given an Illegal Drug on School Property

Nationwide, 19.8% of students had been offered, sold, or given an illegal drug on school property during the 12 months before the survey (Supplementary Table 131). The prevalence of having been offered, sold, or given an illegal drug on school property was higher among male (20.9%) than female (18.7%) students; higher among white male (19.6%) than white female (15.9%) students; and higher among 10th-grade male (22.1%) and 12th-grade male (21.5%) than 10th-grade female (18.5%) and 12th-grade female (17.8%) students, respectively. The prevalence of having been offered, sold, or given an illegal drug on school property was higher among Hispanic (25.4%) than white (17.7%) and black (18.9%) students, higher among Hispanic female (25.0%) than white female (15.9%) and black female (18.2%) students, and higher among Hispanic male (25.8%) than white male (19.6%) and black male (19.6%) students.

Analyses based on the question ascertaining sexual identity indicated that nationwide, 18.9% of heterosexual students; 28.2% of gay, lesbian, and bisexual students; and 19.6% of not sure students had been offered, sold, or given an illegal drug on school property (Supplementary Table 131). The prevalence of having been offered, sold, or given an illegal drug on school property was higher among gay, lesbian, and bisexual (28.2%) than heterosexual (18.9%) and not sure (19.6%) students. Among female students, the prevalence was higher among lesbian and bisexual (28.1%) than heterosexual (17.2%) and not sure (18.7%) students. Among male students, the prevalence was higher among gay and bisexual (28.8%) than heterosexual (20.4%) students. The prevalence also was higher among heterosexual male (20.4%) than heterosexual female (17.2%) students.

Analyses based on the question ascertaining the sex of sexual contacts indicated that nationwide, 24.3% of students who had sexual contact with only the opposite sex, 31.9% of students who had sexual contact with only the same sex or with both sexes, and 13.3% of students who had no sexual contact had been offered, sold, or given an illegal drug on school property (Supplementary Table 131). The prevalence of having been offered, sold, or given an illegal drug on school property was higher among students who had sexual contact with only the opposite sex (24.3%) and students who had sexual contact with only the same sex or with both sexes (31.9%) than students who had no sexual contact (13.3%) and higher among students who had sexual contact with only the same sex or with both sexes (31.9%) than students who had sexual contact with only the opposite sex (24.3%). Among female students, the prevalence was higher among those who had sexual contact with only males (22.2%) and those who had sexual contact with only females or with both sexes (33.1%) than those who had no sexual contact (13.0%) and higher among those who had sexual contact with only females or with both sexes (33.1%) than those who had sexual contact with only males (22.2%). Among male students, the prevalence was higher among those who had sexual contact with only females (26.0%) and those who had sexual contact with only males or with both sexes (28.5%) than those who had no sexual contact (13.6%). The prevalence also was higher among male students who had sexual contact with only females (26.0%) than female students who had sexual contact with only males (22.2%).

Trend analyses indicated that during 1993–2017, a significant linear decrease (24.0%–19.8%) occurred in the overall prevalence of having been offered, sold, or given an illegal drug on school property. A significant quadratic trend also was identified. The prevalence of having been offered, sold, or given an illegal drug on school property increased during 1993–1997 (24.0%–31.7%) and then decreased during 1997–2017 (31.7%–19.8%). The prevalence of having been offered, sold, or given an illegal drug on school property did not change significantly from 2015 (21.7%) to 2017 (19.8%).

Analyses of state and large urban school district data indicated that across 34 states, the overall prevalence of having been offered, sold, or given an illegal drug on school property ranged from 12.1% to 30.7% across state surveys (median: 22.3%) (Supplementary Table 132). Across 19 large urban school districts, the prevalence ranged from 19.7% to 32.2% (median: 27.6%).

### Sexual Behaviors Related to Unintended Pregnancy and Sexually Transmitted Infections, Including HIV Infection

#### Ever Had Sexual Intercourse

Nationwide, 39.5% of students had ever had sexual intercourse (Supplementary Table 133). The prevalence of having ever had sexual intercourse was higher among male (41.4%) than female (37.7%) students; higher among black male (52.7%) and Hispanic male (44.1%) than black female (39.4%) and Hispanic female (37.9%) students, respectively; and higher among 9th-grade male (23.3%) than 9th-grade female (17.2%) students. The prevalence of having ever had sexual intercourse was higher among black (45.8%) than white (38.6%) students, higher among black male (52.7%) and Hispanic male (44.1%) than white male (38.5%) students, and higher among black male (52.7%) than Hispanic male (44.1%) students. The prevalence of having ever had sexual intercourse was higher among 10th-grade (36.2%), 11th-grade (47.3%), and 12th-grade (57.3%) than 9th-grade (20.4%) students; higher among 11th-grade (47.3%) and 12th-grade (57.3%) than 10th-grade (36.2%) students; higher among 12th-grade (57.3%) than 11th-grade (47.3%) students; higher among 10th-grade female (34.4%), 11th-grade female (45.8%), and 12th-grade female (55.8%) than 9th-grade female (17.2%) students; higher among 11th-grade female (45.8%) and 12th-grade female (55.8%) than 10th-grade female (34.4%) students; higher among 12th-grade female (55.8%) than 11th-grade female (45.8%) students; higher among 10th-grade male (38.0%), 11th-grade male (48.8%), and 12th-grade male (58.9%) than 9th-grade male (23.3%) students; higher among 11th-grade male (48.8%) and 12th-grade male (58.9%) than 10th-grade male (38.0%) students; and higher among 12th-grade male (58.9%) than 11th-grade male (48.8%) students.

Analyses based on the question ascertaining sexual identity indicated that nationwide, 39.1% of heterosexual students; 48.4% of gay, lesbian, and bisexual students; and 28.4% of not sure students had ever had sexual intercourse (Supplementary Table 133). The prevalence of having ever had sexual intercourse was higher among heterosexual (39.1%) and gay, lesbian, and bisexual (48.4%) than not sure (28.4%) students and higher among gay, lesbian, and bisexual (48.4%) than heterosexual (39.1%) students. Among female students, the prevalence was higher among heterosexual (36.3%) and lesbian and bisexual (50.1%) than not sure (25.7%) students and higher among lesbian and bisexual (50.1%) than heterosexual (36.3%) students. Among male students, the prevalence was higher among heterosexual (41.6%) than not sure (30.8%) students. The prevalence also was higher among heterosexual male (41.6%) than heterosexual female (36.3%) students.

Analyses based on the question ascertaining the sex of sexual contacts indicated that nationwide, 78.2% of students who had sexual contact with only the opposite sex and 74.5% of students who had sexual contact with only the same sex or with both sexes had ever had sexual intercourse (students who had no sexual contact are excluded from these analyses) (Supplementary Table 133).

Trend analyses indicated that during 1991–2017, a significant linear decrease (54.1%–39.5%) occurred in the overall prevalence of having ever had sexual intercourse. A significant quadratic trend was not identified. The prevalence of having ever had sexual intercourse did not change significantly from 2015 (41.2%) to 2017 (39.5%).

Analyses of state and large urban school district data indicated that across 33 states, the overall prevalence of having ever had sexual intercourse ranged from 29.1% to 45.9% across state surveys (median: 37.7%) (Supplementary Table 134). Across 20 large urban school districts, the prevalence ranged from 21.7% to 49.2% (median: 37.2%).

#### Had Sexual Intercourse Before Age 13 Years

Nationwide, 3.4% of students had had sexual intercourse for the first time before age 13 years (Supplementary Table 135). The prevalence of having had sexual intercourse before age 13 years was higher among male (4.8%) than female (2.0%) students; higher among black male (12.8%) and Hispanic male (6.0%) than black female (2.5%) and Hispanic female (1.9%) students, respectively; and higher among 9th-grade male (5.7%), 10th-grade male (4.6%), 11th-grade male (3.5%), and 12th-grade male (5.1%) than 9th-grade female (2.2%), 10th-grade female (2.2%), 11th-grade female (1.2%), and 12th-grade female (1.9%) students, respectively. The prevalence of having had sexual intercourse before age 13 years was higher among black (7.5%) and Hispanic (4.0%) than white (2.1%) students, higher among black (7.5%) than Hispanic (4.0%) students, higher among black male (12.8%) and Hispanic male (6.0%) than white male (2.3%) students, and higher among black male (12.8%) than Hispanic male (6.0%) students. The prevalence of having had sexual intercourse before age 13 years was higher among 9th-grade (4.1%), 10th-grade (3.4%), and 12th-grade (3.5%) than 11th-grade (2.3%) students and higher among 9th-grade male (5.7%) and 12th-grade male (5.1%) than 11th-grade male (3.5%) students.

Analyses based on the question ascertaining sexual identity indicated that nationwide, 3.0% of heterosexual students; 6.1% of gay, lesbian, and bisexual students; and 4.1% of not sure students had had sexual intercourse before age 13 years (Supplementary Table 135). The prevalence of having had sexual intercourse before age 13 years was higher among gay, lesbian, and bisexual (6.1%) than heterosexual (3.0%) students. Among female students, the prevalence was higher among lesbian and bisexual (5.2%) than heterosexual (1.3%) students. The prevalence also was higher among heterosexual male (4.6%) than heterosexual female (1.3%) students.

Analyses based on the question ascertaining the sex of sexual contacts indicated that nationwide, 5.8% of students who had sexual contact with only the opposite sex and 10.5% of students who had sexual contact with only the same sex or with both sexes had had sexual intercourse before age 13 years (students who had no sexual contact are excluded from these analyses) (Supplementary Table 135). The prevalence of having had sexual intercourse before age 13 years was higher among students who had sexual contact with only the same sex or with both sexes (10.5%) than students who had sexual contact with only the opposite sex (5.8%). Among female students, the prevalence was higher among those who had sexual contact with only females or with both sexes (8.2%) than those who had sexual contact with only males (2.8%). Among male students, the prevalence was higher among those who had sexual contact with only males or with both sexes (17.5%) than those who had sexual contact with only females (8.4%). The prevalence also was higher among male students who had sexual contact with only females (8.4%) than female students who had sexual contact with only males (2.8%) and higher among male students who had sexual contact with only males or with both sexes (17.5%) than female students who had sexual contact with only females or with both sexes (8.2%).

Trend analyses indicated that during 1991–2017, a significant linear decrease (10.2%–3.4%) occurred in the overall prevalence of having had sexual intercourse before age 13 years. A significant quadratic trend was not identified. The prevalence of having had sexual intercourse before age 13 years did not change significantly from 2015 (3.9%) to 2017 (3.4%).

Analyses of state and large urban school district data indicated that across 35 states, the overall prevalence of having had sexual intercourse before age 13 years ranged from 2.1% to 6.0% across state surveys (median: 3.3%) (Supplementary Table 136). Across 20 large urban school districts, the prevalence ranged from 2.7% to 9.0% (median: 4.7%).

#### Had Sexual Intercourse with Four or More Persons

Nationwide, 9.7% of students had had sexual intercourse with four or more persons during their life (Supplementary Table 137). The prevalence of having had sexual intercourse with four or more persons was higher among male (11.6%) than female (7.9%) students; higher among black male (23.2%) and Hispanic male (12.0%) than black female (7.0%) and Hispanic female (6.8%) students, respectively; and higher among 9th-grade male (6.0%), 10th-grade male (9.7%), and 11th-grade male (12.2%) than 9th-grade female (1.8%), 10th-grade female (5.1%), and 11th-grade female (9.1%) students, respectively. The prevalence of having had sexual intercourse with four or more persons was higher among black (14.8%) than white (8.6%) and Hispanic (9.4%) students, higher among black male (23.2%) and Hispanic male (12.0%) than white male (8.6%) students, and higher among black male (23.2%) than Hispanic male (12.0%) students. The prevalence of having had sexual intercourse with four or more persons was higher among 10th-grade (7.3%), 11th-grade (10.6%), and 12th-grade (18.0%) than 9th-grade (4.0%) students; higher among 11th-grade (10.6%) and 12th-grade (18.0%) than 10th-grade (7.3%) students; higher among 12th-grade (18.0%) than 11th-grade (10.6%) students; higher among 10th-grade female (5.1%), 11th-grade female (9.1%), and 12th-grade female (16.5%) than 9th-grade female (1.8%) students; higher among 11th-grade female (9.1%) and 12th-grade female (16.5%) than 10th-grade female (5.1%) students; higher among 12th-grade female (16.5%) than 11th-grade female (9.1%) students; higher among 10th-grade male (9.7%), 11th-grade male (12.2%), and 12th-grade male (19.5%) than 9th-grade male (6.0%) students; higher among 11th-grade male (12.2%) and 12th-grade male (19.5%) than 10th-grade male (9.7%) students; and higher among 12th-grade male (19.5%) than 11th-grade male (12.2%) students.

Analyses based on the question ascertaining sexual identity indicated that nationwide, 9.1% of heterosexual students; 14.7% of gay, lesbian, and bisexual students; and 9.9% of not sure students had had sexual intercourse with four or more persons (Supplementary Table 137). The prevalence of having had sexual intercourse with four or more persons was higher among gay, lesbian, and bisexual (14.7%) than heterosexual (9.1%) and not sure (9.9%) students. Among female students, the prevalence was higher among lesbian and bisexual (15.0%) than heterosexual (6.5%) and not sure (8.0%) students. The prevalence also was higher among heterosexual male (11.5%) than heterosexual female (6.5%) students.

Analyses based on the question ascertaining the sex of sexual contacts indicated that nationwide, 17.7% of students who had sexual contact with only the opposite sex and 28.6% of students who had sexual contact with only the same sex or with both sexes (students who had no sexual contact are excluded from these analyses) had had sexual intercourse with four or more persons (Supplementary Table 137). The prevalence of having had sexual intercourse with four or more persons was higher among students who had sexual contact with only the same sex or with both sexes (28.6%) than students who had sexual contact with only the opposite sex (17.7%). Among female students, the prevalence was higher among those who had sexual contact with only females or with both sexes (30.1%) than those who had sexual contact with only males (12.5%). The prevalence also was higher among male students who had sexual contact with only females (22.1%) than female students who had sexual contact with only males (12.5%).

Trend analyses indicated that during 1991–2017, a significant linear decrease (18.7%–9.7%) occurred in the overall prevalence of having had sexual intercourse with four or more persons. A significant quadratic trend was not identified. The prevalence of having had sexual intercourse with four or more persons did not change significantly from 2015 (11.5%) to 2017 (9.7%).

Analyses of state and large urban school district data indicated that across 32 states, the overall prevalence of having had sexual intercourse with four or more persons ranged from 5.4% to 12.7% across state surveys (median: 8.8%) (Supplementary Table 138). Across 19 large urban school districts, the prevalence ranged from 5.9% to 14.8% (median: 9.5%).

#### Currently Sexually Active

Nationwide, 28.7% of students had had sexual intercourse with at least one person during the 3 months before the survey (i.e., currently sexually active) (Supplementary Table 139). The prevalence of being currently sexually active was higher among black male (34.6%) than black female (28.4%) students. The prevalence of being currently sexually active was higher among black male (34.6%) than white male (27.6%) students. The prevalence of being currently sexually active was higher among 10th-grade (24.9%), 11th-grade (35.3%), and 12th-grade (44.3%) than 9th-grade (12.9%) students; higher among 11th-grade (35.3%) and 12th-grade (44.3%) than 10th-grade (24.9%) students; higher among 12th-grade (44.3%) than 11th-grade (35.3%) students; higher among 10th-grade female (24.6%), 11th-grade female (35.8%), and 12th-grade female (45.1%) than 9th-grade female (11.7%) students; higher among 11th-grade female (35.8%) and 12th-grade female (45.1%) than 10th-grade female (24.6%) students; higher among 12th-grade female (45.1%) than 11th-grade female (35.8%) students; higher among 10th-grade male (25.3%), 11th-grade male (34.7%), and 12th-grade male (43.5%) than 9th-grade male (14.1%) students; higher among 11th-grade male (34.7%) and 12th-grade male (43.5%) than 10th-grade male (25.3%) students; and higher among 12th-grade male (43.5%) than 11th-grade male (34.7%) students.

Analyses based on the question ascertaining sexual identity indicated that nationwide, 28.5% of heterosexual students; 33.7% of gay, lesbian, and bisexual students; and 19.8% of not sure students were currently sexually active (Supplementary Table 139). The prevalence of being currently sexually active was higher among heterosexual (28.5%) and gay, lesbian, and bisexual (33.7%) than not sure (19.8%) students and higher among gay, lesbian, and bisexual (33.7%) than heterosexual (28.5%) students. Among female students, the prevalence was higher among heterosexual (28.0%) and lesbian and bisexual (36.5%) than not sure (18.6%) students and higher among lesbian and bisexual (36.5%) than heterosexual (28.0%) students. The prevalence also was higher among lesbian and bisexual female (36.5%) than gay and bisexual male (26.0%) students.

Analyses based on the question ascertaining the sex of sexual contacts indicated that nationwide, 56.7% of students who had sexual contact with only the opposite sex and 55.6% of students who had sexual contact with only the same sex or with both sexes were currently sexually active (students who had no sexual contact are excluded from these analyses) (Supplementary Table 139). The prevalence of being currently sexually active was higher among female students who had sexual contact with only females or with both sexes (58.0%) than male students who had sexual contact with only males or with both sexes (48.0%).

Trend analyses indicated that during 1991–2017, a significant linear decrease (37.5%–28.7%) occurred in the overall prevalence of being currently sexually active. A significant quadratic trend also was identified. The prevalence of being currently sexually active decreased during 1991–2013 (37.5%–34.0%) and then decreased more rapidly during 2013–2017 (34.0%–28.7%). The prevalence of being currently sexually active did not change significantly from 2015 (30.1%) to 2017 (28.7%).

Analyses of state and large urban school district data indicated that across 35 states, the overall prevalence of being currently sexually active ranged from 19.2% to 33.5% across state surveys (median: 26.3%) (Supplementary Table 140). Across 19 large urban school districts, the prevalence ranged from 15.4% to 35.6% (median: 25.0%).

#### Used a Condom During Last Sexual Intercourse

Among the 28.7% of currently sexually active students nationwide, 53.8% reported that either they or their partner had used a condom during last sexual intercourse (Supplementary Table 141). The prevalence of having used a condom during last sexual intercourse was higher among male (61.3%) than female (46.9%) students; higher among white male (61.9%), black male (57.9%), and Hispanic male (62.4%) than white female (47.0%), black female (45.8%), and Hispanic female (47.1%) students, respectively; and higher among 9th-grade male (61.1%), 10th-grade male (63.2%), 11th-grade male (63.1%), and 12th-grade male (59.1%) than 9th-grade female (46.8%), 10th-grade female (52.4%), 11th-grade female (50.0%), and 12th-grade female (41.3%) students, respectively. The prevalence of having used a condom during last sexual intercourse was higher among 10th-grade (57.8%) and 11th-grade (56.3%) than 12th-grade (49.9%) students and higher among 10th-grade female (52.4%) and 11th-grade female (50.0%) than 12th-grade female (41.3%) students.

Analyses based on the question ascertaining sexual identity indicated that nationwide, among currently sexually active students, 56.1% of heterosexual students; 39.9% of gay, lesbian, and bisexual students; and 44.1% of not sure students had used a condom during last sexual intercourse (Supplementary Table 141). The prevalence of having used a condom during last sexual intercourse was higher among heterosexual (56.1%) than gay, lesbian, and bisexual (39.9%) students. Among female students, the prevalence was higher among heterosexual (49.6%) than lesbian and bisexual (37.3%) students. The prevalence also was higher among heterosexual male (61.8%) than heterosexual female (49.6%) students.

Analyses based on the question ascertaining the sex of sexual contacts indicated that nationwide, among currently sexually active students, 56.3% of students who had sexual contact with only the opposite sex and 39.7% of students who had sexual contact with only the same sex or with both sexes had used a condom during last sexual intercourse (male and female students who had no sexual contact and female students who had sexual contact with only females are excluded from these analyses) (Supplementary Table 141). The prevalence of having used a condom during last sexual intercourse was higher among students who had sexual contact with only the opposite sex (56.3%) than students who had sexual contact with only the same sex or with both sexes (39.7%). Among female students, the prevalence was higher among those who had sexual contact with only males (50.3%) than those who had sexual contact with both sexes (36.1%). The prevalence also was higher among male students who had sexual contact with only females (61.6%) than female students who had sexual contact with only males (50.3%).

Trend analyses indicated that during 1991–2017, a significant linear increase (46.2%–53.8%) occurred in the overall prevalence of having used a condom during last sexual intercourse, among currently sexually active students. A significant quadratic trend also was identified. The prevalence of having used a condom during last sexual intercourse increased during 1991–2005 (46.2%–62.8%) and then decreased during 2005–2017 (62.8%–53.8%). The prevalence of having used a condom during last sexual intercourse did not change significantly from 2015 (56.9%) to 2017 (53.8%).

Analyses of state and large urban school district data indicated that across 35 states, the overall prevalence of having used a condom during last sexual intercourse, among currently sexually active students, ranged from 42.7% to 65.6% across state surveys (median: 54.4%) (Supplementary Table 142). Across 19 large urban school districts, the prevalence ranged from 45.0% to 64.5% (median: 56.3%).

#### Used Birth Control Pills Before Last Sexual Intercourse

Among the 28.7% of currently sexually active students nationwide, 20.7% reported that either they or their partner had used birth control pills to prevent pregnancy before last sexual intercourse (Supplementary Table 143). The prevalence of having used birth control pills before last sexual intercourse was higher among 12th-grade female (31.4%) than 12th-grade male (22.8%) students. The prevalence of having used birth control pills before last sexual intercourse was higher among white (27.1%) than black (13.2%) and Hispanic (12.1%) students, higher among white female (29.6%) than black female (11.2%) and Hispanic female (12.0%) students, and higher among white male (24.5%) than black male (15.1%) and Hispanic male (12.1%) students. The prevalence of having used birth control pills before last sexual intercourse was higher among 10th-grade (17.0%), 11th-grade (20.6%), and 12th-grade (27.2%) than 9th-grade (8.6%) students; higher among 12th-grade (27.2%) than 10th-grade (17.0%) and 11th-grade (20.6%) students; higher among 10th-grade female (17.4%), 11th-grade female (19.9%), and 12th-grade female (31.4%) than 9th-grade female (10.0%) students; higher among 12th-grade female (31.4%) than 10th-grade female (17.4%) and 11th-grade female (19.9%) students; and higher among 10th-grade male (16.7%), 11th-grade male (21.5%), and 12th-grade male (22.8%) than 9th-grade male (7.2%) students.

Analyses based on the question ascertaining sexual identity indicated that nationwide, among currently sexually active students, 21.7% of heterosexual students; 15.4% of gay, lesbian, and bisexual students; and 10.2% of not sure students had used birth control pills before last sexual intercourse (Supplementary Table 143). The prevalence of having used birth control pills before last sexual intercourse was higher among heterosexual (21.7%) than gay, lesbian, and bisexual (15.4%) and not sure (10.2%) students. Among female students, the prevalence was higher among heterosexual (24.2%) than lesbian and bisexual (16.2%) students. Among male students, the prevalence was higher among heterosexual (19.5%) than gay and bisexual (10.5%) students. The prevalence also was higher among heterosexual female (24.2%) than heterosexual male (19.5%) students.

Analyses based on the question ascertaining the sex of sexual contacts indicated that nationwide, among currently sexually active students, 21.8% of students who had sexual contact with only the opposite sex and 17.0% of students who had sexual contact with both sexes had used birth control pills before last sexual intercourse (students who had no sexual contact and students who had sexual contact with only the same sex are excluded from these analyses) (Supplementary Table 143).

Trend analyses indicated that during 1991–2017, a significant linear increase (20.8%–20.7%) occurred in the overall prevalence of having used birth control pills before last sexual intercourse, among currently sexually active students.** A significant quadratic trend also was identified. The prevalence of having used birth control pills before last sexual intercourse decreased during 1991–1995 (20.8%–17.4%) and then increased during 1995–2017 (17.4%–20.7%). The prevalence of having used birth control pills before last sexual intercourse did not change significantly from 2015 (18.2%) to 2017 (20.7%).

Analyses of state and large urban school district data indicated that across 33 states, the overall prevalence of having used birth control pills before last sexual intercourse, among currently sexually active students, ranged from 14.1% to 34.8% across state surveys (median: 21.2%) (Supplementary Table 144). Across 18 large urban school districts, the prevalence ranged from 8.6% to 23.1% (median: 13.5%).

#### Used an IUD or Implant Before Last Sexual Intercourse

Among the 28.7% of currently sexually active students nationwide, 4.1% reported that either they or their partner had used an intrauterine device (IUD) (e.g., Mirena or ParaGard) or implant (e.g., Implanon or Nexplanon) to prevent pregnancy before last sexual intercourse (Supplementary Table 145). The prevalence of having used an IUD or implant before last sexual intercourse was higher among female (5.3%) than male (2.7%) students; higher among white female (6.2%) and Hispanic female (4.4%) than white male (3.4%) and Hispanic male (0.1%) students, respectively; and higher among 12th-grade female (6.0%) than 12th-grade male (3.2%) students. The prevalence of having used an IUD or implant before last sexual intercourse was higher among white (4.9%) than Hispanic (2.2%) students and higher among white male (3.4%) than Hispanic male (0.1%) students. The prevalence of having used an IUD or implant before last sexual intercourse was higher among 12th-grade male (3.2%) than 9th-grade male (1.0%) students.

Analyses based on the question ascertaining sexual identity indicated that nationwide, among currently sexually active students, 4.0% of heterosexual students; 4.1% of gay, lesbian, and bisexual students; and 6.2% of not sure students had used an IUD or implant before last sexual intercourse (Supplementary Table 145). Among male students, the prevalence of having used an IUD or implant before last sexual intercourse was higher among heterosexual (2.6%) and not sure (13.0%) than gay and bisexual (0.0%) students. The prevalence also was higher among heterosexual female (5.5%) than heterosexual male (2.6%) students and higher among lesbian and bisexual female (4.9%) than gay and bisexual male (0.0%) students.

Analyses based on the question ascertaining the sex of sexual contacts indicated that nationwide, among currently sexually active students, 4.0% of students who had sexual contact with only the opposite sex and 5.9% of students who had sexual contact with both sexes had used an IUD or implant before last sexual intercourse (students who had no sexual contact and students who had sexual contact with only the same sex are excluded from these analyses) (Supplementary Table 145). The prevalence of having used an IUD or implant before last sexual intercourse was higher among female students who had sexual contact with only males (5.4%) than male students who had sexual contact with only females (2.7%).

Trend analyses indicated that during 2013–2017, a significant linear increase (1.6%–4.1%) occurred in the overall prevalence of having used an IUD or implant before last sexual intercourse, among currently sexually active students. Not enough data points were available to identify a quadratic trend. The prevalence of having used an IUD or implant before last sexual intercourse did not change significantly from 2015 (3.3%) to 2017 (4.1%).

Analyses of state and large urban school district data indicated that across 33 states, the overall prevalence of having used an IUD or implant before last sexual intercourse, among currently sexually active students, ranged from 1.9% to 13.3% across state surveys (median: 5.0%) (Supplementary Table 146). Across 18 large urban school districts, the prevalence ranged from 0.7% to 10.4% (median: 3.4%).

#### Used a Shot, Patch, or Birth Control Ring Before Last Sexual Intercourse

Among the 28.7% of currently sexually active students nationwide, 4.7% reported that either they or their partner had used a shot (e.g., Depo-Provera), patch (e.g., OrthoEvra), or birth control ring (e.g., NuvaRing) to prevent pregnancy before last sexual intercourse (Supplementary Table 147). The prevalence of having used a shot, patch, or birth control ring before last sexual intercourse was higher among female (6.9%) than male (2.2%) students; higher among white female (8.1%), black female (8.6%), and Hispanic female (3.9%) than white male (2.4%), black male (3.4%), and Hispanic male (1.1%) students, respectively; and higher among 10th-grade female (6.0%), 11th-grade female (7.5%), and 12th-grade female (7.3%) than 10th-grade male (0.9%), 11th-grade male (3.0%), and 12th-grade male (2.6%) students, respectively. The prevalence of having used a shot, patch, or birth control ring before last sexual intercourse was higher among white (5.4%) and black (6.0%) than Hispanic (2.5%) students and higher among white female (8.1%) and black female (8.6%) than Hispanic female (3.9%) students. The prevalence of having used a shot, patch, or birth control ring before last sexual intercourse was higher among 11th-grade (5.4%) than 10th-grade (3.5%) students.

Analyses based on the question ascertaining sexual identity indicated that nationwide, among currently sexually active students, 4.7% of heterosexual students; 5.0% of gay, lesbian, and bisexual students; and 2.4% of not sure students had used a shot, patch, or birth control ring before last sexual intercourse (Supplementary Table 147). Among male students, the prevalence of having used a shot, patch, or birth control ring before last sexual intercourse was higher among heterosexual (2.3%) than gay and bisexual (0.0%) and not sure (0.0%) students. The prevalence also was higher among heterosexual female (7.3%) than heterosexual male (2.3%) students and higher among lesbian and bisexual female (6.0%) than gay and bisexual male (0.0%) students.

Analyses based on the question ascertaining the sex of sexual contacts indicated that nationwide, among currently sexually active students, 4.6% of students who had sexual contact with only the opposite sex and 5.2% of students who had sexual contact with both sexes had used a shot, patch, or birth control ring before last sexual intercourse (students who had no sexual contact and students who had sexual contact with only the same sex are excluded from these analyses) (Supplementary Table 147). The prevalence of having used a shot, patch, or birth control ring before last sexual intercourse was higher among female students who had sexual contact with only males (7.3%) than male students who had sexual contact with only females (2.2%).

Trend analyses did not identify a significant linear trend in the overall prevalence of having used a shot, patch, or birth control ring before last sexual intercourse, among currently sexually active students, during 2013–2017 (4.7%–4.7%). Not enough data points were available to identify a quadratic trend. The prevalence of having used a shot, patch, or birth control ring before last sexual intercourse did not change significantly from 2015 (5.3%) to 2017 (4.7%).

Analyses of state and large urban school district data indicated that across 33 states, the overall prevalence of having used a shot, patch, or birth control ring before last sexual intercourse, among currently sexually active students, ranged from 2.1% to 7.9% across state surveys (median: 4.7%) (Supplementary Table 148). Across 18 large urban school districts, the prevalence ranged from 0.0% to 9.3% (median: 3.3%).

#### Used Birth Control Pills; an IUD or Implant; or a Shot, Patch, or Birth Control Ring Before Last Sexual Intercourse

Among the 28.7% of currently sexually active students nationwide, 29.4% reported that either they or their partner had used birth control pills; an IUD (e.g., Mirena or ParaGard) or implant (e.g., Implanon or Nexplanon); or a shot (e.g., Depo-Provera), patch (e.g., OrthoEvra), or birth control ring (e.g., NuvaRing) to prevent pregnancy before last sexual intercourse (Supplementary Table 149). The prevalence of having used birth control pills; an IUD or implant; or a shot, patch, or birth control ring before last sexual intercourse was higher among female (34.6%) than male (23.9%) students; higher among white female (43.9%) and Hispanic female (20.4%) than white male (30.3%) and Hispanic male (13.4%) students, respectively; and higher among 9th-grade female (19.2%) and 12th-grade female (44.7%) than 9th-grade male (10.1%) and 12th-grade male (28.5%) students, respectively. The prevalence of having used birth control pills; an IUD or implant; or a shot, patch, or birth control ring before last sexual intercourse was higher among white (37.4%) and black (22.5%) than Hispanic (16.8%) students, higher among white (37.4%) than black (22.5%) students, higher among white female (43.9%) than black female (23.7%) and Hispanic female (20.4%) students, higher among white male (30.3%) and black male (21.1%) than Hispanic male (13.4%) students, and higher among white male (30.3%) than black male (21.1%) students. The prevalence of having used birth control pills; an IUD or implant; or a shot, patch, or birth control ring before last sexual intercourse was higher among 10th-grade (24.1%), 11th-grade (30.4%), and 12th-grade (36.9%) than 9th-grade (14.3%) students; higher among 11th-grade (30.4%) and 12th-grade (36.9%) than 10th-grade (24.1%) students; higher among 12th-grade (36.9%) than 11th-grade (30.4%) students; higher among 12th-grade female (44.7%) than 9th-grade female (19.2%), 10th-grade female (28.5%), and 11th-grade female (32.8%) students; higher among 11th-grade female (32.8%) than 9th-grade female (19.2%) students; higher among 10th-grade male (19.6%), 11th-grade male (27.8%), and 12th-grade male (28.5%) than 9th-grade male (10.1%) students; and higher among 11th-grade male (27.8%) and 12th-grade male (28.5%) than 10th-grade male (19.6%) students.

Analyses based on the question ascertaining sexual identity indicated that nationwide, among currently sexually active students, 30.3% of heterosexual students; 24.4% of gay, lesbian, and bisexual students; and 18.8% of not sure students had used birth control pills; an IUD or implant; or a shot, patch, or birth control ring before last sexual intercourse (Supplementary Table 149). The prevalence of having used birth control pills; an IUD or implant; or a shot, patch, or birth control ring before last sexual intercourse was higher among heterosexual (30.3%) than gay, lesbian, and bisexual (24.4%) students. Among female students, the prevalence was higher among heterosexual (37.0%) than lesbian and bisexual (27.2%) and not sure (19.6%) students. Among male students, the prevalence was higher among heterosexual (24.5%) than gay and bisexual (10.5%) students. The prevalence also was higher among heterosexual female (37.0%) than heterosexual male (24.5%) students and higher among lesbian and bisexual female (27.2%) than gay and bisexual male (10.5%) students.

Analyses based on the question ascertaining the sex of sexual contacts indicated that nationwide, among currently sexually active students, 30.4% of students who had sexual contact with only the opposite sex and 28.1% of students who had sexual contact with both sexes had used birth control pills; an IUD or implant; or a shot, patch, or birth control ring before last sexual intercourse (students who had no sexual contact and students who had sexual contact with only the same sex are excluded from these analyses) (Supplementary Table 149). The prevalence of having used birth control pills; an IUD or implant; or a shot, patch, or birth control ring before last sexual intercourse was higher among female students who had sexual contact with only males (36.8%) than male students who had sexual contact with only females (24.6%).

Trend analyses indicated that during 2013–2017, a significant linear increase (25.3%–29.4%) occurred in the overall prevalence of having used birth control pills; an IUD or implant; or a shot, patch, or birth control ring before last sexual intercourse, among currently sexually active students. Not enough data points were available to identify a quadratic trend. The prevalence of having used birth control pills; an IUD or implant; or a shot, patch, or birth control ring before last sexual intercourse did not change significantly from 2015 (26.8%) to 2017 (29.4%).

Analyses of state and large urban school district data indicated that across 33 states, the overall prevalence of having used birth control pills; an IUD or implant; or a shot, patch, or birth control ring before last sexual intercourse, among currently sexually active students, ranged from 20.9% to 50.2% across state surveys (median: 33.1%) (Supplementary Table 150). Across 18 large urban school districts, the prevalence ranged from 14.0% to 36.3% (median: 21.5%).

#### Used Both a Condom During Last Sexual Intercourse and Birth Control Pills; an IUD or Implant; or a Shot, Patch, or Birth Control Ring Before Last Sexual Intercourse

Among the 28.7% of currently sexually active students nationwide, 8.8% reported that either they or their partner had used both a condom during last sexual intercourse and birth control pills; an IUD (e.g., Mirena or ParaGard) or implant (e.g., Implanon or Nexplanon); or a shot (e.g., Depo-Provera), patch (e.g., OrthoEvra), or birth control ring (e.g., NuvaRing) before last sexual intercourse to prevent pregnancy (Supplementary Table 151). The prevalence of having used both a condom during last sexual intercourse and birth control pills; an IUD or implant; or a shot, patch, or birth control ring before last sexual intercourse was higher among white (11.6%) than black (6.4%) and Hispanic (4.2%) students, higher among white female (12.2%) than black female (6.0%) and Hispanic female (3.8%) students, and higher among white male (10.9%) than Hispanic male (4.5%) students. The prevalence of having used both a condom during last sexual intercourse and birth control pills; an IUD or implant; or a shot, patch, or birth control ring before last sexual intercourse was higher among 11th-grade male (10.2%) than 9th-grade male (4.6%) students.

Analyses based on the question ascertaining sexual identity indicated that nationwide, among currently sexually active students, 9.6% of heterosexual students; 4.4% of gay, lesbian, and bisexual students; and 3.7% of not sure students had used both a condom during last sexual intercourse and birth control pills; an IUD or implant; or a shot, patch, or birth control ring before last sexual intercourse (Supplementary Table 151). The prevalence of having used both a condom during last sexual intercourse and birth control pills; an IUD or implant; or a shot, patch, or birth control ring before last sexual intercourse was higher among heterosexual (9.6%) than gay, lesbian, and bisexual (4.4%) and not sure (3.7%) students. Among female students, the prevalence was higher among heterosexual (10.2%) than lesbian and bisexual (4.3%) and not sure (2.3%) students.

Analyses based on the question ascertaining the sex of sexual contacts indicated that nationwide, among currently sexually active students, 9.5% of students who had sexual contact with only the opposite sex and 5.1% of students who had sexual contact with both sexes had used both a condom during last sexual intercourse and birth control pills; an IUD or implant; or a shot, patch, or birth control ring before last sexual intercourse (students who had no sexual contact and students who had sexual contact with only the same sex are excluded from these analyses) (Supplementary Table 151). The prevalence of having used both a condom during last sexual intercourse and birth control pills; an IUD or implant; or a shot, patch, or birth control ring before last sexual intercourse was higher among students who had sexual contact with only the opposite sex (9.5%) than students who had sexual contact with both sexes (5.1%). Among female students, the prevalence was higher among those who had sexual contact with only males (10.1%) than those who had sexual contact with both sexes (4.6%).

Trend analyses did not identify a significant linear trend in the overall prevalence of having used both a condom during last sexual intercourse and birth control pills; an IUD or implant; or a shot, patch, or birth control ring before last sexual intercourse, among currently sexually active students, during 2013–2017 (8.8%–8.8%). Not enough data points were available to identify a quadratic trend. The prevalence of having used both a condom during last sexual intercourse and birth control pills; an IUD or implant; or a shot, patch, or birth control ring before last sexual intercourse did not change significantly from 2015 (8.8%) to 2017 (8.8%).

Analyses of state and large urban school district data indicated that across 33 states, the overall prevalence of having used both a condom during last sexual intercourse and birth control pills; an IUD or implant; or a shot, patch, or birth control ring before last sexual intercourse, among currently sexually active students, ranged from 5.5% to 18.9% across state surveys (median: 11.2%) (Supplementary Table 152). Across 18 large urban school districts, the prevalence ranged from 4.5% to 10.7% (median: 6.6%).

#### Did Not Use Any Method to Prevent Pregnancy

Among the 28.7% of currently sexually active students nationwide, 13.8% reported that neither they nor their partner had used any method to prevent pregnancy during last sexual intercourse (Supplementary Table 153). The prevalence of not having used any method to prevent pregnancy was higher among female (16.7%) than male (10.5%) students; higher among black female (25.5%) than black male (10.8%) students; and higher among 9th-grade female (27.6%) and 11th-grade female (15.4%) than 9th-grade male (13.8%) and 11th-grade male (7.0%) students, respectively. The prevalence of not having used any method to prevent pregnancy was higher among black (17.8%) and Hispanic (19.0%) than white (10.0%) students, higher among black female (25.5%) and Hispanic female (22.0%) than white female (11.8%) students, and higher among Hispanic male (16.1%) than white male (7.7%) students. The prevalence of not having used any method to prevent pregnancy was higher among 9th-grade (20.1%) than 11th-grade (11.5%) and 12th-grade (12.3%) students, higher among 9th-grade female (27.6%) than 11th-grade female (15.4%) and 12th-grade female (13.7%) students, and higher among 9th-grade male (13.8%) than 11th-grade male (7.0%) students.

Analyses based on the question ascertaining sexual identity indicated that nationwide, among currently sexually active students, 11.5% of heterosexual students; 27.4% of gay, lesbian, and bisexual students; and 25.0% of not sure students had not used any method to prevent pregnancy (Supplementary Table 153). The prevalence of not having used any method to prevent pregnancy was higher among gay, lesbian, and bisexual (27.4%) and not sure (25.0%) than heterosexual (11.5%) students. Among female students, the prevalence was higher among lesbian and bisexual (27.8%) than heterosexual (13.7%) students. Among male students, the prevalence was higher among gay and bisexual (25.9%) than heterosexual (9.5%) students. The prevalence also was higher among heterosexual female (13.7%) than heterosexual male (9.5%) students.

Analyses based on the question ascertaining the sex of sexual contacts indicated that nationwide, among currently sexually active students, 11.5% of students who had sexual contact with only the opposite sex and 20.8% of students who had sexual contact with both sexes had not used any method to prevent pregnancy (students who had no sexual contact and students who had sexual contact with only the same sex are excluded from these analyses) (Supplementary Table 153). The prevalence of not having used any method to prevent pregnancy was higher among students who had sexual contact with both sexes (20.8%) than students who had sexual contact with only the opposite sex (11.5%). Among female students, the prevalence was higher among those who had sexual contact with both sexes (22.6%) than those who had sexual contact with only males (13.8%). The prevalence also was higher among female students who had sexual contact with only males (13.8%) than male students who had sexual contact with only females (9.5%) and higher among female students who had sexual contact with both sexes (22.6%) than male students who had sexual contact with both sexes (10.2%).

Trend analyses indicated that during 1991–2017, a significant linear decrease (16.5%–13.8%) occurred in the overall prevalence of not having used any method to prevent pregnancy, among currently sexually active students. A significant quadratic trend also was identified. The prevalence of not having used any method to prevent pregnancy decreased during 1991–2007 (16.5%–12.2%) and then did not change significantly during 2007–2017 (12.2%–13.8%). The prevalence of not having used any method to prevent pregnancy did not change significantly from 2015 (13.8%) to 2017 (13.8%).

Analyses of state and large urban school district data indicated that across 33 states, the overall prevalence of not having used any method to prevent pregnancy, among currently sexually active students, ranged from 6.6% to 23.1% across state surveys (median: 13.3%) (Supplementary Table 154). Across 18 large urban school districts, the prevalence ranged from 12.6% to 27.1% (median: 18.7%).

#### Drank Alcohol or Used Drugs Before Last Sexual Intercourse

Among the 28.7% of currently sexually active students nationwide, 18.8% had drunk alcohol or used drugs before last sexual intercourse (Supplementary Table 155). The prevalence of having drunk alcohol or used drugs before last sexual intercourse was higher among male (21.6%) than female (15.9%) students; higher among Hispanic male (22.6%) than Hispanic female (12.6%) students; and higher among 10th-grade male (25.6%) and 12th-grade male (23.3%) than 10th-grade female (14.1%) and 12th-grade female (17.5%), respectively. The prevalence of having drunk alcohol or used drugs before last sexual intercourse was higher among white female (16.6%) than Hispanic female (12.6%) students. The prevalence of having drunk alcohol or used drugs before last sexual intercourse was higher among 10th-grade (19.7%) and 12th-grade (20.3%) than 11th-grade (14.2%) students; higher among 12th-grade female (17.5%) than 11th-grade female (13.8%) students; and higher among 9th-grade male (24.2%), 10th-grade male (25.6%), and 12th-grade male (23.3%) than 11th-grade male (14.8%) students.

Analyses based on the question ascertaining sexual identity indicated that nationwide, among currently sexually active students, 18.0% of heterosexual students; 20.3% of gay, lesbian, and bisexual students; and 34.6% of not sure students had drunk alcohol or used drugs before last sexual intercourse (Supplementary Table 155). The prevalence of having drunk alcohol or used drugs before last sexual intercourse was higher among not sure (34.6%) than heterosexual (18.0%) students. Among female students, the prevalence was higher among lesbian and bisexual (20.2%) and not sure (30.7%) than heterosexual (14.1%) students. The prevalence also was higher among heterosexual male (21.3%) than heterosexual female (14.1%) students.

Analyses based on the question ascertaining the sex of sexual contacts indicated that nationwide, among currently sexually active students, 17.7% of students who had sexual contact with only the opposite sex and 24.8% of students who had sexual contact with both sexes had drunk alcohol or used drugs before last sexual intercourse (students who had no sexual contact are excluded from these analyses) (Supplementary Table 155). Among female students, the prevalence of having drunk alcohol or used drugs before last sexual intercourse was higher among those who had sexual contact with only females or with both sexes (26.1%) than those who had sexual contact with only males (13.2%). The prevalence also was higher among male students who had sexual contact with only females (21.7%) than female students who had sexual contact with only males (13.2%).

Trend analyses indicated that during 1991–2017, a significant linear decrease (21.6%–18.8%) occurred in the overall prevalence of having drunk alcohol or used drugs before last sexual intercourse, among currently sexually active students. A significant quadratic trend also was identified. The prevalence of having drunk alcohol or used drugs before last sexual intercourse increased during 1991–1999 (21.6%–24.8%) and then decreased during 1999–2017 (24.8%–18.8%). The prevalence of having drunk alcohol or used drugs before last sexual intercourse did not change significantly from 2015 (20.6%) to 2017 (18.8%).

Analyses of state and large urban school district data indicated that across 35 states, the overall prevalence of having drunk alcohol or used drugs before last sexual intercourse, among currently sexually active students, ranged from 13.7% to 22.8% across state surveys (median: 18.2%) (Supplementary Table 156). Across 18 large urban school districts, the prevalence ranged from 11.6% to 24.1% (median: 19.1%).

#### Ever Been Tested for HIV

Nationwide, 9.3% of students had ever been tested for HIV, not counting tests done if they donated blood (Supplementary Table 157). The prevalence of having ever been tested for HIV was higher among female (10.5%) than male (8.1%) students, higher among Hispanic female (10.1%) than Hispanic male (7.7%) students, and higher among 12th-grade female (15.8%) than 12th-grade male (10.2%) students. The prevalence of having ever been tested for HIV was higher among black (15.2%) than white (7.9%) and Hispanic (8.9%) students, higher among black female (16.6%) than white female (8.8%) and Hispanic female (10.1%) students, and higher among black male (13.7%) than white male (6.9%) and Hispanic male (7.7%) students. The prevalence of having ever been tested for HIV was higher among 10th-grade (8.2%), 11th-grade (10.3%), and 12th-grade (13.2%) than 9th-grade (6.2%) students; higher among 11th-grade (10.3%) and 12th-grade (13.2%) than 10th-grade (8.2%) students; higher among 12th-grade (13.2%) than 11th-grade (10.3%) students; higher among 11th-grade female (11.6%) and 12th-grade female (15.8%) than 9th-grade female (6.6%) and 10th-grade female (8.5%) students; higher among 12th-grade female (15.8%) than 11th-grade female (11.6%) students; and higher among 11th-grade male (9.0%) and 12th-grade male (10.2%) than 9th-grade male (5.7%) students.

Analyses based on the question ascertaining sexual identity indicated that nationwide, 9.1% of heterosexual students; 14.0% of gay, lesbian, and bisexual students; and 7.4% of not sure students had ever been tested for HIV (Supplementary Table 157). The prevalence of having ever been tested for HIV was higher among gay, lesbian, and bisexual (14.0%) than heterosexual (9.1%) and not sure (7.4%) students. Among female students, the prevalence was higher among lesbian and bisexual (14.7%) than heterosexual (10.5%) and not sure (6.5%) students. The prevalence also was higher among heterosexual female (10.5%) than heterosexual male (7.9%) students.

Analyses based on the question ascertaining the sex of sexual contacts indicated that nationwide, 13.2% of students who had sexual contact with only the opposite sex, 20.2% of students who had sexual contact with only the same sex or with both sexes, and 3.6% of students who had no sexual contact had ever been tested for HIV (Supplementary Table 157). The prevalence of having ever been tested for HIV was higher among students who had sexual contact with only the opposite sex (13.2%) and students who had sexual contact with only the same sex or with both sexes (20.2%) than students who had no sexual contact (3.6%) and higher among students who had sexual contact with only the same sex or with both sexes (20.2%) than students who had sexual contact with only the opposite sex (13.2%). Among female students, the prevalence was higher among those who had sexual contact with only males (15.6%) and those who had sexual contact with only females or with both sexes (22.0%) than those who had no sexual contact (4.4%) and higher among those who had sexual contact with only females or with both sexes (22.0%) than those who had sexual contact with only males (15.6%). Among male students, the prevalence was higher among those who had sexual contact with only females (11.3%) and those who had sexual contact with only males or with both sexes (15.1%) than those who had no sexual contact (2.7%). The prevalence also was higher among female students who had sexual contact with only males (15.6%) than male students who had sexual contact with only females (11.3%), higher among female students who had sexual contact with only females or with both sexes (22.0%) than male students who had sexual contact with only males or with both sexes (15.1%), and higher among female students who had no sexual contact (4.4%) than male students who had no sexual contact (2.7%).

Trend analyses indicated that during 2005–2017, a significant linear decrease (11.9%–9.3%) occurred in the overall prevalence of having ever been tested for HIV. A significant quadratic trend also was identified. The prevalence of having been tested for HIV did not change significantly during 2005–2013 (11.9%–12.9%) and then decreased during 2013–2017 (12.9%–9.3%). The prevalence of having been tested for HIV did not change significantly from 2015 (10.2%) to 2017 (9.3%).

Analyses of state and large urban school district data indicated that across 29 states, the overall prevalence of having ever been tested for HIV ranged from 8.2% to 23.8% across state surveys (median: 12.0%) (Supplementary Table 158). Across 21 large urban school districts, the prevalence ranged from 10.2% to 37.2% (median: 18.0%).

### Dietary Behaviors

#### Did Not Eat Fruit or Drink 100% Fruit Juices

Nationwide, 5.6% of students had not eaten fruit or drunk 100% fruit juices (e.g., orange juice, apple juice, or grape juice, not counting punch, Kool-Aid, sports drinks, or other fruit-flavored drinks) during the 7 days before the survey (Supplementary Table 159). The prevalence of not having eaten fruit or drunk 100% fruit juices was higher among male (7.2%) than female (4.0%) students; higher among white male (7.1%), black male (9.5%), and Hispanic male (6.3%) than white female (4.1%), black female (4.4%), and Hispanic female (3.7%) students, respectively; and higher among 9th-grade male (8.5%), 10th-grade male (6.4%), 11th-grade male (6.2%), and 12th-grade male (7.2%) than 9th-grade female (3.8%), 10th-grade female (4.4%), 11th-grade female (3.7%), and 12th-grade female (3.9%) students, respectively. The prevalence of not having eaten fruit or drunk 100% fruit juices was higher among black (7.0%) than white (5.5%) and Hispanic (5.0%) students and higher among black male (9.5%) than Hispanic male (6.3%) students. The prevalence of not having eaten fruit or drunk 100% fruit juices was higher among 9th-grade (6.1%) than 11th-grade (4.9%) students and higher among 9th-grade male (8.5%) than 11th-grade male (6.2%) students.

Analyses based on the question ascertaining sexual identity indicated that nationwide, 5.6% of heterosexual students; 4.4% of gay, lesbian, and bisexual students; and 8.9% of not sure students had not eaten fruit or drunk 100% fruit juices (Supplementary Table 159). The prevalence of not having eaten fruit or drunk 100% fruit juices was higher among not sure (8.9%) than gay, lesbian, and bisexual (4.4%) students. The prevalence also was higher among heterosexual male (7.0%) than heterosexual female (4.1%) students, higher among gay and bisexual male (7.5%) than lesbian and bisexual female (3.2%) students, and higher among not sure male (12.8%) than not sure female (5.8%) students.

Analyses based on the question ascertaining the sex of sexual contacts indicated that nationwide, 4.4% of students who had sexual contact with only the opposite sex, 4.6% of students who had sexual contact with only the same sex or with both sexes, and 5.8% of students who had no sexual contact had not eaten fruit or drunk 100% fruit juices (Supplementary Table 159). Among male students, the prevalence was higher among those who had no sexual contact (7.5%) than those who had sexual contact with only females (5.5%). The prevalence also was higher among male students who had sexual contact with only females (5.5%) than female students who had sexual contact with only males (3.1%), higher among male students who had sexual contact with only males or with both sexes (8.9%) than female students who had sexual contact with only females or with both sexes (3.1%), and higher among male students who had no sexual contact (7.5%) than female students who had no sexual contact (4.2%).

Trend analyses did not identify a significant linear trend in the overall prevalence of not having eaten fruit or drunk 100% fruit juices during 1999–2017 (5.4%–5.6%). A significant quadratic trend also was not identified. The prevalence of not having eaten fruit or drunk 100% fruit juices did not change significantly from 2015 (5.2%) to 2017 (5.6%).

Analyses of state and large urban school district data indicated that across 37 states, the overall prevalence of not having eaten fruit or drunk 100% fruit juices ranged from 4.9% to 13.0% across state surveys (median: 7.5%) (Supplementary Table 160). Across 21 large urban school districts, the prevalence ranged from 3.8% to 12.1% (median: 8.1%).

#### Ate Fruit or Drank 100% Fruit Juices One or More Times per Day

Nationwide, 60.8% of students had eaten fruit or drunk 100% fruit juices (e.g., orange juice, apple juice, or grape juice, not counting punch, Kool-Aid, sports drinks, or other fruit-flavored drinks) one or more times per day during the 7 days before the survey (Supplementary Table 161). The prevalence of having eaten fruit or drunk 100% fruit juices one or more times per day was higher among male (63.3%) than female (58.2%) students; higher among white male (62.6%) and Hispanic male (65.3%) than white female (56.7%) and Hispanic female (59.5%) students, respectively; and higher among 10th-grade male (63.7%) and 12th-grade male (63.6%) than 10th-grade female (56.7%) and 12th-grade female (56.6%) students, respectively.

Analyses based on the question ascertaining sexual identity indicated that nationwide, 61.6% of heterosexual students; 56.5% of gay, lesbian, and bisexual students; and 56.2% of not sure students had eaten fruit or drunk 100% fruit juices one or more times per day (Supplementary Table 161). The prevalence of having eaten fruit or drunk 100% fruit juices one or more times per day was higher among heterosexual (61.6%) than gay, lesbian, and bisexual (56.5%) and not sure (56.2%) students. Among female students, the prevalence was higher among heterosexual (59.4%) than not sure (53.0%) students. The prevalence also was higher among heterosexual male (63.5%) than heterosexual female (59.4%) students.

Analyses based on the question ascertaining the sex of sexual contacts indicated that nationwide, 63.0% of students who had sexual contact with only the opposite sex, 59.8% of students who had sexual contact with only the same sex or with both sexes, and 60.6% of students who had no sexual contact had eaten fruit or drunk 100% fruit juices one or more times per day (Supplementary Table 161). The prevalence of having eaten fruit or drunk 100% fruit juices one or more times per day was higher among students who had sexual contact with only the opposite sex (63.0%) than students who had no sexual contact (60.6%). Among male students, the prevalence was higher among those who had sexual contact with only females (67.4%) than those who had no sexual contact (60.8%). The prevalence also was higher among male students who had sexual contact with only females (67.4%) than female students who had sexual contact with only males (57.5%).

Trend analyses did not identify a significant linear trend in the overall prevalence of having eaten fruit or drunk 100% fruit juices one or more times per day during 1999–2017 (62.6%–60.8%). A significant quadratic trend also was not identified. The prevalence of having eaten fruit or drunk 100% fruit juices one or more times per day did not change significantly from 2015 (63.3%) to 2017 (60.8%).

Analyses of state and large urban school district data indicated that across 37 states, the overall prevalence of having eaten fruit or drunk 100% fruit juices one or more times per day ranged from 48.1% to 64.9% across state surveys (median: 57.4%) (Supplementary Table 162). Across 21 large urban school districts, the prevalence ranged from 48.3% to 61.2% (median: 53.8%).

#### Ate Fruit or Drank 100% Fruit Juices Two or More Times per Day

Nationwide, 31.3% of students had eaten fruit or drunk 100% fruit juices (e.g., orange juice, apple juice, or grape juice, not counting punch, Kool-Aid, sports drinks, or other fruit-flavored drinks) two or more times per day during the 7 days before the survey (Supplementary Table 163). The prevalence of having eaten fruit or drunk 100% fruit juices two or more times per day was higher among male (33.8%) than female (28.8%) students; higher among white male (31.5%), black male (40.1%), and Hispanic male (36.2%) than white female (27.4%), black female (33.6%), and Hispanic female (29.7%) students, respectively; and higher among 10th-grade male (37.6%) and 12th-grade male (33.6%) than 10th-grade female (26.4%) and 12th-grade female (28.4%) students, respectively. The prevalence of having eaten fruit or drunk 100% fruit juices two or more times per day was higher among black (36.8%) and Hispanic (33.0%) than white (29.4%) students, higher among black female (33.6%) than white female (27.4%) students, and higher among black male (40.1%) than white male (31.5%) students. The prevalence of having eaten fruit or drunk 100% fruit juices two or more times per day was higher among 9th-grade female (30.3%) than 10th-grade female (26.4%) students and higher among 10th-grade male (37.6%) than 11th-grade male (30.6%) students.

Analyses based on the question ascertaining sexual identity indicated that nationwide, 32.3% of heterosexual students; 26.2% of gay, lesbian, and bisexual students; and 29.1% of not sure students had eaten fruit or drunk 100% fruit juices two or more times per day (Supplementary Table 163). The prevalence of having eaten fruit or drunk 100% fruit juices two or more times per day was higher among heterosexual (32.3%) than gay, lesbian, and bisexual (26.2%) students. Among female students, the prevalence was higher among heterosexual (30.2%) than lesbian and bisexual (25.4%) students. Among male students, the prevalence was higher among heterosexual (34.0%) than gay and bisexual (27.4%) students. The prevalence also was higher among heterosexual male (34.0%) than heterosexual female (30.2%) students.

Analyses based on the question ascertaining the sex of sexual contacts indicated that nationwide, 33.5% of students who had sexual contact with only the opposite sex, 32.0% of students who had sexual contact with only the same sex or with both sexes, and 30.3% of students who had no sexual contact had eaten fruit or drunk 100% fruit juices two or more times per day (Supplementary Table 163). The prevalence of having eaten fruit or drunk 100% fruit juices two or more times per day was higher among students who had sexual contact with only the opposite sex (33.5%) than students who had no sexual contact (30.3%). Among male students, the prevalence was higher among those who had sexual contact with only females (37.9%) than those who had no sexual contact (30.5%). The prevalence also was higher among male students who had sexual contact with only females (37.9%) than female students who had sexual contact with only males (28.2%).

Trend analyses did not identify a significant linear trend in the overall prevalence of having eaten fruit or drunk 100% fruit juices two or more times per day during 1999–2017 (34.8%–31.3%). A significant quadratic trend also was not identified. The prevalence of having eaten fruit or drunk 100% fruit juices two or more times per day did not change significantly from 2015 (31.5%) to 2017 (31.3%).

Analyses of state and large urban school district data indicated that across 37 states, the overall prevalence of having eaten fruit or drunk 100% fruit juices two or more times per day ranged from 20.3% to 33.3% across state surveys (median: 27.5%) (Supplementary Table 164). Across 21 large urban school districts, the prevalence ranged from 23.3% to 34.0% (median: 27.8%).

#### Ate Fruit or Drank 100% Fruit Juices Three or More Times per Day

Nationwide, 18.8% of students had eaten fruit or drunk 100% fruit juices (e.g., orange juice, apple juice, or grape juice, not counting punch, Kool-Aid, sports drinks, or other fruit-flavored drinks) three or more times per day during the 7 days before the survey (Supplementary Table 165). The prevalence of having eaten fruit or drunk 100% fruit juices three or more times per day was higher among male (21.8%) than female (15.9%) students; higher among white male (19.2%), black male (29.2%), and Hispanic male (24.6%) than white female (13.3%), black female (22.3%), and Hispanic female (18.6%) students, respectively; and higher among 9th-grade male (20.9%), 10th-grade male (24.9%), 11th-grade male (20.1%), and 12th-grade male (20.9%) than 9th-grade female (17.0%), 10th-grade female (15.6%), 11th-grade female (16.1%), and 12th-grade female (14.6%) students, respectively. The prevalence of having eaten fruit or drunk 100% fruit juices three or more times per day was higher among black (25.7%) and Hispanic (21.7%) than white (16.1%) students, higher among black (25.7%) than Hispanic (21.7%) students, higher among black female (22.3%) and Hispanic female (18.6%) than white female (13.3%) students, higher among black female (22.3%) than Hispanic female (18.6%) students, higher among black male (29.2%) and Hispanic male (24.6%) than white male (19.2%) students, and higher among black male (29.2%) than Hispanic male (24.6%) students. The prevalence of having eaten fruit or drunk 100% fruit juices three or more times per day was higher among 10th-grade (20.2%) than 11th-grade (18.1%) and 12th-grade (17.6%) students and higher among 10th-grade male (24.9%) than 9th-grade male (20.9%) and 11th-grade male (20.1%) students.

Analyses based on the question ascertaining sexual identity indicated that nationwide, 19.6% of heterosexual students; 15.2% of gay, lesbian, and bisexual students; and 17.9% of not sure students had eaten fruit or drunk 100% fruit juices three or more times per day (Supplementary Table 165). The prevalence of having eaten fruit or drunk 100% fruit juices three or more times per day was higher among heterosexual (19.6%) than gay, lesbian, and bisexual (15.2%) students. The prevalence also was higher among heterosexual male (22.1%) than heterosexual female (16.6%) students.

Analyses based on the question ascertaining the sex of sexual contacts indicated that nationwide, 21.5% of students who had sexual contact with only the opposite sex, 19.9% of students who had sexual contact with only the same sex or with both sexes, and 16.9% of students who had no sexual contact had eaten fruit or drunk 100% fruit juices three or more times per day (Supplementary Table 165). The prevalence of having eaten fruit or drunk 100% fruit juices three or more times per day was higher among students who had sexual contact with only the opposite sex (21.5%) than students who had no sexual contact (16.9%). Among male students, the prevalence was higher among those who had sexual contact with only females (25.9%) and those who had sexual contact with only males or with both sexes (25.2%) than those who had no sexual contact (17.8%). The prevalence also was higher among male students who had sexual contact with only females (25.9%) than female students who had sexual contact with only males (16.2%).

Trend analyses indicated that during 1999–2017, a significant linear decrease (24.9%–18.8%) occurred in the overall prevalence of having eaten fruit or drunk 100% fruit juices three or more times per day. A significant quadratic trend was not identified. The prevalence of having eaten fruit or drunk 100% fruit juices three or more times per day did not change significantly from 2015 (20.0%) to 2017 (18.8%).

Analyses of state and large urban school district data indicated that across 37 states, the overall prevalence of having eaten fruit or drunk 100% fruit juices three or more times per day ranged from 12.0% to 20.2% across state surveys (median: 15.8%) (Supplementary Table 166). Across 21 large urban school districts, the prevalence ranged from 14.8% to 22.8% (median: 17.7%).

#### Did Not Eat Vegetables

Nationwide, 7.2% of students had not eaten vegetables (green salad, potatoes [not counting French fries, fried potatoes, or potato chips], carrots, or other vegetables) during the 7 days before the survey (Supplementary Table 167). The prevalence of not having eaten vegetables was higher among male (8.9%) than female (5.5%) students; higher among white male (6.9%), black male (14.9%), and Hispanic male (11.1%) than white female (3.8%), black female (10.6%), and Hispanic female (7.2%) students, respectively; and higher among 9th-grade male (10.5%), 10th-grade male (8.3%), 11th-grade male (7.8%), and 12th-grade male (8.8%) than 9th-grade female (6.2%), 10th-grade female (5.5%), 11th-grade female (5.6%), and 12th-grade female (4.5%) students, respectively. The prevalence of not having eaten vegetables was higher among black (12.7%) and Hispanic (9.2%) than white (5.3%) students, higher among black (12.7%) than Hispanic (9.2%) students, higher among black female (10.6%) and Hispanic female (7.2%) than white female (3.8%) students, higher among black female (10.6%) than Hispanic female (7.2%) students, higher among black male (14.9%) and Hispanic male (11.1%) than white male (6.9%) students, and higher among black male (14.9%) than Hispanic male (11.1%) students. The prevalence of not having eaten vegetables was higher among 9th-grade (8.3%) than 11th-grade (6.7%) students, higher among 9th-grade female (6.2%) than 12th-grade female (4.5%) students, and higher among 9th-grade male (10.5%) than 11th-grade male (7.8%) students.

Analyses based on the question ascertaining sexual identity indicated that nationwide, 7.5% of heterosexual students; 6.6% of gay, lesbian, and bisexual students; and 7.7% of not sure students had not eaten vegetables (Supplementary Table 167). The prevalence of not having eaten vegetables was higher among heterosexual male (9.1%) than heterosexual female (5.8%) students.

Analyses based on the question ascertaining the sex of sexual contacts indicated that nationwide, 6.9% of students who had sexual contact with only the opposite sex, 5.3% of students who had sexual contact with only the same sex or with both sexes, and 7.4% of students who had no sexual contact had not eaten vegetables (Supplementary Table 167). The prevalence of not having eaten vegetables was higher among male students who had sexual contact with only females (8.2%) than female students who had sexual contact with only males (5.4%) and higher among male students who had no sexual contact (9.1%) than female students who had no sexual contact (5.8%).

Trend analyses indicated that during 1999–2017, a significant linear increase (4.2%–7.2%) occurred in the overall prevalence of not having eaten vegetables. A significant quadratic trend was not identified. The prevalence of not having eaten vegetables did not change significantly from 2015 (6.7%) to 2017 (7.2%).

Analyses of state and large urban school district data indicated that across 33 states, the overall prevalence of not having eaten vegetables ranged from 4.5% to 16.5% across state surveys (median: 7.7%) (Supplementary Table 168). Across 18 large urban school districts, the prevalence ranged from 7.1% to 15.8% (median: 11.8%).

#### Ate Vegetables One or More Times per Day

Nationwide, 59.4% of students had eaten vegetables (green salad, potatoes [not counting French fries, fried potatoes, or potato chips], carrots, or other vegetables) one or more times per day during the 7 days before the survey (Supplementary Table 169). The prevalence of having eaten vegetables one or more times per day was higher among white (62.8%) and Hispanic (56.1%) than black (49.4%) students, higher among white (62.8%) than Hispanic (56.1%) students, higher among white female (64.0%) and Hispanic female (55.2%) than black female (47.4%) students, higher among white female (64.0%) than Hispanic female (55.2%) students, higher among white male (61.5%) and Hispanic male (56.9%) than black male (51.5%) students, and higher among white male (61.5%) than Hispanic male (56.9%) students. The prevalence of having eaten vegetables one or more times per day was higher among 10th-grade (60.8%), 11th-grade (60.4%), and 12th-grade (60.8%) than 9th-grade (56.1%) students; higher among 12th-grade female (62.0%) than 9th-grade female (56.0%) students; and higher among 10th-grade male (61.1%) and 11th-grade male (61.7%) than 9th-grade male (55.9%) students.

Analyses based on the question ascertaining sexual identity indicated that nationwide, 58.9% of heterosexual students; 58.6% of gay, lesbian, and bisexual students; and 66.0% of not sure students had eaten vegetables one or more times per day (Supplementary Table 169). The prevalence of having eaten vegetables one or more times per day was higher among not sure (66.0%) than heterosexual (58.9%) and gay, lesbian, and bisexual (58.6%) students. Among male students, the prevalence was higher among not sure (69.9%) than heterosexual (58.5%) students.

Analyses based on the question ascertaining the sex of sexual contacts indicated that nationwide, 59.9% of students who had sexual contact with only the opposite sex, 64.7% of students who had sexual contact with only the same sex or with both sexes, and 58.6% of students who had no sexual contact had eaten vegetables one or more times per day (Supplementary Table 169). The prevalence of having eaten vegetables one or more times per day was higher among students who had sexual contact with only the same sex or with both sexes (64.7%) than students who had sexual contact with only the opposite sex (59.9%) and students who had no sexual contact (58.6%). Among male students, the prevalence was higher among those who had sexual contact with only females (61.0%) and those who had sexual contact with only males or with both sexes (70.7%) than those who had no sexual contact (57.1%) and higher among those who had sexual contact with only males or with both sexes (70.7%) than those who had sexual contact with only females (61.0%). The prevalence also was higher among male students who had sexual contact with only males or with both sexes (70.7%) than female students who had sexual contact with only females or with both sexes (62.7%).

Trend analyses indicated that during 1999–2017, a significant linear decrease (64.5%–59.4%) occurred in the overall prevalence of having eaten vegetables one or more times per day. A significant quadratic trend was not identified. The prevalence of having eaten vegetables one or more times per day did not change significantly from 2015 (61.0%) to 2017 (59.4%).

Analyses of state and large urban school district data indicated that across 33 states, the overall prevalence of having eaten vegetables one or more times per day ranged from 46.7% to 71.2% across state surveys (median: 57.6%) (Supplementary Table 170). Across 18 large urban school districts, the prevalence ranged from 45.6% to 58.2% (median: 50.1%).

#### Ate Vegetables Two or More Times per Day

Nationwide, 26.6% of students had eaten vegetables (green salad, potatoes [not counting French fries, fried potatoes, or potato chips], carrots, or other vegetables) two or more times per day during the 7 days before the survey (Supplementary Table 171). The prevalence of having eaten vegetables two or more times per day was higher among male (28.7%) than female (24.5%) students; higher among black male (27.4%) and Hispanic male (28.6%) than black female (20.8%) and Hispanic female (23.6%) students, respectively; and higher among 10th-grade male (30.5%) than 10th-grade female (24.3%) students. The prevalence of having eaten vegetables two or more times per day was higher among white female (25.8%) than black female (20.8%) students. The prevalence of having eaten vegetables two or more times per day was higher among 10th-grade (27.3%), 11th-grade (27.5%), and 12th-grade (27.7%) than 9th-grade (24.2%) students; higher among 11th-grade female (25.8%) than 9th-grade female (22.3%) students; and higher among 10th-grade male (30.5%) than 9th-grade male (25.9%) students.

Analyses based on the question ascertaining sexual identity indicated that nationwide, 26.5% of heterosexual students; 26.3% of gay, lesbian, and bisexual students; and 29.2% of not sure students had eaten vegetables two or more times per day (Supplementary Table 171). The prevalence of having eaten vegetables two or more times per day was higher among heterosexual male (27.8%) than heterosexual female (24.9%) students, higher among gay and bisexual male (33.1%) than lesbian and bisexual female (23.9%) students, and higher among not sure male (38.0%) than not sure female (23.6%) students.

Analyses based on the question ascertaining the sex of sexual contacts indicated that nationwide, 27.6% of students who had sexual contact with only the opposite sex, 32.9% of students who had sexual contact with only the same sex or with both sexes, and 24.9% of students who had no sexual contact had eaten vegetables two or more times per day (Supplementary Table 171). The prevalence of having eaten vegetables two or more times per day was higher among students who had sexual contact with only the opposite sex (27.6%) and students who had sexual contact with only the same sex or with both sexes (32.9%) than students who had no sexual contact (24.9%) and higher among students who had sexual contact with only the same sex or with both sexes (32.9%) than students who had sexual contact with only the opposite sex (27.6%). Among female students, the prevalence was higher among those who had sexual contact with only females or with both sexes (28.9%) than those who had sexual contact with only males (24.2%). Among male students, the prevalence was higher among those who had sexual contact with only females (30.5%) and those who had sexual contact with only males or with both sexes (44.8%) than those who had no sexual contact (25.1%) and higher among those who had sexual contact with only males or with both sexes (44.8%) than those who had sexual contact with only females (30.5%). The prevalence also was higher among male students who had sexual contact with only females (30.5%) than female students who had sexual contact with only males (24.2%) and higher among male students who had sexual contact with only males or with both sexes (44.8%) than female students who had sexual contact with only females or with both sexes (28.9%).

Trend analyses did not identify a significant linear trend in the overall prevalence of having eaten vegetables two or more times per day during 1999–2017 (28.5%–26.6%). A significant quadratic trend also was not identified. The prevalence of having eaten vegetables two or more times per day did not change significantly from 2015 (28.0%) to 2017 (26.6%).

Analyses of state and large urban school district data indicated that across 33 states, the overall prevalence of having eaten vegetables two or more times per day ranged from 18.3% to 35.1% across state surveys (median: 24.7%) (Supplementary Table 172). Across 18 large urban school districts, the prevalence ranged from 18.6% to 25.9% (median: 21.2%).

#### Ate Vegetables Three or More Times per Day

Nationwide, 13.9% of students had eaten vegetables (green salad, potatoes [not counting French fries, fried potatoes, or potato chips], carrots, or other vegetables) three or more times per day during the 7 days before the survey (Supplementary Table 173). The prevalence of having eaten vegetables three or more times per day was higher among male (15.9%) than female (12.1%) students; higher among white male (14.4%), black male (19.3%), and Hispanic male (16.2%) than white female (11.4%), black female (12.0%), and Hispanic female (12.5%) students, respectively; and higher among 10th-grade male (17.6%) and 12th-grade male (16.4%) than 10th-grade female (11.0%) and 12th-grade female (13.1%) students, respectively. The prevalence of having eaten vegetables three or more times per day was higher among black (15.6%) than white (12.8%) students and higher among black male (19.3%) than white male (14.4%) students.

Analyses based on the question ascertaining sexual identity indicated that nationwide, 13.8% of heterosexual students; 14.5% of gay, lesbian, and bisexual students; and 17.4% of not sure students had eaten vegetables three or more times per day (Supplementary Table 173). Among male students, the prevalence of having eaten vegetables three or more times per day was higher among gay and bisexual (22.4%) and not sure (25.2%) than heterosexual (15.1%) students. The prevalence also was higher among heterosexual male (15.1%) than heterosexual female (12.4%) students, higher among gay and bisexual male (22.4%) than lesbian and bisexual female (12.1%) students, and higher among not sure male (25.2%) than not sure female (12.3%) students.

Analyses based on the question ascertaining the sex of sexual contacts indicated that nationwide, 15.2% of students who had sexual contact with only the opposite sex, 19.7% of students who had sexual contact with only the same sex or with both sexes, and 12.1% of students who had no sexual contact had eaten vegetables three or more times per day (Supplementary Table 173). The prevalence of having eaten vegetables three or more times per day was higher among students who had sexual contact with only the opposite sex (15.2%) and students who had sexual contact with only the same sex or with both sexes (19.7%) than students who had no sexual contact (12.1%) and higher among students who had sexual contact with only the same sex or with both sexes (19.7%) than students who had sexual contact with only the opposite sex (15.2%). Among female students, the prevalence was higher among those who had sexual contact with only females or with both sexes (16.8%) than those who had sexual contact with only males (12.4%) and those who had no sexual contact (11.6%). Among male students, the prevalence was higher among those who had sexual contact with only females (17.5%) and those who had sexual contact with only males or with both sexes (28.1%) than those who had no sexual contact (12.6%) and higher among those who had sexual contact with only males or with both sexes (28.1%) than those who had sexual contact with only females (17.5%). The prevalence also was higher among male students who had sexual contact with only females (17.5%) than female students who had sexual contact with only males (12.4%) and higher among male students who had sexual contact with only males or with both sexes (28.1%) than female students who had sexual contact with only females or with both sexes (16.8%).

Trend analyses did not identify a significant linear trend in the overall prevalence of having eaten vegetables three or more times per day during 1999–2017 (14.0%–13.9%). A significant quadratic trend also was not identified. The prevalence of having eaten vegetables three or more times per day did not change significantly from 2015 (14.8%) to 2017 (13.9%).

Analyses of state and large urban school district data indicated that across 33 states, the overall prevalence of having eaten vegetables three or more times per day ranged from 9.0% to 18.1% across state surveys (median: 12.3%) (Supplementary Table 174). Across 18 large urban school districts, the prevalence ranged from 9.4% to 14.8% (median: 11.8%).

#### Did Not Drink Milk

Nationwide, 26.7% of students had not drunk milk during the 7 days before the survey (Supplementary Table 175). The prevalence of not having drunk milk was higher among female (33.7%) than male (19.4%) students; higher among white female (31.9%), black female (50.3%), and Hispanic female (27.4%) than white male (18.1%), black male (31.2%), and Hispanic male (15.7%) students, respectively; and higher among 9th-grade female (30.2%), 10th-grade female (33.7%), 11th-grade female (34.0%), and 12th-grade female (37.1%) than 9th-grade male (21.1%), 10th-grade male (15.9%), 11th-grade male (20.0%), and 12th-grade male (20.6%) students, respectively. The prevalence of not having drunk milk was higher among white (25.3%) and black (40.9%) than Hispanic (21.4%) students, higher among black (40.9%) than white (25.3%) students, higher among white female (31.9%) and black female (50.3%) than Hispanic female (27.4%) students, higher among black female (50.3%) than white female (31.9%) students, and higher among black male (31.2%) than white male (18.1%) and Hispanic male (15.7%) students. The prevalence of not having drunk milk was higher among 12th-grade (29.1%) than 9th-grade (25.7%) and 10th-grade (25.0%) students; higher among 12th-grade female (37.1%) than 9th-grade female (30.2%) students; and higher among 9th-grade male (21.1%), 11th-grade male (20.0%), and 12th-grade male (20.6%) than 10th-grade male (15.9%) students.

Analyses based on the question ascertaining sexual identity indicated that nationwide, 25.9% of heterosexual students; 32.4% of gay, lesbian, and bisexual students; and 31.8% of not sure students had not drunk milk (Supplementary Table 175). The prevalence of not having drunk milk was higher among gay, lesbian, and bisexual (32.4%) and not sure (31.8%) than heterosexual (25.9%) students. Among male students, the prevalence was higher among gay and bisexual (29.0%) than heterosexual (18.9%) students. The prevalence also was higher among heterosexual female (34.1%) than heterosexual male (18.9%) students and higher among not sure female (36.8%) than not sure male (21.5%) students.

Analyses based on the question ascertaining the sex of sexual contacts indicated that nationwide, 27.1% of students who had sexual contact with only the opposite sex, 31.8% of students who had sexual contact with only the same sex or with both sexes, and 25.8% of students who had no sexual contact had not drunk milk (Supplementary Table 175). The prevalence of not having drunk milk was higher among students who had sexual contact with only the same sex or with both sexes (31.8%) than students who had sexual contact with only the opposite sex (27.1%) and students who had no sexual contact (25.8%). The prevalence also was higher among female students who had sexual contact with only males (35.7%) than male students who had sexual contact with only females (19.9%), higher among female students who had sexual contact with only females or with both sexes (36.2%) than male students who had sexual contact with only males or with both sexes (19.3%), and higher among female students who had no sexual contact (32.8%) than male students who had no sexual contact (18.2%).

Trend analyses indicated that during 1999–2017, a significant linear increase (17.0%–26.7%) occurred in the overall prevalence of not having drunk milk. A significant quadratic trend also was identified. The prevalence of not having drunk milk increased during 1999–2013 (17.0%–19.4%) and then increased more rapidly during 2013–2017 (19.4%–26.7%). The prevalence of not having drunk milk also increased from 2015 (21.5%) to 2017 (26.7%).

Analyses of state and large urban school district data indicated that across 27 states, the overall prevalence of not having drunk milk ranged from 14.9% to 37.3% across state surveys (median: 25.1%) (Supplementary Table 176). Across 18 large urban school districts, the prevalence ranged from 25.3% to 43.5% (median: 30.9%).

#### Drank One or More Glasses of Milk per Day

Nationwide, 31.3% of students had drunk one or more glasses of milk per day (counting milk in a glass or cup, from a carton, or with cereal and counting the half pint of milk served at school as equal to one glass) during the 7 days before the survey (Supplementary Table 177). The prevalence of having drunk one or more glasses of milk per day was higher among male (40.4%) than female (22.5%) students; higher among white male (44.5%), black male (28.7%), and Hispanic male (38.8%) than white female (24.4%), black female (16.9%), and Hispanic female (23.1%) students, respectively; and higher among 9th-grade male (42.5%), 10th-grade male (42.3%), 11th-grade male (39.2%), and 12th-grade male (37.3%) than 9th-grade female (24.2%), 10th-grade female (24.5%), 11th-grade female (21.5%), and 12th-grade female (19.2%) students, respectively. The prevalence of having drunk one or more glasses of milk per day was higher among white (34.0%) and Hispanic (31.1%) than black (22.7%) students, higher among white female (24.4%) and Hispanic female (23.1%) than black female (16.9%) students, higher among white male (44.5%) and Hispanic male (38.8%) than black male (28.7%) students, and higher among white male (44.5%) than Hispanic male (38.8%) students. The prevalence of having drunk one or more glasses of milk per day was higher among 9th-grade (33.2%) and 10th-grade (33.2%) than 12th-grade (28.0%) students, higher among 10th-grade (33.2%) than 11th-grade (30.2%) students, higher among 9th-grade female (24.2%) and 10th-grade female (24.5%) than 12th-grade female (19.2%) students, higher among 10th-grade female (24.5%) than 11th-grade female (21.5%) students, and higher among 10th-grade male (42.3%) than 12th-grade male (37.3%) students.

Analyses based on the question ascertaining sexual identity indicated that nationwide, 32.1% of heterosexual students; 23.5% of gay, lesbian, and bisexual students; and 28.3% of not sure students had drunk one or more glasses of milk per day (Supplementary Table 177). The prevalence of having drunk one or more glasses of milk per day was higher among heterosexual (32.1%) than gay, lesbian, and bisexual (23.5%) students. Among male students, the prevalence was higher among heterosexual (40.8%) than gay and bisexual (28.0%) students. The prevalence also was higher among heterosexual male (40.8%) than heterosexual female (22.0%) students and higher among not sure male (40.1%) than not sure female (21.7%) students.

Analyses based on the question ascertaining the sex of sexual contacts indicated that nationwide, 30.7% of students who had sexual contact with only the opposite sex, 25.0% of students who had sexual contact with only the same sex or with both sexes, and 32.6% of students who had no sexual contact had drunk one or more glasses of milk per day (Supplementary Table 177). The prevalence of having drunk one or more glasses of milk per day was higher among students who had sexual contact with only the opposite sex (30.7%) and students who had no sexual contact (32.6%) than students who had sexual contact with only the same sex or with both sexes (25.0%). Among female students, the prevalence was higher among those who had no sexual contact (24.1%) than those who had sexual contact with only males (20.1%). The prevalence also was higher among male students who had sexual contact with only females (39.5%) than female students who had sexual contact with only males (20.1%), higher among male students who had sexual contact with only males or with both sexes (37.0%) than female students who had sexual contact with only females or with both sexes (20.8%), and higher among male students who had no sexual contact (41.9%) than female students who had no sexual contact (24.1%).

Trend analyses indicated that during 1999–2017, a significant linear decrease (47.1%–31.3%) occurred in the overall prevalence of having drunk one or more glasses of milk per day. A significant quadratic trend also was identified. The prevalence of having drunk one or more glasses of milk per day decreased during 1999–2013 (47.1%–40.3%) and then decreased more rapidly during 2013–2017 (40.3%–31.3%). The prevalence of having drunk one or more glasses of milk per day decreased from 2015 (37.5%) to 2017 (31.3%).

Analyses of state and large urban school district data indicated that across 27 states, the overall prevalence of having drunk one or more glasses of milk per day ranged from 19.8% to 48.3% across state surveys (median: 28.9%) (Supplementary Table 178). Across 18 large urban school districts, the prevalence ranged from 15.5% to 32.8% (median: 22.8%).

#### Drank Two or More Glasses of Milk per Day

Nationwide, 17.5% of students had drunk two or more glasses of milk per day (counting milk in a glass or cup, from a carton, or with cereal and counting the half pint of milk served at school as equal to one glass) during the 7 days before the survey (Supplementary Table 179). The prevalence of having drunk two or more glasses of milk per day was higher among male (24.7%) than female (10.6%) students; higher among white male (28.2%), black male (17.1%), and Hispanic male (21.9%) than white female (11.4%), black female (9.2%), and Hispanic female (11.0%) students, respectively; and higher among 9th-grade male (26.5%), 10th-grade male (25.5%), 11th-grade male (24.2%), and 12th-grade male (22.0%) than 9th-grade female (12.5%), 10th-grade female (11.1%), 11th-grade female (10.1%), and 12th-grade female (8.4%) students, respectively. The prevalence of having drunk two or more glasses of milk per day was higher among white (19.4%) and Hispanic (16.6%) than black (13.1%) students, higher among white male (28.2%) and Hispanic male (21.9%) than black male (17.1%) students, and higher among white male (28.2%) than Hispanic male (21.9%) students. The prevalence of having drunk two or more glasses of milk per day was higher among 9th-grade (19.4%) and 10th-grade (18.2%) than 12th-grade (15.0%) students, higher among 9th-grade female (12.5%) than 12th-grade female (8.4%) students, and higher among 9th-grade male (26.5%) than 12th-grade male (22.0%) students.

Analyses based on the question ascertaining sexual identity indicated that nationwide, 17.8% of heterosexual students; 14.6% of gay, lesbian, and bisexual students; and 17.9% of not sure students had drunk two or more glasses of milk per day (Supplementary Table 179). The prevalence of having drunk two or more glasses of milk per day was higher among heterosexual (17.8%) than gay, lesbian, and bisexual (14.6%) students. The prevalence also was higher among heterosexual male (24.5%) than heterosexual female (10.0%) students, higher among gay and bisexual male (21.3%) than lesbian and bisexual female (12.3%) students, and higher among not sure male (27.3%) than not sure female (12.5%) students.

Analyses based on the question ascertaining the sex of sexual contacts indicated that nationwide, 17.9% of students who had sexual contact with only the opposite sex, 14.8% of students who had sexual contact with only the same sex or with both sexes, and 17.6% of students who had no sexual contact had drunk two or more glasses of milk per day (Supplementary Table 179). The prevalence of having drunk two or more glasses of milk per day was higher among male students who had sexual contact with only females (24.9%) than female students who had sexual contact with only males (9.6%), higher among male students who had sexual contact with only males or with both sexes (26.1%) than female students who had sexual contact with only females or with both sexes (10.8%), and higher among male students who had no sexual contact (24.3%) than female students who had no sexual contact (11.5%).

Trend analyses indicated that during 1999–2017, a significant linear decrease (33.6%–17.5%) occurred in the overall prevalence of having drunk two or more glasses of milk per day. A significant quadratic trend also was identified. The prevalence of having drunk two or more glasses of milk per day decreased during 1999–2013 (33.6%–25.9%) and then decreased more rapidly during 2013–2017 (25.9%–17.5%). The prevalence of having drunk two or more glasses of milk per day decreased from 2015 (22.4%) to 2017 (17.5%).

Analyses of state and large urban school district data indicated that across 27 states, the overall prevalence of having drunk two or more glasses of milk per day ranged from 10.9% to 33.9% across state surveys (median: 16.7%) (Supplementary Table 180). Across 18 large urban school districts, the prevalence ranged from 7.8% to 17.3% (median: 12.0%).

#### Drank Three or More Glasses of Milk per Day

Nationwide, 7.9% of students had drunk three or more glasses of milk per day (counting milk in a glass or cup, from a carton, or with cereal and counting the half pint of milk served at school as equal to one glass) during the 7 days before the survey (Supplementary Table 181). The prevalence of having drunk three or more glasses of milk per day was higher among male (11.8%) than female (4.1%) students; higher among white male (13.8%), black male (8.8%), and Hispanic male (9.6%) than white female (4.4%), black female (3.5%), and Hispanic female (4.3%) students, respectively; and higher among 9th-grade male (13.1%), 10th-grade male (12.4%), 11th-grade male (12.2%), and 12th-grade male (9.2%) than 9th-grade female (4.4%), 10th-grade female (4.7%), 11th-grade female (3.6%), and 12th-grade female (3.5%) students, respectively. The prevalence of having drunk three or more glasses of milk per day was higher among white (8.9%) than black (6.2%) students and higher among white male (13.8%) than black male (8.8%) and Hispanic male (9.6%) students. The prevalence of having drunk three or more glasses of milk per day was higher among 9th-grade (8.7%) and 10th-grade (8.5%) than 12th-grade (6.3%) students and higher among 9th-grade male (13.1%) than 12th-grade male (9.2%) students.

Analyses based on the question ascertaining sexual identity indicated that nationwide, 7.9% of heterosexual students; 6.6% of gay, lesbian, and bisexual students; and 8.9% of not sure students had drunk three or more glasses of milk per day (Supplementary Table 181). The prevalence of having drunk three or more glasses of milk per day was higher among heterosexual male (11.8%) than heterosexual female (3.5%) students.

Analyses based on the question ascertaining the sex of sexual contacts indicated that nationwide, 8.3% of students who had sexual contact with only the opposite sex, 9.1% of students who had sexual contact with only the same sex or with both sexes, and 7.3% of students who had no sexual contact had drunk three or more glasses of milk per day (Supplementary Table 181). Among female students, the prevalence of having drunk three or more glasses of milk per day was higher among those who had sexual contact with only females or with both sexes (7.1%) than those who had sexual contact with only males (3.6%) and those who had no sexual contact (3.7%). The prevalence also was higher among male students who had sexual contact with only females (12.1%) than female students who had sexual contact with only males (3.6%) and higher among male students who had no sexual contact (11.2%) than female students who had no sexual contact (3.7%).

Trend analyses indicated that during 1999–2017, a significant linear decrease (18.0%–7.9%) occurred in the overall prevalence of having drunk three or more glasses of milk per day. A significant quadratic trend also was identified. The prevalence of having drunk three or more glasses of milk per day decreased during 1999–2013 (18.0%–12.5%) and then decreased more rapidly during 2013–2017 (12.5%–7.9%). The prevalence of having drunk three or more glasses of milk per day decreased from 2015 (10.2%) to 2017 (7.9%).

Analyses of state and large urban school district data indicated that across 27 states, the overall prevalence of having drunk three or more glasses of milk per day ranged from 4.8% to 16.1% across state surveys (median: 8.5%) (Supplementary Table 182). Across 18 large urban school districts, the prevalence ranged from 3.2% to 7.6% (median: 5.5%).

#### Did Not Drink Soda or Pop

Nationwide, 27.8% of students had not drunk soda or pop (e.g., Coke, Pepsi, or Sprite, not counting diet soda or diet pop) during the 7 days before the survey (Supplementary Table 183). The prevalence of not having drunk soda or pop was higher among female (31.4%) than male (24.0%) students; higher among white female (32.9%) than white male (22.7%) students; and higher among 9th-grade female (30.9%), 10th-grade female (30.3%), 11th-grade female (31.7%), and 12th-grade female (32.9%) than 9th-grade male (23.2%), 10th-grade male (22.9%), 11th-grade male (25.1%), and 12th-grade male (25.3%) students, respectively. The prevalence of not having drunk soda or pop was higher among white female (32.9%) than black female (25.6%) students.

Analyses based on the question ascertaining sexual identity indicated that nationwide, 27.0% of heterosexual students; 26.5% of gay, lesbian, and bisexual students; and 30.3% of not sure students had not drunk soda or pop (Supplementary Table 183). Among female students, the prevalence of not having drunk soda or pop was higher among heterosexual (30.7%) and not sure (35.3%) than lesbian and bisexual (26.3%) students. The prevalence also was higher among heterosexual female (30.7%) than heterosexual male (23.7%) students and higher among not sure female (35.3%) than not sure male (23.5%) students.

Analyses based on the question ascertaining the sex of sexual contacts indicated that nationwide, 24.0% of students who had sexual contact with only the opposite sex, 24.0% of students who had sexual contact with only the same sex or with both sexes, and 30.0% of students who had no sexual contact had not drunk soda or pop (Supplementary Table 183). The prevalence of not having drunk soda or pop was higher among students who had no sexual contact (30.0%) than students who had sexual contact with only the opposite sex (24.0%) and students who had sexual contact with only the same sex or with both sexes (24.0%). Among female students, the prevalence was higher among those who had no sexual contact (33.6%) than those who had sexual contact with only males (27.6%) and those who had sexual contact with only females or with both sexes (25.0%). Among male students, the prevalence was higher among those who had no sexual contact (26.1%) than those who had sexual contact with only females (21.0%). The prevalence also was higher among female students who had sexual contact with only males (27.6%) than male students who had sexual contact with only females (21.0%) and higher among female students who had no sexual contact (33.6%) than male students who had no sexual contact (26.1%).

Trend analyses indicated that during 2007–2017, a significant linear increase (18.6%–27.8%) occurred in the overall prevalence of not having drunk soda or pop. A significant quadratic trend was not identified. The prevalence of not having drunk soda or pop did not change significantly from 2015 (26.2%) to 2017 (27.8%).

Analyses of state and large urban school district data indicated that across 36 states, the overall prevalence of not having drunk soda or pop ranged from 21.4% to 38.2% across state surveys (median: 29.1%) (Supplementary Table 184). Across 18 large urban school districts, the prevalence ranged from 21.4% to 36.1% (median: 29.9%).

#### Drank Soda or Pop One or More Times per Day

Nationwide, 18.7% of students had drunk a can, bottle, or glass of soda or pop (e.g., Coke, Pepsi, or Sprite, not counting diet soda or diet pop) one or more times per day during the 7 days before the survey (Supplementary Table 185). The prevalence of having drunk soda or pop one or more times per day was higher among male (22.3%) than female (15.4%) students; higher among white male (24.0%) and Hispanic male (19.9%) than white female (15.5%) and Hispanic female (14.0%) students, respectively; and higher among 9th-grade male (21.5%), 10th-grade male (23.5%), 11th-grade male (21.0%), and 12th-grade male (22.9%) than 9th-grade female (14.3%), 10th-grade female (15.6%), 11th-grade female (15.0%), and 12th-grade female (16.5%) students, respectively. The prevalence of having drunk soda or pop one or more times per day was higher among black (21.5%) than Hispanic (17.0%) students, higher among black female (19.8%) than Hispanic female (14.0%) students, and higher among white male (24.0%) than Hispanic male (19.9%) students.

Analyses based on the question ascertaining sexual identity indicated that nationwide, 19.1% of heterosexual students; 21.1% of gay, lesbian, and bisexual students; and 20.0% of not sure students had drunk soda or pop one or more times per day (Supplementary Table 185). Among female students, the prevalence of having drunk soda or pop one or more times per day was higher among lesbian and bisexual (19.9%) than heterosexual (15.3%) students. The prevalence also was higher among heterosexual male (22.4%) than heterosexual female (15.3%) students and higher among not sure male (28.0%) than not sure female (13.7%) students.

Analyses based on the question ascertaining the sex of sexual contacts indicated that nationwide, 23.9% of students who had sexual contact with only the opposite sex, 25.5% of students who had sexual contact with only the same sex or with both sexes, and 14.6% of students who had no sexual contact had drunk soda or pop one or more times per day (Supplementary Table 185). The prevalence of having drunk soda or pop one or more times per day was higher among students who had sexual contact with only the opposite sex (23.9%) and students who had sexual contact with only the same sex or with both sexes (25.5%) than students who had no sexual contact (14.6%). Among female students, the prevalence was higher among those who had sexual contact with only males (19.1%) and those who had sexual contact with only females or with both sexes (22.9%) than those who had no sexual contact (12.4%). Among male students, the prevalence was higher among those who had sexual contact with only females (27.8%) and those who had sexual contact with only males or with both sexes (32.9%) than those who had no sexual contact (17.0%). The prevalence also was higher among male students who had sexual contact with only females (27.8%) than female students who had sexual contact with only males (19.1%) and higher among male students who had no sexual contact (17.0%) than female students who had no sexual contact (12.4%).

Trend analyses indicated that during 2007–2017, a significant linear decrease (33.8%–18.7%) occurred in the overall prevalence of having drunk soda or pop one or more times per day. A significant quadratic trend was not identified. The prevalence of having drunk soda or pop one or more times per day did not change significantly from 2015 (20.4%) to 2017 (18.7%).

Analyses of state and large urban school district data indicated that across 36 states, the overall prevalence of having drunk soda or pop one or more times per day ranged from 10.2% to 32.0% across state surveys (median: 16.4%) (Supplementary Table 186). Across 18 large urban school districts, the prevalence ranged from 9.4% to 23.4% (median: 15.1%).

#### Drank Soda or Pop Two or More Times per Day

Nationwide, 12.5% of students had drunk a can, bottle, or glass of soda or pop (e.g., Coke, Pepsi, or Sprite, not counting diet soda or diet pop) two or more times per day during the 7 days before the survey (Supplementary Table 187). The prevalence of having drunk soda or pop two or more times per day was higher among male (15.0%) than female (10.0%) students; higher among white male (16.1%) and Hispanic male (12.8%) than white female (9.4%) and Hispanic female (8.8%) students, respectively; and higher among 9th-grade male (14.2%), 10th-grade male (16.5%), 11th-grade male (13.5%), and 12th-grade male (15.9%) than 9th-grade female (9.6%), 10th-grade female (10.1%), 11th-grade female (9.2%), and 12th-grade female (10.8%) students, respectively. The prevalence of having drunk soda or pop two or more times per day was higher among black (16.6%) than white (12.7%) and Hispanic (10.8%) students, higher among black female (16.2%) than white female (9.4%) and Hispanic female (8.8%) students, and higher among white male (16.1%) and black male (17.0%) than Hispanic male (12.8%) students. The prevalence of having drunk soda or pop two or more times per day was higher among 10th-grade (13.2%) and 12th-grade (13.3%) than 11th-grade (11.3%) students and higher among 10th-grade male (16.5%) than 11th-grade male (13.5%) students.

Analyses based on the question ascertaining sexual identity indicated that nationwide, 12.5% of heterosexual students; 15.8% of gay, lesbian, and bisexual students; and 14.2% of not sure students had drunk soda or pop two or more times per day (Supplementary Table 187). The prevalence of having drunk soda or pop two or more times per day was higher among gay, lesbian, and bisexual (15.8%) than heterosexual (12.5%) students. Among female students, the prevalence was higher among lesbian and bisexual (15.3%) than heterosexual (9.6%) and not sure (7.3%) students. The prevalence also was higher among heterosexual male (15.0%) than heterosexual female (9.6%) students and higher among not sure male (23.3%) than not sure female (7.3%) students.

Analyses based on the question ascertaining the sex of sexual contacts indicated that nationwide, 15.8% of students who had sexual contact with only the opposite sex, 19.5% of students who had sexual contact with only the same sex or with both sexes, and 9.2% of students who had no sexual contact had drunk soda or pop two or more times per day (Supplementary Table 187). The prevalence of having drunk soda or pop two or more times per day was higher among students who had sexual contact with only the opposite sex (15.8%) and students who had sexual contact with only the same sex or with both sexes (19.5%) than students who had no sexual contact (9.2%) and higher among students who had sexual contact with only the same sex or with both sexes (19.5%) than students who had sexual contact with only the opposite sex (15.8%). Among female students, the prevalence was higher among those who had sexual contact with only males (11.5%) and those who had sexual contact with only females or with both sexes (18.4%) than those who had no sexual contact (7.9%) and higher among those who had sexual contact with only females or with both sexes (18.4%) than those who had sexual contact with only males (11.5%). Among male students, the prevalence was higher among those who had sexual contact with only females (19.4%) and those who had sexual contact with only males or with both sexes (22.4%) than those who had no sexual contact (10.5%). The prevalence also was higher among male students who had sexual contact with only females (19.4%) than female students who had sexual contact with only males (11.5%) and higher among male students who had no sexual contact (10.5%) than female students who had no sexual contact (7.9%).

Trend analyses indicated that during 2007–2017, a significant linear decrease (24.4%–12.5%) occurred in the overall prevalence of having drunk soda or pop two or more times per day. A significant quadratic trend was not identified. The prevalence of having drunk soda or pop two or more times per day did not change significantly from 2015 (13.0%) to 2017 (12.5%).

Analyses of state and large urban school district data indicated that across 36 states, the overall prevalence of having drunk soda or pop two or more times per day ranged from 5.9% to 21.1% across state surveys (median: 9.7%) (Supplementary Table 188). Across 18 large urban school districts, the prevalence ranged from 4.8% to 16.6% (median: 9.7%).

#### Drank Soda or Pop Three or More Times per Day

Nationwide, 7.1% of students had drunk a can, bottle, or glass of soda or pop (e.g., Coke, Pepsi, or Sprite, not counting diet soda or diet pop) three or more times per day during the 7 days before the survey (Supplementary Table 189). The prevalence of having drunk soda or pop three or more times per day was higher among male (8.7%) than female (5.5%) students; higher among white male (9.3%) and Hispanic male (7.3%) than white female (5.4%) and Hispanic female (4.2%) students, respectively; and higher among 9th-grade male (8.2%), 10th-grade male (9.6%), and 11th-grade male (7.5%) than 9th-grade female (5.3%), 10th-grade female (5.2%), and 11th-grade female (5.4%) students, respectively. The prevalence of having drunk soda or pop three or more times per day was higher among black (9.9%) than white (7.3%) and Hispanic (5.8%) students, higher among black female (8.8%) than white female (5.4%) and Hispanic female (4.2%) students, and higher among black male (11.1%) than Hispanic male (7.3%) students.

Analyses based on the question ascertaining sexual identity indicated that nationwide, 7.1% of heterosexual students; 8.3% of gay, lesbian, and bisexual students; and 9.7% of not sure students had drunk soda or pop three or more times per day (Supplementary Table 189). Among female students, the prevalence of having drunk soda or pop three or more times per day was higher among lesbian and bisexual (8.2%) than heterosexual (5.3%) students. The prevalence also was higher among heterosexual male (8.7%) than heterosexual female (5.3%) students and higher among not sure male (15.1%) than not sure female (5.2%) students.

Analyses based on the question ascertaining the sex of sexual contacts indicated that nationwide, 9.3% of students who had sexual contact with only the opposite sex, 11.9% of students who had sexual contact with only the same sex or with both sexes, and 5.0% of students who had no sexual contact had drunk soda or pop three or more times per day (Supplementary Table 189). The prevalence of having drunk soda or pop three or more times per day was higher among students who had sexual contact with only the opposite sex (9.3%) and students who had sexual contact with only the same sex or with both sexes (11.9%) than students who had no sexual contact (5.0%). Among female students, the prevalence was higher among those who had sexual contact with only males (6.3%) and those who had sexual contact with only females or with both sexes (10.9%) than those who had no sexual contact (4.2%) and higher among those who had sexual contact with only females or with both sexes (10.9%) than those who had sexual contact with only males (6.3%). Among male students, the prevalence was higher among those who had sexual contact with only females (11.8%) and those who had sexual contact with only males or with both sexes (14.6%) than those who had no sexual contact (5.8%). The prevalence also was higher among male students who had sexual contact with only females (11.8%) than female students who had sexual contact with only males (6.3%).

Trend analyses indicated that during 2007–2017, a significant linear decrease (14.4%–7.1%) occurred in the overall prevalence of having drunk soda or pop three or more times per day. A significant quadratic trend was not identified. The prevalence of having drunk soda or pop three or more times per day did not change significantly from 2015 (7.1%) to 2017 (7.1%).

Analyses of state and large urban school district data indicated that across 36 states, the overall prevalence of having drunk soda or pop three or more times per day ranged from 2.5% to 12.0% across state surveys (median: 5.1%) (Supplementary Table 190). Across 18 large urban school districts, the prevalence ranged from 2.2% to 10.5% (median: 6.0%).

#### Did Not Drink a Sports Drink

Nationwide, 47.7% of students had not drunk a sports drink (e.g., Gatorade or Powerade, not counting low-calorie sports drinks such as Propel or G2) during the 7 days before the survey (Supplementary Table 191). The prevalence of not having drunk a sports drink was higher among female (57.7%) than male (37.3%) students; higher among white female (62.2%), black female (49.5%), and Hispanic female (47.5%) than white male (39.4%), black male (28.9%), and Hispanic male (33.7%) students, respectively; and higher among 9th-grade female (53.9%), 10th-grade female (57.2%), 11th-grade female (58.7%), and 12th-grade female (61.6%) than 9th-grade male (36.0%), 10th-grade male (37.9%), 11th-grade male (36.3%), and 12th-grade male (39.1%) students, respectively. The prevalence of not having drunk a sports drink was higher among white (51.3%) than black (39.4%) and Hispanic (40.4%) students, higher among white female (62.2%) than black female (49.5%) and Hispanic female (47.5%) students, higher among white male (39.4%) and Hispanic male (33.7%) than black male (28.9%) students, and higher among white male (39.4%) than Hispanic male (33.7%) students. The prevalence of not having drunk a sports drink was higher among 12th-grade (50.7%) than 9th-grade (45.0%) students, higher among 11th-grade female (58.7%) and 12th-grade female (61.6%) than 9th-grade female (53.9%) students, and higher among 12th-grade female (61.6%) than 10th-grade female (57.2%) students.

Analyses based on the question ascertaining sexual identity indicated that nationwide, 44.7% of heterosexual students; 59.4% of gay, lesbian, and bisexual students; and 57.5% of not sure students had not drunk a sports drink (Supplementary Table 191). The prevalence of not having drunk a sports drink was higher among gay, lesbian, and bisexual (59.4%) and not sure (57.5%) than heterosexual (44.7%) students. Among female students, the prevalence was higher among not sure (67.6%) than heterosexual (55.2%) students. Among male students, the prevalence was higher among gay and bisexual (62.6%) than heterosexual (35.6%) and not sure (42.3%) students. The prevalence also was higher among heterosexual female (55.2%) than heterosexual male (35.6%) students and higher among not sure female (67.6%) than not sure male (42.3%) students.

Analyses based on the question ascertaining the sex of sexual contacts indicated that nationwide, 38.6% of students who had sexual contact with only the opposite sex, 50.8% of students who had sexual contact with only the same sex or with both sexes, and 54.2% of students who had no sexual contact had not drunk a sports drink (Supplementary Table 191). The prevalence of not having drunk a sports drink was higher among students who had sexual contact with only the same sex or with both sexes (50.8%) and students who had no sexual contact (54.2%) than students who had sexual contact with only the opposite sex (38.6%). Among female students, the prevalence was higher among those who had no sexual contact (61.7%) than those who had sexual contact with only males (51.6%) and those who had sexual contact with only females or with both sexes (52.1%). Among male students, the prevalence was higher among those who had sexual contact with only males or with both sexes (47.0%) and those who had no sexual contact (46.2%) than those who had sexual contact with only females (27.9%). The prevalence also was higher among female students who had sexual contact with only males (51.6%) than male students who had sexual contact with only females (27.9%) and higher among female students who had no sexual contact (61.7%) than male students who had no sexual contact (46.2%).

The question measuring the prevalence of not having drunk a sports drink was used for the first time in the 2015 national YRBS. As a result, long-term temporal trends are not available for this variable. The prevalence of not having drunk a sports drink increased from 2015 (42.4%) to 2017 (47.7%).

The question also was not included in the standard questionnaire used in the state and large urban school district surveys in 2017. As a result, the range and median prevalence estimates across states and large urban school districts for the prevalence of not having drunk a sports drink are not available.

#### Drank a Sports Drink One or More Times per Day

Nationwide, 12.4% of students had drunk a can, bottle, or glass of a sports drink (e.g., Gatorade or Powerade, not counting low-calorie sports drinks such as Propel or G2) one or more times per day during the 7 days before the survey (Supplementary Table 192). The prevalence of having drunk a sports drink one or more times per day was higher among male (16.9%) than female (8.2%) students; higher among white male (15.4%), black male (27.6%), and Hispanic male (17.3%) than white female (6.3%), black female (14.8%), and Hispanic female (9.4%) students, respectively; and higher among 9th-grade male (16.7%), 10th-grade male (18.7%), 11th-grade male (14.8%), and 12th-grade male (17.1%) than 9th-grade female (9.5%), 10th-grade female (7.9%), 11th-grade female (7.7%), and 12th-grade female (7.1%) students, respectively. The prevalence of having drunk a sports drink one or more times per day was higher among black (21.1%) and Hispanic (13.5%) than white (10.7%) students, higher among black (21.1%) than Hispanic (13.5%) students, higher among black female (14.8%) and Hispanic female (9.4%) than white female (6.3%) students, higher among black female (14.8%) than Hispanic female (9.4%) students, and higher among black male (27.6%) than white male (15.4%) and Hispanic male (17.3%) students. The prevalence of having drunk a sports drink one or more times per day was higher among 10th-grade male (18.7%) than 11th-grade male (14.8%) students.

Analyses based on the question ascertaining sexual identity indicated that nationwide, 13.2% of heterosexual students; 9.3% of gay, lesbian, and bisexual students; and 11.1% of not sure students had drunk a sports drink one or more times per day (Supplementary Table 192). The prevalence of having drunk a sports drink one or more times per day was higher among heterosexual (13.2%) than gay, lesbian, and bisexual (9.3%) students. Among male students, the prevalence was higher among heterosexual (17.2%) than gay and bisexual (12.0%) students. The prevalence also was higher among heterosexual male (17.2%) than heterosexual female (8.6%) students and higher among not sure male (18.3%) than not sure female (5.9%) students.

Analyses based on the question ascertaining the sex of sexual contacts indicated that nationwide, 16.9% of students who had sexual contact with only the opposite sex, 12.5% of students who had sexual contact with only the same sex or with both sexes, and 8.9% of students who had no sexual contact had drunk a sports drink one or more times per day (Supplementary Table 192). The prevalence of having drunk a sports drink one or more times per day was higher among students who had sexual contact with only the opposite sex (16.9%) than students who had sexual contact with only the same sex or with both sexes (12.5%) and students who had no sexual contact (8.9%). Among female students, the prevalence was higher among those who had sexual contact with only males (10.3%) than those who had no sexual contact (6.6%). Among male students, the prevalence was higher among those who had sexual contact with only females (22.3%) and those who had sexual contact with only males or with both sexes (20.9%) than those who had no sexual contact (11.3%). The prevalence also was higher among male students who had sexual contact with only females (22.3%) than female students who had sexual contact with only males (10.3%), higher among male students who had sexual contact with only males or with both sexes (20.9%) than female students who had sexual contact with only females or with both sexes (9.6%), and higher among male students who had no sexual contact (11.3%) than female students who had no sexual contact (6.6%).

The question measuring the prevalence of having drunk a sports drink one or more times per day was used for the first time in the 2015 national YRBS. As a result, long-term temporal trends are not available for this variable. The prevalence of having drunk a sports drink one or more times per day did not change significantly from 2015 (13.8%) to 2017 (12.4%).

The question also was not included in the standard questionnaire used in the state and large urban school district surveys in 2017. As a result, the range and median prevalence estimates across states and large urban school districts for the prevalence of having drunk a sports drink one or more times per day are not available.

#### Drank a Sports Drink Two or More Times per Day

Nationwide, 7.6% of students had drunk a can, bottle, or glass of a sports drink (e.g., Gatorade or Powerade, not counting low-calorie sports drinks such as Propel or G2) two or more times per day during the 7 days before the survey (Supplementary Table 193). The prevalence of having drunk a sports drink two or more times per day was higher among male (10.7%) than female (4.5%) students; higher among white male (9.7%), black male (18.9%), and Hispanic male (11.2%) than white female (3.6%), black female (8.5%), and Hispanic female (4.9%) students, respectively; and higher among 9th-grade male (11.0%), 10th-grade male (10.9%), 11th-grade male (9.5%), and 12th-grade male (11.3%) than 9th-grade female (4.9%), 10th-grade female (4.6%), 11th-grade female (4.4%), and 12th-grade female (4.0%) students, respectively. The prevalence of having drunk a sports drink two or more times per day was higher among black (13.6%) and Hispanic (8.2%) than white (6.5%) students, higher among black (13.6%) than Hispanic (8.2%) students, higher among black female (8.5%) than white female (3.6%) and Hispanic female (4.9%) students, and higher among black male (18.9%) than white male (9.7%) and Hispanic male (11.2%) students.

Analyses based on the question ascertaining sexual identity indicated that nationwide, 8.0% of heterosexual students; 6.0% of gay, lesbian, and bisexual students; and 8.0% of not sure students had drunk a sports drink two or more times per day (Supplementary Table 193). The prevalence of having drunk a sports drink two or more times per day was higher among heterosexual (8.0%) than gay, lesbian, and bisexual (6.0%) students. The prevalence also was higher among heterosexual male (10.9%) than heterosexual female (4.6%) students.

Analyses based on the question ascertaining the sex of sexual contacts indicated that nationwide, 10.6% of students who had sexual contact with only the opposite sex, 9.2% of students who had sexual contact with only the same sex or with both sexes, and 4.9% of students who had no sexual contact had drunk a sports drink two or more times per day (Supplementary Table 193). The prevalence of having drunk a sports drink two or more times per day was higher among students who had sexual contact with only the opposite sex (10.6%) and students who had sexual contact with only the same sex or with both sexes (9.2%) than students who had no sexual contact (4.9%). Among female students, the prevalence was higher among those who had sexual contact with only males (6.1%) and those who had sexual contact with only females or with both sexes (7.0%) than those who had no sexual contact (3.1%). Among male students, the prevalence was higher among those who had sexual contact with only females (14.4%) and those who had sexual contact with only males or with both sexes (15.6%) than those who had no sexual contact (6.8%). The prevalence also was higher among male students who had sexual contact with only females (14.4%) than female students who had sexual contact with only males (6.1%), higher among male students who had sexual contact with only males or with both sexes (15.6%) than female students who had sexual contact with only females or with both sexes (7.0%), and higher among male students who had no sexual contact (6.8%) than female students who had no sexual contact (3.1%).

The question measuring the prevalence of having drunk a sports drink two or more times per day was used for the first time in the 2015 national YRBS. As a result, long-term temporal trends are not available for this variable. The prevalence of having drunk a sports drink two or more times per day did not change significantly from 2015 (8.3%) to 2017 (7.6%).

The question also was not included in the standard questionnaire used in the state and large urban school district surveys in 2017. As a result, the range and median prevalence estimates across states and large urban school districts for the prevalence of having drunk a sports drink two or more times per day are not available.

#### Drank a Sports Drink Three or More Times per Day

Nationwide, 4.2% of students had drunk a can, bottle, or glass of a sports drink (e.g., Gatorade or Powerade, not counting low-calorie sports drinks such as Propel or G2) three or more times per day during the 7 days before the survey (Supplementary Table 194). The prevalence of having drunk a sports drink three or more times per day was higher among male (5.9%) than female (2.5%) students; higher among white male (5.0%), black male (13.4%), and Hispanic male (5.9%) than white female (2.0%), black female (4.6%), and Hispanic female (2.4%) students, respectively; and higher among 9th-grade male (6.3%), 10th-grade male (5.5%), 11th-grade male (4.9%), and 12th-grade male (6.9%) than 9th-grade female (2.5%), 10th-grade female (2.3%), 11th-grade female (2.4%), and 12th-grade female (2.4%) students, respectively. The prevalence of having drunk a sports drink three or more times per day was higher among black (8.9%) than white (3.4%) and Hispanic (4.2%) students, higher among black female (4.6%) than white female (2.0%) and Hispanic female (2.4%) students, and higher among black male (13.4%) than white male (5.0%) and Hispanic male (5.9%) students.

Analyses based on the question ascertaining sexual identity indicated that nationwide, 4.3% of heterosexual students; 3.0% of gay, lesbian, and bisexual students; and 6.4% of not sure students had drunk a sports drink three or more times per day (Supplementary Table 194). Among male students, the prevalence of having drunk a sports drink three or more times per day was higher among not sure (12.2%) than gay and bisexual (4.5%) students. The prevalence also was higher among heterosexual male (5.9%) than heterosexual female (2.5%) students and higher among not sure male (12.2%) than not sure female (2.5%) students.

Analyses based on the question ascertaining the sex of sexual contacts indicated that nationwide, 6.1% of students who had sexual contact with only the opposite sex, 6.2% of students who had sexual contact with only the same sex or with both sexes, and 2.3% of students who had no sexual contact had drunk a sports drink three or more times per day (Supplementary Table 194). The prevalence of having drunk a sports drink three or more times per day was higher among students who had sexual contact with only the opposite sex (6.1%) and students who had sexual contact with only the same sex or with both sexes (6.2%) than students who had no sexual contact (2.3%). Among female students, the prevalence was higher among those who had sexual contact with only males (3.1%) and those who had sexual contact with only females or with both sexes (4.6%) than those who had no sexual contact (1.5%). Among male students, the prevalence was higher among those who had sexual contact with only females (8.5%) and those who had sexual contact with only males or with both sexes (11.2%) than those who had no sexual contact (3.0%). The prevalence also was higher among male students who had sexual contact with only females (8.5%) than female students who had sexual contact with only males (3.1%) and higher among male students who had no sexual contact (3.0%) than female students who had no sexual contact (1.5%).

The question measuring the prevalence of having drunk a sports drink three or more times per day was used for the first time in the 2015 national YRBS. As a result, long-term temporal trends are not available for this variable. The prevalence of having drunk a sports drink three or more times per day did not change significantly from 2015 (4.8%) to 2017 (4.2%).

The question also was not included in the standard questionnaire used in the state and large urban school district surveys in 2017. As a result, the range and median prevalence estimates across states and large urban school districts for the prevalence of having drunk a sports drink three or more times per day are not available.

#### Did Not Drink Plain Water

Nationwide, 3.8% of students had not drunk plain water (counting tap, bottled, and unflavored sparkling water) during the 7 days before the survey (Supplementary Table 195). The prevalence of not having drunk plain water was higher among male (5.0%) than female (2.7%) students; higher among white male (4.3%) and Hispanic male (5.5%) than white female (1.9%) and Hispanic female (2.3%) students, respectively; and higher among 9th-grade male (5.8%), 11th-grade male (4.2%), and 12th-grade male (5.5%) than 9th-grade female (2.5%), 11th-grade female (2.1%), and 12th-grade female (2.6%) students, respectively. The prevalence of not having drunk plain water was higher among black (6.7%) than white (3.1%) and Hispanic (4.0%) students, higher among black female (5.5%) than white female (1.9%) and Hispanic female (2.3%) students, and higher among black male (8.0%) than white male (4.3%) students.

Analyses based on the question ascertaining sexual identity indicated that nationwide, 3.8% of heterosexual students; 3.5% of gay, lesbian, and bisexual students; and 6.5% of not sure students had not drunk plain water (Supplementary Table 195). The prevalence of not having drunk plain water was higher among not sure (6.5%) than gay, lesbian, and bisexual (3.5%) students. The prevalence also was higher among heterosexual male (4.9%) than heterosexual female (2.6%) students.

Analyses based on the question ascertaining the sex of sexual contacts indicated that nationwide, 3.7% of students who had sexual contact with only the opposite sex, 3.4% of students who had sexual contact with only the same sex or with both sexes, and 3.4% of students who had no sexual contact had not drunk plain water (Supplementary Table 195). The prevalence of not having drunk plain water was higher among male students who had sexual contact with only females (4.7%) than female students who had sexual contact with only males (2.5%) and higher among male students who had no sexual contact (4.4%) than female students who had no sexual contact (2.4%).

The question measuring the prevalence of not having drunk plain water was used for the first time in the 2015 national YRBS. As a result, long-term temporal trends are not available for this variable. The prevalence of not having drunk water did not change significantly from 2015 (3.5%) to 2017 (3.8%).

The question also was not included in the standard questionnaire used in the state and large urban school district surveys in 2017. As a result, the range and median prevalence estimates across states and large urban school districts for the prevalence of not having drunk plain water are not available.

#### Drank Plain Water One or More Times per Day

Nationwide, 75.4% of students had drunk a bottle or glass of plain water (counting tap, bottled, and unflavored sparkling water) one or more times per day during the 7 days before the survey (Supplementary Table 196). The prevalence of having drunk plain water one or more times per day was higher among white (77.8%) and Hispanic (73.4%) than black (67.6%) students, higher among white (77.8%) than Hispanic (73.4%) students, higher among white female (78.5%) than black female (67.6%) and Hispanic female (72.4%) students, and higher among white male (77.3%) and Hispanic male (74.4%) than black male (67.5%) students. The prevalence of having drunk plain water one or more times per day was higher among 11th-grade (76.8%) and 12th-grade (76.8%) than 9th-grade (73.2%) students and higher among 10th-grade male (76.1%), 11th-grade male (77.7%), and 12th-grade male (76.3%) than 9th-grade male (71.9%) students.

Analyses based on the question ascertaining sexual identity indicated that nationwide, 75.3% of heterosexual students; 73.3% of gay, lesbian, and bisexual students; and 70.2% of not sure students had drunk plain water one or more times per day (Supplementary Table 196). The prevalence of having drunk plain water one or more times per day was higher among heterosexual (75.3%) than not sure (70.2%) students. Analyses based on the question ascertaining the sex of sexual contacts indicated that nationwide, 75.1% of students who had sexual contact with only the opposite sex, 74.0% of students who had sexual contact with only the same sex or with both sexes, and 76.0% of students who had no sexual contact had drunk plain water one or more times per day (Supplementary Table 196).

The question measuring the prevalence of having drunk plain water one or more times per day was used for the first time in the 2015 national YRBS. As a result, long-term temporal trends are not available for this variable. The prevalence of having drunk plain water one or more times per day did not change significantly from 2015 (73.6%) to 2017 (75.4%).

The question also was not included in the standard questionnaire used in the state and large urban school district surveys in 2017. As a result, the range and median prevalence estimates across states and large urban school districts for the prevalence of having drunk plain water one or more times per day are not available.

#### Drank Plain Water Two or More Times per Day

Nationwide, 66.8% of students had drunk a bottle or glass of plain water (counting tap, bottled, and unflavored sparkling water) two or more times per day during the 7 days before the survey (Supplementary Table 197). The prevalence of having drunk plain water two or more times per day was higher among Hispanic male (67.7%) than Hispanic female (64.1%) students. The prevalence of having drunk plain water two or more times per day was higher among white (68.0%) and Hispanic (66.0%) than black (61.1%) students, higher among white female (68.0%) than black female (61.6%) students, and higher among white male (68.1%) and Hispanic male (67.7%) than black male (60.6%) students. The prevalence of having drunk plain water two or more times per day was higher among 11th-grade (68.3%) and 12th-grade (68.9%) than 9th-grade (63.9%) students and higher among 10th-grade male (68.6%), 11th-grade male (69.2%), and 12th-grade male (69.7%) than 9th-grade male (62.8%) students.

Analyses based on the question ascertaining sexual identity indicated that nationwide, 66.8% of heterosexual students; 64.4% of gay, lesbian, and bisexual students; and 63.0% of not sure students had drunk plain water two or more times per day (Supplementary Table 197). Analyses based on the question ascertaining the sex of sexual contacts indicated that nationwide, 66.9% of students who had sexual contact with only the opposite sex, 65.7% of students who had sexual contact with only the same sex or with both sexes, and 67.0% of students who had no sexual contact had drunk plain water two or more times per day (Supplementary Table 197).

The question measuring the prevalence of having drunk plain water two or more times per day was used for the first time in the 2015 national YRBS. As a result, long-term temporal trends are not available for this variable. The prevalence of having drunk plain water two or more times per day did not change significantly from 2015 (64.3%) to 2017 (66.8%).

The question also was not included in the standard questionnaire used in the state and large urban school district surveys in 2017. As a result, the range and median prevalence estimates across states and large urban school districts for the prevalence of having drunk plain water two or more times per day are not available.

#### Drank Plain Water Three or More Times per Day

Nationwide, 51.3% of students had drunk a bottle or glass of plain water (counting tap, bottled, and unflavored sparkling water) three or more times per day during the 7 days before the survey (Supplementary Table 198). The prevalence of having drunk plain water three or more times per day was higher among Hispanic (52.5%) than black (47.3%) students and higher among Hispanic male (54.6%) than black male (47.1%) students. The prevalence of having drunk plain water three or more times per day was higher among 12th-grade male (54.1%) than 9th-grade male (48.7%) students.

Analyses based on the question ascertaining sexual identity indicated that nationwide, 51.5% of heterosexual students; 47.3% of gay, lesbian, and bisexual students; and 49.2% of not sure students had drunk plain water three or more times per day (Supplementary Table 198). The prevalence of having drunk plain water three or more times per day was higher among heterosexual (51.5%) than gay, lesbian, and bisexual (47.3%) students. Among male students, the prevalence was higher among heterosexual (52.0%) than gay and bisexual (42.3%) students.

Analyses based on the question ascertaining the sex of sexual contacts indicated that nationwide, 51.8% of students who had sexual contact with only the opposite sex, 48.9% of students who had sexual contact with only the same sex or with both sexes, and 51.2% of students who had no sexual contact had drunk plain water three or more times per day (Supplementary Table 198). Among male students, the prevalence of having drunk plain water three or more times per day was higher among those who had sexual contact with only females (52.9%) than those who had sexual contact with only males or with both sexes (42.0%).

The question measuring the prevalence of having drunk plain water three or more times per day was used for the first time in the 2015 national YRBS. As a result, long-term temporal trends are not available for this variable. The prevalence of having drunk plain water three or more times per day did not change significantly from 2015 (49.5%) to 2017 (51.3%).

The question also was not included in the standard questionnaire used in the state and large urban school district surveys in 2017. As a result, the range and median prevalence estimates across states and large urban school districts for the prevalence of having drunk plain water three or more times per day are not available.

#### Did Not Eat Breakfast

Nationwide, 14.1% of students had not eaten breakfast during the 7 days before the survey (Supplementary Table 199). The prevalence of having not eaten breakfast was higher among 10th-grade female (15.4%) than 10th-grade male (12.0%) students. The prevalence of having not eaten breakfast was higher among Hispanic (16.0%) than white (12.8%) students and higher among Hispanic male (16.4%) than white male (12.4%) students. The prevalence of having not eaten breakfast was higher among 12th-grade male (16.4%) than 9th-grade male (12.9%), 10th-grade male (12.0%), and 11th-grade male (12.9%) students.

Analyses based on the question ascertaining sexual identity indicated that nationwide, 13.9% of heterosexual students; 18.1% of gay, lesbian, and bisexual students; and 16.0% of not sure students had not eaten breakfast (Supplementary Table 199). The prevalence of having not eaten breakfast was higher among gay, lesbian, and bisexual (18.1%) than heterosexual (13.9%) students. Among female students, the prevalence was higher among lesbian and bisexual (18.9%) than heterosexual (14.0%) students.

Analyses based on the question ascertaining the sex of sexual contacts indicated that nationwide, 15.0% of students who had sexual contact with only the opposite sex, 19.5% of students who had sexual contact with only the same sex or with both sexes, and 12.4% of students who had no sexual contact had not eaten breakfast (Supplementary Table 199). The prevalence of having not eaten breakfast was higher among students who had sexual contact with only the opposite sex (15.0%) and students who had sexual contact with only the same sex or with both sexes (19.5%) than students who had no sexual contact (12.4%) and higher among students who had sexual contact with only the same sex or with both sexes (19.5%) than students who had sexual contact with only the opposite sex (15.0%). Among female students, the prevalence was higher among those who had sexual contact with only males (16.0%) and those who had sexual contact with only females or with both sexes (19.5%) than those who had no sexual contact (12.6%). Among male students, the prevalence was higher among those who had sexual contact with only males or with both sexes (19.7%) than those who had sexual contact with only females (14.1%) and those who had no sexual contact (12.2%).

Trend analyses did not identify a significant linear trend in the overall prevalence of having not eaten breakfast during 2011–2017 (13.1%–14.1%). Not enough data points were available to identify a quadratic trend. The prevalence of having not eaten breakfast did not change significantly from 2015 (13.8%) to 2017 (14.1%).

Analyses of state and large urban school district data indicated that across 33 states, the overall prevalence of having not eaten breakfast ranged from 10.5% to 24.5% across state surveys (median: 14.6%) (Supplementary Table 200). Across 20 large urban school districts, the prevalence ranged from 12.2% to 22.0% (median: 17.7%).

#### Ate Breakfast on All 7 Days

Nationwide, 35.3% of students had eaten breakfast on all 7 days during the 7 days before the survey (Supplementary Table 201). The prevalence of having eaten breakfast on all 7 days was higher among male (39.9%) than female (31.0%) students; higher among white male (43.4%) and black male (35.1%) than white female (33.2%) and black female (22.7%) students, respectively; and higher among 9th-grade male (43.8%), 10th-grade male (44.1%), 11th-grade male (36.4%), and 12th-grade male (34.3%) than 9th-grade female (32.8%), 10th-grade female (31.2%), 11th-grade female (29.5%), and 12th-grade female (30.2%) students, respectively. The prevalence of having eaten breakfast on all 7 days was higher among white (38.1%) than black (28.7%) and Hispanic (31.7%) students, higher among white female (33.2%) and Hispanic female (29.8%) than black female (22.7%) students, and higher among white male (43.4%) than black male (35.1%) and Hispanic male (33.6%) students. The prevalence of having eaten breakfast on all 7 days was higher among 9th-grade (38.1%) and 10th-grade (37.5%) than 11th-grade (32.8%) and 12th-grade (32.1%) students and higher among 9th-grade male (43.8%) and 10th-grade male (44.1%) than 11th-grade male (36.4%) and 12th-grade male (34.3%) students.

Analyses based on the question ascertaining sexual identity indicated that nationwide, 36.6% of heterosexual students; 24.6% of gay, lesbian, and bisexual students; and 33.8% of not sure students had eaten breakfast on all 7 days (Supplementary Table 201). The prevalence of having eaten breakfast on all 7 days was higher among heterosexual (36.6%) and not sure (33.8%) than gay, lesbian, and bisexual (24.6%) students. Among female students, the prevalence was higher among heterosexual (32.1%) and not sure (33.1%) than lesbian and bisexual (23.4%) students. Among male students, the prevalence was higher among heterosexual (40.7%) than gay and bisexual (28.9%) students. The prevalence also was higher among heterosexual male (40.7%) than heterosexual female (32.1%) students.

Analyses based on the question ascertaining the sex of sexual contacts indicated that nationwide, 31.5% of students who had sexual contact with only the opposite sex, 22.1% of students who had sexual contact with only the same sex or with both sexes, and 41.3% of students who had no sexual contact had eaten breakfast on all 7 days (Supplementary Table 201). The prevalence of having eaten breakfast on all 7 days was higher among students who had no sexual contact (41.3%) than students who had sexual contact with only the opposite sex (31.5%) and students who had sexual contact with only the same sex or with both sexes (22.1%) and higher among students who had sexual contact with only the opposite sex (31.5%) than students who had sexual contact with only the same sex or with both sexes (22.1%). Among female students, the prevalence was higher among those who had no sexual contact (36.5%) than those who had sexual contact with only males (26.3%) and those who had sexual contact with only females or with both sexes (21.3%). Among male students, the prevalence was higher among those who had no sexual contact (46.5%) than those who had sexual contact with only females (35.7%) and those who had sexual contact with only males or with both sexes (24.6%) and higher among those who had sexual contact with only females (35.7%) than those who had sexual contact with only males or with both sexes (24.6%). The prevalence also was higher among male students who had sexual contact with only females (35.7%) than female students who had sexual contact with only males (26.3%) and higher among male students who had no sexual contact (46.5%) than female students who had no sexual contact (36.5%).

Trend analyses did not identify a significant linear trend in the overall prevalence of having eaten breakfast on all 7 days during 2011–2017 (37.7%–35.3%). Not enough data points were available to identify a quadratic trend. The prevalence of having eaten breakfast on all 7 days did not change significantly from 2015 (36.3%) to 2017 (35.3%).

Analyses of state and large urban school district data indicated that across 33 states, the overall prevalence of having eaten breakfast on all 7 days ranged from 20.9% to 39.7% across state surveys (median: 34.6%) (Supplementary Table 202). Across 20 large urban school districts, the prevalence ranged from 16.3% to 40.3% (median: 27.8%).

### Physical Activity

#### Were Not Physically Active for a Total of at Least 60 Minutes on at Least 1 Day

Nationwide, 15.4% of students had not been physically active for a total of at least 60 minutes on at least 1 day during the 7 days before the survey (adding up time spent in any kind of physical activity that increased their heart rate and made them breathe hard some of the time) (Supplementary Table 203). The prevalence of not having been physically active for a total of at least 60 minutes on at least 1 day was higher among female (19.5%) than male (11.0%) students; higher among white female (16.7%), black female (26.6%), and Hispanic female (20.0%) than white male (10.2%), black male (12.7%), and Hispanic male (12.3%) students, respectively; and higher among 9th-grade female (12.9%), 10th-grade female (19.1%), 11th-grade female (23.0%), and 12th-grade female (23.7%) than 9th-grade male (8.1%), 10th-grade male (10.7%), 11th-grade male (12.3%), and 12th-grade male (13.5%) students, respectively. The prevalence of not having been physically active for a total of at least 60 minutes on at least 1 day was higher among black (19.8%) than white (13.6%) students and higher among black female (26.6%) than white female (16.7%) students. The prevalence of not having been physically active for a total of at least 60 minutes on at least 1 day was higher among 10th-grade (14.9%), 11th-grade (17.7%), and 12th-grade (18.7%) than 9th-grade (10.5%) students; higher among 12th-grade (18.7%) than 10th-grade (14.9%) students; higher among 10th-grade female (19.1%), 11th-grade female (23.0%), and 12th-grade female (23.7%) than 9th-grade female (12.9%) students; higher among 10th-grade male (10.7%), 11th-grade male (12.3%), and 12th-grade male (13.5%) than 9th-grade male (8.1%) students; and higher among 12th-grade male (13.5%) than 10th-grade male (10.7%) students.

Analyses based on the question ascertaining sexual identity indicated that nationwide, 13.9% of heterosexual students; 20.8% of gay, lesbian, and bisexual students; and 23.1% of not sure students had not been physically active for a total of at least 60 minutes on at least 1 day (Supplementary Table 203). The prevalence of not having been physically active for a total of at least 60 minutes on at least 1 day was higher among gay, lesbian, and bisexual (20.8%) and not sure (23.1%) than heterosexual (13.9%) students. Among female students, the prevalence was higher among lesbian and bisexual (21.5%) than heterosexual (18.1%) students. Among male students, the prevalence was higher among gay and bisexual (19.4%) and not sure (25.6%) than heterosexual (10.1%) students. The prevalence also was higher among heterosexual female (18.1%) than heterosexual male (10.1%) students.

Analyses based on the question ascertaining the sex of sexual contacts indicated that nationwide, 13.1% of students who had sexual contact with only the opposite sex, 20.9% of students who had sexual contact with only the same sex or with both sexes, and 14.4% of students who had no sexual contact had not been physically active for a total of at least 60 minutes on at least 1 day (Supplementary Table 203). The prevalence of not having been physically active for a total of at least 60 minutes on at least 1 day was higher among students who had sexual contact with only the same sex or with both sexes (20.9%) than students who had sexual contact with only the opposite sex (13.1%) and students who had no sexual contact (14.4%). Among female students, the prevalence was higher among those who had sexual contact with only females or with both sexes (22.3%) than those who had no sexual contact (16.8%). Among male students, the prevalence was higher among those who had sexual contact with only males or with both sexes (16.7%) and those who had no sexual contact (11.8%) than those who had sexual contact with only females (8.8%). The prevalence also was higher among female students who had sexual contact with only males (18.4%) than male students who had sexual contact with only females (8.8%) and higher among female students who had no sexual contact (16.8%) than male students who had no sexual contact (11.8%).

Trend analyses did not identify a significant linear trend in the overall prevalence of not having been physically active for a total of at least 60 minutes on at least 1 day during 2011–2017 (13.8%–15.4%). Not enough data points were available to identify a quadratic trend. The prevalence of not having been physically active for a total of at least 60 minutes on at least 1 day did not change significantly from 2015 (14.3%) to 2017 (15.4%).

Analyses of state and large urban school district data indicated that across 39 states, the overall prevalence of not having been physically active for a total of at least 60 minutes on at least 1 day ranged from 11.1% to 28.2% across state surveys (median: 15.9%) (Supplementary Table 204). Across 21 large urban school districts, the prevalence ranged from 14.2% to 29.8% (median: 22.8%).

#### Were Physically Active for a Total of at Least 60 Minutes per Day on 5 or More Days

Nationwide, 46.5% of students had been physically active for a total of at least 60 minutes per day on 5 or more days during the 7 days before the survey (adding up time spent in any kind of physical activity that increased their heart rate and made them breathe hard some of the time) (Supplementary Table 205). The prevalence of having been physically active for a total of at least 60 minutes per day on 5 or more days was higher among male (56.9%) than female (36.8%) students; higher among white male (59.4%), black male (54.5%), and Hispanic male (52.6%) than white female (38.8%), black female (29.9%), and Hispanic female (36.9%) students, respectively; and higher among 9th-grade male (63.1%), 10th-grade male (56.4%), 11th-grade male (56.3%), and 12th-grade male (51.2%) than 9th-grade female (45.3%), 10th-grade female (34.2%), 11th-grade female (34.6%), and 12th-grade female (32.2%) students, respectively. The prevalence of having been physically active for a total of at least 60 minutes per day on 5 or more days was higher among white (48.7%) than black (42.0%) students, higher among white female (38.8%) and Hispanic female (36.9%) than black female (29.9%) students, and higher among white male (59.4%) than Hispanic male (52.6%) students. The prevalence of having been physically active for a total of at least 60 minutes per day on 5 or more days was higher among 9th-grade (54.1%) than 10th-grade (45.0%), 11th-grade (45.1%), and 12th-grade (41.4%) students; higher among 10th-grade (45.0%) and 11th-grade (45.1%) than 12th-grade (41.4%) students; higher among 9th-grade female (45.3%) than 10th-grade female (34.2%), 11th-grade female (34.6%), and 12th-grade female (32.2%) students; higher among 9th-grade male (63.1%) than 10th-grade male (56.4%), 11th-grade male (56.3%), and 12th-grade male (51.2%) students; and higher among 10th-grade male (56.4%) and 11th-grade male (56.3%) than 12th-grade male (51.2%) students.

Analyses based on the question ascertaining sexual identity indicated that nationwide, 49.6% of heterosexual students; 32.2% of gay, lesbian, and bisexual students; and 34.2% of not sure students had been physically active for a total of at least 60 minutes per day on 5 or more days (Supplementary Table 205). The prevalence of having been physically active for a total of at least 60 minutes per day on 5 or more days was higher among heterosexual (49.6%) than gay, lesbian, and bisexual (32.2%) and not sure (34.2%) students. Among female students, the prevalence was higher among heterosexual (39.4%) than lesbian and bisexual (31.5%) students. Among male students, the prevalence was higher among heterosexual (58.7%) than gay and bisexual (33.6%) and not sure (35.5%) students. The prevalence also was higher among heterosexual male (58.7%) than heterosexual female (39.4%) students.

Analyses based on the question ascertaining the sex of sexual contacts indicated that nationwide, 52.7% of students who had sexual contact with only the opposite sex, 34.8% of students who had sexual contact with only the same sex or with both sexes, and 46.2% of students who had no sexual contact had been physically active for a total of at least 60 minutes per day on 5 or more days (Supplementary Table 205). The prevalence of having been physically active for a total of at least 60 minutes per day on 5 or more days was higher among students who had sexual contact with only the opposite sex (52.7%) and students who had no sexual contact (46.2%) than students who had sexual contact with only the same sex or with both sexes (34.8%) and higher among students who had sexual contact with only the opposite sex (52.7%) than students who had no sexual contact (46.2%). Among female students, the prevalence was higher among those who had sexual contact with only males (39.3%) and those who had no sexual contact (39.7%) than those who had sexual contact with only females or with both sexes (32.5%). Among male students, the prevalence was higher among those who had sexual contact with only females (63.8%) and those who had no sexual contact (53.2%) than those who had sexual contact with only males or with both sexes (41.5%) and higher among those who had sexual contact with only females (63.8%) than those who had no sexual contact (53.2%). The prevalence also was higher among male students who had sexual contact with only females (63.8%) than female students who had sexual contact with only males (39.3%) and higher among male students who had no sexual contact (53.2%) than female students who had no sexual contact (39.7%).

Trend analyses did not identify a significant linear trend in the overall prevalence of having been physically active for a total of at least 60 minutes per day on 5 or more days during 2011–2017 (49.5%–46.5%). Not enough data points were available to identify a quadratic trend. The prevalence of having been physically active for a total of at least 60 minutes per day on 5 or more days did not change significantly from 2015 (48.6%) to 2017 (46.5%).

Analyses of state and large urban school district data indicated that across 39 states, the overall prevalence of having been physically active for a total of at least 60 minutes per day on 5 or more days ranged from 35.1% to 53.4% across state surveys (median: 45.6%) (Supplementary Table 206). Across 21 large urban school districts, the prevalence ranged from 25.5% to 48.5% (median: 33.6%).

#### Were Physically Active for a Total of at Least 60 Minutes per Day on All 7 Days

Nationwide, 26.1% of students had been physically active for a total of at least 60 minutes per day on all 7 days during the 7 days before the survey (calculated by adding up time spent in any kind of physical activity that increased their heart rate and made them breathe hard some of the time) (Supplementary Table 207). The prevalence of having been physically active for a total of at least 60 minutes per day on all 7 days was higher among male (35.3%) than female (17.5%) students; higher among white male (36.7%), black male (33.7%), and Hispanic male (33.3%) than white female (18.4%), black female (15.5%), and Hispanic female (18.1%) students, respectively; and higher among 9th-grade male (39.7%), 10th-grade male (36.7%), 11th-grade male (34.5%), and 12th-grade male (29.8%) than 9th-grade female (22.0%), 10th-grade female (15.2%), 11th-grade female (15.9%), and 12th-grade female (16.4%) students, respectively. The prevalence of having been physically active for a total of at least 60 minutes per day on all 7 days was higher among 9th-grade (30.6%) than 10th-grade (25.6%), 11th-grade (24.9%), and 12th-grade (22.9%) students; higher among 10th-grade (25.6%) than 12th-grade (22.9%) students; higher among 9th-grade female (22.0%) than 10th-grade female (15.2%), 11th-grade female (15.9%), and 12th-grade female (16.4%) students; higher among 9th-grade male (39.7%) and 10th-grade male (36.7%) than 12th-grade male (29.8%) students; and higher among 9th-grade male (39.7%) than 11th-grade male (34.5%) students.

Analyses based on the question ascertaining sexual identity indicated that nationwide, 28.5% of heterosexual students; 14.7% of gay, lesbian, and bisexual students; and 19.0% of not sure students had been physically active for a total of at least 60 minutes per day on all 7 days (Supplementary Table 207). The prevalence of having been physically active for a total of at least 60 minutes per day on all 7 days was higher among heterosexual (28.5%) than gay, lesbian, and bisexual (14.7%) and not sure (19.0%) students. Among female students, the prevalence was higher among heterosexual (19.0%) than lesbian and bisexual (14.3%) students. Among male students, the prevalence was higher among heterosexual (37.0%) than gay and bisexual (15.0%) and not sure (24.1%) students. The prevalence also was higher among heterosexual male (37.0%) than heterosexual female (19.0%) students and higher among not sure male (24.1%) than not sure female (16.1%) students.

Analyses based on the question ascertaining the sex of sexual contacts indicated that nationwide, 31.6% of students who had sexual contact with only the opposite sex, 16.2% of students who had sexual contact with only the same sex or with both sexes, and 24.9% of students who had no sexual contact had been physically active for a total of at least 60 minutes per day on all 7 days (Supplementary Table 207). The prevalence of having been physically active for a total of at least 60 minutes per day on all 7 days was higher among students who had sexual contact with only the opposite sex (31.6%) than students who had sexual contact with only the same sex or with both sexes (16.2%) and students who had no sexual contact (24.9%) and higher among students who had no sexual contact (24.9%) than students who had sexual contact with only the same sex or with both sexes (16.2%). Among male students, the prevalence was higher among those who had sexual contact with only females (41.9%) than those who had sexual contact with only males or with both sexes (19.5%) and those who had no sexual contact (31.6%) and higher among those who had no sexual contact (31.6%) than those who had sexual contact with only males or with both sexes (19.5%). The prevalence also was higher among male students who had sexual contact with only females (41.9%) than female students who had sexual contact with only males (19.2%) and higher among male students who had no sexual contact (31.6%) than female students who had no sexual contact (18.7%).

Trend analyses did not identify a significant linear trend in the overall prevalence of having been physically active for a total of at least 60 minutes per day on all 7 days during 2011–2017 (28.7%–26.1%). Not enough data points were available to identify a quadratic trend. The prevalence of having been physically active for a total of at least 60 minutes per day on all 7 days did not change significantly from 2015 (27.1%) to 2017 (26.1%).

Analyses of state and large urban school district data indicated that across 39 states, the overall prevalence of having been physically active for a total of at least 60 minutes per day on all 7 days ranged from 17.9% to 30.8% across state surveys (median: 23.4%) (Supplementary Table 208). Across 21 large urban school districts, the prevalence ranged from 13.4% to 24.0% (median: 18.0%).

#### Did Exercises to Strengthen or Tone Muscles on 3 or More Days

Nationwide, 51.1% of students had done exercises to strengthen or tone their muscles (e.g., push-ups, sit-ups, or weightlifting) on 3 or more days during the 7 days before the survey (Supplementary Table 209). The prevalence of having done exercises to strengthen or tone their muscles on 3 or more days was higher among male (62.1%) than female (40.8%) students; higher among white male (61.2%), black male (65.9%), and Hispanic male (60.9%) than white female (41.2%), black female (36.2%), and Hispanic female (43.1%) students, respectively; and higher among 9th-grade male (66.4%), 10th-grade male (63.8%), 11th-grade male (60.2%), and 12th-grade male (56.6%) than 9th-grade female (49.3%), 10th-grade female (39.8%), 11th-grade female (36.8%), and 12th-grade female (36.1%) students, respectively. The prevalence of having done exercises to strengthen or tone their muscles on 3 or more days was higher among Hispanic female (43.1%) than black female (36.2%) students and higher among black male (65.9%) than white male (61.2%) students. The prevalence of having done exercises to strengthen or tone their muscles on 3 or more days was higher among 9th-grade (57.6%) than 10th-grade (51.5%), 11th-grade (48.2%), and 12th-grade (46.0%) students; higher among 9th-grade female (49.3%) than 10th-grade female (39.8%), 11th-grade female (36.8%), and 12th-grade female (36.1%) students; higher among 9th-grade male (66.4%) and 10th-grade male (63.8%) than 12th-grade male (56.6%) students; and higher among 9th-grade male (66.4%) than 11th-grade male (60.2%) students.

Analyses based on the question ascertaining sexual identity indicated that nationwide, 54.1% of heterosexual students; 36.4% of gay, lesbian, and bisexual students; and 39.4% of not sure students had done exercises to strengthen or tone their muscles on 3 or more days (Supplementary Table 209). The prevalence of having done exercises to strengthen or tone their muscles on 3 or more days was higher among heterosexual (54.1%) than gay, lesbian, and bisexual (36.4%) and not sure (39.4%) students. Among female students, the prevalence was higher among heterosexual (43.7%) than lesbian and bisexual (34.5%) and not sure (35.7%) students. Among male students, the prevalence was higher among heterosexual (63.2%) than gay and bisexual (42.4%) and not sure (46.3%) students. The prevalence also was higher among heterosexual male (63.2%) than heterosexual female (43.7%) students and higher among not sure male (46.3%) than not sure female (35.7%) students.

Analyses based on the question ascertaining the sex of sexual contacts indicated that nationwide, 56.9% of students who had sexual contact with only the opposite sex, 38.7% of students who had sexual contact with only the same sex or with both sexes, and 49.2% of students who had no sexual contact had done exercises to strengthen or tone their muscles on 3 or more days (Supplementary Table 209). The prevalence of having done exercises to strengthen or tone their muscles on 3 or more days was higher among students who had sexual contact with only the opposite sex (56.9%) than students who had sexual contact with only the same sex or with both sexes (38.7%) and students who had no sexual contact (49.2%) and higher among students who had no sexual contact (49.2%) than students who had sexual contact with only the same sex or with both sexes (38.7%). Among female students, the prevalence was higher among those who had sexual contact with only males (42.4%) and those who had no sexual contact (43.6%) than those who had sexual contact with only females or with both sexes (33.7%). Among male students, the prevalence was higher among those who had sexual contact with only females (69.0%) than those who had sexual contact with only males or with both sexes (54.8%) and those who had no sexual contact (55.1%). The prevalence also was higher among male students who had sexual contact with only females (69.0%) than female students who had sexual contact with only males (42.4%), higher among male students who had sexual contact with only males or with both sexes (54.8%) than female students who had sexual contact with only females or with both sexes (33.7%), and higher among male students who had no sexual contact (55.1%) than female students who had no sexual contact (43.6%).

Trend analyses indicated that during 1991–2017, a significant linear increase occurred in the overall prevalence of having done exercises to strengthen or tone their muscles on 3 or more days (47.8%–51.1%). A significant quadratic trend also was identified. The prevalence of having done exercises to strengthen or tone their muscles on 3 or more days increased during 1991–2011 (47.8%–55.6%) and then did not change significantly during 2011–2017 (55.6%–51.1%). The prevalence of having done exercises to strengthen or tone their muscles on 3 or more days did not change significantly from 2015 (53.4%) to 2017 (51.1%).

The question measuring the prevalence of having done exercises to strengthen or tone their muscles on 3 or more days was not included in the standard questionnaire used in the state and large urban school district surveys in 2017. As a result, the range and median prevalence estimates across states and large urban school districts for the prevalence of having done exercises to strengthen or tone their muscles on 3 or more days are not available.

#### Played Video or Computer Games or Used a Computer 3 or More Hours per Day

Nationwide, 43.0% of students played video or computer games or used a computer 3 or more hours per day on an average school day for something that was not school work (counting “time spent on things such as Xbox, PlayStation, an iPad or other tablet, a smartphone, texting, YouTube, Instagram, Facebook, or other social media”) (Supplementary Table 210). The prevalence of playing video or computer games or using a computer 3 or more hours per day was higher among black (47.2%) and Hispanic (45.4%) than white (40.7%) students, higher among black female (46.7%) and Hispanic female (46.8%) than white female (39.6%) students, and higher among black male (47.7%) than white male (41.7%) students. The prevalence of playing video or computer games or using a computer 3 or more hours per day was higher among 9th-grade (45.0%) and 10th-grade (45.1%) than 12th-grade (39.2%) students; higher among 9th-grade female (44.0%), 10th-grade female (46.5%), and 11th-grade female (43.4%) than 12th-grade female (37.5%) students; and higher among 9th-grade male (45.7%) than 12th-grade male (40.8%) students.

Analyses based on the question ascertaining sexual identity indicated that nationwide, 42.6% of heterosexual students; 52.9% of gay, lesbian, and bisexual students; and 47.4% of not sure students had played video or computer games or using a computer 3 or more hours per day (Supplementary Table 210). The prevalence of playing video or computer games or using a computer 3 or more hours per day was higher among gay, lesbian, and bisexual (52.9%) than heterosexual (42.6%) students. Among female students, the prevalence was higher among lesbian and bisexual (51.5%) than heterosexual (42.8%) students. Among male students, the prevalence was higher among gay and bisexual (57.4%) than heterosexual (42.6%) students.

Analyses based on the question ascertaining the sex of sexual contacts indicated that nationwide, 43.1% of students who had sexual contact with only the opposite sex, 51.9% of students who had sexual contact with only the same sex or with both sexes, and 44.3% of students who had no sexual contact played video or computer games or used a computer 3 or more hours per day (Supplementary Table 210). The prevalence of playing video or computer games or using a computer 3 or more hours per day was higher among students who had sexual contact with only the same sex or with both sexes (51.9%) than students who had sexual contact with only the opposite sex (43.1%) and students who had no sexual contact (44.3%). Among female students, the prevalence was higher among those who had sexual contact with only males (46.0%) and those who had sexual contact with only females or with both sexes (51.6%) than those who had no sexual contact (42.4%). Among male students, the prevalence was higher among those who had sexual contact with only males or with both sexes (52.8%) and those who had no sexual contact (46.3%) than those who had sexual contact with only females (40.7%). The prevalence also was higher among female students who had sexual contact with only males (46.0%) than male students who had sexual contact with only females (40.7%) and higher among male students who had no sexual contact (46.3%) than female students who had no sexual contact (42.4%).

Trend analyses indicated that during 2003–2017, a significant linear increase (22.1%–43.0%) occurred in the overall prevalence of playing video or computer games or using a computer 3 or more hours per day. A significant quadratic trend was not identified. The prevalence of playing video or computer games or using a computer 3 or more hours per day did not change significantly from 2015 (41.7%) to 2017 (43.0%).

Analyses of state and large urban school district data indicated that across 37 states, the overall prevalence of playing video or computer games or using a computer 3 or more hours per day ranged from 33.7% to 47.9% across state surveys (median: 41.2%) (Supplementary Table 211). Across 20 large urban school districts, the prevalence ranged from 38.0% to 49.7% (median: 40.6%).

#### Watched Television 3 or More Hours per Day

Nationwide, 20.7% of students watched television 3 or more hours per day on an average school day (Supplementary Table 212). The prevalence of watching television 3 or more hours per day was higher among black (35.2%) and Hispanic (20.7%) than white (17.7%) students, higher among black (35.2%) than Hispanic (20.7%) students, higher among black female (32.8%) than white female (18.4%) and Hispanic female (19.5%) students, higher among black male (37.8%) and Hispanic male (21.9%) than white male (16.9%) students, and higher among black male (37.8%) than Hispanic male (21.9%) students. The prevalence of watching television 3 or more hours per day was higher among 10th-grade female (22.7%) than 12th-grade female (18.6%) students.

Analyses based on the question ascertaining sexual identity indicated that nationwide, 20.5% of heterosexual students; 25.6% of gay, lesbian, and bisexual students; and 24.4% of not sure students watched television 3 or more hours per day (Supplementary Table 212). The prevalence of watching television 3 or more hours per day was higher among gay, lesbian, and bisexual (25.6%) than heterosexual (20.5%) students. Among female students, the prevalence was higher among lesbian and bisexual (27.2%) than heterosexual (20.2%) students.

Analyses based on the question ascertaining the sex of sexual contacts indicated that nationwide, 21.9% of students who had sexual contact with only the opposite sex, 23.6% of students who had sexual contact with only the same sex or with both sexes, and 19.9% of students who had no sexual contact watched television 3 or more hours per day (Supplementary Table 212). The prevalence of watching television 3 or more hours per day was higher among students who had sexual contact with only the opposite sex (21.9%) than students who had no sexual contact (19.9%). Among male students, the prevalence was higher among those who had sexual contact with only females (22.5%) than those who had no sexual contact (19.3%).

Trend analyses indicated that during 1999–2017, a significant linear decrease (42.8%–20.7%) occurred in the overall prevalence of watching television 3 or more hours per day. A significant quadratic trend also was identified. The prevalence of watching television 3 or more hours per day decreased during 1999–2013 (42.8%–32.5%) and then decreased more rapidly during 2013–2017 (32.5%–20.7%). The prevalence of watching television 3 or more hours per day decreased from 2015 (24.7%) to 2017 (20.7%).

Analyses of state and large urban school district data indicated that across 35 states, the overall prevalence of watching television 3 or more hours per day ranged from 14.5% to 28.7% across state surveys (median: 20.8%) (Supplementary Table 213). Across 20 large urban school districts, the prevalence ranged from 19.1% to 32.7% (median: 23.6%).

#### Went to Physical Education Classes on 1 or More Days

Nationwide, 51.7% of students went to physical education (PE) classes on 1 or more days in an average week when they were in school (Supplementary Table 214). The prevalence of going to PE classes on 1 or more days was higher among male (55.9%) than female (47.6%) students; higher among white male (52.7%), black male (62.4%), and Hispanic male (58.8%) than white female (45.1%), black female (47.8%), and Hispanic female (53.1%) students, respectively; and higher among 10th-grade male (60.0), 11th-grade male (44.9), and 12th-grade male (42.0) than 10th-grade female (51.0), 11th-grade female (33.4), and 12th-grade female (32.2) students, respectively. The prevalence of going to PE classes on 1 or more days was higher among Hispanic (56.0%) than white (48.7%) students and higher among black male (62.4%) than white male (52.7%) students. The prevalence of going to PE classes on 1 or more days was higher among 9th-grade (72.1%) than 10th-grade (55.4%), 11th-grade (39.0%), and 12th-grade (39.0%) students; higher among 10th-grade (55.4%) than 11th-grade (39.0%) and 12th-grade (36.9%) students; higher among 9th-grade female (70.8%) than 10th-grade female (51.0%), 11th-grade female (33.4%), and 12th-grade female (32.2%) students; higher among 10th-grade female (51.0%) than 11th-grade female (33.4%) and 12th-grade female (32.2%) students; higher among 9th-grade male (73.5%) than 10th-grade male (60.0%), 11th-grade male (44.9%), and 12th-grade male (42.0%) students; and higher among 10th-grade male (60.0%) than 11th-grade male (44.9%) and 12th-grade male (42.0%) students.

Analyses based on the question ascertaining sexual identity indicated that nationwide, 52.0% of heterosexual students; 43.5% of gay, lesbian, and bisexual students; and 51.0% of not sure students went to PE classes on 1 or more days (Supplementary Table 214). The prevalence of going to PE classes on 1 or more days was higher among heterosexual (52.0%) and not sure (51.0%) than gay, lesbian, and bisexual (43.5%) students. Among female students, the prevalence was higher among heterosexual (46.7%) and not sure (51.6%) than lesbian and bisexual (42.0%) students. Among male students, the prevalence was higher among heterosexual (56.7%) than gay and bisexual s (47.6%) students. The prevalence also was higher among heterosexual male (56.7%) than heterosexual female (46.7%) students.

Analyses based on the question ascertaining the sex of sexual contacts indicated that nationwide, 50.1% of students who had sexual contact with only the opposite sex, 39.8% of students who had sexual contact with only the same sex or with both sexes, and 55.1% of students who had no sexual contact went to PE classes on 1 or more days (Supplementary Table 214). The prevalence of going to PE classes on 1 or more days was higher among students who had no sexual contact (55.1%) than students who had sexual contact with only the opposite sex (50.1%) and students who had sexual contact with only the same sex or with both sexes (39.8%) and higher among students who had sexual contact with only the opposite sex (50.1%) than students who had sexual contact with only the same sex or with both sexes (39.8%). Among female students, the prevalence was higher among those who had no sexual contact (52.9%) than those who had sexual contact with only males (42.3%) and those who had sexual contact with only females or with both sexes (36.8%). The prevalence also was higher among male students who had sexual contact with only females (56.5%) than female students who had sexual contact with only males (42.3%), higher among male students who had sexual contact with only males or with both sexes (48.5%) than female students who had sexual contact with only females or with both sexes (36.8%), and higher among male students who had no sexual contact (57.4%) than female students who had no sexual contact (52.9%).

Trend analyses did not identify a significant linear trend in the overall prevalence of going to PE classes on 1 or more days during 1991–2017 (48.9%–51.7%). A significant quadratic trend also was not identified. The prevalence of going to PE classes on 1 or more days did not change significantly from 2015 (51.6%) to 2017 (51.7%).

Analyses of state and large urban school district data indicated that across 35 states, the overall prevalence of going to PE classes on 1 or more days ranged from 27.9% to 91.5% across state surveys (median: 46.4%) (Supplementary Table 215). Across 17 large urban school districts, the prevalence ranged from 28.0% to 86.1% (median: 44.6%).

#### Went to Physical Education Classes on All 5 Days

Nationwide, 29.9% of students went to PE classes on all 5 days in an average week when they were in school (Supplementary Table 216). The prevalence of going to PE classes on all 5 days was higher among male (34.7%) than female (25.3%) students; higher among white male (32.2%), black male (35.8%), and Hispanic male (40.5%) than white female (22.6%), black female (21.6%), and Hispanic female (34.1%) students, respectively; and higher among 9th-grade male (45.5%), 10th-grade male (36.7%), 11th-grade male (28.3%), and 12th-grade male (26.5%) than 9th-grade female (39.2%), 10th-grade female (24.2%), 11th-grade female (20.3%), and 12th-grade female (15.9%) students, respectively. The prevalence of going to PE classes on all 5 days was higher among Hispanic (37.4%) than white (27.2%) students and higher among Hispanic female (34.1%) than white female (22.6%) and black female (21.6%) students. The prevalence of going to PE classes on all 5 days was higher among 9th-grade (42.3%) than 10th-grade (30.2%), 11th-grade (24.3%), and 12th-grade (21.0%) students; higher among 10th-grade (30.2%) than 11th-grade (24.3%) and 12th-grade (21.0%) students; higher among 11th-grade (24.3%) than 12th-grade (21.0%) students; higher among 9th-grade female (39.2%) than 10th-grade female (24.2%), 11th-grade female (20.3%), and 12th-grade female (15.9%) students; higher among 10th-grade female (24.2%) and 11th-grade female (20.3%) than 12th-grade female (15.9%) students; higher among 9th-grade male (45.5%) than 10th-grade male (36.7%), 11th-grade male (28.3%), and 12th-grade male (26.5%) students; and higher among 10th-grade male (36.7%) than 11th-grade male (28.3%) and 12th-grade male (26.5%) students.

Analyses based on the question ascertaining sexual identity indicated that nationwide, 32.3% of heterosexual students; 21.7% of gay, lesbian, and bisexual students; and 24.3% of not sure students went to PE classes on all 5 days (Supplementary Table 216). The prevalence of going to PE classes on all 5 days was higher among heterosexual (32.3%) than gay, lesbian, and bisexual (21.7%) and not sure (24.3%) students. Among female students, the prevalence was higher among heterosexual (28.2%) than lesbian and bisexual (20.4%) students. Among male students, the prevalence was higher among heterosexual (35.9%) than gay and bisexual (25.5%) and not sure (26.9%) students. The prevalence also was higher among heterosexual male (35.9%) than heterosexual female (28.2%) students.

Analyses based on the question ascertaining the sex of sexual contacts indicated that nationwide, 31.3% of students who had sexual contact with only the opposite sex, 19.8% of students who had sexual contact with only the same sex or with both sexes, and 33.8% of students who had no sexual contact went to PE classes on all 5 days (Supplementary Table 216). The prevalence of going to PE classes on all 5 days was higher among students who had sexual contact with only the opposite sex (31.3%) and students who had no sexual contact (33.8%) than students who had sexual contact with only the same sex or with both sexes (19.8%). Among female students, the prevalence was higher among those who had no sexual contact (30.9%) than those who had sexual contact with only males (25.4%) and those who had sexual contact with only females or with both sexes (18.8%) and higher among those who had sexual contact with only males (25.4%) those who had sexual contact with only females or with both sexes (18.8%). Among male students, the prevalence was higher among those who had sexual contact with only females (36.3%) and those who had no sexual contact (36.9%) than those who had sexual contact with only males or with both sexes (22.8%). The prevalence also was higher among male students who had sexual contact with only females (36.3%) than female students who had sexual contact with only males (25.4%) and higher among male students who had no sexual contact (36.9%) than female students who had no sexual contact (30.9%).

Trend analyses did not identify a significant linear trend in the overall prevalence of going to PE classes on all 5 days during 1991–2017 (41.6%–29.9%). A significant quadratic trend also was not identified. The prevalence of going to PE classes on all 5 days did not change significantly from 2015 (29.8%) to 2017 (29.9%).

Analyses of state and large urban school district data indicated that across 35 states, the overall prevalence of going to PE classes on all 5 days ranged from 5.8% to 68.4% across state surveys (median: 22.0%) (Supplementary Table 217). Across 17 large urban school districts, the prevalence ranged from 7.1% to 43.5% (median: 22.1%).

#### Played on at Least One Sports Team

Nationwide, 54.3% of students had played on at least one sports team (counting any teams run by their school or community groups) during the 12 months before the survey (Supplementary Table 218). The prevalence of having played on at least one sports team was higher among male (59.7%) than female (49.3%) students; higher among white male (59.6%), black male (67.5%), and Hispanic male (56.7%) than white female (49.8%), black female (51.1%), and Hispanic female (47.5%) students, respectively; and higher among 9th-grade male (63.9%), 10th-grade male (59.2%), 11th-grade male (59.5%), and 12th-grade male (55.9%) than 9th-grade female (56.4%), 10th-grade female (49.2%), 11th-grade female (47.0%), and 12th-grade female (43.8%) students, respectively. The prevalence of having played on at least one sports team was higher among black (59.1%) than Hispanic (52.2%) students and higher among black male (67.5%) than white male (59.6%) and Hispanic male (56.7%) students. The prevalence of having played on at least one sports team was higher among 9th-grade (60.0%) than 10th-grade (54.0%), 11th-grade (53.1%), and 12th-grade (49.6%) students; higher among 10th-grade (54.0%) and 11th-grade (53.1%) than 12th-grade (49.6%) students; higher among 9th-grade female (56.4%) than 10th-grade female (49.2%), 11th-grade female (47.0%), and 12th-grade female (43.8%) students; higher among 10th-grade female (49.2%) than 12th-grade female (43.8%) students; and higher among 9th-grade male (63.9%) than 10th-grade male (59.2%) and 12th-grade male (55.9%) students.

Analyses based on the question ascertaining sexual identity indicated that nationwide, 57.9% of heterosexual students; 38.5% of gay, lesbian, and bisexual students; and 43.7% of not sure students had played on at least one sports team (Supplementary Table 218). The prevalence of having played on at least one sports team was higher among heterosexual (57.9%) than gay, lesbian, and bisexual (38.5%) and not sure (43.7%) students. Among female students, the prevalence was higher among heterosexual (54.1%) than lesbian and bisexual (38.1%) and not sure (44.9%) students. Among male students, the prevalence was higher among heterosexual (61.2%) than gay and bisexual (40.0%) and not sure (42.6%) students. The prevalence also was higher among heterosexual male (61.2%) than heterosexual female (54.1%) students.

Analyses based on the question ascertaining the sex of sexual contacts indicated that nationwide, 60.0% of students who had sexual contact with only the opposite sex, 41.8% of students who had sexual contact with only the same sex or with both sexes, and 53.4% of students who had no sexual contact had played on at least one sports team (Supplementary Table 218). The prevalence of having played on at least one sports team was higher among students who had sexual contact with only the opposite sex (60.0%) and students who had no sexual contact (53.4%) than students who had sexual contact with only the same sex or with both sexes (41.3%) and higher among students who had sexual contact with only the opposite sex (60.0%) than students who had no sexual contact (53.4%). Among female students, the prevalence was higher among those who had sexual contact with only males (52.9%) and those who had no sexual contact (52.2%) than those who had sexual contact with only females or with both sexes (41.3%). Among male students, the prevalence was higher among those who had sexual contact with only females (66.0%) than those who had sexual contact with only males or with both sexes (43.4%) and those who had no sexual contact (54.8%). The prevalence also was higher among male students who had sexual contact with only females (66.0%) than female students who had sexual contact with only males (52.9%).

Trend analyses did not identify a significant linear trend in the overall prevalence of having played on at least one sports team during 1999–2017 (55.1%–54.3%). A significant quadratic trend also was not identified. The prevalence of having played on at least one sports team did not change significantly from 2015 (57.6%) to 2017 (54.3%).

Analyses of state and large urban school district data indicated that across 26 states, the overall prevalence of having played on at least one sports team ranged from 46.8% to 62.8% across state surveys (median: 54.6%) (Supplementary Table 219). Across 15 large urban school districts, the prevalence ranged from 40.4% to 54.7% (median: 47.7%).

#### Had a Concussion One or More Times from Playing a Sport or Being Physically Active

Nationwide, 15.1% of students had a concussion one or more times during the 12 months before the survey from playing a sport or being physically active (Supplementary Table 220). The prevalence of having had a concussion one or more times was higher among male (17.1%) than female (13.0%) students; higher among white male (16.7%), black male (20.0%), and Hispanic male (16.5%) than white female (12.6%), black female (13.9%), and Hispanic female (13.5%) students, respectively; and higher among 10th-grade male (18.6%) and 12th-grade male (13.9%) than 10th-grade female (11.9%) and 12th-grade female (10.5%) students, respectively. The prevalence of having had a concussion one or more times was higher among black male (20.0%) than Hispanic male (16.5%) students. The prevalence of having had a concussion one or more times was higher among 9th-grade (17.0%), 10th-grade (15.2%), and 11th-grade (15.3%) than 12th-grade (12.2%) students; higher among 9th-grade female (15.5%) than 10th-grade female (11.9%) and 12th-grade female (10.5%) students; and higher among 9th-grade male (18.6%), 10th-grade male (18.6%), and 11th-grade male (17.1%) than 12th-grade male (13.9%) students.

Analyses based on the question ascertaining sexual identity indicated that nationwide, 15.0% of heterosexual students; 15.7% of gay, lesbian, and bisexual students; and 17.2% of not sure students had had a concussion one or more times (Supplementary Table 220). Among female students, the prevalence of having had a concussion one or more times was higher among lesbian and bisexual (15.7%) than heterosexual (12.8%) students. The prevalence also was higher among heterosexual male (16.9%) than heterosexual female (12.8%) students.

Analyses based on the question ascertaining the sex of sexual contacts indicated that nationwide, 18.3% of students who had sexual contact with only the opposite sex, 18.9% of students who had sexual contact with only the same sex or with both sexes, and 11.2% of students who had no sexual contact had had a concussion one or more times (Supplementary Table 220). The prevalence of having had a concussion one or more times was higher among students who had sexual contact with only the opposite sex (18.3%) and students who had sexual contact with only the same sex or with both sexes (18.9%) than students who had no sexual contact (11.2%). Among female students, the prevalence was higher among those who had sexual contact with only males (14.5%) and those who had sexual contact with only females or with both sexes (18.2%) than those who had no sexual contact (11.1%) and higher among those who had sexual contact with only females or with both sexes (18.2%) than those who had sexual contact with only males (14.5%). Among male students, the prevalence was higher among those who had sexual contact with only females (21.5%) and those who had sexual contact with only males or with both sexes (20.8%) than those who had no sexual contact (11.3%). The prevalence also was higher among male students who had sexual contact with only females (21.5%) than female students who had sexual contact with only males (14.5%).

The question measuring the prevalence of having had a concussion one or more times was used for the first time in the 2017 national YRBS. As a result, long-term temporal trends and 2-year temporal changes are not available for this variable.

Analyses of state and large urban school district data indicated that across 28 states, the overall prevalence of having had a concussion one or more times ranged from 12.7% to 21.5% across state surveys (median: 15.8%) (Supplementary Table 221). Across 15 large urban school districts, the prevalence ranged from 10.7% to 20.9% (median: 16.2%).

### Obesity, Overweight, and Weight Control

#### Obesity

Nationwide, 14.8% of students had obesity (were ≥95th percentile for body mass index, based on sex- and age-specific reference data from the 2000 CDC growth charts) (Supplementary Table 222). The prevalence of obesity was higher among male (17.5%) than female (12.1%) students; higher among white male (14.8%) and Hispanic male (22.2%) than white female (10.3%) and Hispanic female (14.0%) students, respectively; and higher among 9th-grade male (15.9%), 10th-grade male (18.9%), 11th-grade male (18.6%), and 12th-grade male (16.2%) than 9th-grade female (10.3%), 10th-grade female (11.0%), 11th-grade female (15.2%), and 12th-grade female (12.4%) students, respectively. The prevalence of obesity was higher among black (18.2%) and Hispanic (18.2%) than white (12.5%) students, higher among black female (16.7%) and Hispanic female (14.0%) than white female (10.3%) students, and higher among black male (19.7%) and Hispanic male (22.2%) than white male (14.8%) students. The prevalence of obesity was higher among 11th-grade (16.9%) than 9th-grade (13.1%) and 12th-grade (14.2%) students and higher among 11th-grade female (15.2%) than 9th-grade female (10.3%) and 10th-grade female (11.0%) students.

Analyses based on the question ascertaining sexual identity indicated that nationwide, the prevalence of obesity was 14.4% among heterosexual students; 20.5% among gay, lesbian, and bisexual students; and 16.5% among not sure students (Supplementary Table 222). The prevalence of obesity was higher among gay, lesbian, and bisexual (20.5%) than heterosexual (14.4%) students. Among female students, the prevalence was higher among lesbian and bisexual (20.0%) and not sure (17.6%) than heterosexual (10.8%) students. The prevalence also was higher among heterosexual male (17.5%) than heterosexual female (10.8%) students.

Analyses based on the question ascertaining the sex of sexual contacts indicated that nationwide, the prevalence of obesity was 13.5% among students who had sexual contact with only the opposite sex, 21.2% among students who had sexual contact with only the same sex or with both sexes, and 15.6% among students who had no sexual contact (Supplementary Table 222). The prevalence of obesity was higher among students who had sexual contact with only the same sex or with both sexes (21.2%) and students who had no sexual contact (15.6%) than students who had sexual contact with only the opposite sex (13.5%) and higher among students who had sexual contact with only the same sex or with both sexes (21.2%) than students who had no sexual contact (15.6%). Among female students, the prevalence was higher among those who had sexual contact with only females or with both sexes (20.7%) and those who had no sexual contact (12.8%) than those who had sexual contact with only males (10.0%) and higher among those who had sexual contact with only females or with both sexes (20.7%) than those who had no sexual contact (12.8%). The prevalence also was higher among male students who had sexual contact with only females (16.5%) than female students who had sexual contact with only males (10.0%) and higher among male students who had no sexual contact (18.6%) than female students who had no sexual contact (12.8%).

Trend analyses indicated that during 1999–2017, a significant linear increase (10.6%–14.8%) occurred in the overall prevalence of obesity. A significant quadratic trend was not identified. The prevalence of obesity did not change significantly from 2015 (13.9%) to 2017 (14.8%).

Analyses of state and large urban school district data indicated that across 39 states, the overall prevalence of obesity ranged from 9.5% to 21.7% across state surveys (median: 14.2%) (Supplementary Table 223). Across 21 large urban school districts, the prevalence ranged from 10.1% to 20.4% (median: 16.1%).

#### Overweight

Nationwide, 15.6% of students were overweight (≥85th percentile but <95th percentile for body mass index, based on sex- and age-specific reference data from the 2000 CDC growth charts) (Supplementary Table 224). The prevalence of overweight was higher among female (16.8%) than male (14.4%) students, higher among black female (20.8%) than black male (14.8%) students, and higher among 11th-grade female (18.8%) than 11th-grade male (14.1%) students. The prevalence of overweight was higher among black (17.8%) and Hispanic (19.5%) than white (14.0%) students, higher among black female (20.8%) and Hispanic female (21.9%) than white female (14.3%) students, and higher among Hispanic male (17.1%) than white male (13.6%) students.

Analyses based on the question ascertaining sexual identity indicated that nationwide, the prevalence of overweight was 15.5% among heterosexual students; 19.2% among gay, lesbian, and bisexual students; and 15.7% among not sure students (Supplementary Table 224). The prevalence of overweight was higher among gay, lesbian, and bisexual (19.2%) than heterosexual (15.5%) students. Among female students, the prevalence was higher among lesbian and bisexual (20.5%) than heterosexual (16.6%) students. The prevalence also was higher among heterosexual female (16.6%) than heterosexual male (14.5%) students.

Analyses based on the question ascertaining the sex of sexual contacts indicated that nationwide, the prevalence of overweight was 16.2% among students who had sexual contact with only the opposite sex, 18.3% among students who had sexual contact with only the same sex or with both sexes, and 15.2% among students who had no sexual contact (Supplementary Table 224). The prevalence of overweight was higher among female students who had no sexual contact (16.8%) than male students who had no sexual contact (13.6%).

Trend analyses indicated that during 1999–2017, a significant linear increase occurred in the overall prevalence of overweight (14.1%–15.6%). A significant quadratic trend was not identified. The prevalence of overweight did not change significantly from 2015 (16.0%) to 2017 (15.6%).

Analyses of state and large urban school district data indicated that across 39 states, the overall prevalence of overweight ranged from 12.3% to 18.3% across state surveys (median: 15.9%) (Supplementary Table 225). Across 21 large urban school districts, the prevalence ranged from 12.2% to 20.4% (median: 16.6%).

#### Described Themselves as Overweight

Nationwide, 31.5% of students described themselves as slightly or very overweight (Supplementary Table 226). The prevalence of describing themselves as overweight was higher among female (37.5%) than male (25.3%) students; higher among white female (35.4%), black female (36.8%), and Hispanic female (42.5%) than white male (23.9%), black male (19.1%), and Hispanic male (31.9%) students, respectively; and higher among 9th-grade female (35.2%), 10th-grade female (34.6%), 11th-grade female (41.8%), and 12th-grade female (38.6%) than 9th-grade male (25.6%), 10th-grade male (24.6%), 11th-grade male (25.3%), and 12th-grade male (25.5%) students, respectively. The prevalence of describing themselves as overweight was higher among Hispanic (37.1%) than white (29.9%) and black (28.1%) students, higher among Hispanic female (42.5%) than white female (35.4%) and black female (36.8%) students, higher among white male (23.9%) and Hispanic male (31.9%) than black male (19.1%) students, and higher among Hispanic male (31.9%) than white male (23.9%) students. The prevalence of describing themselves as overweight was higher among 11th-grade (33.8%) than 9th-grade (30.5%) and 10th-grade (29.7%) students and higher among 11th-grade female (41.8%) than 9th-grade female (35.2%) and 10th-grade female (34.6%) students.

Analyses based on the question ascertaining sexual identity indicated that nationwide, 29.9% of heterosexual students; 45.6% of gay, lesbian, and bisexual students; and 43.0% of not sure students described themselves as overweight (Supplementary Table 226). The prevalence of describing themselves as overweight was higher among gay, lesbian, and bisexual (45.6%) and not sure (43.0%) than heterosexual (29.9%) students. Among female students, the prevalence was higher among lesbian and bisexual (48.4%) and not sure (48.7%) than heterosexual (36.0%) students. Among male students, the prevalence was higher among gay and bisexual (37.3%) than heterosexual (24.6%) students. The prevalence also was higher among heterosexual female (36.0%) than heterosexual male (24.6%) students, higher among lesbian and bisexual female (48.4%) than gay and bisexual male (37.3%) students, and higher among not sure female (48.7%) than not sure male (33.6%) students.

Analyses based on the question ascertaining the sex of sexual contacts indicated that nationwide, 28.6% of students who had sexual contact with only the opposite sex, 46.4% of students who had sexual contact with only the same sex or with both sexes, and 34.3% of students who had no sexual contact described themselves as overweight (Supplementary Table 226). The prevalence of describing themselves as overweight was higher among students who had sexual contact with only the same sex or with both sexes (46.4%) and students who had no sexual contact (34.3%) than students who had sexual contact with only the opposite sex (28.6%) and higher among students who had sexual contact with only the same sex or with both sexes (46.4%) than students who had no sexual contact (34.3%). Among female students, the prevalence was higher among those who had sexual contact with only females or with both sexes (49.8%) and those who had no sexual contact (39.9%) than those who had sexual contact with only males (35.6%) and higher among those who had sexual contact with only females or with both sexes (49.8%) than those who had no sexual contact (39.9%). Among male students, the prevalence was higher among those who had sexual contact with only males or with both sexes (36.5%) and those who had no sexual contact (28.4%) than those who had sexual contact with only females (22.8%). The prevalence also was higher among female students who had sexual contact with only males (35.6%) than male students who had sexual contact with only females (22.8%), higher among female students who had sexual contact with only females or with both sexes (49.8%) than male students who had sexual contact with only males or with both sexes (36.5%), and higher among female students who had no sexual contact (39.9%) than male students who had no sexual contact (28.4%).

Trend analyses did not identify a significant linear trend in the overall prevalence of describing themselves as overweight during 1991–2017 (31.8%–31.5%). A significant quadratic trend was identified. The prevalence of describing themselves as overweight decreased during 1991–1995 (31.8%–27.6%) and then increased during 1995–2017 (27.6%–31.5%). The prevalence of describing themselves as overweight did not change significantly from 2015 (31.5%) to 2017 (31.5%).

Analyses of state and large urban school district data indicated that across 30 states, the overall prevalence of describing themselves as overweight ranged from 25.5% to 35.9% across state surveys (median: 30.7%) (Supplementary Table 227). Across 19 large urban school districts, the prevalence ranged from 22.4% to 37.3% (median: 29.2%).

#### Trying to Lose Weight

Nationwide, 47.1% of students were trying to lose weight (Supplementary Table 228). The prevalence of trying to lose weight was higher among female (59.9%) than male (34.0%) students; higher among white female (58.6%), black female (55.3%), and Hispanic female (65.6%) than white male (30.6%), black male (28.9%), and Hispanic male (45.7%) students, respectively; and higher among 9th-grade female (56.9%), 10th-grade female (57.9%), 11th-grade female (63.4%), and 12th-grade female (62.0%) than 9th-grade male (35.4%), 10th-grade male (34.3%), 11th-grade male (33.0%), and 12th-grade male (32.9%) students, respectively. The prevalence of trying to lose weight was higher among Hispanic (55.4%) than white (45.1%) and black (42.3%) students, higher among Hispanic female (65.6%) than white female (58.6%) and black female (55.3%) students, and higher among Hispanic male (45.7%) than white male (30.6%) and black male (28.9%) students. The prevalence of trying to lose weight was higher among 11th-grade female (63.4%) than 9th-grade female (56.9%) and 10th-grade female (57.9%) students.

Analyses based on the question ascertaining sexual identity indicated that nationwide, 45.8% of heterosexual students; 59.5% of gay, lesbian, and bisexual students; and 49.3% of not sure students were trying to lose weight (Supplementary Table 228). The prevalence of trying to lose weight was higher among gay, lesbian, and bisexual (59.5%) than heterosexual (45.8%) and not sure (49.3%) students. Among male students, the prevalence was higher among gay and bisexual (48.5%) than heterosexual (33.7%) students. The prevalence also was higher among heterosexual female (60.0%) than heterosexual male (33.7%) students, higher among lesbian and bisexual female (63.1%) than gay and bisexual male (48.5%) students, and higher among not sure female (61.5%) than not sure male (32.1%) students.

Analyses based on the question ascertaining the sex of sexual contacts indicated that nationwide, 46.0% of students who had sexual contact with only the opposite sex, 58.5% of students who had sexual contact with only the same sex or with both sexes, and 47.2% of students who had no sexual contact were trying to lose weight (Supplementary Table 228). The prevalence of trying to lose weight was higher among students who had sexual contact with only the same sex or with both sexes (58.5%) than students who had sexual contact with only the opposite sex (46.0%) and students who had no sexual contact (47.2%). Among male students, the prevalence was higher among those who had sexual contact with only males or with both sexes (44.0%) than those who had sexual contact with only females (32.6%). The prevalence also was higher among female students who had sexual contact with only males (62.2%) than male students who had sexual contact with only females (32.6%), higher among female students who had sexual contact with only females or with both sexes (63.5%) than male students who had sexual contact with only males or with both sexes (44.0%), and higher among female students who had no sexual contact (58.7%) than male students who had no sexual contact (35.0%).

Trend analyses indicated that during 1991–2017, a significant linear increase (41.8%–47.1%) occurred in the overall prevalence of trying to lose weight. A significant quadratic trend was not identified. The prevalence of trying to lose weight did not change significantly from 2015 (45.6%) to 2017 (47.1%).

Analyses of state and large urban school district data indicated that across 29 states, the overall prevalence of trying to lose weight ranged from 41.1% to 52.3% across state surveys (median: 44.8%) (Supplementary Table 229). Across 18 large urban school districts, the prevalence ranged from 41.0% to 50.6% (median: 44.5%).

### Other Health-Related Topics

#### Asthma

Nationwide, 22.5% of students had ever been told by a doctor or nurse that they have asthma (Supplementary Table 230). The prevalence of having ever been told they have asthma was higher among black (29.8%) than white (20.9%) and Hispanic (21.1%) students, higher among black female (29.1%) than white female (21.2%) and Hispanic female (20.7%) students, and higher among black male (30.5%) than white male (20.6%) and Hispanic male (21.6%) students.

Analyses based on the question ascertaining sexual identity indicated that nationwide, 22.1% of heterosexual students; 29.1% of gay, lesbian, and bisexual students; and 23.3% of not sure students had ever been told they have asthma (Supplementary Table 230). The prevalence of having ever been told they have asthma was higher among gay, lesbian, and bisexual (29.1%) than heterosexual (22.1%) students. Among female students, the prevalence was higher among lesbian and bisexual (27.6%) than heterosexual (22.4%) students. Among male students, the prevalence was higher among gay and bisexual (32.3%) than heterosexual (22.0%) and not sure (19.8%) students.

Analyses based on the question ascertaining the sex of sexual contacts indicated that nationwide, 23.2% of students who had sexual contact with only the opposite sex, 27.6% of students who had sexual contact with only the same sex or with both sexes, and 21.1% of students who had no sexual contact had ever been told they have asthma (Supplementary Table 230). The prevalence of having ever been told they have asthma was higher among students who had sexual contact with only the same sex or with both sexes (27.6%) than students who had no sexual contact (21.1%). Among female students, the prevalence was higher among those who had sexual contact with only females or with both sexes (28.1%) than those who had no sexual contact (21.8%).

Trend analyses indicated that during 2003–2017, a significant linear increase (18.9%–22.5%) occurred in the overall prevalence of having ever been told they have asthma. A significant quadratic trend also was identified. The prevalence of having ever been told they have asthma increased during 2003–2009 (18.9%–22.0%) and then did not change significantly during 2009–2017 (22.0%–22.5%). The prevalence of having ever been told they have asthma did not change significantly from 2015 (22.8%) to 2017 (22.5%).

Analyses of state and large urban school district data indicated that across 29 states, the overall prevalence of having ever been told they have asthma ranged from 19.3% to 33.4% across state surveys (median: 24.3%) (Supplementary Table 231). Across 20 large urban school districts, the prevalence ranged from 17.4% to 33.4% (median: 23.9%).

#### Never Saw a Dentist

Nationwide, 1.5% of students had never seen a dentist for a check-up, exam, teeth cleaning, or other dental work (Supplementary Table 232). The prevalence of having never seen a dentist was higher among male (1.7%) than female (1.2%) students, higher among Hispanic male (2.5%) than Hispanic female (1.2%) students, and higher among 12th-grade male (2.2%) than 12th-grade female (0.7%) students. The prevalence of having never seen a dentist was higher among black (2.3%) and Hispanic (1.9%) than white (1.0%) students and higher among black male (2.7%) and Hispanic male (2.5%) than white male (1.2%) students. The prevalence of having never seen a dentist was higher among 9th-grade male (2.0%) and 12th-grade male (2.2%) than 10th-grade male (1.1%) students.

Analyses based on the question ascertaining sexual identity indicated that nationwide, 1.4% of heterosexual students; 1.5% of gay, lesbian, and bisexual students; and 2.6% of not sure students had never seen a dentist (Supplementary Table 232). Analyses based on the question ascertaining the sex of sexual contacts indicated that nationwide, 1.2% of students who had sexual contact with only the opposite sex, 2.8% of students who had sexual contact with only the same sex or with both sexes, and 1.2% of students who had no sexual contact had never seen a dentist (Supplementary Table 232). The prevalence of having never seen a dentist was higher among students who had sexual contact with only the same sex or with both sexes (2.8%) than students who had sexual contact with only the opposite sex (1.2%) and students who had no sexual contact (1.2%). The prevalence also was higher among male students who had sexual contact with only females (1.6%) than female students who had sexual contact with only males (0.7%).

Trend analyses indicated that during 1999–2017, a significant linear decrease (3.0%–1.5%) occurred in the overall prevalence of having never seen a dentist. Not enough data points were available to identify a quadratic trend, because the question measuring the prevalence of having never seen a dentist was only used in 1999, 2001, 2003, 2015, and 2017. The prevalence of having never seen a dentist did not change significantly from 2015 (1.9%) to 2017 (1.5%).

Analyses of state and large urban school district data indicated that across 31 states, the overall prevalence of having never seen a dentist ranged from 0.9% to 4.7% across state surveys (median: 1.9%) (Supplementary Table 233). Across 19 large urban school districts, the prevalence ranged from 1.6% to 4.3% (median: 2.7%).

#### Saw a Dentist

Nationwide, 75.7% of students had seen a dentist for a check-up, exam, teeth cleaning, or other dental work during the 12 months before the survey (Supplementary Table 234). The prevalence of having seen a dentist during the 12 months before the survey was higher among female (77.3%) than male (74.2%) students; higher among white female (82.7%) and Hispanic female (74.1%) than white male (79.0%) and Hispanic male (69.3%) students, respectively; and higher among 10th-grade female (79.1%) than 10th-grade male (75.3%) students. The prevalence of having seen a dentist during the 12 months before the survey was higher among white (80.8%) and Hispanic (71.6%) than black (64.5%) students, higher among white (80.8%) than Hispanic (71.6%) students, higher among white female (82.7%) and Hispanic female (74.1%) than black female (66.1%) students, higher among white female (82.7%) than Hispanic female (74.1%) students, higher among white male (79.0%) and Hispanic male (69.3%) than black male (62.9%) students, and higher among white male (79.0%) than Hispanic male (69.3%) students. The prevalence of having seen a dentist during the 12 months before the survey was higher among 10th-grade (77.1%) than 12th-grade (73.8%) students and higher among 10th-grade male (75.3%) than 12th-grade male (71.8%) students.

Analyses based on the question ascertaining sexual identity indicated that nationwide, 76.2% of heterosexual students; 70.0% of gay, lesbian, and bisexual students; and 67.6% of not sure students had seen a dentist during the 12 months before the survey (Supplementary Table 234). The prevalence of having seen a dentist during the 12 months before the survey was higher among heterosexual (76.2%) than gay, lesbian, and bisexual (70.0%) and not sure (67.6%) students. Among male students, the prevalence was higher among heterosexual (75.8%) than gay and bisexual (60.3%) and not sure (51.6%) students. The prevalence also was higher among lesbian and bisexual female (73.6%) than gay and bisexual male (60.3%) students and higher among not sure female (79.3%) than not sure male (51.6%) students.

Analyses based on the question ascertaining the sex of sexual contacts indicated that nationwide, 75.8% of students who had sexual contact with only the opposite sex, 68.8% of students who had sexual contact with only the same sex or with both sexes, and 76.5% of students who had no sexual contact had seen a dentist during the 12 months before the survey (Supplementary Table 234). The prevalence of having seen a dentist during the 12 months before the survey was higher among students who had sexual contact with only the opposite sex (75.8%) and students who had no sexual contact (76.5%) than students who had sexual contact with only the same sex or with both sexes (68.8%). Among male students, the prevalence was higher among those who had sexual contact with only females (74.1%) and those who had no sexual contact (76.3%) than those who had sexual contact with only males or with both sexes (57.3%). The prevalence also was higher among female students who had sexual contact with only males (77.8%) than male students who had sexual contact with only females (74.1%) and higher among female students who had sexual contact with only females or with both sexes (72.8%) than male students who had sexual contact with only males or with both sexes (57.3%).

Trend analyses indicated that during 1999–2017, a significant linear increase (66.8%–75.7%) occurred in the overall prevalence of having seen a dentist during the 12 months before the survey. Not enough data points were available to identify a quadratic trend, because the question measuring the prevalence of having seen a dentist during the 12 months before the survey was only used in 1999, 2001, 2003, 2015, and 2017. The prevalence of having seen a dentist during the 12 months before the survey did not change significantly from 2015 (74.4%) to 2017 (75.7%).

Analyses of state and large urban school district data indicated that across 31 states, the overall prevalence of having seen a dentist during the 12 months before the survey ranged from 65.0% to 82.8% across state surveys (median: 76.1%) (Supplementary Table 235). Across 19 large urban school districts, the prevalence ranged from 60.9% to 74.2% (median: 68.1%).

#### Got 8 or More Hours of Sleep

Nationwide, 25.4% of students got 8 or more hours of sleep on an average school night (Supplementary Table 236). The prevalence of getting 8 or more hours of sleep was higher among 9th-grade male (37.5%) than 9th-grade female (32.3%) students. The prevalence of getting 8 or more hours of sleep was higher among 9th-grade (34.8%) than 10th-grade (26.6%), 11th-grade (21.4%), and 12th-grade (17.6%) students; higher among 10th-grade (26.6%) than 11th-grade (21.4%) and 12th-grade (17.6%) students; higher among 11th-grade (21.4%) than 12th-grade (17.6%) students; higher among 9th-grade female (32.3%) than 10th-grade female (26.0%), 11th-grade female (21.1%), and 12th-grade female (17.9%) students; higher among 10th-grade female (26.0%) than 11th-grade female (21.1%) and 12th-grade female (17.9%) students; higher among 9th-grade male (37.5%) than 10th-grade male (27.0%), 11th-grade male (21.6%), and 12th-grade male (17.3%) students; and higher among 10th-grade male (27.0%) than 11th-grade male (21.6%) and 12th-grade male (17.3%) students.

Analyses based on the question ascertaining sexual identity indicated that nationwide, 25.9% of heterosexual students; 17.8% of gay, lesbian, and bisexual students; and 24.7% of not sure students had gotten 8 or more hours of sleep (Supplementary Table 236). The prevalence of getting 8 or more hours of sleep was higher among heterosexual (25.9%) and not sure (24.7%) than gay, lesbian, and bisexual (17.8%) students. Among female students, the prevalence was higher among heterosexual (25.6%) than lesbian and bisexual (18.1%) students. Among male students, the prevalence was higher among heterosexual (26.4%) and not sure (32.1%) than gay and bisexual (18.0%) students. The prevalence also was higher among not sure male (32.1%) than not sure female (20.5%) students.

Analyses based on the question ascertaining the sex of sexual contacts indicated that nationwide, 21.9% of students who had sexual contact with only the opposite sex, 15.8% of students who had sexual contact with only the same sex or with both sexes, and 29.8% of students who had no sexual contact had gotten 8 or more hours of sleep (Supplementary Table 236). The prevalence of getting 8 or more hours of sleep was higher among students who had sexual contact with only the opposite sex (21.9%) and students who had no sexual contact (29.8%) than students who had sexual contact with only the same sex or with both sexes (15.8%) and higher among students who had no sexual contact (29.8%) than students who had sexual contact with only the opposite sex (21.9%). Among female students, the prevalence was higher among those who had sexual contact with only males (21.2%) and those who had no sexual contact (28.4%) than those who had sexual contact with only females or with both sexes (16.6%) and higher among those who had no sexual contact (28.4%) than those who had sexual contact with only males (21.2%). Among male students, the prevalence was higher among those who had sexual contact with only females (22.5%) and those who had no sexual contact (31.3%) than those who had sexual contact with only males or with both sexes (13.5%) and higher among those who had no sexual contact (31.3%) than those who had sexual contact with only females (22.5%).

Trend analyses indicated that during 2007–2017, a significant linear decrease (31.1%–25.4%) occurred in the overall prevalence of getting 8 or more hours of sleep. A significant quadratic trend also was identified. The prevalence of getting 8 or more hours of sleep did not change significantly during 2007–2013 (31.1%–31.7%) and then decreased during 2013–2017 (31.7%–25.4%). The prevalence of getting 8 or more hours of sleep did not change significantly from 2015 (27.3%) to 2017 (25.4%).

Analyses of state and large urban school district data indicated that across 34 states, the overall prevalence of getting 8 or more hours of sleep ranged from 19.4% to 32.8% across state surveys (median: 23.7%) (Supplementary Table 237). Across 21 large urban school districts, the prevalence ranged from 12.1% to 30.5% (median: 20.2%).

#### Indoor Tanning Device Use

Nationwide, 5.6% of students had used an indoor tanning device (e.g., a sunlamp, sunbed, or tanning booth, not counting getting a spray-on tan) one or more times during the 12 months before the survey (i.e., indoor tanning device use) (Supplementary Table 238). The prevalence of indoor tanning device use was higher among female (7.5%) than male (3.5%) students; higher among white female (10.1%) than white male (2.8%) students; higher among black male (7.0%) than black female (3.8%) students; and higher among 9th-grade female (5.0%), 11th-grade female (8.1%), and 12th-grade female (12.9%) than 9th-grade male (2.3%), 11th-grade male (2.9%), and 12th-grade male (4.5%) students, respectively. The prevalence of indoor tanning device use was higher among white (6.6%) and black (5.5%) than Hispanic (3.2%) students, higher among white female (10.1%) than black female (3.8%) and Hispanic female (3.0%) students, and higher among black male (7.0%) than white male (2.8%) and Hispanic male (3.4%) students. The prevalence of indoor tanning device use was higher among 12th-grade (8.9%) than 9th-grade (3.7%), 10th-grade (4.3%), and 11th-grade (5.5%) students; higher among 11th-grade (5.5%) than 9th-grade (3.7%) students; higher among 11th-grade female (8.1%) and 12th-grade female (12.9%) than 9th-grade female (5.0%) and 10th-grade female (4.2%) students; higher among 12th-grade female (12.9%) than 11th-grade female (8.1%) students; higher among 10th-grade male (4.3%) and 12th-grade male (4.5%) than 9th-grade male (2.3%) students; and higher among 12th-grade male (4.5%) than 11th-grade male (2.9%) students.

Analyses based on the question ascertaining sexual identity indicated that nationwide, the prevalence of indoor tanning device use was 5.4% among heterosexual students; 6.0% among gay, lesbian, and bisexual students; and 9.9% among not sure students (Supplementary Table 238). The prevalence of indoor tanning device use was higher among not sure (9.9%) than heterosexual (5.4%) students. Among female students, the prevalence was higher among heterosexual (8.4%) than lesbian and bisexual (4.9%) and not sure (5.0%) students. Among male students, the prevalence was higher among gay and bisexual (9.4%) and not sure (15.6%) than heterosexual (2.8%) students. The prevalence also was higher among heterosexual female (8.4%) than heterosexual male (2.8%) students and higher among not sure male (15.6%) than not sure female (5.0%).

Analyses based on the question ascertaining the sex of sexual contacts indicated that nationwide, the prevalence of indoor tanning device use was 7.7% among students who had sexual contact with only the opposite sex, 10.8% among students who had sexual contact with only the same sex or with both sexes, and 2.3% among students who had no sexual contact (Supplementary Table 238). The prevalence of indoor tanning device use was higher among students who had sexual contact with only the opposite sex (7.7%) and students who had sexual contact with only the same sex or with both sexes (10.8%) than students who had no sexual contact (2.3%). Among female students, the prevalence was higher among those who had sexual contact with only males (12.1%) and those who had sexual contact with only females or with both sexes (9.2%) than those who had no sexual contact (3.4%). Among male students, the prevalence was higher among those who had sexual contact with only females (4.1%) and those who had sexual contact with only males or with both sexes (15.6%) than those who had no sexual contact (1.1%) and higher among those who had sexual contact with only males or with both sexes (15.6%) than those who had sexual contact with only females (4.1%). The prevalence also was higher among female students who had sexual contact with only males (12.1%) than male students who had sexual contact with only females (4.1%) and higher among female students who had no sexual contact (3.4%) than male students who had no sexual contact (1.1%).

Trend analyses indicated that during 2009–2017, a significant linear decrease (15.6%–5.6%) occurred in the overall prevalence of indoor tanning device use. Not enough data points were available to identify a quadratic trend. The prevalence of indoor tanning device use decreased from 2015 (7.3%) to 2017 (5.6%).

The question measuring the prevalence of indoor tanning device use was not included in the standard questionnaire used in the state and large urban school district surveys in 2017. As a result, the range and median prevalence estimates across states and large urban school districts for the prevalence of indoor tanning device use are not available.

#### Had a Sunburn

Nationwide, 57.2% of students had had a sunburn one or more times (counting the number of times even a small part of their skin turned red or hurt for 12 or more hours after being outside in the sun or after using a sunlamp or other indoor tanning device) during the 12 months before the survey (Supplementary Table 239). The prevalence of having had a sunburn was higher among female (61.6%) than male (52.8%) students; higher among white female (78.8%), black female (15.5%), and Hispanic female (50.1%) than white male (70.5%), black male (10.4%), and Hispanic male (40.3%) students, respectively; and higher among 9th-grade female (61.5%), 10th-grade female (61.2%), 11th-grade female (59.9%), and 12th-grade female (63.9%) than 9th-grade male (53.6%), 10th-grade male (52.9%), 11th-grade male (51.2%), and 12th-grade male (53.2%) students, respectively. The prevalence of having had a sunburn was higher among white (74.8%) and Hispanic (45.0%) than black (13.0%) students, higher among white (74.8%) than Hispanic (45.0%) students, higher among white female (78.8%) and Hispanic female (50.1%) than black female (15.5%) students, higher among white female (78.8%) than Hispanic female (50.1%) students, higher among white male (70.5%) and Hispanic male (40.3%) than black male (10.4%) students, and higher among white male (70.5%) than Hispanic male (40.3%) students.

Analyses based on the question ascertaining sexual identity indicated that nationwide, 57.0% of heterosexual students; 56.2% of gay, lesbian, and bisexual students; and 52.4% of not sure students had had a sunburn (Supplementary Table 239). Among female students, the prevalence of having had a sunburn was higher among heterosexual (62.7%) than lesbian and bisexual (54.6%) students. Among male students, the prevalence was higher among gay and bisexual (62.3%) than heterosexual (52.2%) and not sure (45.7%) students. The prevalence also was higher among heterosexual female (62.7%) than heterosexual male (52.2%) students and higher among gay and bisexual male (62.3%) than lesbian and bisexual female (54.6%) students.

Analyses based on the question ascertaining the sex of sexual contacts indicated that nationwide, 58.9% of students who had sexual contact with only the opposite sex, 57.7% of students who had sexual contact with only the same sex or with both sexes, and 55.7% of students who had no sexual contact had had a sunburn (Supplementary Table 239). The prevalence of having had a sunburn was higher among students who had sexual contact with only the opposite sex (58.9%) than students who had no sexual contact (55.7%). Among female students, the prevalence was higher among those who had sexual contact with only males (65.6%) than those who had sexual contact with only females or with both sexes (57.0%) and those who had no sexual contact (58.9%). The prevalence also was higher among female students who had sexual contact with only males (65.6%) than male students who had sexual contact with only females (53.4%) and higher among female students who had no sexual contact (58.9%) than male students who had no sexual contact (52.4%).

The question measuring the prevalence of having had a sunburn was used for the first time in the 2015 national YRBS. As a result, long-term temporal trends are not available for this variable. The prevalence of having had a sunburn did not change significantly from 2015 (55.8%) to 2017 (57.2%).

The question also was not included in the standard questionnaire used in the state and large urban school district surveys in 2017. As a result, the range and median prevalence estimates across states and large urban school districts for the prevalence of having had a sunburn are not available.

#### Have to Avoid Some Foods Because Eating the Food Could Cause an Allergic Reaction

Nationwide, 15.2% of students have to avoid some foods because eating the food could cause an allergic reaction (e.g., skin rashes, swelling, itching, vomiting, coughing, or trouble breathing) (Supplementary Table 240). The prevalence of having to avoid some foods because eating the food could cause an allergic reaction was higher among female (18.4%) than male (11.9%) students; higher among white female (17.6%), black female (24.1%), and Hispanic female (17.2%) than white male (10.5%), black male (16.6%), and Hispanic male (11.1%) students, respectively; and higher among 9th-grade female (17.3%), 10th-grade female (19.7%), 11th-grade female (18.1%), and 12th-grade female (18.5%) than 9th-grade male (11.5%), 10th-grade male (13.0%), 11th-grade male (10.0%), and 12th-grade male (12.9%) students, respectively. The prevalence of having to avoid some foods because eating the food could cause an allergic reaction was higher among black (20.4%) than white (14.3%) and Hispanic (14.1%) students, higher among black female (24.1%) than white female (17.6%) and Hispanic female (17.2%) students, and higher among black male (16.6%) than white male (10.5%) and Hispanic male (11.1%) students. The prevalence of having to avoid some foods because eating the food could cause an allergic reaction was higher among 10th-grade (16.5%) than 9th-grade (14.5%) and 11th-grade (14.1%) students and higher among 10th-grade male (13.0%) and 12th-grade male (12.9%) than 11th-grade male (10.0%) students.

Analyses based on the question ascertaining sexual identity indicated that nationwide, 14.5% of heterosexual students; 19.6% of gay, lesbian, and bisexual students; and 18.3% of not sure students have to avoid food because eating the food could cause an allergic reaction (Supplementary Table 240). The prevalence of having to avoid some foods because eating the food could cause an allergic reaction was higher among gay, lesbian, and bisexual (19.6%) than heterosexual (14.5%) students. The prevalence also was higher among heterosexual female (17.9%) than heterosexual male (11.6%) students and higher among not sure female (23.0%) than not sure male (11.4%) students.

Analyses based on the question ascertaining the sex of sexual contacts indicated that nationwide, 15.5% of students who had sexual contact with only the opposite sex, 20.2% of students who had sexual contact with only the same sex or with both sexes, and 14.0% of students who had no sexual contact have to avoid some foods because eating the food could cause an allergic reaction (Supplementary Table 240). The prevalence of having to avoid some foods because eating the food could cause an allergic reaction was higher among students who had sexual contact with only the same sex or with both sexes (20.2%) than students who had sexual contact with only the opposite sex (15.5%) and students who had no sexual contact (14.0%). Among male students, the prevalence was higher among those who had sexual contact with only males or with both sexes (18.1%) than those who had no sexual contact (10.9%). The prevalence also was higher among female students who had sexual contact with only males (19.2%) than male students who had sexual contact with only females (12.3%) and higher among female students who had no sexual contact (16.9%) than male students who had no sexual contact (10.9%).

The question measuring the prevalence of having to avoid some foods because eating the food could cause an allergic reaction was used for the first time in the 2015 national YRBS. As a result, long-term temporal trends are not available for this variable. The prevalence of having to avoid some foods because eating the food could cause an allergic reaction did not change significantly from 2015 (16.0%) to 2017 (15.2%).

The question also was not included in the standard questionnaire used in the state and large urban school district surveys in 2017. As a result, the range and median prevalence estimates across states and large urban school districts for the prevalence of having to avoid some foods because eating the food could cause an allergic reaction are not available.

## Discussion

YRBSS is the largest public health surveillance system in the United States monitoring a broad range of health-related behaviors among high school students. In addition, YRBSS has been measuring sexual identity and sex of sexual contacts at the state and local levels longer than any other public health surveillance system in the United States. YRBSS data are used widely to compare the prevalence of health-related behaviors among subpopulations of students, assess trends in health-related behaviors over time, monitor progress toward achieving national health objectives, provide comparable state and large urban school district data, and take public health actions to decrease health-risk behaviors and improve health outcomes among youth. This report provides an update on the prevalence of health-related behaviors among students in grades 9–12 nationwide and across 39 states and 21 large urban school districts. More specifically, it describes nationwide disparities in health-related behaviors by demographic subgroups (defined by sex, race/ethnicity, and grade in school) and sexual minority status (as defined by sexual identity and sex of sexual contacts), describes trends in the overall prevalence of health-related behaviors at the national level, and provides an update on the size of sexual minority subgroups nationwide.

Although the majority of the 16,311,000 students projected to have attended public and private schools in grades 9–12 nationwide in 2017 (*25*) are heterosexual, this report indicates that approximately 391,000 are gay or lesbian, 1,305,000 are bisexual, and 685,000 are not sure of their sexual identity. In addition, approximately 261,000 of all students in grades 9–12 have had sexual contact with only the same sex and 864,000 have had sexual contact with both sexes. These counts are somewhat higher than previously reported (*15*) reflecting both an increase in the size of the overall estimated number of students in grade 9–12 nationwide and social and demographic changes in the sexual minority community (*26*). Sexual minority students are part of every community and are as racially, ethnically, socially, economically, and geographically diverse as their nonsexual minority peers.

### Comparison of the Prevalence of Health-Related Behaviors Among Subpopulations of Students

YRBSS is designed to identify how health-related behaviors vary by subpopulations of high school students. Understanding these variations (or lack of variation) in health-related behaviors might help design, target, and identify the impact of school and community policies, programs, and practices. However, isolating the effects of demographic subgroups ascertained by the YRBSS from the effects of socioeconomic status (SES) or culture on the prevalence of health-related behaviors is not possible. For example, in a national study, the likelihood of cardiovascular disease risks such as obesity, sedentary behaviors, and tobacco exposure increased among adolescents aged 12–17 years as their SES based on a poverty-income ratio decreased (*27*).

#### Variations by Sex

The prevalence of most health-related behaviors varies by sex. For example, the prevalence of three of the five injury-related behaviors (rarely or never wearing a seatbelt, having driven when they had been drinking alcohol, and having driven when they had been using marijuana) was higher among male than female students. The prevalence of six of the 13 violence-related behaviors (having carried a weapon, having carried a weapon on school property, having carried a gun, having been threatened or injured with a weapon on school property, having been in a physical fight, and having been in a physical fight on school property) also was higher among male than female students. However, the prevalence of six other violence-related behaviors (having been electronically bullied, having been bullied on school property, having been forced to have sexual intercourse, having experienced sexual violence by anyone, having experienced sexual dating violence, and having experienced physical dating violence) was higher among female than male students. The prevalence of all five suicide-related behaviors (having felt sad or hopeless, having seriously considered attempting suicide, having made a suicide plan, having attempted suicide, and having made a suicide attempt resulting in an injury, poisoning, or overdose that had to be treated by a doctor or nurse) also was higher among female than male students. The prevalence of having ridden with a driver who had been drinking alcohol, having texted or e-mailed while driving, and having not gone to school because of safety concerns did not vary by sex.

The prevalence of 17 of the 20 tobacco use risk behaviors (having ever tried cigarette smoking; having first tried cigarette smoking before age 13 years; current cigarette use; having smoked more than 10 cigarettes per day; having ever used an electronic vapor product; current, current frequent, and current daily electronic vapor product use; current, current frequent, and current daily smokeless tobacco use; current, current frequent, and current daily cigar use; current cigarette or cigar use; current cigarette, cigar, or smokeless tobacco use; and current cigarette, cigar, smokeless tobacco, or current electronic vapor product use) was higher among male than female students. However, among all the tobacco-use behaviors, the only behavior that was health promoting (having tried to quit using all tobacco products) had a higher prevalence among female than male students. The prevalence of three tobacco use behaviors (current frequent and current daily cigarette use and having usually gotten their own electronic vapor products by buying them in a store) did not vary by sex.

The prevalence of 11 of the 20 alcohol and other drug use behaviors (having drunk alcohol for the first time before age 13 years; having reported 10 or more as the largest number of alcoholic drinks in a row; having tried marijuana for the first time before age 13 years; having ever used cocaine, heroin, methamphetamines, ecstasy, and hallucinogenic drugs; having ever taken steroids without a doctor’s prescription; having ever injected any illegal drug; and having been offered, sold, or given an illegal drug on school property) was higher among male than female students. However, the prevalence of having ever drunk alcohol, current alcohol use, and having usually gotten the alcohol they drank by someone giving it to them was higher among female than male students. Six alcohol and other drug use behaviors (current binge drinking, having ever used marijuana, current marijuana use, having ever used synthetic marijuana, having ever used inhalants, and having ever taken prescription pain medicine without a doctor’s prescription or differently than how a doctor told them to use it) did not vary by sex.

The prevalence of four of the six sexual risk behaviors (having ever had sexual intercourse, having had sexual intercourse before age 13 years, having had sexual intercourse with four or more persons, and having drunk alcohol or used drugs before last sexual intercourse) and one of the six protective sexual behaviors (having used a condom during last sexual intercourse) was higher among male than female students. The prevalence of three protective sexual behaviors (having used an IUD or implant before last sexual intercourse; having used a shot, patch, or birth control ring before last sexual intercourse; and having used birth control pills; an IUD or implant; or a shot, patch, or birth control ring before last sexual intercourse) was higher among female than male students, while one sexual risk behavior (not having used any method to prevent pregnancy) also was higher among female than male students. Similarly, the prevalence of having ever been tested for HIV was higher among female than male students. The prevalence of being currently sexually active; having used birth control pills before last sexual intercourse; and having used both a condom during last sexual intercourse and birth control pills; an IUD or implant; or a shot, patch, or birth control ring before last sexual intercourse did not vary by sex.

The prevalence of 18 of the 26 dietary behaviors (not having eaten fruit or drunk 100% fruit juices; having eaten fruit or drunk 100% fruit juices one or more times, two or more times, and three or more times per day; not having eaten vegetables; having eaten vegetables two or more times and three or more times per day; having drunk one or more glasses, two or more glasses, and three or more glasses of milk per day; having drunk soda or pop one or more times, two or more times, and three or more times per day; having drunk a sports drink one or more times, two or more times, and three or more times per day; not having drunk plain water; and having eaten breakfast on all 7 days) was higher among male than female students. In contrast, the prevalence of only three dietary behaviors (not having drunk milk, not having drunk soda or pop, and not having drunk a sports drink) was higher among female than male students. The prevalence of having eaten vegetables one or more times per day; having drunk plain water one or more times, two or more times, and three or more times per day; and having not eaten breakfast did not vary by sex.

The prevalence of all six protective physical activity behaviors (having been physically active for a total of at least 60 minutes per day on 5 or more days, having been physically active for a total of at least 60 minutes per day on all 7 days, having done exercises to strengthen or tone their muscles on 3 or more days, going to PE classes on 1 or more days, going to PE classes on all 5 days, and having played on at least one sports team), and one of the four physical activity risk behaviors (having had a concussion one or more times from playing a sport or being physically active) was higher among male than female students. Only one physical activity risk behavior (were not physically active for a total of at least 60 minutes on at least 1 day) had a higher prevalence estimate among female than male students. The prevalence of playing video or computer games or using a computer 3 or more hours per day and watching television 3 or more hours per day did not vary by sex.

The prevalence of obesity was higher among male than female students, whereas the prevalence of being overweight, describing themselves as overweight, and trying to lose weight was higher among female than male students. The prevalence of having never seen a dentist was higher among male than female students, whereas the prevalence of having seen a dentist during the 12 months before the survey, indoor tanning device use, having had a sunburn, and having to avoid some foods because eating the food could cause an allergic reaction was higher among female than male students. The prevalence of having ever been told they have asthma and getting 8 or more hours of sleep did not vary by sex.

#### Variations by Race/Ethnicity

The prevalence of most health-related behaviors varies by race/ethnicity. The prevalence of 25 behaviors (17 risk and eight protective) was higher among white than black and Hispanic students, the prevalence of 21 behaviors (19 risk and two protective) was higher among black than white and Hispanic students, and the prevalence of 11 risk behaviors was higher among Hispanic than white and black students. Twenty-four behaviors (17 risk and seven protective) did not vary by race/ethnicity.

White students had a higher prevalence than black and Hispanic students of one injury-related risk behavior (having texted or e-mailed while driving), three violence-related risk behaviors (having carried a weapon, having been electronically bullied, and having been bullied on school property), 12 tobacco-use related risk behaviors (current, current frequent, and current daily cigarette use; current, current frequent, and current daily electronic vapor product use; current, current frequent, and current daily smokeless tobacco use; current cigarette or cigar use; current cigarette, cigar, or smokeless tobacco use; and current cigarette, cigar, smokeless tobacco, or electronic vapor product use), three protective sexual behaviors (having used birth control pills before last sexual intercourse; having used birth control pills, an IUD or implant, or a shot, patch, or birth control ring before last sexual intercourse; and having used both a condom during last sexual intercourse and birth control pills, an IUD or implant, or a shot, patch, or birth control ring before last sexual intercourse), four protective dietary behaviors (having eaten vegetables one or more times per day, not having drunk a sports drink, having drunk plain water one or more times per day, and having eaten breakfast on all 7 days), one additional protective behavior (having seen a dentist during the 12 months before the survey) and one additional risk behavior (having had a sunburn).

Black students had a higher prevalence than white and Hispanic students of one injury-related risk behavior (rarely or never wearing a seatbelt), four violence-related risk behaviors (having been threatened or injured with a weapon on school property, having been in a physical fight, having been in a physical fight on school property, and having experienced physical dating violence), two sexual risk behaviors (having had sexual intercourse before age 13 years and having had sexual intercourse with four or more persons), having ever been tested for HIV, nine dietary risk behaviors (not having eaten fruit or drunk 100% fruit juices; not having eaten vegetables; not having drunk milk; having drunk soda or pop two or more times and three or more times per day; having drunk a sports drink one or more times, two or more times, and three or more times per day; and not having drunk plain water), one protective dietary behavior (having eaten fruit or drunk 100% fruit juices three or more times per day), one physical activity risk behavior (watching television 3 or more hours per day), and two additional risk behaviors (having ever been told they have asthma and having to avoid some foods because eating the food could cause an allergic reaction).

Hispanic students had a higher prevalence than white and black students of two injury-related risk behaviors (having ridden with a driver who had been drinking alcohol and having driven when they had been drinking alcohol), one suicide-related risk behavior (having felt sad or hopeless), one tobacco use-related risk behavior (having ever used an electronic vapor product), five risk behaviors related to alcohol and other drug use (having drunk alcohol before age 13 years; having ever used synthetic marijuana; having ever used cocaine; having ever used ecstasy; and having been offered, sold or given an illegal drug on school property), and two weight control-related behaviors (describing themselves as overweight and trying to lose weight).

The prevalence of some health-related behaviors did not vary by race/ethnicity: one injury-related risk behavior (having driven when using marijuana), four violence-related risk behaviors (having carried a weapon on school property, having carried a gun, having been forced to have sexual intercourse, and having experienced sexual violence by anyone), one suicide-related risk behavior (having made a suicide plan), five tobacco-related risk behaviors (having first tried cigarette smoking before age 13 years, having smoked more than 10 cigarettes per day, having usually gotten their own electronic vapor products by buying them in a store, and current frequent and current daily cigar use), three risk behaviors related to alcohol and other drug use (having usually gotten the alcohol they drank by someone giving it to them, having ever used methamphetamines, and having ever injected any illegal drug), two sexual risk behaviors (being currently sexually active and having drunk alcohol or used drugs before last sexual intercourse), one protective sexual behavior (having used a condom during last sexual intercourse), three protective dietary behaviors (having eaten fruit or drunk 100% fruit juices one or more times per day, having eaten vegetables two or more times per day, and not having drunk soda or pop), two protective physical activity-related behaviors (having been physically active for a total of at least 60 minutes per day on all 7 days and having done exercises to strengthen or tone their muscles on 3 or more days), one physical activity risk behavior (having had a concussion one or more times from playing a sport or being physically active), and one additional protective behavior (getting 8 or more hours of sleep).

#### Variations by Sexual Identity and Sex of Sexual Contacts

The prevalence of most health-related behaviors varies by sexual identity and sex of sexual contacts. However, unlike the variations by sex and race/ethnicity, this report documents that the differences are almost always in the same direction with sexual minority students having a higher prevalence of health-risk behaviors compared with nonsexual minority students. For example, across the 13 violence-related risk behaviors, the prevalence of 10 was higher among gay, lesbian, and bisexual students than heterosexual students and the prevalence of nine was higher among students who had sexual contact with only the same sex or with both sexes than students who had sexual contact with only the opposite sex. The prevalence for five of these behaviors (having been electronically bullied, having been forced to have sexual intercourse, having experienced sexual violence by anyone, having experienced sexual dating violence, and having experienced physical dating violence) was twofold or greater for gay, lesbian, and bisexual students compared with heterosexual students and the prevalence for four of these same behaviors (having been forced to have sexual intercourse, having experienced sexual violence by anyone, having experienced sexual dating violence, and having experienced physical dating violence) was twofold or greater for students who had sexual contact with only the same sex or with both sexes than students who had sexual contact with only the opposite sex. Similarly, across the five suicide-related risk behaviors, the prevalence of all five was higher among gay, lesbian, and bisexual students than heterosexual students and higher among students who had sexual contact with only the same sex or with both sexes than students who had sexual contact with only the opposite sex. The prevalence of all five of these behaviors (having felt sad or hopeless; having seriously considered attempting suicide; having made a suicide plan; having attempted suicide; and having made a suicide attempt resulting in an injury, poisoning, or overdose that had to be treated by a doctor or nurse) was twofold or greater for gay, lesbian, and bisexual students compared with heterosexual students and the prevalence for four of these same behaviors (having seriously considered attempting suicide; having made a suicide plan; having attempted suicide; and having made a suicide attempt resulting in an injury, poisoning, or overdose that had to be treated by a doctor or nurse) was twofold or greater for students who had sexual contact with only the same sex or with both sexes than students who had sexual contact with only the opposite sex.

Across the 19 tobacco use-related risk behaviors, the prevalence of 11 was higher among gay, lesbian, and bisexual students than heterosexual students and the prevalence of 12 was higher among students who had sexual contact with only the same sex or with both sexes than students who had sexual contact with only the opposite sex. The prevalence for three of these behaviors (current, current frequent, and current daily cigarette use) was twofold or greater for gay, lesbian, and bisexual students compared with heterosexual students and the prevalence for four of these behaviors (current frequent and current daily cigarette use and current frequent and current daily cigar use) was twofold or greater for students who had sexual contact with only the same sex or with both sexes than students who had sexual contact with only the opposite sex.

Similarly, across the 19 risk behaviors related to alcohol and other drug use, the prevalence of 18 was higher among gay, lesbian, and bisexual students than heterosexual students and the prevalence of 16 was higher among students who had sexual contact with only the same sex or with both sexes than students who had sexual contact with only the opposite sex. The prevalence for eight of these behaviors (having ever used synthetic marijuana, inhalants, heroin, methamphetamines, ecstasy, and hallucinogenic drugs; having ever taken steroids without a doctor’s prescription, and having ever injected any illegal drug) was twofold or greater for gay, lesbian, and bisexual students compared with heterosexual students and the prevalence for eight of these behaviors (having ever used cocaine, inhalants, heroin, methamphetamines, ecstasy, and hallucinogenic drugs; having ever taken steroids without a doctor’s prescription, and having ever injected any illegal drug) was twofold or greater for students who had sexual contact with only the same sex or with both sexes than students who had sexual contact with only the opposite sex.

The same pattern also was evident across the six sexual risk behaviors. The prevalence of five of these behaviors was higher among gay, lesbian, and bisexual students than heterosexual students and the prevalence of three of these behaviors was higher among students who had sexual contact with only the same sex or with both sexes than students who had sexual contact with only the opposite sex. The prevalence for two of these behaviors (having had sexual intercourse before age 13 years and not having used any method to prevent pregnancy) was twofold or greater for gay, lesbian, and bisexual students compared with heterosexual students.

In contrast, no clear pattern of differences by sexual identity or sex of sexual contact subgroups was detected for dietary behaviors, physical activity, and other health-related behaviors. However, the prevalence of having never seen a dentist was twofold or greater for students who had sexual contact with only the same sex or with both sexes than students who had sexual contact with only the opposite sex.

This report also demonstrates that some students are not yet sure of their sexual identity. Not sure students and gay, lesbian, and bisexual students often have a similar prevalence of many health-risk behaviors. For example, not sure students and gay, lesbian, and bisexual students had a similar prevalence for all five of the injury-related risk behaviors, eight of the 13 violence-related risk behaviors, 12 of the 19 tobacco use-related risk behaviors, 11 of the 19 risk behaviors related to alcohol and other drug use, three of the six sexual risk behaviors, nine of the 11 dietary risk behaviors, all four physical activity risk behaviors, and four of the five other health-related risk behaviors and obesity and overweight. In addition, not sure students often have a higher prevalence of many health-risk behaviors than heterosexual students. For example, not sure students had a higher prevalence for eight of the 13 violence-related risk behaviors, all five suicide-related risk behaviors, and 10 of the 19 risk behaviors related to alcohol and other drug use.

Students who had no sexual contact have a much lower prevalence of most health-risk behaviors compared with students who had sexual contact with only the opposite sex and students who had sexual contact with only the same sex or with both sexes. For example, the prevalence of all five injury-related risk behaviors, all 13 violence-related risk behaviors, all five suicide-related risk behaviors, all 19 tobacco use-related risk behaviors, all 19 risk behaviors related to alcohol and other drug use, and six of the 11 dietary risk behaviors was higher among students who had sexual contact with only the opposite sex and students who had sexual contact with only the same sex or with both sexes than students who had no sexual contact.

### Assessment of Trends in Health-Related Behaviors Over Time

Because YRBSS has been implemented since 1991, YRBSS data can be used to assess both long-term temporal trends (i.e., as long as 26 years) and more recent 2-year changes in most of the health-related behaviors included in this report. Although this report describes many overall long-term temporal trends and 2-year changes in prevalence, a more in-depth trend analysis by demographic subgroups would increase understanding of how to implement effective interventions among the students who need them most. Nonetheless, almost all of the overall trends reflect actual reductions in risk behaviors and potential improvements in health outcomes among high school students nationwide.

For behaviors for which long-term trend data are available, long-term linear decreases occurred in the prevalence of three of the four injury-related risk behaviors (rarely or never wearing a seatbelt, having ridden with a driver who had been drinking alcohol, and having driven when they had been drinking alcohol). Long-term linear decreases also occurred in the prevalence of eight of the 11 violence-related risk behaviors (having carried a weapon, having carried a weapon on school property, having been threatened or injured with a weapon on school property, having been in a physical fight, having been in a physical fight on school property, having been forced to have sexual intercourse, having experienced sexual dating violence, and having experienced physical dating violence), whereas a long-term linear increase was identified in the prevalence of having not gone to school because of safety concerns. A linear decrease occurred in the prevalence of having carried a weapon from 1991–2017; however, based on significant quadratic trends, no change has occurred since 1997. In addition, long-term linear decreases occurred in the prevalence of three of the five suicide-related risk behaviors (having seriously considered attempting suicide, having made a suicide plan, and having attempted suicide), whereas a long-term linear increase occurred in the prevalence of having felt sad or hopeless. Despite the linear decreases in the prevalence of having seriously considered suicide and having made a suicide plan, based on significant quadratic trends, having seriously considered attempting suicide increased since 2007 and having made a suicide plan increased since 2009. No long-term trends occurred in the prevalence of one injury-related risk behavior (having texted or e-mailed while driving), two violence-related risk behaviors (having been electronically bullied and having been bullied on school property), and one suicide-related risk behavior (having made a suicide attempt resulting in an injury, poisoning, or overdose that had to be treated by a doctor or nurse).

Long-term linear decreases occurred in the prevalence of seven of the nine tobacco use-related risk behaviors (having ever tried cigarette smoking; current, current frequent, and current daily cigarette use; having smoked more than 10 cigarettes per day; current cigar use; and current cigarette or cigar use). No long-term linear trends occurred in the prevalence of current frequent and current daily cigar use. However, based on significant quadratic trends, current frequent cigar use increased from 1997–2013 and then decreased from 2013–2017 and current daily cigar use increased from 1997–2011 and then decreased from 2011–2017.

Long-term linear decreases occurred in the prevalence of 13 of the 17 risk behaviors related to alcohol and other drug use (having ever drunk alcohol; having drunk alcohol for the first time before age 13 years; current alcohol use; having reported 10 or more as the largest number of drinks in a row; having tried marijuana for the first time before age 13 years; having ever used cocaine, inhalants, heroin, methamphetamines, ecstasy, and hallucinogenic drugs; having ever injected an illegal drug; and having been offered, sold, or given an illegal drug on school property). Although no long-term linear trends occurred for three additional behaviors related to alcohol and other drug use, based on significant quadratic trends, the prevalence of having ever used marijuana increased from 1991–1997 and then decreased from 1997–2017, current marijuana use increased from 1991–1995 and then decreased from 1995–2017, and having ever taken steroids without a doctor’s prescription increased from 1991–2001 and then decreased from 2001–2017. No long-term trends (linear or quadratic) occurred in the prevalence of having usually gotten the alcohol they drank by someone giving it to them.

Long-term linear decreases occurred in the prevalence of all six sexual risk behaviors (having ever had sexual intercourse, having had sexual intercourse before age 13 years, having had sexual intercourse with four or more persons, being currently sexually active, not having used any method to prevent pregnancy, and having drunk alcohol before last sexual intercourse), whereas long-term linear increases occurred in the prevalence of four of the six protective sexual behaviors (having used a condom during last sexual intercourse; having used birth control pills before last sexual intercourse; having used an IUD or implant before last sexual intercourse; and having used birth control pills, an IUD or implant, or a shot, patch, or birth control ring before last sexual intercourse). However, based on significant quadratic trends, the prevalence of having used a condom during last sexual intercourse has decreased since 2005 and not having used any method to prevent pregnancy has not changed since 2007. In addition, a significant linear decrease occurred in the prevalence of having ever been tested for HIV.

Although a long-term linear increase occurred in the prevalence of not having drunk soda or pop and long-term linear decreases occurred in the prevalence of having drunk soda or pop one or more times, two or more times, and three or more times per day (improvements in dietary behaviors), long-term linear increases occurred in the prevalence of not having eaten vegetables and not having drunk milk and long-term linear decreases occurred in the prevalence of having eaten fruit or drunk 100% fruit juices three or more times per day, having eaten vegetables one or more times per day, and having drunk one or more glasses, two or more glasses, and three or more glasses of milk per day (worsening dietary behaviors). No long-term linear or quadratic trends occurred in the prevalence of not having eaten fruit or drunk 100% fruit juices, having eaten fruit or drunk 100% fruit juices one or more times and two or more times per day, having eaten vegetables two or more times and three or more times per day, having not eaten breakfast, and having eaten breakfast on all 7 days.

A long-term linear increase occurred in the prevalence of having done exercises to strengthen or tone their muscles on 3 or more days; however, based on significant quadratic trends, the prevalence of having done exercises to strengthen or tone their muscles on 3 or more days has not changed since 2011. Although a long-term linear decrease occurred in the prevalence of watching television 3 or more hours per day, this decrease in sedentary behavior might have been offset by a long-term linear increase in the prevalence of playing video or computer games or using a computer 3 or more hours per day. No long-term trends occurred in the prevalence of six of the nine behaviors related to physical activity (not having been physically active for a total of at least 60 minutes on at least 1 day, having been physically active for a total of at least 60 minutes per day on 5 or more days, having been physically active for a total of at least 60 minutes per day on all 7 days, going to PE classes on 1 or more days, going to PE classes on all 5 days, and having played on at least one sports team).

Long-term linear increases occurred in the prevalence of obesity, overweight, trying to lose weight, and having ever been told they have asthma; however, based on significant quadratic trends, no change has occurred in the prevalence of having ever been told they have asthma since 2009. Long-term linear decreases occurred in the prevalence of getting 8 or more hours of sleep and indoor tanning device use. No long-term linear trend occurred in the prevalence of describing themselves as overweight, though a significant quadratic trend indicated that describing themselves as overweight decreased from 1991–1995 and then increased from 1995–2017.

### Monitor Progress Toward Achieving National Health Objectives

The national YRBS is the primary source of data to measure 21 *Healthy People 2020* objectives, including one leading health indicator (*28*). The *Healthy People 2020* objectives provide a comprehensive agenda for improving the health of all persons in the United States during 2011–2020. This report provides the *Healthy People 2020* targets and data from the 2017 national YRBS for 16 of the 21 objectives (Supplementary Table 241). Because of changes in the questions included in the 2017 national YRBS or changes in question wording, 2017 data are not available for five objectives. The data indicate that as of 2017, eight of the 16 objectives have been achieved, which is one more than the number met when the 2015 national YRBS data were reported in 2016 (*15*) and twice the number met when the 2013 national YRBS data were reported in 2014 (*29*). *Healthy People 2020* objective AH-7 is to reduce the proportion of adolescents who have been offered, sold, or given an illegal drug on school property to ≤20.4%. During 2017, 19.8% of high school students nationwide had been offered, sold, or given an illegal drug on school property during the 12 months before the survey. This is the first time this objective has been met. *Healthy People 2020* objective C-20.3 is to reduce the proportion of adolescents in grades 9–12 who report using artificial sources of ultraviolet light for tanning to ≤14.0%. During 2017, 5.6% of high school students nationwide had used an indoor tanning device (e.g., sunlamp, sunbed, or tanning booth) one or more times during the 12 months before the survey. *Healthy People 2020* objective IVP-34 is to reduce physical fighting among adolescents to ≤28.4%. During 2017, 23.6% of high school students nationwide had been in a physical fight one or more times during the 12 months before the survey. *Healthy People 2020* objective IVP-36 is to reduce weapon carrying by adolescents on school property to ≤4.6%. During 2017, 3.8% of high school students nationwide had carried a weapon on school property on at least 1 day during the 30 days before the survey. *Healthy People 2020* objective PA-8.2.3 is to increase the proportion of adolescents in grades 9–12 who view television, watch videos, or play video games for no more than 2 hours per day. During 2017, 79.3% of high school students nationwide watched television for no more than 2 hours per day on an average school day. *Healthy People 2020* objective SA-1 is to reduce the proportion of adolescents who report that they rode, during the previous 30 days, with a driver who had been drinking alcohol to ≤25.5%. During 2017, 16.5% of high school students nationwide had ridden one or more times during the 30 days before the survey in a car or other vehicle driven by someone who had been drinking alcohol. *Healthy People 2020* objective TU-2.2 is to reduce the proportion of adolescents who use cigarettes during the past 30 days to ≤16.0%. During 2017, 8.8% of high school students smoked cigarettes on at least 1 day during the 30 days before the survey. *Healthy People 2020* objective TU-2.4 is to reduce the proportion of adolescents who use cigars during the past 30 days to ≤8.0%. During 2017, 8.0% of high school students smoked cigars, cigarillos, or little cigars on at least 1 day during the 30 days before the survey. This is the first time this objective has been met.

To meet additional *Healthy People 2020* objectives, changes in school and community policies, programs, and practices might be needed. For example, *Healthy People 2020* objective IVP-35 is to reduce bullying among adolescents to ≤17.9%. During 2017, 19.0% of high school students nationwide were bullied on school property during the 12 months before the survey. Similarly, *Healthy People 2020* objective SH-3 is to increase the proportion of students in grades 9–12 who get sufficient sleep to ≥33.2%. During 2017, 25.4% of high school students nationwide got 8 or more hours of sleep on an average school night. The 2015 and 2017 prevalence estimates for both of these objectives were not significantly different suggesting that more work might be needed to address these issues.

### Provide Comparable State and Large Urban School District Data

One of the strengths of YRBSS is that it provides not just national but state and large urban school district data. These data are more likely to be used to develop, improve, and evaluate state and local policies, programs, and practices because they reflect a more relevant population for local stakeholders and decision makers than national data. Because participating states and large urban school districts use similar sampling designs, questionnaires, data collection strategies, and data processing procedures, their YRBS data can be compared which provides even more information to guide decision making about public health interventions that can help reduce health-risk behaviors among youth.

Across states, a range of 25 or more percentage points or a fivefold variation or greater was identified for the following 19 behaviors:

- having texted or e-mailed while driving (minimum: 27.4%; maximum: 55.2%);
- current frequent cigarette use (minimum: 0.4%; maximum: 5.5%);
- current daily cigarette use (minimum: 0.3%; maximum: 4.5%);
- having smoked more than 10 cigarettes per day (minimum: 2.3%; maximum: 18.1%);
- current frequent smokeless tobacco use (minimum: 0.6%; maximum: 5.8%);
- current daily smokeless tobacco use (minimum: 0.4%; maximum: 5.1%);
- current frequent cigar use (minimum: 0.4%; maximum: 2.9%);
- current daily cigar use (minimum: 0.3%; maximum: 2.4%);
- having ever drunk alcohol (minimum: 30.4%; maximum: 68.0%);
- having ever used marijuana (minimum: 16.6%; maximum: 44.1%);
- having ever used heroin (minimum: 1.2%; maximum: 9.6%);
- having ever used methamphetamines (minimum: 1.7%; maximum: 10.5%);
- having ever injected any illegal drug (minimum: 1.4%; maximum: 8.0%);
- having used an IUD or implant before last sexual intercourse (minimum: 1.9%; maximum: 13.3%);
- having used birth control pills; an IUD or implant; or a shot, patch, or birth control ring before last sexual intercourse (minimum: 20.9%; maximum: 50.2%);
- having drunk one or more glasses of milk per day (minimum: 19.8%; maximum: 48.3%);
- going to PE classes on 1 or more days (minimum: 27.9%; maximum: 91.5%);
- going to PE classes on all 5 days (minimum: 5.8%; maximum: 68.4%); and
- having never seen a dentist (minimum: 0.9%; maximum: 4.7%).

Across large urban school districts, a range of 25 or more percentage points or a fivefold variation or greater was identified for the following 13 behaviors:

- current frequent cigarette use (minimum: 0.1%; maximum: 1.4%);
- current daily cigarette use (minimum: 0.1%; maximum: 0.8%);
- current frequent electronic vapor product use (minimum: 0.4%; maximum: 2.5%);
- current daily electronic vapor product use (minimum: 0.1%; maximum: 1.9%);
- current daily smokeless tobacco use (minimum: 0.1%; maximum: 1.2%);
- having ever drunk alcohol (minimum: 38.2%; maximum: 64.8%);
- having ever used heroin (minimum: 1.3%; maximum: 7.6%);
- having ever had sexual intercourse (minimum: 21.7%; maximum: 49.2%);
- having used an IUD or implant before last sexual intercourse (minimum: 0.7%; maximum: 10.4%);
- having used a shot, patch, or birth control ring before last sexual intercourse (minimum: 0.0%; maximum: 9.3%);
- having ever been tested for HIV (minimum: 10.2%; maximum: 37.2%);
- going to PE classes on 1 or more days (minimum: 28.0%; maximum: 86.1%); and
- going to PE classes on all 5 days (minimum: 7.1%; maximum: 43.5%).

All these substantial differences across states and large urban school districts might reflect differences in state and local laws and policies, enforcement practices, access to drugs, availability of effective school and community interventions, prevailing behavioral and social norms (including attitudes toward sexual minorities), the amount of stigma and discrimination, demographic characteristics of the population, and adult practices and health-related behaviors. Positive changes in one or more of these factors might contribute to important reductions in health-risk behaviors within and across states and large urban school districts among students in grades 9–12.

### Take Public Health Action

Most high school students cope with the transition from childhood through adolescence to adulthood successfully and become healthy and productive adults. However, this report documents that some subgroups of students defined by sex, race/ethnicity, grade in school, and sexual minority status have a higher prevalence of many health-risk behaviors that might place them at risk for unnecessary or premature mortality, morbidity, and social problems. Sexual minority students in particular struggle because of the disparities in health-related behaviors documented in this report, including violence-related behaviors and alcohol and other drug use, that can be compounded by stigma, discrimination, and homophobia. Because many health-risk behaviors initiated during adolescence often extend into adulthood, they might have life-long negative effects on health outcomes, educational attainment, employment, housing, and overall quality of life.

Schools have a unique and an important role to play in addressing the health-related behaviors of all students, including sexual minority students. In the United States, schools have direct contact with more than 56 million students (*25*) for at least 6 hours a day during 13 key years of their social, physical, and intellectual development. After the family home, schools are the primary places responsible for the development of young persons. This gives schools an opportunity to dramatically improve the health and well-being of their students each day. Research shows that well-designed, well-implemented, school-based prevention programs can significantly reduce health-risk behaviors among all students (*30*) as well as sexual minority students (*31*–*33*).

During 2013–2018, CDC supported schools in implementing prevention programs through two major cooperative agreements. The first, Promoting Adolescent Health Through School-Based HIV/STD Prevention and School-Based Surveillance (http://www.cdc.gov/healthyyouth/fundedpartners/1308/pdf/rfa-1308.pdf), provided funding and technical assistance to the education agency in 18 states and the District of Columbia and to 17 large urban school districts to help schools implement effective policies and practices to reduce sexual risk behaviors among youth. These programs focused partly on adolescents most at risk as part of their HIV, STI, and pregnancy prevention activities. Examples of program activities included the implementation of quality health education, connecting youth to school-linked and school-based health services, and supporting schools in establishing safe and supportive environments. This cooperative agreement also provided funding to 46 states and 21 large urban school districts to conduct the YRBS and School Health Profiles (https://www.cdc.gov/healthyyouth/data/profiles/index.htm).

The second major cooperative agreement, State Public Health Actions to Prevent and Control Diabetes, Heart Disease, Obesity and Associated Risk Factors and Promote School Health (http://www.cdc.gov/chronicdisease/about/state-public-health-actions.htm), provided funding to the health agency in all 50 states and the District of Columbia to reduce the risk factors associated with childhood obesity and to promote the well-being and healthy development of all children and youth. As part of this program, CDC supported use of the following proven strategies in schools: healthier nutrition environments, comprehensive physical activity programs and physical education policies, and improved processes and better training to help students manage chronic conditions. In addition, CDC gives schools well-researched and effective guidance and support to help them improve school health services, policies, and practices. This support helps schools and students manage challenges associated with chronic conditions such as diabetes, asthma, and food allergies. Providing health services in schools helps reduce absences among children with chronic conditions.

In addition, CDC provides resources to help states and communities take advantage of the best available evidence to prevent violence. Specifically, CDC has developed several technical packages containing strategies to prevent or reduce youth violence, sexual violence, dating violence, and suicide (available at https://www.cdc.gov/violenceprevention/pub/technical-packages.html).

YRBS data are a primary data source for monitoring the impact of both of the cooperative agreements described previously at the state and local levels. In addition, health and education agencies and nongovernmental organizations in these jurisdictions use their YRBS data in myriad ways to improve health-related policies, programs, and practices. For example, state and local agencies use YRBS results to inform key stakeholders, help develop local health promotion programs, identify the highest risk behaviors around which programmatic funds should be focused, combine with results from other surveys on health topics, review and set goals for children’s health and wellness, measure long-term outcomes related to certain projects or goals, and demonstrate need for public health funding and grant programs. More specifically, state and local health and education agencies used YRBS results in the following ways:

- The Rhode Island Department of Health and the Rhode Island Department of Education collaboratively developed a Rhode Island Adolescent Sexual Health Profile. The data source for many of the indicators in the profile is the Rhode Island YRBS. The profile was presented to Rhode Island decision-makers and is also being shared with other stakeholders to help guide discussions about policy and program recommendations.
- In Vermont, YRBS results have been instrumental in helping the Vermont Agency of Education and Department of Health to document the need for and support schools in instituting condom availability programs in high schools throughout the state. The two agencies released a joint memo on comprehensive sex education citing YRBS results and encouraging schools to provide access to condoms, which has led to supportive newspaper coverage and discussions within schools and at school board meetings.
- The New York City Department of Education’s Office of School Wellness Programs routinely uses YRBS results during its professional development workshops and trainings for health and physical education teachers. During these trainings, YRBS results are used to inform educators about students’ health-related behaviors. The New York City Teens Connection, a program of the Department of Health and Mental Hygiene’s Center for Health Equity, relies heavily on YRBS results to support its teen pregnancy and STI prevention work. The New York City Teens Connection works through local and citywide partnerships to provide evidence-based sexual health education programs and access to health care for youth. To recruit implementation partners (e.g., community-based organizations, schools, clinics, and citywide agencies), the New York City Teens Connection routinely presents YRBS results on adolescent sexual behaviors to demonstrate the need for sexual health education programs and access to health care.
- San Diego Unified School District used their YRBS results to help determine which sexual health curriculum would best meet the needs of students; revise curriculum to include up-to-date information on sexual orientation, sexual behaviors, and harassment; educate parents, caregivers, and community members about the importance of the new curriculum via community forum presentations and panel discussions; and revise their sexual health instruction training for district teachers.
- The State of Alaska Obesity Prevention and Control Program makes extensive use of YRBS results to support their Play Every Day public education campaign, describe the burden of childhood obesity, and document the need to increase physical activity in Alaska schools. YRBS results were helpful in passing the Physical Activity in Schools Law that requires every student in grades K–8 be provided with opportunities for 54 minutes (90% of the recommended 60 minutes) of physical activity every school day.
- The Montana Office of Public Instruction’s tobacco use prevention trainings for school administrators and faculty feature their YRBS results on electronic vapor product use along with examples of the products themselves, how easily they can be concealed in school, and the health risks produced by electronic vapor product use. The Montana Department of Public Health and Human Services ran a public education campaign that included television commercials featuring Montana YRBS results on electronic vapor product use.
- North Dakota YRBS results are used by the North Dakota Center for Tobacco Prevention and Control Policy, also known as BreatheND, to support a public education campaign designed to prevent tobacco use among youth and exposure to second hand smoke. North Dakota’s YRBS results documented a statewide decrease in cigarette use among high school students that they attribute in part to this public education campaign.
- New Hampshire YRBS results revealed a real need for a program on distracted driving and informed regional efforts on suicide prevention and dating violence prevention efforts in high schools for both students and parents. In addition, a prevention program called Life of an Athlete was brought into a region’s high schools because of their YRBS results on substance use. Student teams reviewed YRBS results to help inform the type of outreach and other activities they will conduct throughout the school year to specifically address the attitudes around substance misuse and student misperceptions about the prevalence of use.

CDC and other federal agencies use YRBS data in various reports and publications including State Health Profiles (*34*); Indicators for Chronic Disease Surveillance (*35*); America’s Children: Key National Indicators of Well-Being (*36*); Prevention Status Reports (*37*); Indicators of School Crime and Safety (*38*); and Nutrition, Physical Activity, and Obesity: Data, Trends and Maps (*39*). Each of these reports and other similar reports using YRBS data are intended to stimulate support for and improvements in public health interventions.

## Limitations

The findings in this report are subject to at least eight limitations. First, these data apply only to youth who attend school and therefore are not representative of all persons in this age group. Nationwide, in 2013, of persons aged 16–17 years, approximately 5% were not enrolled in high school and lacked a high school credential (*40*). However, sexual minority youth might represent a disproportionate percentage of high school dropouts and other youths who are absent from or do not attend school (*41*). Second, the extent of underreporting or overreporting of health-related behaviors cannot be determined, although the survey questions demonstrate good test-retest reliability (*18*,*19*). Third, some students might not have known their sexual identity; might have been unwilling to disclose it on the YRBS questionnaire; might have been unwilling to label themselves as heterosexual, gay, lesbian, or bisexual; or might not have understood the sexual identity question. Although the “not sure” response option for the sexual identity question is a credible choice for youth who might truly be unsure of their sexual identity at this point in their lives, this response option might have been selected by students who did not know what the question or the other response options meant. Nonetheless, evidence that the words used to describe various types of sexual identity are unclear to youth is not available. Fourth, because no definition was provided for sexual contact, students likely considered a range of sexual activities when responding to this question, possibly including involuntary activities. Fifth, the questions used to ascertain sexual minority status focused only on sexual identity and sex of sexual contacts. Questions focused on sexual attraction or gender identity might have identified a different subgroup of sexual minority students and different estimates of the prevalence of health-related behaviors. Sixth, BMI is calculated on the basis of self-reported height and weight, and therefore tends to underestimate the prevalence of obesity and overweight (*19*). Seventh, not all states and large urban school districts included all of the standard questions on their YRBS questionnaire; therefore, data for certain variables are not available for some sites. Finally, these analyses are based on cross-sectional surveys and can only provide an indication of association, not causality.

## Conclusion

YRBSS is an ongoing source of high-quality data at the national, state, and large urban school district levels for monitoring health-related behaviors that contribute to the leading causes of mortality and morbidity among youth and adults in the United States. In 2017, in addition to the national data, 39 states and 21 large urban school districts obtained data representative of their high school students. Questionnaires for the national survey, 30 of the 39 state surveys, and all 21 large urban school district surveys included a question to ascertain sexual identity, sex of sexual contacts, or both.

YRBSS data are an important tool for planning, implementing, and evaluating public health policies, programs, and practices. Although beyond the scope of this report, a particular strength of YRBSS (as compared with more narrowly focused surveys) is that it allows analysis of the interrelationships among health-related behaviors (e.g., how alcohol and other drug use is associated with sexual behaviors). Similarly, because of its long history and consistent methodology, YRBSS can identify not only national long-term temporal trends in health-related behaviors overall as described in this report but also long-term trends among demographic subgroups of students (e.g., by sex or race/ethnicity) and long-term temporal trends at the state and large urban school district levels. These trend analyses are particularly valuable for understanding the impact of broad public health and school health policies and practices designed to improve the health outcomes of students over time.

This report documents important disparities in health-related behaviors among subgroups of students defined by sex, race/ethnicity, and grade in school and experienced by sexual minority students. Using this and other reports based on scientifically sound data is important to raise awareness about the prevalence of health-related behaviors among students in grades 9–12, especially sexual minority students, among decision-makers, the public, and a wide variety of agencies and organizations that work with youth. These agencies and organizations, including schools and youth-friendly health care providers, can help facilitate access to critically important education, health care, and evidence-based interventions.

To maintain the quality of YRBSS data, enhanced training and technical assistance for participating state and local health and education agencies, an increase in the number of states with representative data, more substate surveys at the county- or school district-level, and more universal use of all standard YRBSS questions are needed. Because sexual minority students represent a relatively small proportion of all students, use of large, population-based samples of students is key to obtaining the most generalizable and highest quality data on which to base policy and programmatic decisions that can help eliminate the health-related behavior disparities and improve health status, educational outcomes, and overall quality of life for this population as well as all other youth.

## Footnotes

* A questionnaire that fails quality control has <20 remaining responses after editing or has the same answer to ≥15 consecutive questions.

† Overall response rate = (number of participating schools/number of eligible sampled schools) x (number of usable questionnaires/number of eligible students sampled).

§ The prevalence of driving a car or other vehicle during the 30 days before the survey varies slightly for the three variables (having driven when they had been drinking alcohol, having driven when they had been using marijuana, and having texted or e-mailed while driving) because of differences in the number of students who selected the response option “I did not drive a car or other vehicle during the past 30 days” for each question.

¶ The prevalence of dating or going out with someone during the 12 months before the survey varies slightly for the two variables (having experienced sexual dating violence and having experienced physical dating violence) because of differences in the number of students who selected the response option “I did not date or go out with anyone during the past 12 months” for each question.

** Review of only the oldest and most recent data points are not necessarily indicative of long-term temporal trends because the logistic regression analyses take into account all data points and adjust for changes in sex, grade, and race/ethnicity over time.

†† Pellet-sized pieces of highly purified cocaine.

§§ A process in which cocaine is dissolved in ether or sodium hydroxide and the precipitate is filtered off.
